# Simple Synthetic Approach to *N*-(Pyridin-2-yl)imidates from Nitrostyrenes and 2-Aminopyridines via the *N*-(Pyridin-2-yl)iminonitriles as Intermediates

**DOI:** 10.3390/molecules28083321

**Published:** 2023-04-09

**Authors:** Andriani G. Chaidali, Ioannis N. Lykakis

**Affiliations:** Department of Chemistry, Aristotle University of Thessaloniki, University Campus, 54124 Thessaloniki, Greece; chaidali.and@gmail.com

**Keywords:** imidates, iminonitriles, nitrostyrenes, 2-aminopyridine, *N*-heterocycles

## Abstract

A facile, green, synthetic protocol of several substituted *N*-(pyridin-2-yl)imidates from nitrostyrenes and 2-aminopyridines via the corresponding *N*-(pyridin-2-yl)iminonitriles as intermediates is reported. The reaction process involved the in situ formation of the corresponding α-iminontriles under heterogeneous Lewis acid catalysis in the presence of Al_2_O_3_. Subsequently, α-iminonitriles were selectively transformed into the desired *N*-(pyridin-2-yl)imidates under ambient conditions and in the presence of Cs_2_CO_3_ in alcoholic media. Under these conditions, 1,2- and 1,3-propanediols also led to the corresponding mono-substituted imidates at room temperature. The present synthetic protocol was also developed on one mmol scale, providing access to this important scaffold. A preliminary synthetic application of the present *N*-(pyridin-2-yl)imidates was carried out for their facile conversion into the *N*-heterocycles 2-(4-chlorophenyl)-4,5-dihydro-1*H*-imidazole and 2-(4-chlorophenyl)-1,4,5,6-tetrahydropyrimidine in the presence of the corresponding ethylenediamine and 1,3-diaminopropane.

## 1. Introduction

**Figure 1:**
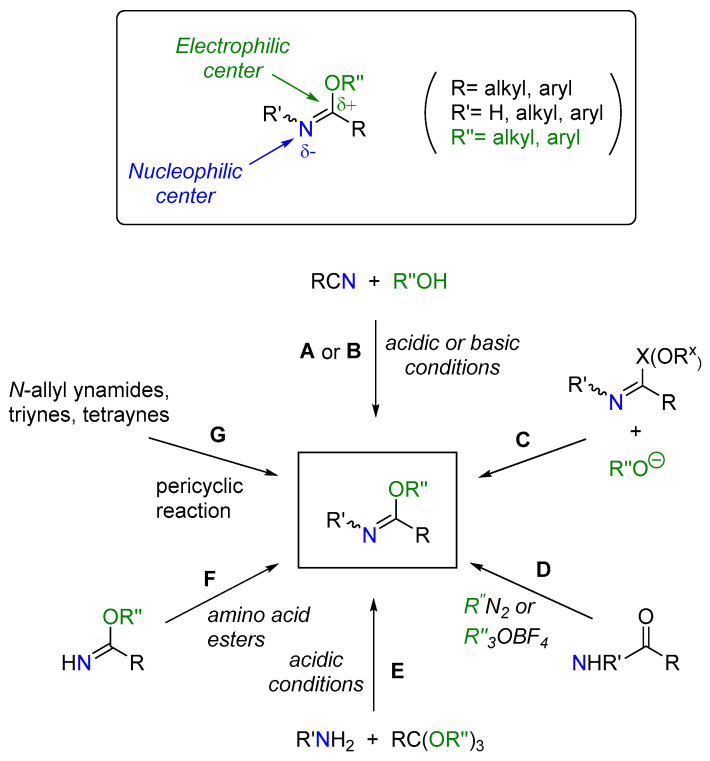
Synthetic methodologies (**A**–**G**) of imidates.

Imidates are considered to be one of the most important organic patterns due to their varied electronic nature [1,2]. In general, imidates serve as powerful molecules of electrophiles and nucleophiles in reactions (Figure 1) with several applications not only in structure functionalization, e.g., the synthesis of esters, amides, and amidines, but also in the synthesis of heterocyclic molecules [3]. For example, reported studies [4] on the successful transformation of imidates into a series of *N*-heterocycles, such as imidazolines, (benz)imidazoles, (benz)oxazoles, oxazolines, thiazolines, and azines, were summarized recently (Figure S1) [5]. Thus, it is interesting to note here that the last decade has witnessed the development of versatile synthetic methodologies towards their one-step synthesis using suitable imidate precursors. This synthetic strategy is still interesting and attractive, because it enables the formation of C-C and C-N bonds in one step with the nitrogen atom that comes from the imidates present in the final *N*-heterocyclic structure. Imidates have been applied in the synthesis of oxazoline-fused sugars from corresponding glycosyl-imidates [6,7] and a new C-C bond between sugars and aromatic compounds can be formed using glycosyl-trichloroacetimidates [8].

The most common synthetic process of the imidate moiety is the transformation of nitriles under acidic (a Pinner reaction) or basic conditions in alcoholic media (Figure 1A,B) [9,10,11,12,13,14]. For Pinner reactions, several methodologies have been reported, including the reactions of imidoyl halides with alkoxides and phenoxides or transesterification of imidates (Figure 1C) [2,15]; the conversion of amides to imidates in the presence of Meerwein reagents or diazo-compounds (Figure 1D) [2,16,17,18]; reactions of amino compounds with ortho-esters under acidic conditions (Figure 1E) [2]; the direct *N*-alkylation of imidates, using amino acid derivatives (Figure 1F) [2]; pericyclic reactions of unsaturated *N*-allyl ynamides, triynes, tetraynes, or ring openings of *N,N*- and *N,O*- heterocyclic compounds (Figure 1G) [19,20,21,22]; and syntheses from metal complexes and organometallic compounds [2].

**Scheme 1:**
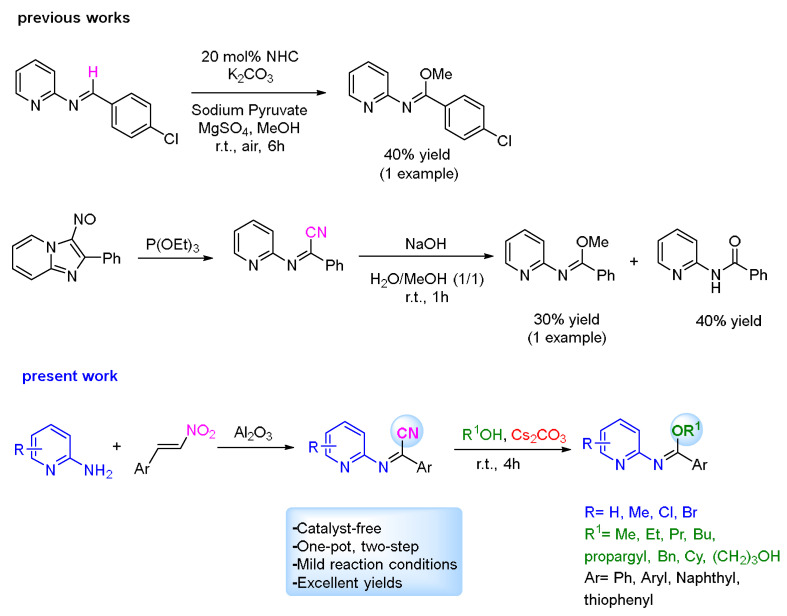
Synthetic approaches to *N*-(pyridin-2-yl)imidates [23,24].

To date, the emphasis in research has been on developing new, efficient, and “green” methods for the conversion of α-iminonitriles to valuable imidates. To the best of our knowledge, only specific examples with limited applicability on the synthesis of *N*-(pyridin-2-yl)imidates have been reported (Scheme 1). In the literature, methyl (*Z*)-*N*-(pyridin-2-yl)benzimidate was the only example observed in an equimolar ratio with the corresponding amide under basic conditions in aqueous media (NaOH, MeOH/H_2_O=1/1), initially starting from α-iminonitrile [23]. Recently, *N*-benzothiazolo-imines have been transformed into the corresponding methoxy imidates catalyzed by carbenes in the presence of sodium pyruvate, under oxidative conditions. Via this synthetic approach, only one example of *N*-(pyridin-2-yl)imidate has been presented [24]. Given the importance of this type of transformation and in terms of sustainability, the use of ambient and more eco-friendly heterogeneous conditions for the synthesis of substituted imidates continues to be a long-standing goal of chemical research. In light of our ongoing research directions for developing sustainable processes to construct *N*-heterocyclic organic molecules of high biological interest [25,26,27,28,29,30], herein we report the synthesis of a library of substituted *N*-(pyridin-2-yl)imidates from the corresponding *N*-(pyridin-2-yl)iminonitriles in the presence of Cs_2_CO_3_ and alcoholic media at ambient conditions (Scheme 1). Subsequently, the starting *N*-(pyridin-2-yl)iminonitriles were synthesized via a new heterogeneous catalytic process using Al_2_O_3_ in 1,2-dichloroethane (DCE) (Scheme 1). Thus, the present protocol is timely and of high interest, as it has a selective and sustainable synthetic character permitting the further diversification of the initial synthesized *N*-(pyridin-2-yl)iminonitriles libraries and giving us access to the synthetic, valuable substituted *N*-(pyridin-2-yl)imidates.

## 2. Results and Discussion

### 2.1. Evaluation of the Reaction Conditions

To optimize the reaction conditions, (*Z*)-4-methyl-*N*-(pyridin-2-yl)benzimidoyl cyanide (**1a**) was synthesized and selected as the model substrate. So far, the synthesis of *N*-(pyridin-2-yl)benzimidoyl cyanides from nitrostyrenes and 2-aminopyridine, using Ce(OTf)_3_ as catalyst in toluene and at 120 °C, has been reported [31]. Herein, we developed a facile and green procedure for the selective synthesis of the desired α-iminonitriles by the reaction of 2-aminopyridine and nitrostyrene in the presence of Al_2_O_3_ and DCE as solvents (see Materials and Methods part for details). According to the literature previous work [31], herein, alumina is initially catalyzing the Michael addition of 2-aminopyridine to nitrostyrene [32,33,34], leading to an intermediate enamine that further undergoes a proton transfer, dehydration, and [1.5]-H sigmatropic rearrangement to produce the final α-iminonitrile product. Thus, using the present procedure, the synthesized **1a** was determined and characterized by an HRMS analysis and IR spectroscopy, as described in Figure S2, with the characteristic absorbance of the nitrile group at ca. 2200 nm. All the spectroscopic data are in agreement with those reported in the literature [31]. After that, control experiments using **1a** (0.1 mmol) in MeOH (1 mL) in the presence of different bases were performed and the results are summarized in Table 1. Among the used bases, Cs_2_CO_3_ and DBU were found to promote the studied transformation within 4 h and with the quantitative transformation of **1a** to the desired methyl (*Z*)-4-methyl-*N*-(pyridin-2-yl)benzimidate **2aa** (Table 1, entries 7 and 10). Byproducts such as amide **3a**, ester **4a** and **5a**, and the starting amine **4** were observed in significant amounts in the case of ^t^BuOK or K_2_CO_3_ (Table 1, entries 3, 4, and 13). Similar results were observed when there was a lower amount of DBU (one equiv.) in the presence of molecular sieves or under an O_2_ atmosphere (Table 1, entries 11 and 12). NaOH was found to promote the developed transformation; however, the corresponding amide **3a** was detected in a 6% yield (Table 1, entry 14). It is worth noting that in the absence of a base, only a 18% yield was measured (Table 1, entry 15). Further increases in the temperature (50 °C) did not lead to significant increases in the desired product **2aa**’s yield (Table 1, entry 16); however, at 80 °C, **5a** and **6a** were formed as major products (Table 1, entry 17). All reactions were monitored by TLC and the products were characterized by ^1^H NMR spectroscopy.

To study further the present transformation, 0.1 mmol of **1a** were added into different alcoholic solvents, such as ethanol (EtOH), 1-propanol (PrOH), and 1-butanol (BuOH), and the reactions were performed in the presence of different equiv. of the Cs_2_CO_3_ and DBU (Tables S1–S3). In all cases, the corresponding imidates **2ab**, **2ac**, **2ad,** and **2ae** were formed in high yields. The optimum amount of Cs_2_CO_3_ was found to be between one and two equiv. based on the amount of **1a**. Under a lower amount of the base, no reaction completion was observed, and significant amounts of the amide **3a** were measured (Tables S1–S3). In the case of BuOH, a higher temperature was required (50 °C) for reaction completion and for better solubility of the base Cs_2_CO_3_ (see Table S3). The results under optimum conditions, two equiv. of Cs_2_CO_3_ and 24 h, from the experiments in alcoholic solvents are presented in Figure S3. It can be concluded that bulkier alcohols could lead to a decrease in the yield of the desired imidates, because the nucleophilic attack of the alcohol to the electrophilic carbon of the α-iminonitrile is more difficult due to steric effects.

To increase the synthetic value of the present protocol, we studied the selective transformation of α-iminonitrile **1a** to imidate **2aa** in the presence of two equiv. of Cs_2_CO_3_, using different solvent mixtures with MeOH in ratios of 1/1 and 1/4. As shown in Table 2, in all cases, the quantitative consumption of the initial **1a** was observed and the desired **2aa** was formed in a high yield (95–99%), except in the presence of water, in which a significant amount of amide was observed (Table 2, Entry 8). These encouraging results support the plausible general application of the present facile protocol in synthetic chemistry.

### 2.2. Application of the Synthetic Transformation of N-(Pyridin-2-yl)benzimidoyl Cyanides to the N-(Pyridine-2-yl)imidates

**Scheme 2:**
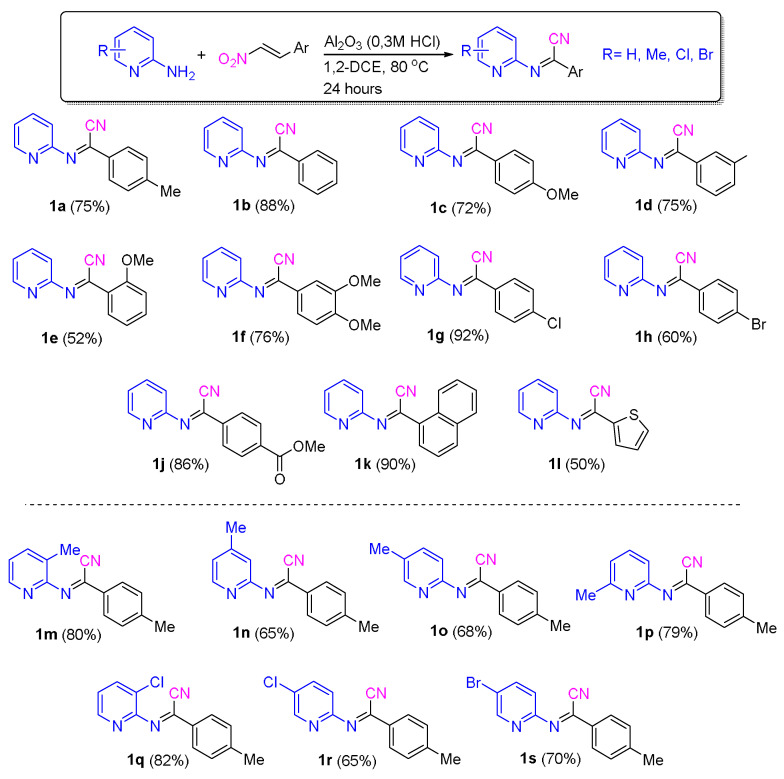
Multifunctional *N*-pyridinyl substituted iminonitriles synthesized by the Al_2_O_3_-mediated reaction of nitrostyrenes with aminopyridines.

To explore the substrate broadness of the described synthetic protocol, initially, a series of multifunctional *N*-(pyridin-2-yl)benzimidoyl cyanides were synthesized according to the above reported reaction of nitrostyrenes with 2-aminopyridine in the presence of Al_2_O_3_ and DCE as solvents (Scheme 2). The corresponding α-iminonitriles **1a**–**1h** and **1j**–**1s** were isolated in moderate to high yields (50–88%) after the simple filtration of the catalyst and chromatographic purification using silica gel and Hexane/EtOAc as the solvent mixture eluent (for details, see the Section 3 and the Supplementary Materials). The α-iminonitriles were characterized by ^1^H NMR and compared with those of reported examples in the literature [31].

**Scheme 3:**
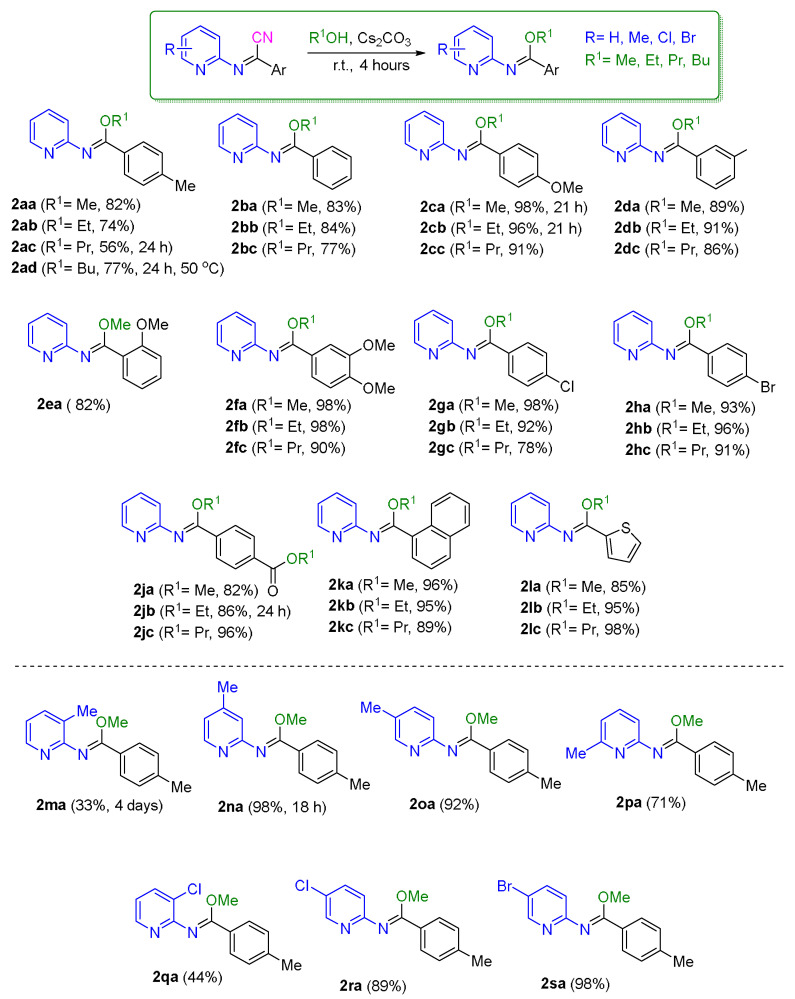
Synthetic protocol of *N*-(pyridine-2-yl)imidates from *N*-(pyridin-2-yl)benzimidoyl cyanides.

Having in our hands the above optimal conditions, the selective transformation of the synthesized α-iminonitriles to the corresponding imidates (**2aa**–**2hc** and **2ja**–**2sa**) was studied at ambient conditions. The observed products were summarized in Scheme 3 and the values in parentheses correspond to the isolated yields after purification by column chromatography on a silica gel using a gradient mixture of EtOAc−hexane (see Supplementary Materials). To our delight, in most cases, the desired imidates (**2aa**–**2hc** and **2ja**–**2sa**) were formed in good to high yields (56–98%). In particular, when MeOH was used, clean and quantitative transformations of the α-iminonitriles to the corresponding imidates (**2aa**–**2ha** and **2ja**–**2la**) were observed. Similarly high yields were also observed in the case of ethanolic (**2ab**–**2hb** and **2jb**–**2lb**) and propanolic (**2ac**–**2hc** and **2jc**–**2lc**) solutions (Scheme 2). Only when butanol was used as the reaction solvent was a higher temperature required (50 °C) for reaction completion, and the corresponding imidate (**2ad**) was isolated with a 77% yield within 24 h (Scheme 2). Importantly, in the reaction of **1j** (substrate bearing a -COOMe moiety in the *para*-position of the phenyl ring), an in situ transesterification was observed in the presence of EtOH and PrOH. Thus, the isolated imidates **2jb** and **2jc** contained in their structures the -COOEt and -COOPr moieties, respectively (Scheme 3). Subsequently, a series of methyl-, chloro-, and bromo-substituted 2-aminopyridino-iminonitriles (**1m**–**1s**, Scheme 2) were successfully transformed into the corresponding imidates (**2ma**–**2sa**) using methanol and isolated in moderate to high yields, from 33% to 98% (Scheme 3). It is worth noting that the *o*-Me-substituted pyridine derivative led to the corresponding imidate (**2ma**) in a low yield (33%), even after a prolonged reaction time (4 days), probably for steric reasons.

The synthesized imidates were characterized by NMR and HRMS spectroscopies. During HRMS operation, two fragments, [M−31]^+^ and [M+15]^+^, were observed with the use of a higher fragmentor voltage (200 V). The [M−31]^+^ fragment presumably resulted from the elimination of the methoxy group (−31) to form the corresponding stable cationic intermediate, and then the addition of a formic acid molecule (+46), which was present in the eluent solvent, resulted in the [M+15]^+^ fragment. For example, during the HRMS analysis of imidate **2ca**, except for the main fragment [M+H]^+^ = 243 observed at 50 V, two new fragments appeared at 200 V [M−31]^+^ = 211 and [M+15]^+^ = 257, which corresponded to the intermediates derived by a methoxy moiety elimination and further trapping by the formic acid (Figure S4). These observations also support the structure of the present desired imidates.

**Scheme 4:**
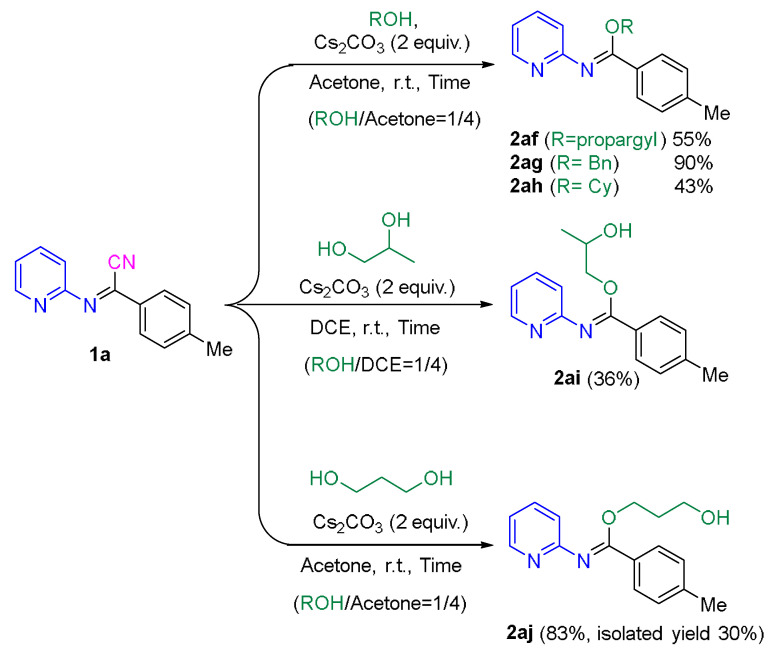
Application using alcohols with higher molecular weight and diols, and synthesis of the corresponding imidates.

To extend the substrate broadness, alcohols with a high molecular weight, such as benzyl alcohol (BnOH) and cyclohexyl alcohol (CyOH), were used in the presence of acetone as co-solvents in a ratio of 1/4 (Scheme 4). The corresponding imidates **2ag** and **2ah** were formed in good yields (90% and 43%). In addition, propargyl alcohol was found to be active under the present conditions and led to the desired imidate **2af** in a 55% yield (Scheme 4). Biobased products 1,2-propanediol and 1,3-propanediol were also tested under the present proposed conditions, with 0.1 mmol of **1a** and in the presence of different co-solvents (DCE, acetone, and DMSO), as shown in Tables S4 and S5. To our surprise, the corresponding imidates, **2ai** and **2aj**, were formed as the major products (36% and 83%, Scheme 4), accompanied with significant amount of the amide **3a** and the esters **5i** and **5j** (Tables S4 and S5). It is worth mentioning that the imidate **2aj** was purified by column chromatography in a 30% yield; however, the yields of **2af**, **2ag, 2ah,** and **2ai** were calculated by the ^1^H NMR of the crude reaction mixture, with the use of 1,3-dimethoxybenzene as the internal standard.

**Scheme 5:**
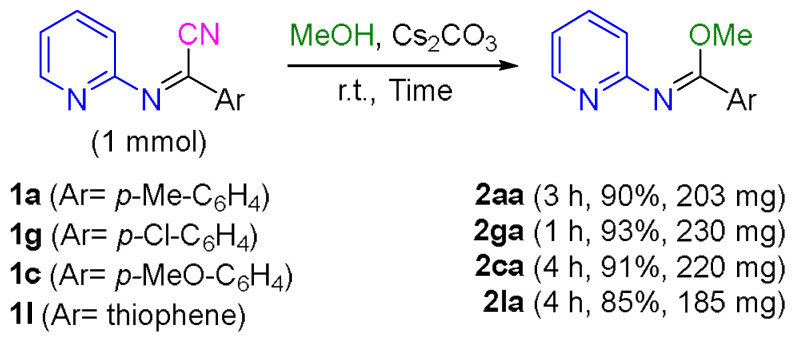
One-mmol-scale process for the selective transformation of α-iminonitriles into imidates in MeOH.

These results indicate the broad generality of the present protocol toward the synthesis of substituted *N*-(pyridin-2-yl)imidates in the presence of alcoholic media. Based on these encouraging results, the synthesis of the imidates **2aa**, **2ga**, **2ca,** and **2la** was further tested at the lab scale of one mmol. Thus, the corresponding amount of each iminonitrile, **1a**, **1g**, **1c,** and **1l,** was diluted in 2 mL of MeOH in the presence of two equiv. of Cs_2_CO_3_ at ambient temperature and stirred for an appropriate time. After reaction completion (ca. 1–4 h, based on TLC), the reaction mixture was filtered with the use of a short pad of silica gel and washed with ca. 10 mL of EtOAc. The corresponding imidates were isolated after chromatographic purification (see Materials and Methods) in 90%, 93%, 91%, and 85% yields, respectively (Scheme 5).

**Scheme 6:**
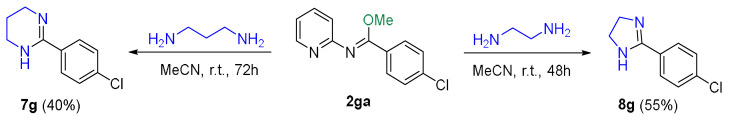
Preliminary application of imidate **2ga** transformation into *N*,*N*-six- and *N*,*N*-five-membered ring heterocycles **7g** and **8g**.

Furthermore, an attempt was made to synthesize the skeleton of *N*,*N*-six- and *N*,*N*-five-membered ring heterocycles, such as 2-substituted tetrahydropyrimidines and dihydroimidazoles. These heterocycles constitute an important core of many natural products and exhibit a variety of biological effects, including antimicrobial and anti-inflammatory effects. They can even be used as therapeutic agents for the treatment of Alzheimer’s disease [35,36,37,38]. Herein, we succeed in applying the present simple and mild protocol to the synthesis of 2-(4-chlorophenyl)-1,4,5,6-tetrahydropyrimidine **7g** and 2-(4-chlorophenyl)-4,5-dihydro-1*H*-imidazole **8g** via a reaction between **2ga** and 1,3-diaminopropane and ethylenediamine, respectively. The corresponding heterocycles were isolated in moderate yields, 40% and 55%, as shown in Scheme 6. These are the preliminarily results for the synthesis of *N*,*N*-heterocycles from imidates; however, further studies evaluating the reaction conditions are in progress.

## 3. Materials and Methods

### 3.1. General and Aparatus

All the reagents and solvents were purchased from Sigma-Aldrich, TCI Chemicals, AK Scientific, Fluorochem, and were used without further purification. Thin-layer chromatography was performed on Millipore precoated silica gel plates (0.20 mm thick, particle size of 25 μm). Nuclear magnetic resonance spectra were recorded on Bruker Avance 500 or 600 spectrometers and on Agilent 500 (^1^H NMR (500 MHz), ^13^C{H} NMR (126 MHz)). Chemical shifts for ^1^H NMR were reported as *δ* values and coupling constants were measured in hertz (Hz). The following abbreviations were used for spin multiplicity: s = singlet, br s = broad singlet, d = doublet, t = triplet, q = quartet, quin = quintet, dd = double of doublets, ddd = double doublet of doublets, and m = multiplet. Chemical shifts for ^13^C{H} NMR were reported in ppm relative to the solvent peak. Mass spectra were measured on a Waters Investigator Supercritical Fluid Chromatograph with a 3100 MS Detector (ESI) using a solvent system of methanol and CO_2_ on a Viridis silica gel column (4.6 × 250 mm, 5 μm particle size) or Viridis 2-ethyl pyridine column (4.6 × 250 mm, 5 μm particle size). Mass spectra (HRMS) were carried out on an Agilent Q-TOF Mass Spectrometer, G6540B model with a Dual AJS ESI-MS source. All of the compounds (dissolved in LC-MS-grade acetonitrile containing 0.05% formic acid) were introduced into the ESI source of the mass spectrometer with a single injection of 15 μL of the sample and a flow rate of 300 μL/min of 100% methanol as the solvent in the binary pump. The experiments were run using a Dual AJS ESI source, operating in the positive ionization mode. Source operating conditions were as follows: gas temperature of 330 °C, gas flow of 8 L/min, sheath gas temperature of 250 °C, sheath gas flow of 10 L/min, and fragmentor voltage of 50–200 V. Data-dependent MS/MS analysis was performed in parallel with MS analysis in the centroid mode, using different collision energies (10, 20, 30, 40 V). All accurate mass measurements of the [M+H]^+^ ions were performed by scanning from 100 to 800 m/z. The Q-TOF was calibrated 1 h prior to the infusion experiments by a calibration mixture. Data were acquired in an external calibration mode.

### 3.2. Synthesis of Aromatic β-Nitrostyrenes

Aromatic *β*-nitrostyrenes were synthesized according to the literature procedure [39]. In a solution of ammonium acetate (12.5 mmol) in acetic acid (10 mL), aromatic aldehyde (5 mmol) and nitromethane (15.5 mmol) in one portion were added. The mixture was heated at reflux for 24 h. The reaction mixture was cooled at room temperature and then poured into ice water to precipitate the corresponding nitrostyrene. After extraction with organic solvent (EtOAc), the organic layer was evaporated under vacuum, and the residue was purified by column chromatography using silica gel to give final the corresponding products in good yields. In alternative, the precipitated solid was collected in pure form by filtration under vacuum in a Buchner funnel and washed with distilled water.

### 3.3. Synthesis of N-(Pyridin-2-yl)iminonitriles from Nitrostyrenes and 2-Aminopyridine

In a sealed tube of 1 mmol of nitrostyrene and 1.2 mmol of 2-aminopyridine, 10 mL of DCE as a solvent and 300 mg Al_2_O_3_ (0.3 M HCl) as a catalyst were added. The reaction mixture was stirred at 80 °C for 24 h. The reaction was monitored by thin-layer chromatography (TLC) and the slurry was filtered under pressure through a short pad of silica to withhold the catalyst with the aid of dichloromethane (DCM) and ethyl acetate (EtOAc). The filtrate was evaporated under vacuum and purified by column chromatography on a silica gel using a gradient mixture of EtOAc−hexane to afford the corresponding products in good yields.

The Al_2_O_3_ (0.3 M HCl) was prepared by the addition of 1 g of Al_2_O_3_ in 10 mL of a 0.3 M HCl aqueous medium and stirred for 2 h at room temperature. After filtration, the solid catalyst was dried in an oven at 100 °C for 24 h and was used for the present transformation.

### 3.4. Synthesis of N-(Pyridin-2-yl)imidates from N-(Pyridin-2-yl)iminonitriles

In a 4 mL vial, 2 equivalents of Cs_2_CO_3_ (0.4 mmol, 130.4 mg) and 1 mL of the corresponding alcohol were added. After stirring for a few minutes, 0.2 mmol of α-iminonitrile was added until the amount of Cs_2_CO_3_ was fully dissolved. The reaction mixture was stirred at room temperature for 4 h. The reaction was monitored by thin-layer chromatography (TLC) and after completion, the reaction mixture was filtered under pressure through a short pad of silica and celite to withhold the salt. The vial and the silica layer were washed with ca. 5 mL of dichloromethane (DCM) and ca. 5 mL of ethyl acetate. The organic solvents were evaporated under vacuum and the product was determined by ^1^H NMR spectroscopy. In most cases, the imidate was formed in pure form; however, when a mixture of compounds was obtained, the desired imidate was purified by column chromatography on a silica gel using a gradient mixture of EtOAc−hexane and obtained in good to high isolated yields.

### 3.5. 1 mmol Scale Synthesis of N-(Pyridin-2-yl)imidates ***2aa***, ***2ca***, ***2ga***, and ***2la***

In a 15 mL vial, 2 mmol of Cs_2_CO_3_ and 2 mL of the methanol were added. After stirring for a few minutes, 1 mmol of α-iminonitrile was added until the amount of Cs_2_CO_3_ was fully dissolved. The reaction mixture was stirred at room temperature for 1–4 h, based on iminonitrile’s conversion, which was monitored by thin-layer chromatography (TLC). After completion, the reaction mixture was filtered under pressure through a short pad of silica and celite to withhold the salt. The vial and the silica layer were washed with ca. 5 mL dichloromethane (DCM) and ca. 5 mL ethyl acetate. The organic solvents were evaporated under vacuum and the product was determined by ^1^H NMR spectroscopy.

### 3.6. Synthesis of N,N-Heterocyclic Compounds from N-(Pyridin-2-yl)imidates

To a 4 mL vial containing 0.2 mmol of *N*-(pyridin-2-yl)imidate, 0.5 mL of acetonitrile and 0.4 mmol of diamine were added. The reaction mixture was stirred at room temperature for a few hours or days, depending on the progress of the reaction, monitored by thin-layer chromatography (TLC). After completion of the reaction, the solution was evaporated and then left under vacuum for 2–3 h. The mixture was then rinsed to remove the 2-aminopyridine resulting from the starting material, with simultaneous crystallization of the product using hexane.

## 4. Conclusions

In conclusion, we showed that a series of multi-functional *N*-(pyridin-2-yl)iminonitriles were selectively transformed into the desired *N*-(pyridin-2-yl)imidates under a simple and mild synthetic protocol. For the present study, a series of substituted *N*-(pyridin-2-yl)imidates were synthesized with the Al_2_O_3_-mediated reaction of the corresponding nitrostyrenes with 2-aminopyridines. The α-iminonitriles were efficiently transformed into the desired *N*-(pyridin-2-yl)imidates in the presence of Cs_2_CO_3_ in alcoholic media and under ambient conditions. In addition to the commonly studied methanol, ethanol, propanol, and butanol, 1,2- and 1,3-propanediols were also studied under the present conditions, leading to the corresponding imidates. The present synthetic protocol can easily be applied to at a one mmol scale, resulting an important synthetic access to this interesting scaffold. A preliminary synthetic application to the *N,N*-heterocycles 2-(4-chlorophenyl)-4,5-dihydro-1*H*-imidazole and 2-(4-chlorophenyl)-1,4,5,6-tetrahydropyrimidine was also presented, using the corresponding 1,2- and 1,3-diamines.

## Figures and Tables

**Table 1:** Conditions and different bases evaluation for the transformation of **1a** to **2aa** in the presence of MeOH.

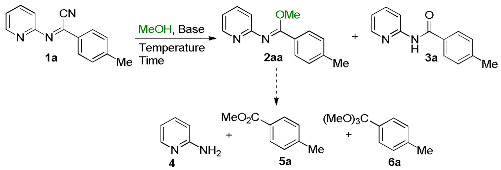								
			Products (%) ^[b]^					
Entry	Base (eq.) ^[a]^	Time (h)	1a	2aa	3a	4	5a	6a
1	Et_3_N (1.5)	4	50	50	-	-	-	-
2	Imidazole (1.5)	4	87	13	-	-	-	-
3	^t^BuOK (1.5)	4	-	70	10	10	3	7
4	^t^BuOK (2)	4	-	90	4	3	2	1
5	AcONa (1.5)	4	62	38	-	-	-	-
6	AcONa (2)	4	58	42	-	-	-	-
7	DBU (1.5)	4	-	100	-	-	-	-
8	DBU (2)	4	-	100	-	-	-	-
9	NaHCO_3_ (1.5)	4	36	56	8	-	-	-
10	Cs_2_CO_3_ (1.5)	4	-	100	-	-	-	-
11 ^[c]^	Cs_2_CO_3_ (1)	4	-	92	2	3	2	1
12 ^[d]^	Cs_2_CO_3_ (1)	4	**-**	91	3	3	1	2
13	K_2_CO_3_ (1.5)	4	-	91	3	3	1	2
14	NaOH (1)	4	-	94	6	-	-	-
15	-	4	82	18	-	-	-	-
16 ^[e]^	-	24	51	49	-	-	-	-
17 ^[f]^	-	24	-	10	-	45	10	35

^[a]^ Conditions: **1a** (0,1 mmol), MeOH (1 mL), room temperature. ^[b]^ Yields measured by ^1^H NMR based on the addition of specific amount of 1,3-dimethoxybenzene as internal standard. ^[c]^ Molecular Sieves. ^[d]^ O_2_ atmosphere. ^[e]^ Temperature = 50 °C. ^[f]^ Temperature = 80 °C.

**Table 2:** Reaction evaluation using different co-solvents for the transformation of **1a** to **2aa** in the presence of MeOH.

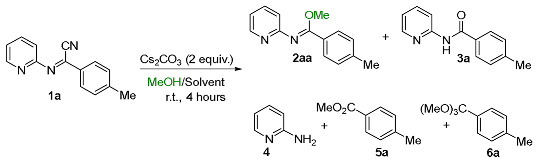							
			Products (%) ^[b]^				
Entry	Solvent ^[a]^	MeOH/Solvent	2aa	3a	4	5a	6a
1	THF	1/1	98	2	-	-	-
2	THF	1/4	98	2	-	-	-
3	DCE	1/4	95	5	-	-	-
4	Acetone	1/4	97	3	-	-	-
5	CH_3_CN	1/4	99	1	-	-	-
6	DMSO	1/1	99	1	-	-	-
7	EtOAc	1/1	95	5	-	-	-
8 ^[c,d]^	H_2_O	1/1	44	43	-	-	-

^[a]^ Conditions: **1a** (0,1 mmol), MeOH/Solvent (1/1 or 1/4) in total volume of 1 mL, room temperature. ^[b]^ Relative yields and conversion measured by ^1^H NMR of the crude reaction mixture based on the addition of specific amount of 1,3-dimethoxybenzene as internal standard. ^[c]^ Reaction time 24 h. ^[d]^ A total of 13% of starting material **1a** was observed.

## Data Availability

Not applicable.

## Supplementary Materials

The following supporting information can be downloaded at https://www.mdpi.com/article/10.3390/molecules28083321/s1: Figures S1–S4; Tables S1–S5; and the NMR data and spectra of compounds.

## Sample Availability

Not available.
