# Supplementary material for: Simple Synthetic Approach to N-(Pyridin-2-yl)imidates from Nitrostyrenes and 2-Aminopyridines via the N-(Pyridin-2-yl)iminonitriles as Intermediates
Source: Molecules. 2023 Apr 9;28(8):3321. doi: 10.3390/molecules28083321 (PMC10147006; doi:10.3390/molecules28083321)
Supplement: Supplementary file 1 [file molecules-28-03321-s001.zip › molecules-2310298-supplementary.pdf]

Supplementary Materials

Simple Synthetic Approach to *N*-(Pyridin-2-yl)imidates from Nitrostyrenes and 2-Aminopyridines via the *N*-(Pyridin-2-yl)iminonitriles as Intermediates

Andriani G. Chaidali and Ioannis N. Lykakis*

Department of Chemistry, Aristotle University of Thessaloniki, University Campus 54124, Thessaloniki, Greece.
* Correspondence: Email: lykakis@chem.auth.gr

**Table of contents**	Page
**Figure S1.** Synthetic approaches to N-heterocycles starting from imidates	2
**Figure S2.** IR and HRMS spectra of the compound **1a**	3
**Figure S3**. Study of alcoholic solvent in the transformation of **1a** in the presence of Cs_2CO_3	4
**Figure S4.** HRMS spectra of **2ca** at 50 V and 200 V and the corresponding observed fragments $[M+H]^+$, $[M-31]^+$ and $[M+15]^+$	5
**Table S1.** Screening of base equivalents, with EtOH as solvent	6
**Table S2.** Screening of base equivalents, with PrOH as solvent	7
**Table S3.** Screening of base equivalents, with BuOH as solvent	8
**Table S4.** Screening of reaction, in the presence of 1,2-propanediol	9
**Table S5.** Screening of reaction, in the presence of 1,3-propanediol	10
**^1H and $^{13}C\{H\}$ NMR data for α-iminonitriles 1a-1h, 1j-1s**	11-14
**^1H and $^{13}C\{H\}$ NMR data for imidates 2aa-2ad, 2aj, 2ba-2hc, 2ja-2sa**	15-23
**^1H and $^{13}C\{H\}$ NMR data for *N*,*N*-heterocycles 7g, 8g**	24
**References**	25
**Copies of ^1H and $^{13}C\{H\}$ NMR spectra of α-iminotriles 1a-1h, 1j-1s**	26-43
**Copies of ^1H and $^{13}C\{H\}$ NMR spectra of imidates imidates 2aa-2ad, 2aj, 2ba-2hc, 2ja-2sa**	44-83

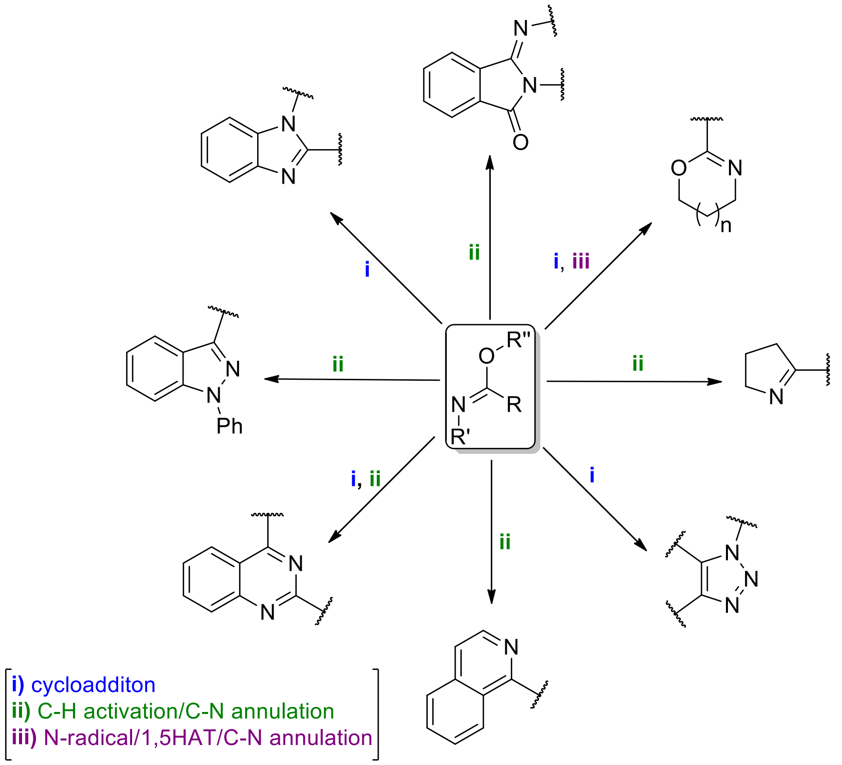

Figure S1. Synthetic approaches to N-heterocycles starting from imidates

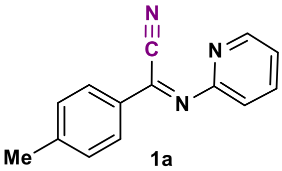

1a

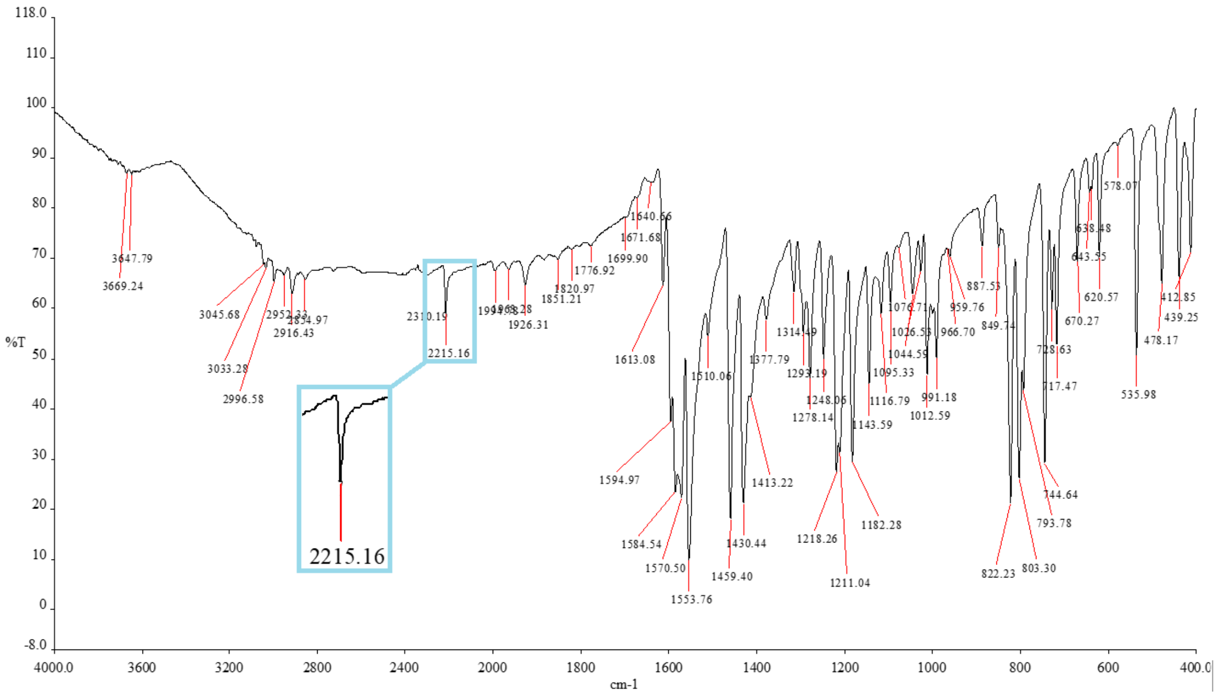

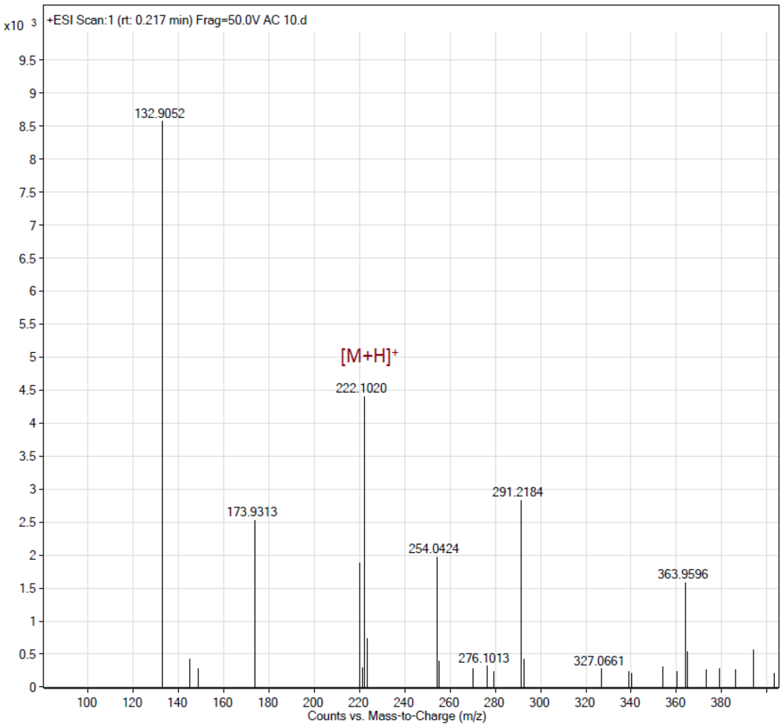

Figure S2. IR and HRMS spectra of the compound **1a**

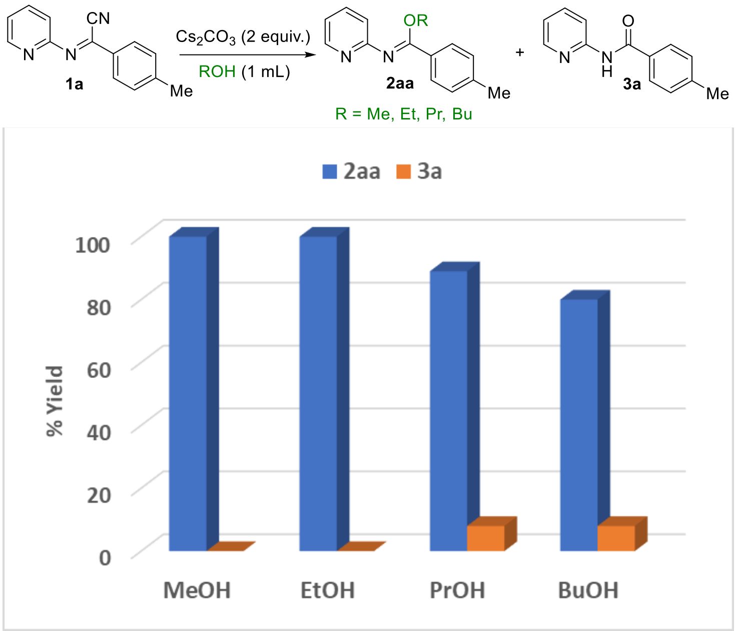

Figure S3. Study of alcoholic solvent in the transformation of **1a** in the presence of Cs_2CO_3.

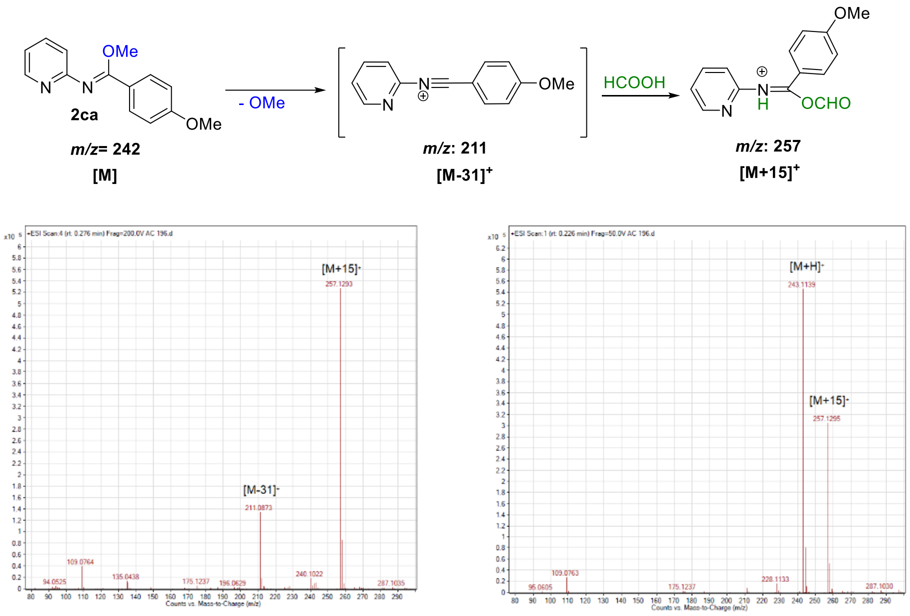

Figure S4. HRMS spectra of **2ca** at 50 V (left) and 200 V (right) and the corresponding observed fragments $[M+H]^+$, $[M-31]^+$ and $[M+15]^+$

Table S1. Screening of base equivalents, with EtOH as solvent

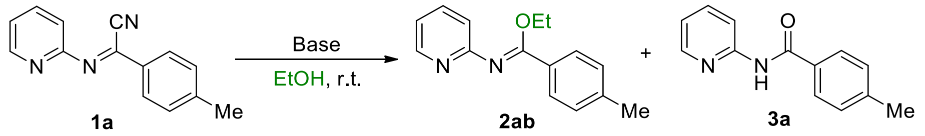

Entry	Base	Base equiv.	Time (h)	Conversion (%) [b]	2ab (%) [b]	3a (%) [b]
**1**	Cs_2CO_3	2	4	100	100	0
**2**	DBU	2	24	100	100	0
**3**	Cs_2CO_3	1	24	100	91	9
**4**	DBU	1	24	100	84	16
**5**	Cs_2CO_3	0.5	24	100	92	8
**6**	DBU	0.5	48	100	73	7
**7**	Cs_2CO_3	0.2	48	95	77	6
**8**	DBU	0.2	48	96	75	5
**9**	-	-	24	17	17	0
**10**	-	-	48	28	28	0

[a] Conditions: (*Z*)-4-methyl-N-(pyridin-2-yl)benzimidoyl cyanide **1a** (0,1 mmol), EtOH (1 ml). [b] Relative yields and conversion measured by ^{1}H NMR of the crude reaction mixture.

Table S2. Screening of base equivalents, with PrOH as solvent

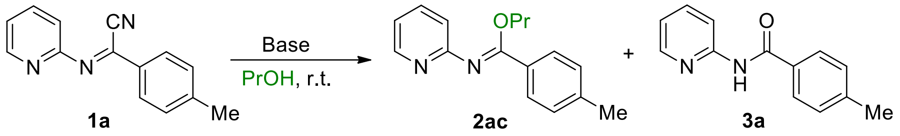

Entry	Base	Base equiv.	Time (h)	Conversion (%) [b]	2ac (%) [b]	3a (%) [b]
**1**	Cs_2CO_3	2	24	100	89	6
**2**	DBU	2	24	100	80	15
**3**	Cs_2CO_3	1	24	100	84	11
**4**	DBU	1	24	100	79	16
**5**	Cs_2CO_3	0.5	24	100	73	22
**6**	DBU	0.5	24	100	71	24
**7**	-	-	24	14	14	0
**8**	-	-	48	27	27	0

[a] Conditions: (*Z*)-4-methyl-N-(pyridin-2-yl)benzimidoyl cyanide **1a** (0,1 mmol), PrOH (1 ml). [b] Relative yields and conversion measured by ^{1}H NMR of the crude reaction mixture, 2-aminopyridine and propyl 4-methylbenzoate of about 5% is observed in all reactions.

Table S3. Screening of base equivalents, with BuOH as solvent

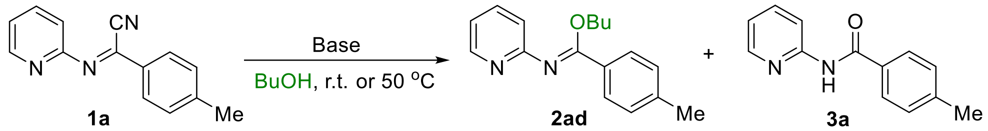

Entry	Base	Base equiv.	Time (h)	Conversion (%) [c]	2ad (%) [c]	3a (%) [c]
**1**	Cs_2CO_3	2	24	36	16	20
**2**	DBU	2	24	70	37	33
**3**	Cs_2CO_3	1	24	50	15	15
**4** [b]	Cs_2CO_3	1	24	88	80	8
**5**	DBU	1	24	65	20	20
**6**	DBU	0.5	24	70	25	20
**7** [b]	-	-	48	65	35	30

[a] Conditions: (*Z*)-4-methyl-N-(pyridin-2-yl)benzimidoyl cyanide **1a** (0,1 mmol), BuOH (1 ml). [b] The reaction was carried out at 50 °C. [c] Relative yields and conversion measured by ^{1}H NMR of the crude reaction mixture.

Table S4. Screening of reaction, in the presence of 1,2-propanediol

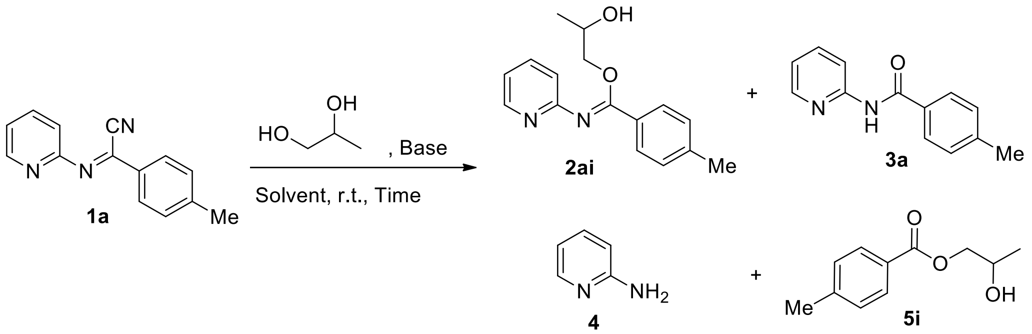

Entry	Base (equiv)	Solvent	Time (h)	Conversion (%) [b]	2ai (%) [b]	Byproducts (%) [b]
**1**	Cs_2CO_3 (1)	DCE	24	90	20	**3a** (19), **4** (33), **5i** (28)
**2**	Cs_2CO_3 (2)	DCE	24	100	36	**3a** (17), **4** (24), **5i** (23)
**3**	Cs_2CO_3 (2)	Acetone	4	100	0	**Unidentified products**
**4**	-	DCE	24	0	0	0

[a] Conditions: **1a** (0,1 mmol), 1,2-propanediol (0.2 ml), Solvent (0.8 mL). [b] Relative yields and conversion measured by ^{1}H NMR of the crude reaction mixture.

Table S5. Screening of reaction, in the presence of 1,3-propanediol

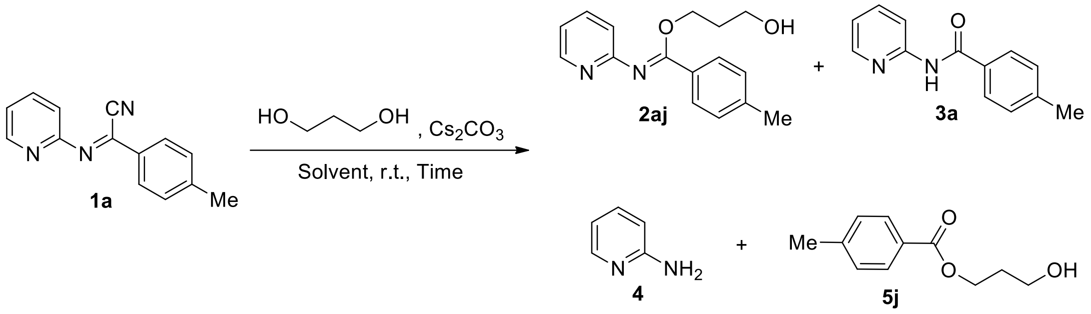

Entry	Solvent	Cs_2CO_3 equiv.	Time (h)	Conversion (%) [b]	2aj (%) [b]	Byproducts (%) [b]
**1**	DCE	1	48	53	36	**3a** (17)
**2**	DCE	2	48	69	42	**3a** (27)
**3**	Acetone	2	4	100	83	**3a** (17)
**4**	DMSO	2	4	100	72	**4** (12), **5j** (12)

[a] Conditions: **1a** (0,1 mmol), 1,3-propanediol (0,2 ml), Solvent (0,8 ml). [b] Relative yields and conversion measured by ^{1}H NMR of the crude reaction mixture.

^{1}H and ^{13}C{H} NMR data for *α*-iminonitriles 1a-1h, 1j-1s

*(Z)-4-methyl-N-(pyridin-2-yl)benzimidoyl cyanide (**1a**):*[S1] Yellow solid, 165 mg, yield 75%; Purified by flash column chromatography (SiO_2, elution system Hexane/EtOAc 10/1); mp: (88–90 °C); ^{1}H NMR (500 MHz, $CDCl_3$) δ 8.60 (dd, J_1 = 5.0 Hz, J_2 = 1.0 Hz, 1H), 8.12 (d, J = 8.0 Hz, 2H), 7.86 (td, J_1 = 7.5 Hz, J_2 = 2.0 Hz, 1H), 7.34 (d, J = 8.0 Hz, 2H), 7.32 – 7.22 (m, 2H), 2.46 (s, 3H); ^{13}C{H} NMR (125 MHz, $CDCl_3$): δ 159.5, 149.0, 144.6, 141.3, 138.5, 131.2, 129.9, 128.9, 122.9, 118.3, 111.7, 21.9; HRMS (ESI) *m/z*: calcd for $C_{14}H_{12}N_3$ $[M+H]^+$ 222.1026, found: 222.1020.	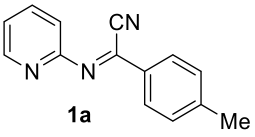 **1a**
*(Z)-N-(pyridin-2-yl)benzimidoyl cyanide (**1b**):*[S1] Brown solid, 182 mg, yield 88%; Purified by flash column chromatography (SiO_2, elution system Hexane/EtOAc 10/1); mp: (61-63 °C); ^{1}H NMR (500 MHz, $CDCl_3$) δ 8.61 (d, J = 4.0 Hz, 1H), 8.23 (d, J = 8.0 Hz, 2H), 7.85 (t, J = 7.5 Hz, 1H), 7.62 (t, J = 7.5 Hz, 1H), 7.54 (t, J = 8.0 Hz, 2H), 7.30 – 7.26 (m, 2H); ^{13}C{H} NMR (125 MHz, $CDCl_3$): δ 159.3, 149.0, 141.4, 138.5, 133.8, 133.5, 129.2, 128.9, 123.1, 118.5, 111.6; HRMS (ESI) *m/z*: calcd for $C_{13}H_9N_3$ $[M+H]^+$ 208.0869, found 208.0870.	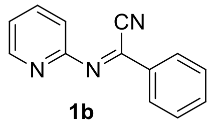 **1b**
*(Z)-4-methoxy-N-(pyridin-2-yl)benzimidoyl cyanide (**1c**):*[S2] Yellow solid, 171 mg, yield 72%; Purified by flash column chromatography (SiO_2, elution system Hexane/EtOAc 10/1); mp: (76-78 °C); ^{1}H NMR (500 MHz, $CDCl_3$) δ 8.58 (d, J = 4.0 Hz, 1H), 8.18 (d, J = 8.5 Hz, 2H), 7.82 (t, J = 7.5 Hz, 1H), 7.25 – 7.21 (m, 2H), 7.02 (d, J = 8.5 Hz, 2H), 3.91 (s, 3H); ^{13}C{H} NMR (125 MHz, $CDCl_3$) δ 164.1, 159.6, 148.9, 140.6, 138.5, 131.0, 126.7, 122.6, 118.2, 114.6, 111.7, 55.8; HRMS (ESI) *m/z*: calcd for $C_{14}H_{12}N_3O$ $[M+H]^+$ 238.0975; found 238.0979.	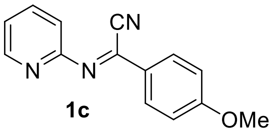 **1c**
*(Z)-3-methoxy-N-(pyridin-2-yl)benzimidoyl cyanide (**1d**):*[S1] Yellow oil, 178 mg, yield 75%; Purified by flash column chromatography (SiO_2, elution system Hexane/EtOAc 10/1); ^{1}H NMR (500 MHz, $CDCl_3$) δ 8.59 (d, J = 4.0 Hz, 1H), 7.83 (dd, J_1 = 8.0 Hz, J_2 = 1.5 Hz, 1H), 7.79 (d, J = 8.0 Hz, 1H), 7.74 (s, 1H), 7.43 (t, J = 8.0 Hz, 1H), 7.30 – 7.26 (m, 2H), 7.14 (dd, J_1 = 8.5Hz, J_2 = 2.5 Hz, 1H), 3.87 (s, 3H); ^{13}C{H} NMR (125 MHz, $CDCl_3$): δ 160.2, 159.3, 149.0, 141.3, 138.5, 135.1, 130.2, 123.1, 122.2, 120.5, 118.3, 112.2, 111.6, 55.7; HRMS (ESI) *m/z*: calcd for $C_{14}H_{12}N_3O$ $[M+H]^+$ 238.0975; found 238.0979.	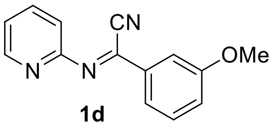 **1d**
*(Z)-2-methoxy-N-(pyridin-2-yl)benzimidoyl cyanide (**1e**):* Brown oil, 123 mg, yield 52%; Purified by flash column chromatography (SiO_2, elution system Hexane/EtOAc 8/1 to 5/1); ^{1}H NMR (500 MHz, $CDCl_3$) δ 8.59 (d, J = 4.5 Hz, 1H), 7.91 (d, J = 8.0 Hz, 1H), 7.81 (t, J = 7.5 Hz, 1H), 7.54 (t, J = 8.0 Hz, 1H), 7.25 – 7.22 (m, 1H), 7.17 (d, J = 8.0 Hz, 1H), 7.07 (t, J = 7.5 Hz, 1H), 7.03 (d, J = 8.5 Hz, 1H), 3.97 (s, 3H); ^{13}C{H} NMR (125 MHz, $CDCl_3$): δ 160.1, 159.1, 149.1, 140.4, 138.3, 134.4, 130.6, 123.6, 122.4, 121.2, 117.0, 112.2, 112.1, 56.0; HRMS (ESI) *m/z*: calcd for $C_{14}H_{12}N_3O$ $[M+H]^+$ 238.0975; found 238.0979.	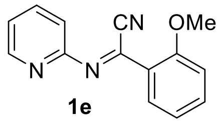 **1e**

*(Z)-3,4-dimethoxy-N-(pyridin-2-yl)benzimidoyl cyanide (**1f**):* Yellow solid, 203 mg, yield 76%; Purified by flash column chromatography (SiO_2, elution system Hexane/EtOAc 7/1 to 5/1); mp: (88-90 °C); ^1H NMR (500 MHz, $CDCl_3$) δ 8.59 (d, *J* = 4.5 Hz, 1H), 7.84 – 7.80 (m, 2H), 7.77 (s, 1H), 7.23 (d, *J* = 8.5 Hz, 2H), 6.98 (d, *J* = 8.5 Hz, 1H), 3.99 (s, 3H), 3.98 (s, 3H). $^{13}C\{H\}$ NMR (125 MHz, $CDCl_3$) δ 159.8, 154.0, 149.7, 149.1, 140.7, 138.4, 127.0, 124.7, 122.6, 117.9, 111.6, 110.7, 109.4, 56.4, 56.3; HRMS (ESI) *m/z*: calcd for $C_{15}H_{14}N_3O_2$ [M+H]$^+$ 268.1081; found 268.1084.	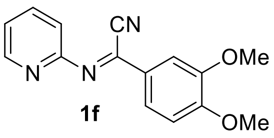 **1f**
*(Z)-4-chloro-N-(pyridin-2-yl)benzimidoyl cyanide (**1g**):*[S2] Yellow solid, 222 mg, yield 92%; Purified by flash column chromatography (SiO_2, elution system Hexane/EtOAc 10/1); mp: (120-121 °C); ^1H NMR (500 MHz, $CDCl_3$) δ 8.61 (d, *J* = 5.0 Hz, 1H), 8.18 (d, *J* = 8.5 Hz, 2H), 7.87 (t, *J* = 7.5 Hz, 1H), 7.53 (d, *J* = 8.5 Hz, 2H), 7.33 – 7.28 (m, 2H); $^{13}C\{H\}$ NMR (125 MHz, $CDCl_3$): δ 158.8, 148.9, 140.1, 140.0, 138.7, 132.3, 130.1, 129.6, 123.4, 118.9, 111.5; HRMS (ESI) *m/z*: calcd for $C_{13}H_9ClN_3$ [M+H]$^+$ 242.048; found 242.0482.	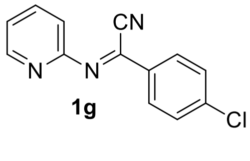 **1g**
*(Z)-4-bromo-N-(pyridin-2-yl)benzimidoyl cyanide (**1h**):*[S2] Yellow solid, 172 mg, yield 60%; Purified by flash column chromatography (SiO_2, elution system Hexane/EtOAc 10/1); mp: (110-111 °C); ^1H NMR (500 MHz, $CDCl_3$) δ 8.61 (dd, J_1 = 5.0 Hz, J_2 = 2.0 Hz, 1H), 8.10 (d, *J* = 8.5 Hz, 2H), 7.86 (td, J_1 = 7.5 Hz, J_2 = 2.0 Hz, 1H), 7.69 (d, *J* = 8.5 Hz, 2H), 7.34 – 7.24 (m, 2H); $^{13}C\{H\}$ NMR (125 MHz, $CDCl_3$) δ 158.8, 149.0, 140.0, 138.5, 132.8, 132.5, 130.2, 128.7, 123.4, 119.0, 111.5; HRMS (ESI) *m/z*: calcd for $C_{13}H_9BrN_3$ [M+H]$^+$ 285.9974 and 287.9954; found 285.9975 and 287.9963.	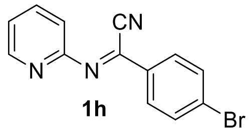 **1h**
*(Z)-methyl 4-(cyano(pyridin-2-ylimino)methyl)benzoate (**1j**):* Brown oil, 228 mg, yield 86%; Purified by flash column chromatography (SiO_2, elution system Hexane/EtOAc 10/1 to 7/1); ^1H NMR (500 MHz, $CDCl_3$) δ 8.62 (dd, J_1 = 5.0 Hz, J_2 = 2.0 Hz, 1H), 8.30 (d, *J* = 8.5 Hz, 2H), 8.19 (d, *J* = 8.5 Hz, 2H), 7.87 (td, J_1 = 8.0 Hz, J_2 = 2.0 Hz, 1H), 7.34 – 7.31 (m, 2H), 3.97 (s, 3H); $^{13}C\{H\}$ NMR (125 MHz, $CDCl_3$) δ 166.2, 158.7, 149.1, 140.0, 138.5, 137.4, 134.2, 130.2, 128.8, 123.7, 119.3, 111.6, 52.7; HRMS (ESI) *m/z*: calcd for $C_{15}H_{12}N_3O_2$ [M+H]$^+$ 266.0924; found 266.0925.	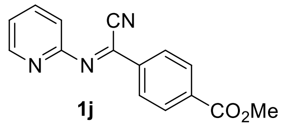 **1j**
*(Z)-N-(pyridin-2-yl)-1-naphthimidoyl cyanide (**1k**):*[S3] Brown oil, 231 mg, yield 90%; Purified by flash column chromatography (SiO_2, elution system Hexane/EtOAc 10/1); ^1H NMR (500 MHz, $CDCl_3$) δ 9.24 (d, *J* = 8.5 Hz, 1H), 8.66 (d, *J* = 4.0 Hz, 1H), 8.34 (d, *J* = 7.0 Hz, 1H), 8.08 (d, *J* = 8.0 Hz, 1H), 7.95 (d, *J* = 8.0 Hz, 1H), 7.88 (t, *J*= 7.5 Hz, 1H), 7.69 – 7.59 (m, 3H), 7.32 – 7.30 (m, 2H); $^{13}C\{H\}$ NMR (125 MHz, $CDCl_3$) δ 159.7, 149.1, 142.7, 138.5, 134.33, 134.25, 132.7, 130.5, 130.0, 129.1, 128.9, 127.1, 125.8, 124.9, 122.9, 117.9, 112.3; HRMS (ESI) *m/z*: calcd for $C_{17}H_{12}N_3$ [M+H]$^+$ 258.1026; found 258.1028.	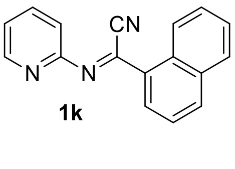 **1k**
*(E)-N-(pyridin-2-yl)thiophene-2-carbimidoyl cyanide (**1l**):*[S1] Brown solid, 106 mg, yield 50%; Purified by flash column chromatography (SiO_2, elution system Hexane/EtOAc 10/1); mp (56–58 °C); ^1H NMR (500 MHz, $CDCl_3$) δ 8.59 (d, *J* = 4.0 Hz, 1H), 7.95 (d, *J* = 3.5 Hz, 1H), 7.83 (t, *J* = 7.0 Hz, 1H), 7.66	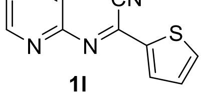 **1l**

(d, J = 4.5 Hz, 1H), 7.31-7.26 (m, 2H), 7.21 (t, J = 4.5 Hz, 1H); ^{13}C NMR (125 MHz, $CDCl_3$) δ 158.0, 148.6, 141.4, 138.7, 135.0, 134.5, 134.0, 128.7, 123.3, 119.7, 111.5; HRMS (ESI) *m/z*: calcd for $C_{11}H_8N_3S$ $[M+H]^+$ 214.0433; found 214.0437.	
*(Z)-4-methyl-N-(3-methylpyridin-2-yl)benzimidoyl cyanide (**1m**):* Brown oil, 188 mg, 80%; Purified by flash column chromatography (SiO_2, elution system Hexane/EtOAc 10/1); ^{1}H NMR (500 MHz, $CDCl_3$) δ 8.40 (d, J = 3.5 Hz, 1H), 8.13 (d, J = 8.2 Hz, 2H), 7.62 (d, J = 7.5 Hz, 1H), 7.34 (d, J = 8.2 Hz, 2H), 7.19 (dd, J_1 = 7.5 Hz, J_2 = 4.8 Hz, 1H), 2.46 (s, 3H), 2.36 (s, 3H); ^{13}C{H} NMR (125 MHz, $CDCl_3$) δ 157.8, 146.1, 144.3, 140.5, 139.2, 131.5, 129.9, 128.8, 128.0, 123.2, 112.0, 21.9, 17.4; HRMS (ESI) *m/z*: calcd for $C_{15}H_{14}N_3$ $[M+H]^+$ 236.1182; found 236.1190.	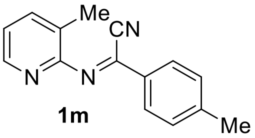 **1m**
*(Z)-4-methyl-N-(4-methylpyridin-2-yl)benzimidoyl cyanide (**1n**):*[S3] Yellow oil, 153 mg, yield 65%; Purified by flash column chromatography (SiO_2, elution system Hexane/EtOAc 10/1); ^{1}H NMR (500 MHz, $CDCl_3$) δ 8.43 (d, J = 5.0 Hz, 1H), 8.09 (d, J = 8.0 Hz, 2H), 7.32 (d, J = 8.0 Hz, 2H), 7.07 (d, J = 5.0 Hz, 1H), 7.05 (s, 1H), 2.44 (s, 3H), 2.41 (s, 3H); ^{13}C{H} NMR (125 MHz, $CDCl_3$) δ 159.5, 149.7, 148.5, 144.3, 140.96, 131.2, 129.7, 128.7, 123.8, 118.6, 111.6, 21.7, 21.0; HRMS (ESI) *m/z*: calcd for $C_{15}H_{14}N_3$ $[M+H]^+$ 236.1182; found 236.1188.	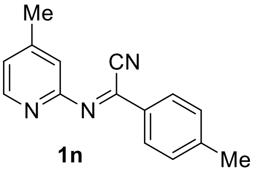 **1n**
*(Z)-4-methyl-N-(5-methylpyridin-2-yl)benzimidoyl cyanide (**1o**):* Orange oil, 160 mg, yield 68%; Purified by flash column chromatography (SiO_2, elution system Hexane/EtOAc 10/1); ^{1}H NMR (500 MHz, $CDCl_3$) δ 8.40 (d, J = 0.7 Hz, 1H), 8.09 (d, J = 8.0 Hz, 2H), 7.61 (dd, J_1 = 8.0 Hz, J_2 = 2.0 Hz, 1H), 7.31 (d, J = 8.0 Hz, 2H), 7.17 (d, J = 8.0 Hz, 1H), 2.43 (s, 3H), 2.38 (s, 3H); ^{13}C{H} NMR (125 MHz, $CDCl_3$) δ 157.0, 148.9, 144.1, 140.2, 138.7, 132.8, 131.3, 129.7, 128.6, 118.2, 111.9, 21.7, 18.2; HRMS (ESI) *m/z*: calcd for $C_{15}H_{14}N_3$ $[M+H]^+$ 236.1182; found 236.1189.	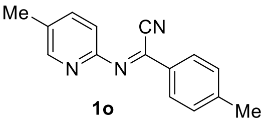 **1o**
*(Z)-4-methyl-N-(6-methylpyridin-2-yl)benzimidoyl cyanide (**1p**):* Yellow oil, 185 mg, yield 79%; Purified by flash column chromatography (SiO_2, elution system Hexane/EtOAc 10/1); ^{1}H NMR (500 MHz, $CDCl_3$) δ 8.10 (d, J = 8.3 Hz, 2H), 7.71 (t, J = 8.0 Hz, 1H), 7.33 (d, J = 8.0 Hz, 2H), 7.12 (d, J = 7.5 Hz, 1H), 7.03 (d, J = 7.8 Hz, 1H), 2.62 (s, 3H), 2.45 (s, 3H); ^{13}C{H} NMR (125 MHz, $CDCl_3$) δ 158.9, 158.2, 144.3, 141.1, 138.5, 131.1, 129.7, 128.7, 122.2, 114.5, 111.4, 23.9, 21.7; HRMS (ESI) *m/z*: calcd for $C_{15}H_{14}N_3$ $[M+H]^+$ 236.1182; found 236.1185.	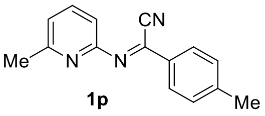 **1p**
*(Z)-N-(3-chloropyridin-2-yl)-4-methylbenzimidoyl cyanide (**1q**):* Yellow oil, 210 mg, yield 82%; Purified by flash column chromatography (SiO_2, elution system Hexane/EtOAc 10/1); ^{1}H NMR (500 MHz, $CDCl_3$) δ 8.47 (d, J = 3.8 Hz, 1H), 8.15 (d, J = 8.0 Hz, 2H), 7.84 (d, J = 8.0 Hz, 1H), 7.36 (d, J = 8.0 Hz, 2H), 7.23 (dd, J_1 = 8.0 Hz, J_2= 5.0 Hz, 1H), 2.47 (s, 3H); ^{13}C{H} NMR (125 MHz, $CDCl_3$) δ 156.1, 146.7, 145.0, 142.4, 138.5, 130.9, 129.9, 129.1, 125.5, 123.5, 111.2, 21.8; HRMS (ESI) *m/z*: calcd for $C_{14}H_{11}ClN_3$ $[M+H]^+$ 256.0636; found 256.0635.	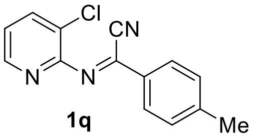 **1q**

(Z)-N-(5-chloropyridin-2-yl)-4-methylbenzimidoyl cyanide (***1r***)*:* Orange oil, 166 mg, yield 65%; Purified by flash column chromatography (SiO_2, elution system Hexane/EtOAc 10/1); ^1H NMR (500 MHz, $CDCl_3$) δ 8.51 (s, 1H), 8.07 (d, *J* = 8.0 Hz, 2H), 7.75 (dd, J_1 = 8.5 Hz, J_2 = 2.0 Hz, 1H), 7.31 (d, *J* = 8.0 Hz, 2H), 7.20 (d, *J* = 8.5 Hz, 1H), 2.43 (s, 3H); $^{13}C\{H\}$ NMR (125 MHz, $CDCl_3$) δ 157.3, 147.6, 144.7, 141.2, 137.9, 131.0, 130.8, 129.8, 128.8, 119.6, 111.5, 21.6; HRMS (ESI) *m/z*: calcd for $C_{14}H_{11}ClN_3$ $[M+H]^+$ 256.0636; found 256.0637.	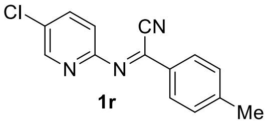 **1r**
(Z)-N-(5-bromopyridin-2-yl)-4-methylbenzimidoyl cyanide (***1s***)*:*[S3] Yellow oil, 210 mg, yield 70%; Purified by flash column chromatography (SiO_2, elution system Hexane/EtOAc 10/1); ^1H NMR (500 MHz, $CDCl_3$) δ 8.63 (d, *J* = 2.1 Hz, 1H), 8.10 (d, *J* = 8.0 Hz, 2H), 7.93 (dd, J_1 = 8.5 Hz, J_2 = 2.0 Hz, 1H), 7.34 (d, *J* = 8.0 Hz, 2H), 7.16 (d, *J* = 8.5 Hz, 1H), 2.46 (s, 3H); ^{13}C NMR (125 MHz, $CDCl_3$) δ 157.8, 149.9, 144.8, 141.3, 140.8, 131.0, 129.8, 128.9, 119.9, 119.3, 111.4, 21.8; HRMS (ESI) *m/z*: calcd for $C_{14}H_{11}BrN_3$ $[M+H]^+$ 300.0131 and 302.0110; found 300.0093 and 302.0117.	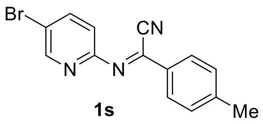 **1s**

^{1}H and ^{13}C{H} NMR data for imidates 2aa-2ad, 2aj, 2ba-2hc, 2ja-2sa

*(Z)-methyl 4-methyl-N-(pyridin-2-yl)benzimidate (**2aa**):* Yellow oil, 37 mg, yield 82%; Purified by flash column chromatography (SiO_2, elution system Hexane/EtOAc 7/1 to 2/1); ^{1}H NMR (500 MHz, $CDCl_3$) δ 8.33 (dd, J_1 = 5.0 Hz, J_2 = 1 Hz, 1H), 7.46 (td, J_1 = 7.5 Hz, J_2 = 2.0 Hz, 1H), 7.18 (d, J = 8.0 Hz, 2H), 7.01 (d, J = 8.0 Hz, 2H), 6.88 (ddd, J_1 = 7.0, J_2 = 5.0 Hz, J_3 = 1.0 Hz, 1H), 6.55 (d, J = 8.0 Hz, 1H), 4.00 (s, 3H), 2.27 (s, 3H); ^{13}C{H} NMR (125 MHz, $CDCl_3$) δ 161.29, 161.25, 148.8, 140.6, 137.6, 129.1, 128.7, 128.2, 118.1, 116.3, 54.3, 21.4; HRMS (ESI) *m/z*: calcd for $C_{14}H_{15}N_2O$ $[M+H]^+$ 227.1179; found 227.1203.	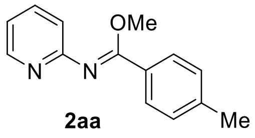 **2aa**
*(Z)-ethyl 4-methyl-N-(pyridin-2-yl)benzimidate (**2ab**):* Yellow oil, 35 mg, yield 74%; Purified by flash column chromatography (SiO_2, elution system Hexane/EtOAc 7/1 to 2/1); ^{1}H NMR (500 MHz, $CDCl_3$) δ 8.33 (d, J = 5.0 Hz, 1H), 7.44 (td, J_1 = 7.5 Hz, J_2 = 2.0 Hz, 1H), 7.19 (d, J = 8.0 Hz, 2H), 7.01 (d, J = 8.0 Hz, 2H), 6.87 (dd, J_1 = 7.0, J_2 = 5.0 Hz, 1H), 6.54 (d, J = 8.0 Hz, 1H), 4.45 (q, J = 7.0 Hz, 2H), 2.27 (s, 3H), 1.43 (t, J = 7.0 Hz, 3H); ^{13}C{H} NMR (125 MHz, $CDCl_3$) δ 161.5, 160.8, 148.8, 140.4, 137.5, 129.1, 128.7, 128.6, 118.0, 116.3, 62.8, 21.4, 14.3; HRMS (ESI) *m/z*: calcd for $C_{15}H_{17}N_2O$ $[M+H]^+$ 241.1335; found 241.1387.	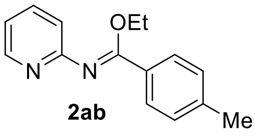 **2ab**
*(Z)-propyl 4-methyl-N-(pyridin-2-yl)benzimidate (**2ac**):* Light yellow oil, 29 mg, yield 56%; Purified by flash column chromatography (SiO_2, elution system Hexane/EtOAc 7/1 to 2/1); ^{1}H NMR (500 MHz, $CDCl_3$) δ 8.31 (dd, J_1 = 5.0 Hz, J_2 = 1.0 Hz, 1H), 7.43 (td, J_1 = 7.5 Hz, J_2 = 2.0 Hz, 1H), 7.18 (d, J = 8.0 Hz, 2H), 6.99 (d, J = 8.0 Hz, 2H), 6.85 (dd, J_1 = 7.0, J_2 = 5.0 Hz, 1H), 6.53 (d, J = 8.0 Hz, 1H), 4.33 (t, J = 6.5 Hz, 2H), 2.26 (s, 3H), 1.87 – 1.79 (m, 2H), 1.04 (t, J = 7.5 Hz, 3H); ^{13}C{H} NMR (125 MHz, $CDCl_3$) δ 161.4, 161.0, 147.9, 140.7, 138.2, 129.1, 128.7, 128.4, 118.0, 116.6, 68.9, 22.0, 21.4, 10.7; HRMS (ESI) *m/z*: calcd for $C_{16}H_{19}N_2O$ $[M+H]^+$ 255.1492; found 255.1543.	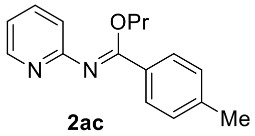 **2ac**
*(Z)-butyl 4-methyl-N-(pyridin-2-yl)benzimidate (**2ad**):* Yellow oil, 41 mg, yield 77%; Purified by flash column chromatography (SiO_2, elution system Hexane/EtOAc 7/1 to 2/1); ^{1}H NMR (500 MHz, $CDCl_3$) δ 8.32 (d, J = 5.0 Hz, 1H), 7.47 (td, J_1 = 8.0 Hz, J_2 = 2.0 Hz, 1H), 7.18 (d, J = 8.0 Hz, 2H), 7.01 (d, J = 8.0 Hz, 2H), 6.88 (dd, J_1 = 7.0 Hz, J_2 = 5.5 Hz, 1H), 6.55 (d, J = 8.0 Hz, 1H), 4.38 (t, J = 6.5 Hz, 2H), 2.27 (s, 3H), 1.83 – 1.76 (m, 2H), 1.51 (sextet, J = 7.5 Hz, 2H), 0.98 (t, J = 7.5 Hz, 3H); ^{13}C{H} NMR (125 MHz, $CDCl_3$) δ 161.3, 161.1, 148.1, 140.6, 138.0, 129.1, 128.7, 128.4, 118.0, 116.5, 67.1, 30.7, 21.3, 19.4, 13.9; HRMS (ESI) *m/z*: calcd for $C_{17}H_{21}N_2O$ $[M+H]^+$ 269.1648; found 269.1655.	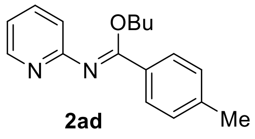 **2ad**
*(Z)-3-hydroxypropyl 4-methyl-N-(pyridin-2-yl)benzimidate (**2aj**):* Brown oil, 16 mg, yield 30%; Purified by flash column chromatography (SiO_2, elution system Hexane/EtOAc 5/1 to 1/1); ^{1}H NMR (500 MHz, $CDCl_3$) δ 8.32 (d, J = 5.0 Hz, 1H), 7.48 (t, J = 8.0 Hz, 1H), 7.18 (d, J = 8.0 Hz, 2H), 7.02	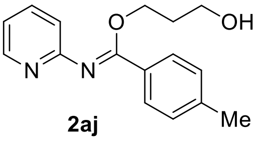 **2aj**

(d, *J* = 8.0 Hz, 2H), 6.90 (dd, J_1 = 7.0 Hz, J_2 = 5.5 Hz, 1H), 6.59 (d, *J* = 8.0 Hz, 2H), 4.59 (t, *J* = 6.0 Hz, 2H), 3.83 (t, *J* = 6.0 Hz, 2H), 2.28 (s, 3H), 2.07 – 2.02 (m, 2H); $^{13}C\{H\}$ NMR (125 MHz, $CDCl_3$) δ 161.7, 160.9, 148.8, 140.8, 137.7, 129.1, 128.8, 128.2, 118.3, 116.4, 63.9, 59.3, 32.1, 21.4; MS (ESI) *m/z*: calcd for $C_{16}H_{19}N_2O_2$ $[M+H]^+$ 271.14; found 271.19 (sensitive compound)	
*(Z)-methyl N-pyridin-2-ylbenzimidate (**2ba**):*[S4] Brown oil, 35 mg, yield 83%; Purified by flash column chromatography (SiO_2, elution system Hexane/EtOAc 7/1 to 2/1); ^1H NMR (500 MHz, $CDCl_3$): δ 8.32 (dd, J_1 = 5.0 Hz, J_2 = 1.0 Hz, 1H), 7.46 (td, J_1 = 8.0 Hz, J_2 = 2.0 Hz, 1H), 7.32 – 7.28 (m, 3H), 7.23 – 7.18 (m, 2H), 6.88 (dd, J_1 = 7.0 Hz, J_2 = 5.0 Hz, 1H), 6.56 (d, *J* = 8.0 Hz, 1H), 4.02 (s, 3H); $^{13}C\{H\}$ NMR (125 MHz, $CDCl_3$) δ 161.4, 161.0, 148.6, 137.8, 131.2, 130.3, 129.1, 128.0, 118.3, 116.4, 54.5; HRMS (ESI) *m/z*: calcd for $C_{13}H_{13}N_2O$ $[M+H]^+$ 213.1022; found 213.1026.	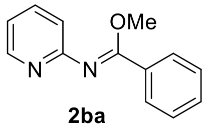 **2ba**
*(Z)-ethyl N-pyridin-2-ylbenzimidate (**2bb**):* Brown oil, 38 mg, yield 84%; Purified by flash column chromatography (SiO_2, elution system Hexane/EtOAc 7/1 to 2/1); ^1H NMR (500 MHz, $CDCl_3$) δ 8.31 (dd, J_1 = 5.0 Hz, J_2 = 1.0 Hz, 1H), 7.43 (td, J_1 = 8.0 Hz, J_2 = 2.0 Hz, 1H), 7.31 – 7.27 (m, 3H), 7.20 (t, *J* = 7.5 Hz, 2H), 6.85 (ddd, J_1 = 7.0 Hz, J_2 = 5.0 Hz, J_3 = 1.0 Hz, 1H), 6.54 (d, *J* = 8.0 Hz, 1H), 4.46 (q, *J* = 7.0 Hz, 2H), 1.44 (t, *J* = 7.0 Hz, 3H); $^{13}C\{H\}$ NMR (125 MHz, $CDCl_3$) δ 161.1, 161.0, 148.3, 137.8, 131.4, 130.2, 129.1, 128.0, 118.1, 116.5, 63.1, 14.2; HRMS (ESI) *m/z*: calcd for $C_{14}H_{15}N_2O$ $[M+H]^+$ 227.1179; found 227.1225.	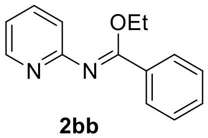 **2bb**
*(Z)-propyl N-pyridin-2-ylbenzimidate (**2bc**):* Brown oil, 37 mg, yield 77%; Purified by flash column chromatography (SiO_2, elution system Hexane/EtOAc 7/1 to 2/1); ^1H NMR (500 MHz, $CDCl_3$) δ 8.31 (dd, J_1 = 5.0 Hz, J_2 = 1.0 Hz, 1H), 7.44 (ddd, J_1 = 8.0 Hz, J_2 = 7.5 Hz, J_3 = 2.0 Hz, 1H), 7.31 – 7.27 (m, 3H), 7.20 (t, *J* = 8.0 Hz, 2H), 6.86 (ddd, J_1 = 7.0 Hz, J_2 = 5.0 Hz, J_3 = 1.0 Hz, 1H), 6.55 (d, *J* = 8.0 Hz, 1H), 4.36 (t, *J* = 6.5 Hz, 2H), 1.89 – 1.81 (m, 2H), 1.05 (t, *J* = 7.5 Hz, 3H); $^{13}C\{H\}$ NMR (125 MHz, $CDCl_3$) δ 161.2, 161.0, 148.7, 137.6, 131.6, 130.1, 129.1, 128.0, 118.1, 116.4, 68.7, 22.0, 10.7; HRMS (ESI) *m/z*: calcd for $C_{15}H_{17}N_2O$ $[M+H]^+$ 241.1335; found 241.1339.	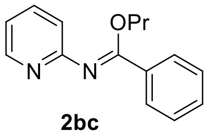 **2bc**
*(Z)-methyl 4-methoxy-N-(pyridin-2-yl)benzimidate (**2ca**):* Yellow oil, 47 mg, yield 98%; Purified by flash column chromatography (SiO_2, elution system Hexane/EtOAc 2/1); ^1H NMR (500 MHz, $CDCl_3$) δ 8.33 (dd, J_1 = 5.0 Hz, J_2= 1.0 Hz, 1H), 7.47 (td, J_1 = 8.0 Hz, J_2= 2.0 Hz, 1H), 7.24 (d, *J* = 9.0 Hz, 2H), 6.88 (ddd, J_1 = 7.0 Hz, J_2= 5.0 Hz, J_3= 1.0 Hz, 1H), 6.70 (d, *J* = 9.0 Hz, 2H), 6.57 (d, *J* = 8.0 Hz, 1H), 3.99 (s, 3H), 3.73 (s, 3H); $^{13}C\{H\}$ NMR (125 MHz, $CDCl_3$) δ 161.4, 161.0, 160.7, 148.8, 137.7, 130.9, 123.3, 118.0, 116.3, 113.3, 55.2, 54.3; HRMS (ESI) *m/z*: calcd for $C_{14}H_{15}N_2O_2$ $[M+H]^+$ 243.1128; found 243.1136.	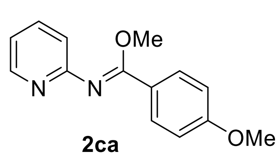 **2ca**
*(Z)-ethyl 4-methoxy-N-(pyridin-2-yl)benzimidate (**2cb**):* Brown oil, 49 mg, yield 96%; Purified by flash column chromatography (SiO_2, elution system Hexane/EtOAc 2/1); ^1H NMR (500 MHz, $CDCl_3$) δ 8.33 (d, *J* = 5.0	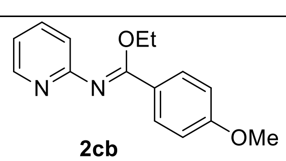 **2cb**

Hz, 1H), 7.46 (t, *J* = 8.0 Hz, 1H), 7.26 (d, *J* = 9.0 Hz, 2H), 6.87 (dd, J_1 = 7.0 Hz, J_2 = 5.0 Hz, 1H), 6.70 (d, *J* = 9 Hz, 2H), 6.55 (d, *J* = 8.0 Hz, 1H), 4.43 (q, *J* = 7.0 Hz, 2H), 3.74 (s, 3H), 1.43 (t, *J* = 7.0 Hz, 3H); ^{13}C{H} NMR (125 MHz, $CDCl_3$) δ 161.5, 160.9, 160.3, 148.7, 137.6, 131.0, 123.6, 117.9, 116.3, 113.3, 62.8, 55.2, 14.3; HRMS (ESI) *m/z*: calcd for $C_{15}H_{17}N_2O_2$ $[M+H]^+$ 257.1285; found 257.1290.	
*(Z)-propyl 4-methoxy-N-(pyridin-2-yl)benzimidate (**2cc**):* Yellow oil, 49 mg, yield 91%; Purified by flash column chromatography (SiO_2, elution system Hexane/EtOAc 7/1 to 2/1); ^{1}H NMR (500 MHz, $CDCl_3$) δ 8.32 (d, *J* = 5.0 Hz, 1H), 7.46 (td, J_1 = 8.0 Hz, J_2 = 2.0 Hz, 1H), 7.25 (d, *J* = 9.0 Hz, 2H), 6.86 (dd, J_1 = 7.0 Hz, J_2 = 5.0 Hz, 1H), 6.70 (d, *J* = 9.0 Hz, 2H), 6.56 (d, *J* = 8.0 Hz, 1H), 4.33 (t, *J* = 6.5 Hz, 2H), 3.73 (s, 3H), 1.87 – 1.80 (m, 2H), 1.05 (t, *J* = 7.5 Hz, 3H); ^{13}C{H} NMR (125 MHz, $CDCl_3$) δ 161.5, 161.0, 160.4, 148.7, 137.7, 131.0, 123.6, 117.9, 116.3, 113.3, 68.6, 55.2, 22.0, 10.7; HRMS (ESI) *m/z*: calcd for $C_{16}H_{19}N_2O_2$ $[M+H]^+$ 271.1441; found 271.1448.	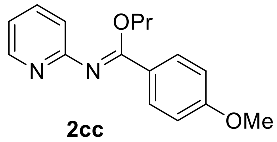 **2cc**
*(Z)-methyl 3-methoxy-N-(pyridin-2-yl)benzimidate (**2da**):* Yellow oil, 43 mg, yield 89%; Purified by flash column chromatography (SiO_2, elution system Hexane/EtOAc 7/1 to 2/1); ^{1}H NMR (500 MHz, $CDCl_3$) δ 8.33 (dd, J_1 = 5.0 Hz, J_2 = 1.0 Hz, 1H), 7.46 (td, J_1 = 7.5 Hz, J_2 = 1.5 Hz, 1H), 7.11 (t, *J* = 8.0 Hz, 1H), 6.90 – 6.86 (m, 2H), 6.85 – 6.81 (m, 2H), 6.56 (d, *J* = 8.0 Hz, 1H), 4.01 (s, 3H), 3.60 (s, 3H); ^{13}C{H} NMR (125 MHz, $CDCl_3$) δ 161.1, 161.0, 159.0, 148.7, 137.7, 132.3, 129.1, 121.5, 118.2, 116.9, 116.3, 113.9, 55.1, 54.4; HRMS (ESI) *m/z*: calcd for $C_{14}H_{15}N_2O_2$ $[M+H]^+$ 243.1128; found 243.1168.	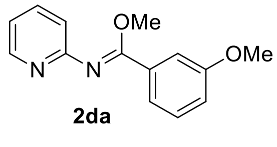 **2da**
*(Z)-ethyl 3-methyl-N-(pyridin-2-yl)benzimidate (**2db**):* Brown oil, 46 mg, yield 91%; Purified by flash column chromatography (SiO_2, elution system Hexane/EtOAc 2/1); ^{1}H NMR (500 MHz, $CDCl_3$) δ 8.32 (dd, *J* = 5.0 Hz, 1.0 Hz, 1H), 7.45 (td, J_1 = 7.5 Hz, J_2 = 1.5 Hz, 1H), 7.11 (t, *J* = 8.0 Hz, 1H), 6.90 – 6.82 (m, 4H), 6.55 (d, *J* = 8.0 Hz, 1H), 4.45 (q, *J* = 7.0 Hz, 2H), 3.60 (s, 3H), 1.43 (t, *J* = 7.0 Hz, 3H); ^{13}C{H} NMR (125 MHz, $CDCl_3$) δ 161.2, 160.7, 159.0, 148.5, 137.8, 132.6, 129.1, 121.5, 118.1, 116.7, 116.4, 114.0, 63.1, 55.1, 14.2; HRMS (ESI) *m/z*: calcd for $C_{15}H_{17}N_2O_2$ $[M+H]^+$ 257.1285; found 257.1336.	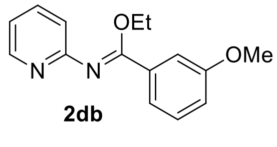 **2db**
*(Z)-propyl 3-methoxy-N-(pyridin-2-yl)benzimidate (**2dc**):* Yellow oil, 46 mg, yield 86%; Purified by flash column chromatography (SiO_2, elution system Hexane/EtOAc 7/1 to 2/1); ^{1}H NMR (500 MHz, $CDCl_3$) δ 8.33 (dd, *J* = 5.0 Hz, 1.0 Hz, 1H), 7.47 (t, *J* = 7.5 Hz, 1H), 7.13 (t, *J* = 8.0 Hz, 1H), 6.91 – 6.82 (m, 4H), 6.56 (d, *J* = 8.0 Hz, 1H), 4.36 (t, *J* = 6.5 Hz, 2H), 3.61 (s, 3H), 1.89 – 1.82 (m, 2H), 1.06 (t, *J* = 7.5 Hz, 3H); ^{13}C{H} NMR (125 MHz, $CDCl_3$) δ 161.2, 160.8, 158.9, 148.6, 137.7, 132.7, 129.1, 121.5, 118.1, 116.6, 116.4, 114.0, 68.8, 55.1, 22.0, 10.7; HRMS (ESI) *m/z*: calcd for $C_{16}H_{19}N_2O_2$ $[M+H]^+$ 271.1441; found 271.1443.	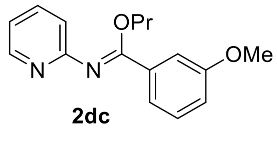 **2dc**

*(Z)-methyl 2-methoxy-N-(pyridin-2-yl)benzimidate (**2ea**):* Brown oil, 39 mg, yield 82%; Purified by flash column chromatography (SiO_2, elution system Hexane/EtOAc 7/1 to 2/1); ^{1}H NMR (500 MHz, $CDCl_3$) δ 8.23 (dd, J_1 = 5.0 Hz, J_2 = 1.0 Hz, 1H), 7.38 (td, J_1 = 8.0 Hz, J_2 = 1.5 Hz, 1H), 7.29 – 7.23 (m, 2H), 6.86 (t, J = 7.5 Hz, 1H), 6.79 (dd, J_1 = 7.0 Hz, J_2 = 5.5 Hz, 1H), 6.71 (d, J = 8.5 Hz, 1H), 6.57 (d, J = 8.0 Hz,), 4.03 (s, 3H), 3.54 (s, 3H); ^{13}C{H} NMR (125 MHz, $CDCl_3$) δ 161.5, 160.9, 155.9, 148.2, 136.9, 131.1, 130.0, 121.7, 120.3, 118.2, 116.1, 110.8, 55.2, 54.4; HRMS (ESI) *m/z*: calcd for $C_{14}H_{15}N_2O_2$ [M+H]$^+$ 243.1128; found 243.1133.	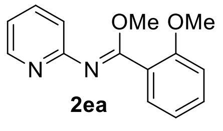 **2ea**
*(Z)-methyl 3,4-dimethoxy-N-(pyridin-2-yl)benzimidate (**2fa**):* Yellow oil, 53 mg, yield 98%; Purified by flash column chromatography (SiO_2, elution system Hexane/EtOAc 2/1); ^{1}H NMR (500 MHz, $CDCl_3$) δ 8.34 (dd, J_1 = 5.0 Hz, J_2 = 1.0 Hz, 1H), 7.47 (td, J_1 = 8.0 Hz, J_2 = 2.0 Hz, 1H), 6.96 (dd, J_1 = 8.0 Hz, J_2 = 2.0 Hz, 1H), 6.88 (ddd, J_1 = 7.0 Hz, J_2 = 5.0 Hz, J_3 = 1.0 Hz, 1H), 6.76 (d, J = 2.0 Hz, 1H), 6.69 (d, J = 8.5 Hz, 1H), 6.57 (d, J = 8.0 Hz, 1H), 3.99 (s, 3H), 3.81 (s, 3H), 3.58 (s, 3H); ^{13}C{H} NMR (125 MHz, $CDCl_3$) δ 161.6, 160.5, 150.6, 148.8, 148.0, 137.7, 123.3, 122.7, 118.0, 116.3, 112.2, 110.2, 55.8, 55.6, 54.3; HRMS (ESI) *m/z*: calcd for $C_{15}H_{17}N_2O_3$ [M+H]$^+$ 273.1234; found 273.1282.	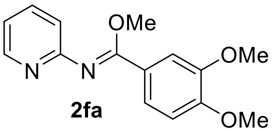 **2fa**
*(Z)-ethyl 3,4-dimethoxy-N-(pyridin-2-yl)benzimidate (**2fb**):* Brown oil, 56 mg, yield 98%; Purified by flash column chromatography (SiO_2, elution system Hexane/EtOAc 2/1); ^{1}H NMR (500 MHz, $CDCl_3$) δ 8.33 (dd, J_1 = 5.0, J_2 = 1.0 Hz, 1H), 7.45 (td, J_1 = 8.0 Hz, J_2 = 2.0 Hz, 1H), 6.99 (dd, J_1 = 8.0 Hz, J_2 = 2.0 Hz, 1H), 6.86 (ddd, J_1 = 7.5 Hz, J_2 = 5.0 Hz, J_3 = 1.0 Hz, 1H), 6.73 (d, J = 2.0 Hz, 1H), 6.69 (d, J = 8.5 Hz, 1H), 6.55 (d, J = 8.0 Hz, 1H), 4.43 (q, J = 7.0 Hz, 2H), 3.80 (s, 3H), 3.56 (s, 3H), 1.43 (t, J = 7.0 Hz, 3H); ^{13}C{H} NMR (125 MHz, $CDCl_3$) δ 161.8, 160.1, 150.5, 148.8, 148.0, 137.7, 123.6, 122.7, 117.9, 116.3, 112.3, 110.2, 62.8, 55.8, 55.5, 14.3; HRMS (ESI) *m/z*: calcd for $C_{16}H_{19}N_2O_3$ [M+H]$^+$ 287.1390; found 287.1434.	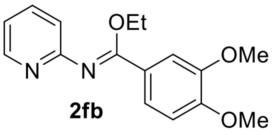 **2fb**
*(Z)-propyl 3,4-dimethoxy-N-(pyridin-2-yl)benzimidate (**2fc**):* Brown oil, 54 mg, yield 90%; Purified by flash column chromatography (SiO_2, elution system Hexane/EtOAc 7/1 to 2/1); ^{1}H NMR (500 MHz, $CDCl_3$) δ 8.33 (ddd, J_1 = 5.0 Hz, J_2 = 2.0 Hz, J_3 = 1.0 Hz, 1H), 7.45 (ddd, J_1 = 8.0 Hz, J_2 = 7.5 Hz, J_3 = 2.0 Hz, 1H), 7.00 (dd, J_1 = 8.5 Hz, J_2 = 2.0 Hz, 1H), 6.86 (ddd, J_1 = 7.5 Hz, J_2 = 5.0, J_3 = 1.0 Hz, 1H), 6.73 (d, J = 2.0 Hz, 1H), 6.70 (d, J = 8.5 Hz, 1H), 6.55 (d, J = 8.0 Hz, 1H), 4.32 (t, J = 6.5 Hz, 2H), 3.80 (s, 3H), 3.56 (s, 3H), 1.88 – 1.80 (m, 2H), 1.04 (t, J = 7.5 Hz, 3H); ^{13}C{H} NMR (125 MHz, $CDCl_3$) δ 161.8, 160.2, 150.5, 148.7, 147.9, 137.7, 123.7, 122.7, 117.9, 116.3, 112.3, 110.2, 68.6, 55.8, 55.5, 22.0, 10.7; HRMS (ESI) *m/z*: calcd for $C_{17}H_{21}N_2O_3$ [M+H]$^+$ 301.1547; found 301.1440.	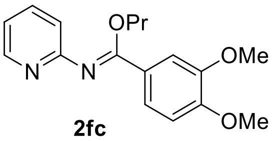 **2fc**
*(Z)-methyl 4-chloro-N-(pyridin-2-yl)benzimidate (**2ga**):*[S5] Brown oil, 48 mg, yield 98%; Purified by flash column chromatography (SiO_2, elution system Hexane/EtOAc 2/1); ^{1}H NMR (500 MHz, $CDCl_3$) δ 8.31 (dd, J_1 = 5.0	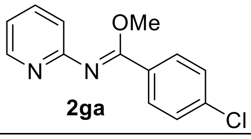 **2ga**

Hz, J_2 = 1.0 Hz, 1H), 7.48 (td, J_1 = 7.5 Hz, J_2 = 2.0 Hz, 1H), 7.23 (d, J = 8.5 Hz, 2H), 7.18 (d, J = 8.5 Hz, 2H), 6.90 (dd, J_1 = 7.0 Hz, J_2 = 5.5 Hz, 1H), 6.58 (d, J = 8.0 Hz, 1H), 4.00 (s, 3H); ^{13}C{H} NMR (125 MHz, $CDCl_3$) δ 160.8, 160.1, 148.9, 137.8, 136.4, 130.5, 129.6, 128.3, 118.4, 116.2, 54.5; HRMS (ESI) *m/z*: calcd for $C_{13}H_{12}ClN_2O$ $[M+H]^+$ 247.0633 and 249.0603; found 247.0682 and 249.0635.	
*(Z)-ethyl 4-chloro-N-(pyridin-2-yl)benzimidate (**2gb**):* White oil, 48 mg, yield 92%; Purified by flash column chromatography (SiO_2, elution system Hexane/EtOAc 7/1 to 2/1); ^{1}H NMR (500 MHz, $CDCl_3$) δ 8.31 (dd, J_1 = 5.0 Hz, J_2 = 1.0 Hz, 1H), 7.48 (td, J_1 = 7.5 Hz, J_2 = 2.0 Hz, 1H), 7.24 (d, J = 8.5 Hz, 2H), 7.18 (d, J = 8.5 Hz, 2H), 6.89 (ddd, J_1 = 7.0 Hz, J_2 = 5.0 Hz, J_3 = 1.0 Hz, 1H), 6.57 (d, J = 8.0 Hz, 1H), 4.45 (q, J = 7.0 Hz, 2H), 1.43 (t, J = 7.0 Hz, 3H); ^{13}C{H} NMR (125 MHz, $CDCl_3$) δ 160.9, 159.6, 148.9, 137.7, 136.3, 130.5, 129.9, 128.3, 118.3, 116.2, 63.1, 14.2; HRMS (ESI) *m/z*: calcd for $C_{14}H_{14}ClN_2O$ $[M+H]^+$ 261.0790 and 263.0760; found 261.0843 and 263.0819.	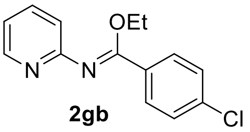 2gb
*(Z)-propyl 4-chloro-N-(pyridin-2-yl)benzimidate (**2gc**):* Brown oil, 43 mg, yield 78%; Purified by flash column chromatography (SiO_2, elution system Hexane/EtOAc 7/1 to 2/1); ^{1}H NMR (500 MHz, $CDCl_3$) δ 8.32 (d, J = 5.0 Hz, 1H), 7.53 (td, J_1 = 8.0, J_2 = 2.0 Hz, 1H), 7.25 (d, J = 8.5 Hz, 2H), 7.20 (d, J = 8.5 Hz, 2H), 6.93 (dd, J_1 = 7.0 Hz, J_2 = 5.0 Hz, 1H), 6.60 (d, J = 8.0 Hz, 1H), 4.36 (t, J = 6.5 Hz, 2H), 1.89 – 1.81 (m, 2H), 1.06 (t, J = 7.5 Hz, 3H); ^{13}C{H} NMR (125 MHz, $CDCl_3$) δ 160.6, 160.1, 148.3, 138.2, 136.5, 130.5, 129.8, 128.4, 118.4, 116.5, 69.1, 22.0, 10.7; HRMS (ESI) *m/z*: calcd for $C_{15}H_{16}ClN_2O$ $[M+H]^+$ 275.0946; found 275.0952.	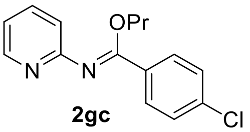 2gc
*(Z)-methyl 4-bromo-N-(pyridin-2-yl)benzimidate (**2ha**):* Yellow oil, 54 mg, yield 93%; Purified by flash column chromatography (SiO_2, elution system Hexane/EtOAc 7/1 to 2/1); ^{1}H NMR (500 MHz, $CDCl_3$) δ 8.31 (dd, J_1 = 5.0 Hz, J_2 = 1.0 Hz, 1H), 7.51 (td, J_1 = 8.0 Hz, J_2 = 2.0 Hz, 1H), 7.35 (d, J = 8.5 Hz, 2H), 7.16 (d, J = 8.5 Hz, 2H), 6.92 (ddd, J_1 = 7.0 Hz, J_2 = 5.0 Hz, J_3 = 1.0 Hz, 1H), 6.59 (d, J = 8.0 Hz, 1H), 4.00 (s, 3H); ^{13}C{H} NMR (125 MHz, $CDCl_3$) δ 161.23, 159.82, 147.17, 139.17, 131.47, 130.71, 129.68, 125.32, 118.56, 116.87, 55.00; HRMS (ESI) *m/z*: calcd for $C_{13}H_{12}BrN_2O$ $[M+H]^+$ 291.0128 and 293.0107; found 291.0186 and 293.0164.	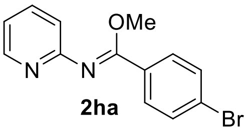 2ha
*(Z)-ethyl 4-bromo-N-(pyridin-2-yl)benzimidate (**2hb**):* Brown oil, 58 mg, yield 96%; Purified by flash column chromatography (SiO_2, elution system Hexane/EtOAc 2/1); ^{1}H NMR (500 MHz, $CDCl_3$) δ 8.30 (dd, J_1 = 5.0 Hz, J_2 = 1.0 Hz, 1H), 7.49 (ddd, J_1 = 8.0 Hz, J_2 = 7.5 Hz, J_3 = 2.0 Hz, 1H), 7.34 (d, J = 8.5 Hz, 2H), 7.17 (d, J = 8.5 Hz, 2H), 6.89 (ddd, J_1 = 7.0 Hz, J_2 = 5.0 Hz, J_3 = 1.0 Hz, 1H), 6.57 (d, J = 8.0 Hz, 1H), 4.44 (q, J = 7.0 Hz, 2H), 1.43 (t, J = 7.0 Hz, 3H); ^{13}C{H} NMR (125 MHz, $CDCl_3$) δ 160.7, 159.9, 148.6, 137.9, 131.3, 130.7, 130.3, 124.8, 118.3, 116.3, 63.2, 14.2; HRMS (ESI) *m/z*: calcd for $C_{14}H_{14}BrN_2O$ $[M+H]^+$ 305.0284 and 307.0264; found 305.0347 and 307.0328.	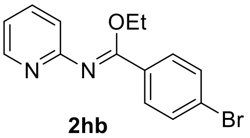 2hb

*(Z)-propyl 4-bromo-N-(pyridin-2-yl)benzimidate (**2hc**):* Brown oil, 58 mg, yield 91%; Purified by flash column chromatography (SiO_2, elution system Hexane/EtOAc 7/1 to 2/1); ^{1}H NMR (500 MHz, $CDCl_3$) δ 8.30 (dd, J_1 = 5.0 Hz, J_2 = 1.0 Hz, 1H), 7.49 (td, J_1 = 8.0 Hz, J_2 = 2.0 Hz, 1H), 7.34 (d, J = 8.5 Hz, 2H), 7.17 (d, J = 8.5 Hz, 2H), 6.89 (dd, J_1 = 7.0 Hz, J_2 = 5.0 Hz, 1H), 6.58 (d, J = 8.0 Hz, 1H), 4.34 (t, J = 6.5 Hz, 2H), 1.87 – 1.79 (m, 2H), 1.04 (t, J = 7.5 Hz, 3H); ^{13}C{H} NMR (125 MHz, $CDCl_3$) δ 160.7, 160.0, 148.6, 137.9, 131.3, 130.7, 130.4, 124.8, 118.3, 116.3, 69.0, 22.0, 10.7; HRMS (ESI) *m/z*: calcd for $C_{15}H_{16}BrN_2O$ $[M+H]^+$ 319.0441 and 321.0420; found 319.0470 and 321.0488.	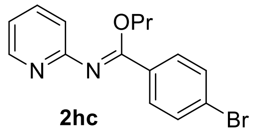 **2hc**
*(Z)-methyl 4-(methoxy(pyridin-2-ylimino)methyl)benzoate (**2ja**):* Yellow oil, 44 mg, yield 82%; Purified by flash column chromatography (SiO_2, elution system Hexane/EtOAc 8/1 to 2/1); ^{1}H NMR (500 MHz, $CDCl_3$) δ 8.28 (d, J = 5.0 Hz, 1H), 7.86 (d, J = 8.0 Hz, 2H), 7.46 (t, J = 7.5 Hz, 1H), 7.34 (d, J = 8.0 Hz, 2H), 6.88 (t, J = 5.0 Hz, 1H), 6.57 (d, J = 8.0 Hz, 1H), 4.02 (s, 3H), 3.85 (s, 3H); ^{13}C{H} NMR (125 MHz, $CDCl_3$) δ 166.2, 160.52, 160.49, 148.7, 137.8, 135.4, 131.4, 129.2, 129.0, 118.6, 116.3, 54.6, 52.3; HRMS (ESI) *m/z*: calcd for $C_{15}H_{15}N_2O_3$ $[M+H]^+$ 271.1077; found 271.1079.	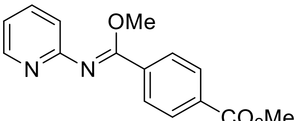 **2ja**
*(Z)-ethyl 4-(ethoxy(pyridin-2-ylimino)methyl)benzoate (**2jb**):* Yellow oil, 51 mg, yield 86%; Purified by flash column chromatography (SiO_2, elution system Hexane/EtOAc 7/1 to 2/1); ^{1}H NMR (500 MHz, $CDCl_3$) δ 8.29 (d, J = 5.0 Hz, 1H), 7.88 (d, J = 8.0 Hz, 2H), 7.47 (td, J_1 = 7.5 Hz, J_2 = 1.0 Hz, 1H), 7.36 (d, J = 8.0 Hz, 2H), 6.89 (dd, J_1 = 7.0 Hz, J_2 = 5.0 Hz, 1H), 6.57 (d, J = 8.0 Hz, 1H), 4.48 (q, J = 7.0 Hz, 2H), 4.33 (q, J = 7.0 Hz, 2H), 1.45 (t, J = 7.0 Hz, 3H), 1.35 (t, J = 7.0 Hz, 3H); ^{13}C{H} NMR (125 MHz, $CDCl_3$) δ 165.8, 160.6, 160.2, 148.6, 137.9, 135.6, 131.7, 129.2, 129.0, 118.4, 116.4, 63.3, 61.2, 14.24, 14.21; HRMS (ESI) *m/z*: calcd for $C_{17}H_{19}N_2O_3$ $[M+H]^+$ 299.1390; found 299.1392.	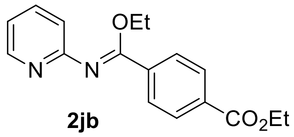 **2jb**
*(Z)-propyl 4-(propoxy(pyridin-2-ylimino)methyl)benzoate (**2jc**):* Brown oil, 63 mg, yield 96%; Purified by flash column chromatography (SiO_2, elution system Hexane/EtOAc 2/1); ^{1}H NMR (500 MHz, $CDCl_3$) δ 8.28 (dd, J_1 = 5.0 Hz, J_2 = 1.0 Hz, 1H), 7.88 (d, J = 8.0 Hz, 2H), 7.46 (td, J_1 = 7.5 Hz, J_2 = 1.0 Hz, 1H), 7.35 (d, J = 8.0 Hz, 2H), 6.88 (dd, J_1 = 7.0 Hz, J_2 = 5.0 Hz, 1H), 6.57 (d, J = 8.0 Hz, 1H), 4.36 (t, J = 6.5 Hz, 2H), 4.22 (t, J = 6.5 Hz, 2H), 1.88 – 1.80 (m, 2H), 1.77 – 1.69 (m, 2H), 1.04 (t, J = 7.5 Hz, 3H), 0.97 (t, J = 7.5 Hz, 3H); ^{13}C{H} NMR (125 MHz, $CDCl_3$) δ 165.8, 160.7, 160.3, 148.7, 137.8, 135.7, 131.7, 129.1, 129.0, 118.4, 116.3, 69.0, 66.8, 22.00, 21.97, 10.7, 10.5; HRMS (ESI) *m/z*: calcd for $C_{19}H_{23}N_2O_3$ $[M+H]^+$ 327,1703; found 327.1704.	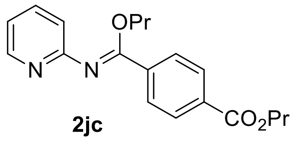 **2jc**
*(Z)-methyl N-pyridin-2-yl-1-naphthimidate (**2ka**):* Brown oil, 50 mg, yield 96%; Purified by flash column chromatography (SiO_2, elution system Hexane/EtOAc 2/1); ^{1}H NMR (500 MHz, $CDCl_3$); δ 8.21 (d, J = 4.0 Hz, 1H), 7.94 (d, J = 8.5 Hz, 1H), 7.76 (dd, J_1 = 8.0 Hz, J_2= 5.0 Hz, 2H), 7.51 (t, J = 7.5 Hz, 1H), 7.45 (t, J = 7.5 Hz, 1H), 7.37 (d, J = 7.0 Hz, 1H), 7.29 (t, J = 7.5 Hz,	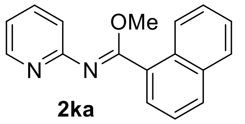 **2ka**

1H), 7.22 – 7.18 (m, 1H), 6.70 (t, *J* = 6.0 Hz, 1H), 6.42 (d, *J* = 8.0 Hz, 1H), 4.17 (s, 3H); ^{13}C{H} NMR (125 MHz, $CDCl_3$) δ 163.5, 160.4, 148.3, 137.3, 133.1, 130.4, 130.2, 129.8, 128.2, 127.1, 126.8, 126.2, 125.4, 124.6, 118.4, 116.0, 54.5; HRMS (ESI) *m/z*: calcd for $C_{17}H_{15}N_2O$ $[M+H]^+$ 263.1179; found 263.1186.	
*(Z)-ethyl N-pyridin-2-yl-1-naphthimidate (**2kb**):* Brown oil, 52 mg, yield 95%; Purified by flash column chromatography (SiO_2, elution system Hexane/EtOAc 2/1); ^{1}H NMR (500 MHz, $CDCl_3$) δ 8.20 (d shown as s, 1H), 7.98 (d, *J* = 8.5 Hz, 1H), 7.76 (t, *J* = 8.0 Hz, 2H), 7.51 (t, *J* = 7.5 Hz, 1H), 7.45 (t, *J* = 7.5 Hz, 1H), 7.38 (d, *J* = 7.0 Hz, 1H), 7.29 (t, *J* = 7.5 Hz, 1H), 7.18 (d, *J* = 6.5 Hz, 1H), 6.69 (d shown as s, 1H), 6.41 (d, *J* = 6.5 Hz, 1H), 4.66 (d, *J* = 6.5 Hz, 2H), 1.50 (t, *J* = 7.0 Hz, 3H); ^{13}C{H} NMR (125 MHz, $CDCl_3$) δ 162.9, 160.6, 148.3, 137.3, 133.2, 130.6, 130.2, 129.7, 128.2, 127.1, 126.8, 126.1, 125.3, 124.7, 118.3, 116.0, 63.2, 14.3; HRMS (ESI) *m/z*: calcd for $C_{18}H_{17}N_2O$ $[M+H]^+$ 277.1335; found 277.1342.	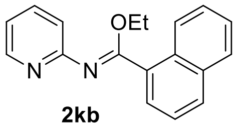 **2kb**
*(Z)-propyl N-pyridin-2-yl-1-naphthimidate (**2kc**):* Brown oil, 51 mg, yield 89%; Purified by flash column chromatography (SiO_2, elution system Hexane/EtOAc 7/1 to 2/1); ^{1}H NMR (500 MHz, $CDCl_3$) δ 8.20 (dd, J_1 = 5.0 Hz, J_2= 1.0 Hz, 1H), 7.98 (d, *J* = 8.5 Hz, 1H), 7.76 (t, *J* = 8.0 Hz, 2H), 7.51 (t, *J* = 7.5 Hz, 1H), 7.45 (t, *J* = 7.5 Hz, 1H), 7.38 (dd, J_1 = 7.0, J_2 = 1.0 Hz, 1H), 7.29 (dd, J_1 = 8.0 Hz, J_2 = 7.5 Hz, 1H), 7.19 (td, J_1 = 8.0 Hz, J_2= 2.0 Hz, 1H), 6.69 (ddd, J_1 = 7.0 Hz, J_2 = 5.0 Hz, J_3 = 1.0 Hz, 1H), 6.41 (d, *J* = 8.0 Hz, 1H), 4.55 (t, *J* = 6.5 Hz, 2H), 1.93 – 1.85 (m, 2H), 1.07 (t, *J* = 7.5 Hz, 3H); ^{13}C{H} NMR (125 MHz, $CDCl_3$) δ 163.2, 160.6, 148.3, 137.2, 133.2, 130.7, 130.3, 129.7, 128.2, 127.1, 126.7, 126.1, 125.4, 124.7, 118.2, 116.0, 69.0, 22.0, 10.7; HRMS (ESI) *m/z*: calcd for $C_{19}H_{19}N_2O$ $[M+H]^+$ 291.1492; found 291.1502.	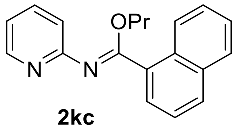 **2kc**
*(Z)-methyl N-pyridin-2-ylthiophene-2-carbimidate (**2la**):* Brown oil, 37 mg, yield 85%; Purified by flash column chromatography (SiO_2, elution system Hexane/EtOAc 7/1 to 2/1); ^{1}H NMR (500 MHz, $CDCl_3$) δ 8.42 (dd, J_1 = 5.0 Hz, J_2 = 1.5 Hz, 1H), 7.63 (td, J_1 = 7.5 Hz, J_2 = 2.0 Hz, 1H), 7.32 (d, *J* = 5.0 Hz, 1H), 7.03 (dd, J_1 = 7.5 Hz, J_2 = 5.0 Hz, 1H), 6.86 – 6.83 (m, 1H), 6.82 – 6.79 (m, 2H), 3.99 (s, 3H); ^{13}C{H} NMR (125 MHz, $CDCl_3$) δ 160.8, 154.2, 149.1, 138.2, 131.7, 131.6, 129.8, 126.9, 118.9, 116.1, 54.3; HRMS (ESI) *m/z*: calcd for $C_{11}H_{11}N_2OS$ $[M+H]^+$ 219.0587; found 219.0592.	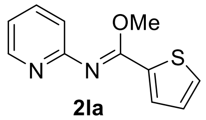 **2la**
*(Z)-ethyl N-pyridin-2-ylthiophene-2-carbimidate (**2lb**):* Brown oil, 44 mg, yield 95%; Purified by flash column chromatography (SiO_2, elution system Hexane/EtOAc 2/1); ^{1}H NMR (500 MHz, $CDCl_3$) δ 8.41 (ddd, J_1 = 5.0 Hz, J_2 = 1.5 Hz, J_3 = 1.0 Hz, 1H), 7.61 (ddd, J_1 = 8.0 Hz, J_2 = 7.5 Hz, J_3 = 2.0 Hz, 1H), 7.30 (dd, J_1 = 5.0 Hz, J_2 = 1.0 Hz, 1H), 7.00 (ddd, J_1 = 7.5 Hz, J_2 = 5.0 Hz, J_3 = 1.0 Hz, 1H), 6.84 – 6.81 (m, 2H), 6.79 – 6.77 (m, 1H), 4.43 (q, *J* = 7.0 Hz, 2H), 1.43 (t, *J* = 7.0 Hz, 3H); ^{13}C{H} NMR (125 MHz, $CDCl_3$) δ 160.9, 153.7, 149.1, 138.1, 132.0, 131.6, 129.7, 126.9, 118.7, 116.0, 62.8, 14.2; HRMS (ESI) *m/z*: calcd for $C_{12}H_{13}N_2OS$ $[M+H]^+$ 233.0743; found 233.0753.	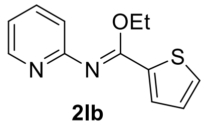 **2lb**

(Z)-propyl N-pyridin-2-ylthiophene-2-carbimidate (***2lc***): Brown oil, 48 mg, yield 98%; Purified by flash column chromatography (SiO_2, elution system Hexane/EtOAc 2/1); ^{1}H NMR (500 MHz, $CDCl_3$) δ 8.41 (dd, J_1 = 5.0 Hz, J_2 = 1.0 Hz, 1H), 7.61 (td, J_1 = 7.5 Hz, J_2= 2.0 Hz, 1H), 7.30 (dd, J_1 = 5.0, J_2 = 1.0 Hz, 1H), 7.01 (dd, J_1 = 7.0 Hz, J_2 = 5.5 Hz, 1H), 6.85 – 6.82 (m, 1H), 6.81 – 6.77 (m, 2H), 4.33 (t, *J* = 6.5 Hz, 2H), 1.88 – 1.80 (m, 2H), 1.06 (t, *J* = 7.5 Hz, 3H); ^{13}C{H} NMR (125 MHz, $CDCl_3$) δ 160.9, 153.8, 149.1, 138.1, 132.1, 131.5, 129.7, 126.9, 118.7, 116.1, 68.6, 22.0, 10.7; HRMS (ESI) *m/z*: calcd for $C_{13}H_{15}N_2OS$ $[M+H]^+$ 247.0900; found 247.0688.	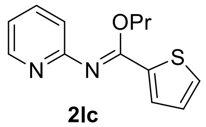 **2lc**
(Z)-methyl 4-methyl-N-(3-methylpyridin-2-yl)benzimidate (***2ma***): Brown oil, 16 mg, yield 33%; Purified by flash column chromatography (SiO_2, elution system Hexane/EtOAc 8/1 to 2/1); ^{1}H NMR (500 MHz, $CDCl_3$) δ 8.14 (d, *J* = 5.0 Hz, 1H), 7.36 (d, *J* = 7.5 Hz, 1H), 7.18 (d, *J* = 8.0 Hz, 2H), 6.99 (d, *J* = 8.0 Hz, 2H), 6.83 (dd, J_1 = 7.5 Hz, J_2 = 5.0 Hz, 1H), 4.02 (s, 3H), 2.26 (s, 3H), 2.02 (s, 3H); ^{13}C{H} NMR (125 MHz, $CDCl_3$) δ 159.9, 159.8, 146.1, 140.6, 138.4, 128.8, 128.7, 128.5, 123.7, 118.4, 54.2, 21.4, 17.6; HRMS (ESI) *m/z*: calcd for $C_{15}H_{17}N_2O$ $[M+H]^+$ 241.1335; found 241.1338.	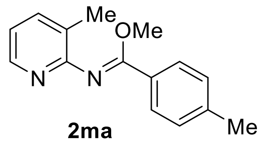 **2ma**
(Z)-methyl 4-methyl-N-(4-methylpyridin-2-yl)benzimidate (***2na***): Brown oil, 47 mg, yield 98%; Purified by flash column chromatography (SiO_2, elution system Hexane/EtOAc 2/1); ^{1}H NMR (500 MHz, $CDCl_3$) δ 8.15 (d, *J* = 5.0 Hz, 1H), 7.19 (d, *J* = 8.0 Hz, 2H), 6.99 (d, *J* = 8.0 Hz, 2H), 6.69 (dd, J_1 = 5.0 Hz, J_2 = 0.5 Hz, 1H), 6.41 (s, 1H), 3.96 (s, 3H), 2.25 (s, 3H), 2.17 (s, 3H); ^{13}C{H} NMR (125 MHz, $CDCl_3$) δ 161.2, 160.8, 148.8, 148.3, 140.5, 129.1, 128.6, 128.3, 119.4, 116.6, 54.2, 21.3, 20.9; HRMS (ESI) *m/z*: calcd for $C_{15}H_{17}N_2O$ $[M+H]^+$ 241.1335; found 241.1339.	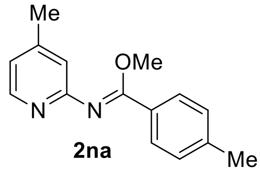 **2na**
(Z)-methyl 4-methyl-N-(5-methylpyridin-2-yl)benzimidate (***2oa***): Brown oil, 44 mg, yield 92%; Purified by flash column chromatography (SiO_2, elution system Hexane/EtOAc 2/1); ^{1}H NMR (500 MHz, $CDCl_3$) δ 8.14 (s, 1H), 7.25 (dd, J_1 = 8.0 Hz, J_2 = 2.0 Hz, 1H), 7.17 (d, *J* = 8.0 Hz, 2H), 6.99 (d, *J* = 8.0 Hz, 2H), 6.44 (d, *J* = 8.0 Hz, 1H), 3.97 (s, 3H), 2.25 (s, 3H), 2.20 (s, 3H); ^{13}C{H} NMR (125 MHz, $CDCl_3$) δ 161.3, 158.9, 148.5, 140.4, 138.5, 129.1, 128.7, 128.4, 127.2, 115.7, 54.2, 21.3, 17.7; HRMS (ESI) *m/z*: calcd for $C_{15}H_{17}N_2O$ $[M+H]^+$ 241.1335; found 241.1339.	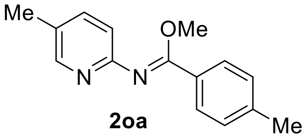 **2oa**
(Z)-methyl 4-methyl-N-(6-methylpyridin-2-yl)benzimidate (***2pa***): Yellow oil, 34 mg, yield 71%; Purified by flash column chromatography (SiO_2, elution system Hexane/EtOAc 7/1 to 2/1); ^{1}H NMR (500 MHz, $CDCl_3$) δ 7.32 (t, *J* = 7.5 Hz, 1H), 7.19 (d, *J* = 8.0 Hz, 2H), 7.01 (d, *J* = 8.0 Hz, 2H), 6.75 (d, *J* = 7.5 Hz, 1H), 6.26 (d, *J* = 8.0 Hz, 1H), 4.00 (s, 3H), 2.49 (s, 3H), 2.27 (s, 3H); ^{13}C{H} NMR (125 MHz, $CDCl_3$) ^{13}C{H} NMR (125 MHz, $CDCl_3$) δ 161.3, 160.6, 157.5, 140.6, 138.1, 129.1, 128.7, 128.2, 117.4, 113.0, 54.4, 24.3, 21.4; HRMS (ESI) *m/z*: calcd for $C_{15}H_{17}N_2O$ $[M+H]^+$ 241.1335; found 241.1339.	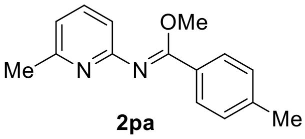 **2pa**

*(Z)-methyl N-(3-chloropyridin-2-yl)-4-methylbenzimidate (**2qa**):* Light yellow oil, 23 mg, yield 44%; Purified by flash column chromatography (SiO_2, elution system Hexane/EtOAc 8/1 to 1/1); ^1H NMR (500 MHz, $CDCl_3$) δ 8.20 (d, *J* = 5.0 Hz, 1H), 7.57 (d, *J* = 8.0 Hz, 1H), 7.23 (d, *J* = 8.0 Hz, 2H), 7.02 (d, *J* = 8.0 Hz, 2H), 6.86 (dd, J_1 = 8.0 Hz, J_2 = 5.0 Hz, 1H), 4.05 (s, 3H), 2.28 (s, 3H); $^{13}C\{H\}$ NMR (125 MHz, $CDCl_3$) δ 162.1, 158.0, 146.7, 140.9, 137.9, 128.8, 128.7, 128.5, 122.5, 119.0, 54.7, 21.4; HRMS (ESI) *m/z*: calcd for $C_{14}H_{14}ClN_2O$ $[M+H]^+$ 261.0789; found 261.0787.	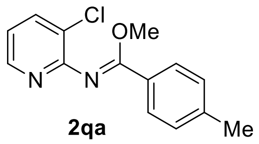 2qa
*(Z)-methyl N-(5-chloropyridin-2-yl)-4-methylbenzimidate (**2ra**):* Brown oil, 46 mg, yield 89%; Purified by flash column chromatography (SiO_2, elution system Hexane/EtOAc 7/1 to 2/1); ^1H NMR (500 MHz, $CDCl_3$) δ 8.27 (s, 1H), 7.41 (dd, J_1 = 8.5 Hz, J_2 = 1.5 Hz, 1H), 7.16 (d, *J* = 8.0 Hz, 2H), 7.02 (d, *J* = 8.0 Hz, 2H), 6.50 (d, *J* = 8.5 Hz, 1H), 3.98 (s, 3H), 2.27 (s, 3H); $^{13}C\{H\}$ NMR (125 MHz, $CDCl_3$) δ 162.0, 159.7, 147.2, 140.1, 137.5, 129.0, 128.9, 127.9, 125.7, 117.2, 54.5, 21.4; HRMS (ESI) *m/z*: calcd for $C_{14}H_{14}ClN_2O$ $[M+H]^+$ 261.0789; found 261.0792.	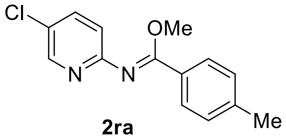 2ra
*(Z)-methyl N-(5-bromopyridin-2-yl)-4-methylbenzimidate (**2sa**):* Brown oil, 60 mg, yield 98%; Purified by flash column chromatography (SiO_2, elution system Hexane/EtOAc 2/1); ^1H NMR (500 MHz, $CDCl_3$) δ 8.36 (s, 1H), 7.53 (dd, J_1 = 8.5 Hz, J_2 = 2.5 Hz, 1H), 7.17 (d, *J* = 7.5 Hz, 2H), 7.03 (d, *J* = 7.5 Hz, 2H), 6.45 (dd, J_1 = 8.5, J_2 = 1.0 Hz, 1H), 3.98 (s, 3H), 2.28 (s, 3H); $^{13}C\{H\}$ NMR (125 MHz, $CDCl_3$) δ 161.9, 160.1, 149.6, 140.9, 140.1, 129.1, 128.9, 127.9, 117.8, 113.8, 54.5, 21.4; HRMS (ESI) *m/z*: calcd for $C_{14}H_{14}BrN_2O$ $[M+H]^+$ 305.0284 and 307.0264; found 305.0438 and 307.0445.	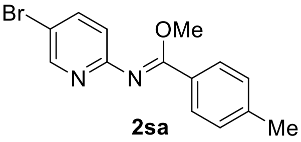 2sa

^{1}H and ^{13}C{H} NMR data for *N,N*-heterocycles 7g, 8g

*2-(4-chlorophenyl)-1,4,5,6-tetrahydropyrimidine (**7g**):*[S6] White solid, 13 mg, yield 35%; mp (144-145 °C); ^{1}H NMR (500 MHz, $CDCl_3$) δ 7.61 (d, *J* = 8.5 Hz, 2H), 7.31 (d, *J* = 8.5 Hz, 2H), 5.05 (brs, 1H), 3.46 (t, *J* = 5.5 Hz 4H), 1.86 (quin, *J* = 5.5 Hz, 2H); ^{13}C{H} NMR (125 MHz, $CDCl_3$) δ 155.0, 136.6, 133.8, 128.7, 128.0, 41.7, 20.2.	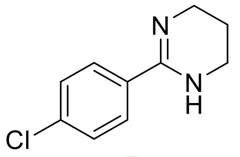 **7g**
*2-(4-chlorophenyl)-4,5-dihydro-1H-imidazole (**8g**):*[S7] White solid, 20 mg, yield 55%; mp (186-187 °C); ^{1}H NMR (500 MHz, CD_3OD) δ 7.76 (d, *J* = 8.5 Hz, 2H), 7.45 (d, *J* = 8.5 Hz, 2H), 3.76 (s, 4H); ^{13}C{H} NMR (125 MHz, CD_3OD): δ 172.1, 144.4, 138.8, 138.3, 137.7, 59.1.	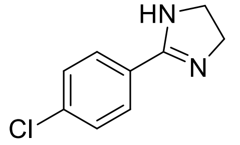 **8g**

The spectroscopic data are in agreement with the literature reported data in ref [S6 and S7]

References

S1. Wang, R.; Wang, S.; Li, D.; Ye, F.; Leng, Y.; Wu, Y.; Chang, J.; Wu, Y. Radical Ring Opening Reaction of Pyridine Fused Heterocycles with IBA-N3 Catalyzed by Tetra-n-Butylammonium Iodide. *Tetrahedron* **2019**, *75*, 2298–2305. https://doi.org/10.1016/j.tet.2019.02.061

S2. Chen, Z.; Liang, P.; Zheng, J.; Zhou, Z.; Wen, X.; Liu, T.; Ye, M. Cyanide-Free Ce(III)-Catalyzed Highly Efficient Synthesis of α-Iminonitriles from 2-Aminopyridines and Nitroalkenes via Intermolecular Dehydration Reaction. *ACS Omega* **2018**, *3*, 12520–12529. https://doi.org/10.1021/acsomega.8b02214

S3. Kalbandhe, A.H.; Kavale, A.C.; Karade, N.N. Ring-Opening Reaction of Imidazo[1,2-a]Pyridines Using (Diacetoxyiodo)Benzene and NaN3: The Synthesis of α-Iminonitriles. *Eur. J. Org. Chem.* **2017**, *2017*, 1318–1322. https://doi.org/10.1002/ejoc.201601480

S4. Birch, D.J.; Guildford, A.J.; Tometzki, M.A.; Turner, R.W. Reaction of 3-Nitroso-2-Phenylimidazo[1,2-a]Pyridine with Triethyl Phosphite. A Revised Structure for the Product. *J. Org. Chem.* **1982**, *47*, 3547–3548. https://doi.org/https://doi.org/10.1021/jo00139a034

S5. Wang, G.; Wei, C.; Hong, X.; Fu, Z.; Huang, W. Sodium Pyruvate as a Peroxide Scavenger in Aerobic Oxidation under Carbene Catalysis. *Green Chem.* **2020**, *22*, 6819–6826. https://doi.org/10.1039/d0gc02555k

S6. Nenajdenko G.V.; Muzalevskiy M.V.; Shastin A.V.; Balenkova S.E.; Kondrashov E.V.; Ushakov I.A.; Rulev A.Y. Fragmentation of Trifluoromethylated Alkenes and Acetylenes by N,N,-Binucleophiles. Synthesis of Imidazolines or Imidazolidines (Oxazolidines) Controlled by Substituent. *J. Org. Chem.* **2010**, 5679-5688 https://doi.org/10.1021/jo101107t

S7. Hegedus A.; Vigh I.; Hell Z. Zeolite-catalyzed simple synthesis of different heterocyclic rings part 2, *Heteroat. Chem.* **2004**, *15*, 428 https://doi.org/10.1002/hc.20036

Copies of ^{1}H and ^{13}C{H} NMR spectra of α-iminotriles 1a-1h, 1j-1s

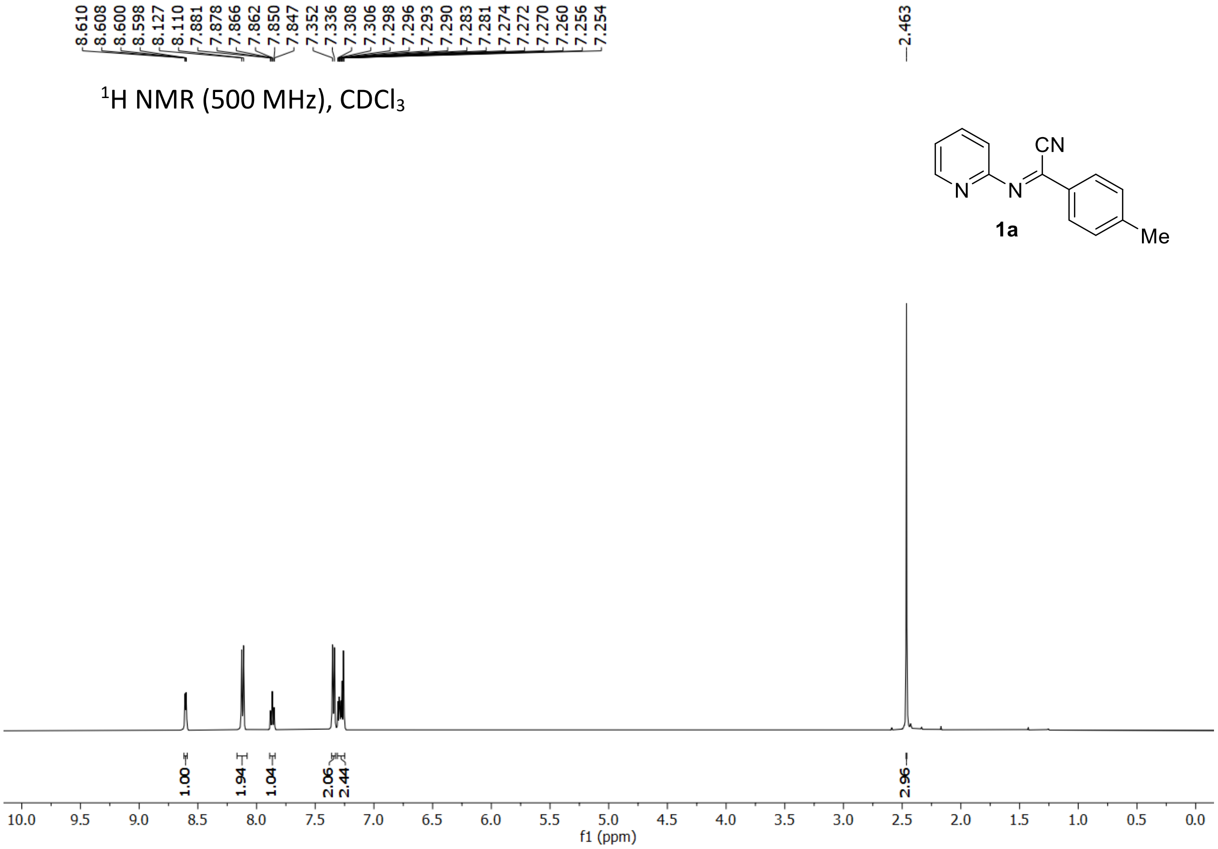

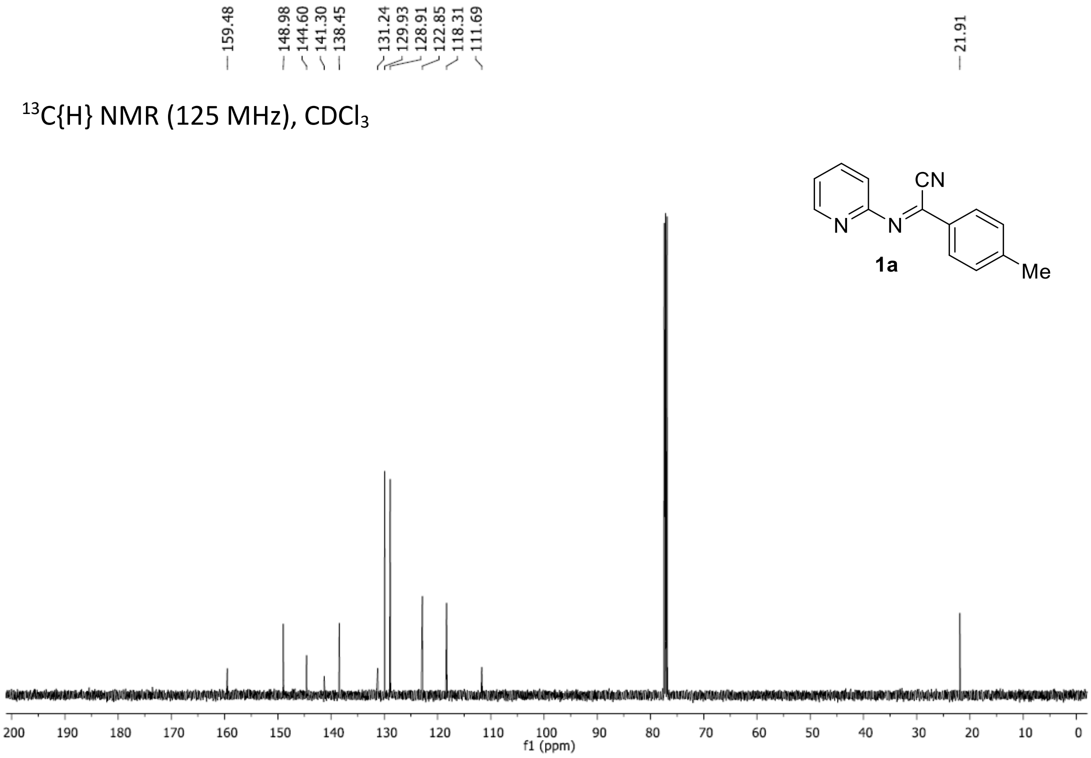

¹H NMR (500 MHz), $CDCl_3$

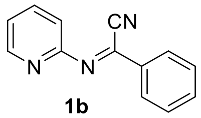

1b

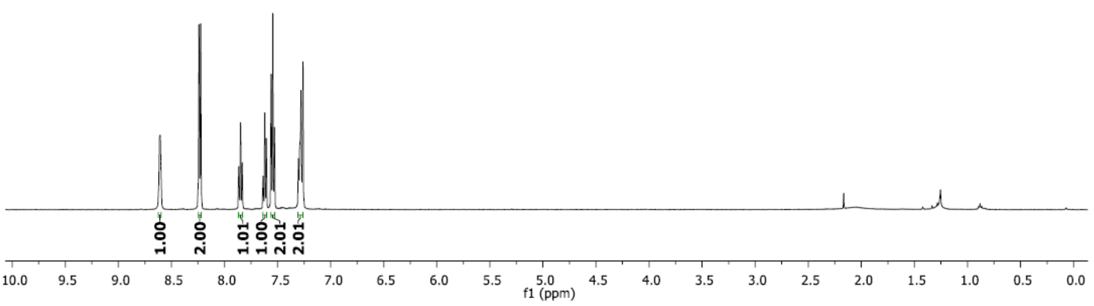

$^{13}C\{H\}$ NMR (125 MHz), $CDCl_3$

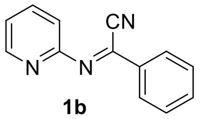

1b

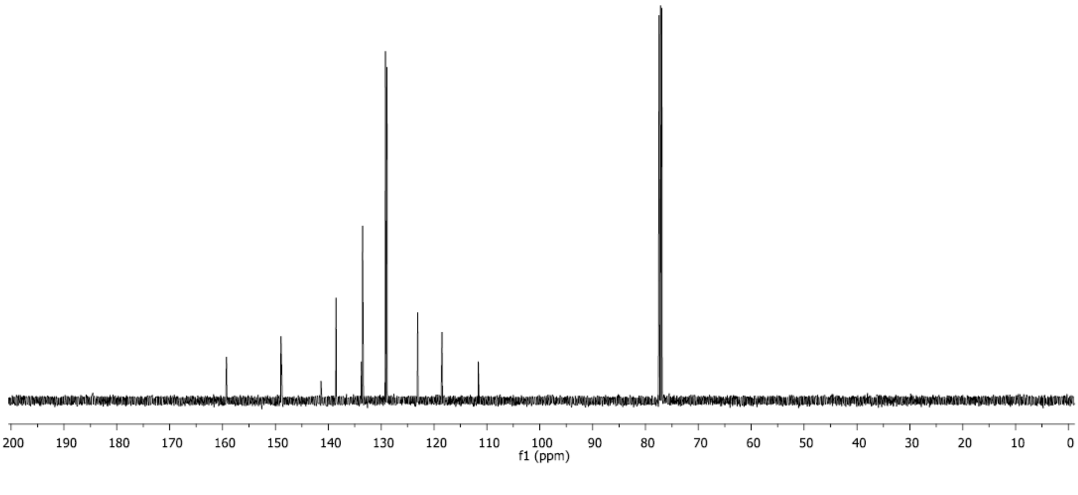

^{1}H NMR (500 MHz), $CDCl_3$

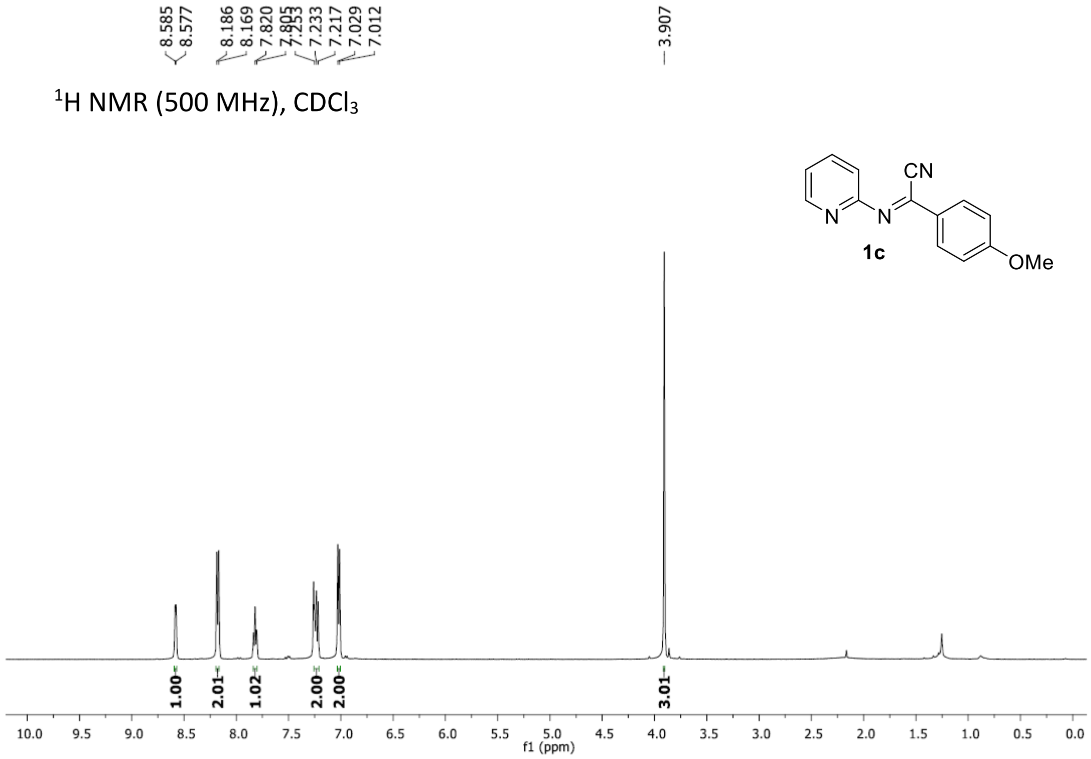

$^{13}C\{H\}$ NMR (125 MHz), $CDCl_3$

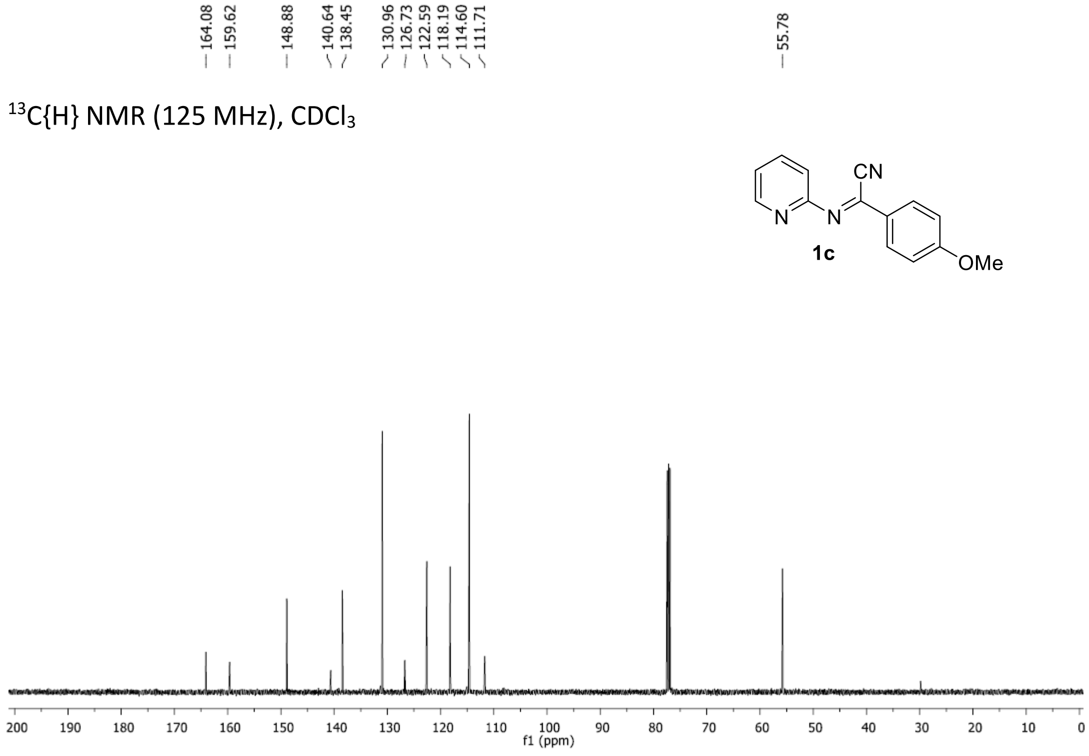

^{1}H NMR (500 MHz), $CDCl_3$

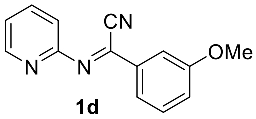

1d

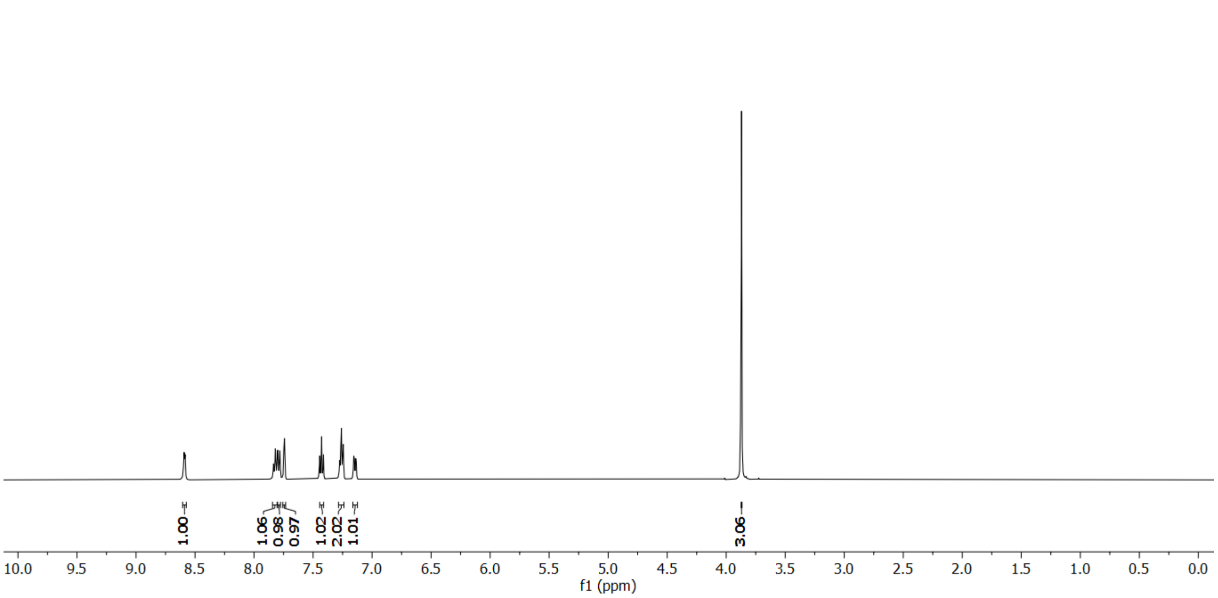

^{13}C{H} NMR (125 MHz), $CDCl_3$

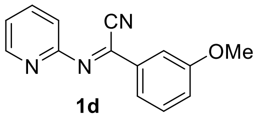

1d

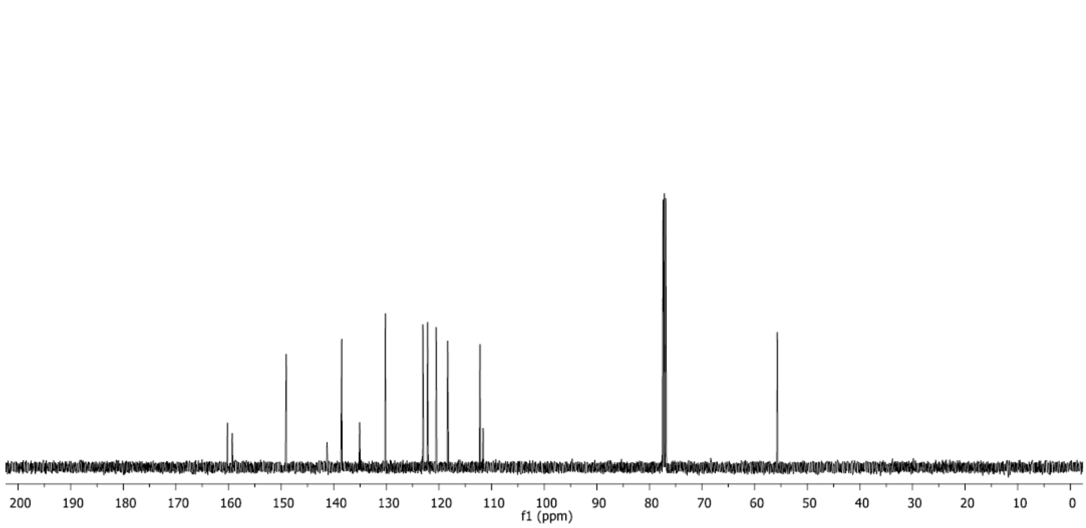

^{1}H NMR (500 MHz), $CDCl_3$

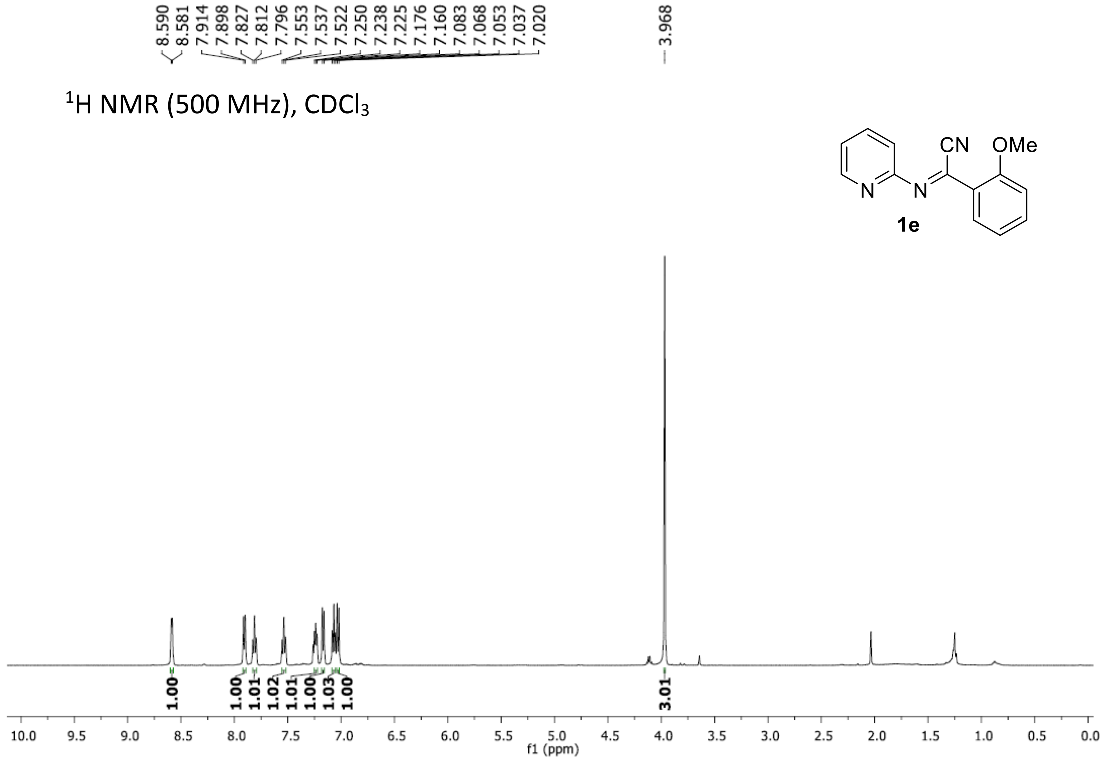

$^{13}C\{H\}$ NMR (125 MHz), $CDCl_3$

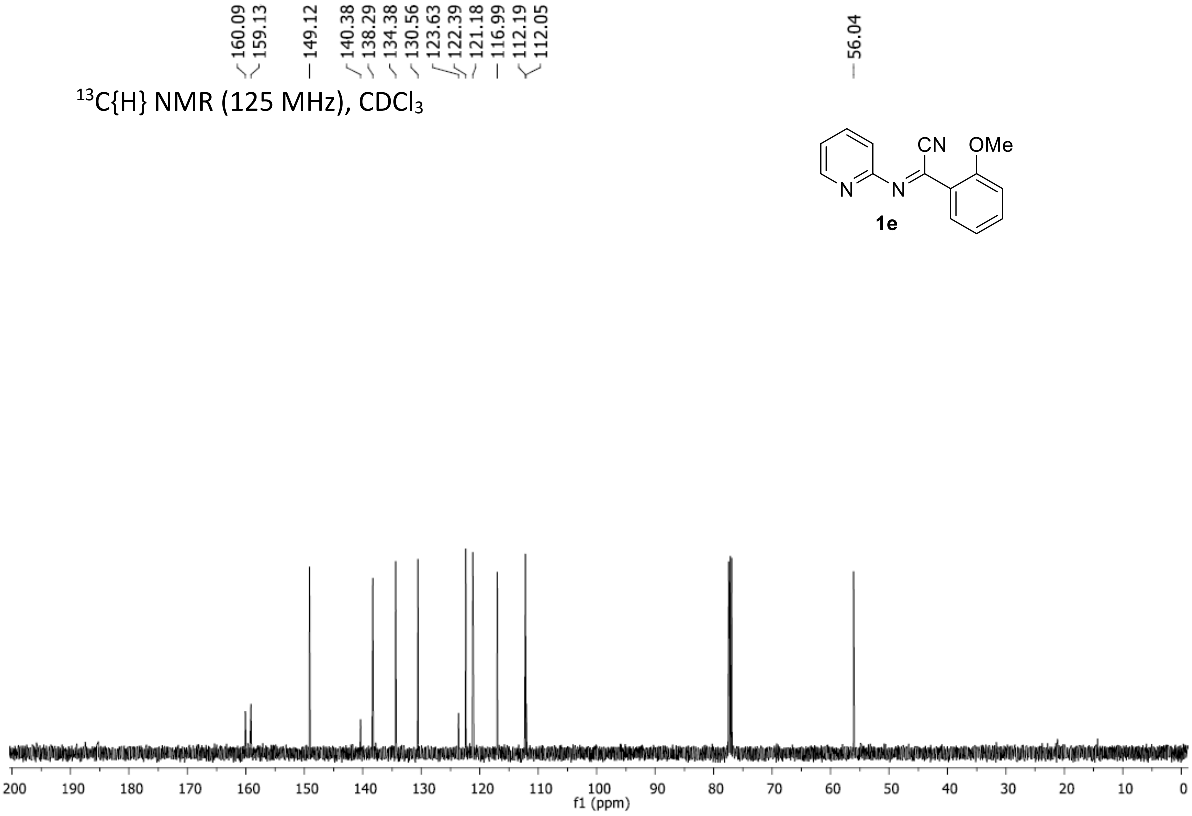

^{1}H NMR (500 MHz), $CDCl_3$

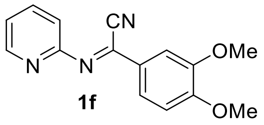

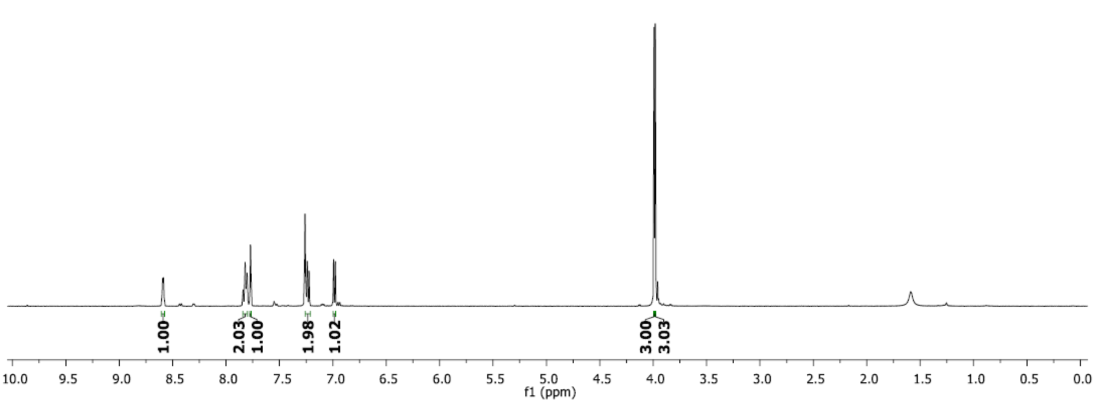

^{13}C{H} NMR (125 MHz), $CDCl_3$

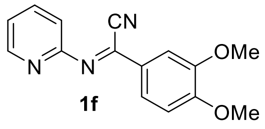

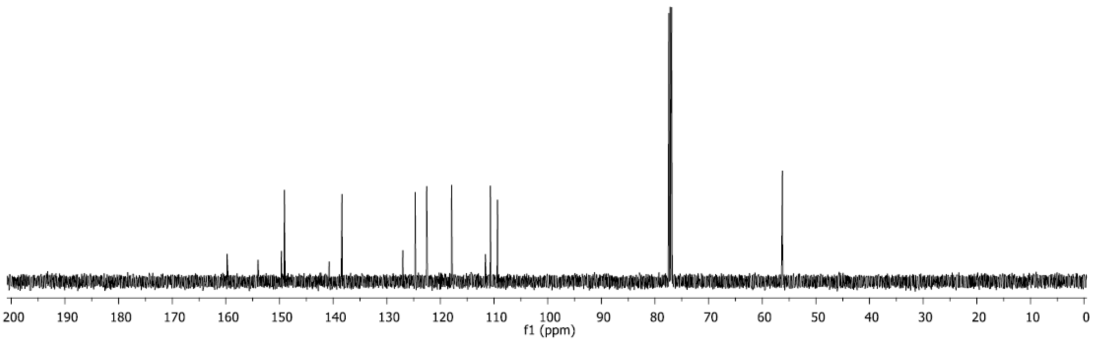

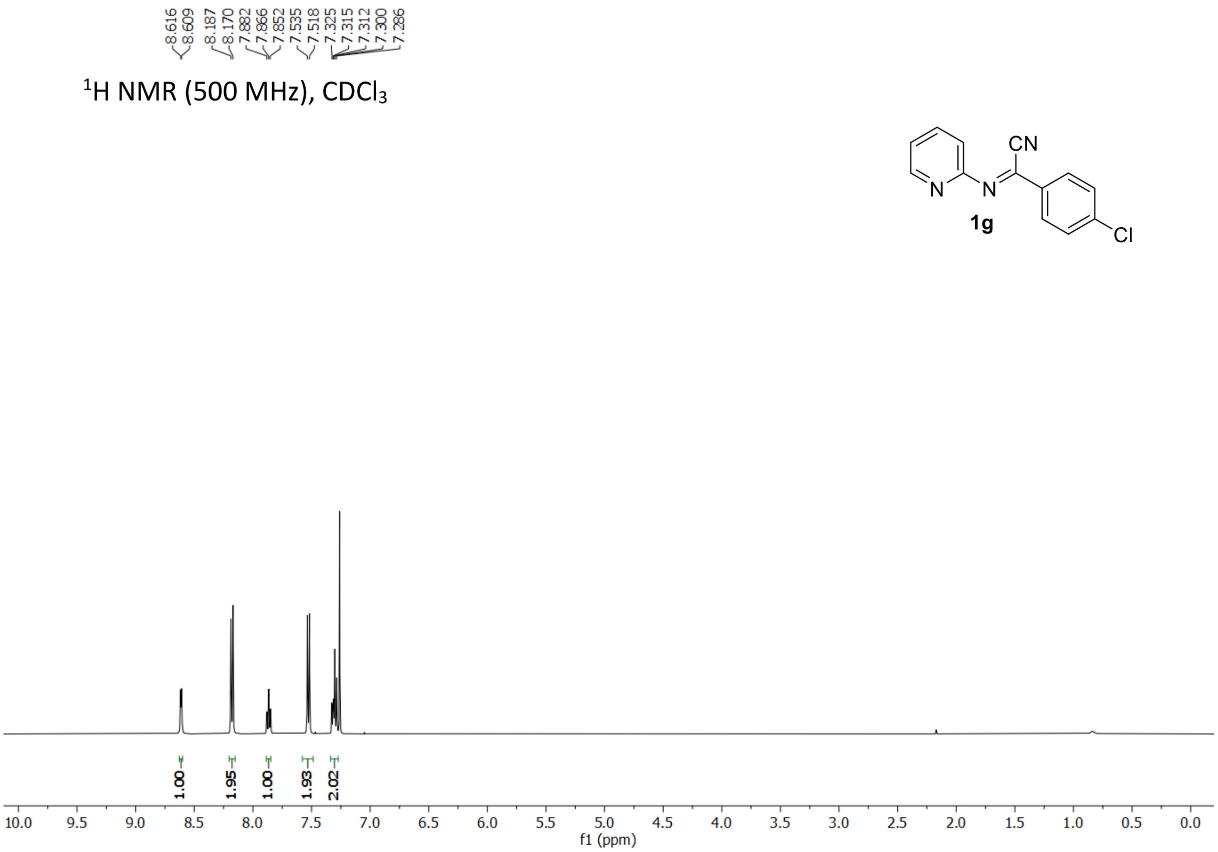
8.616
8.609
8.187
8.170
7.882
7.866
7.852
7.535
7.518
7.325
7.315
7.312
7.300
7.286
¹H NMR (500 MHz), CDCl₃
CN
N
N
1g
Cl
1.00
1.95
1.00
1.93
2.02
f1 (ppm)

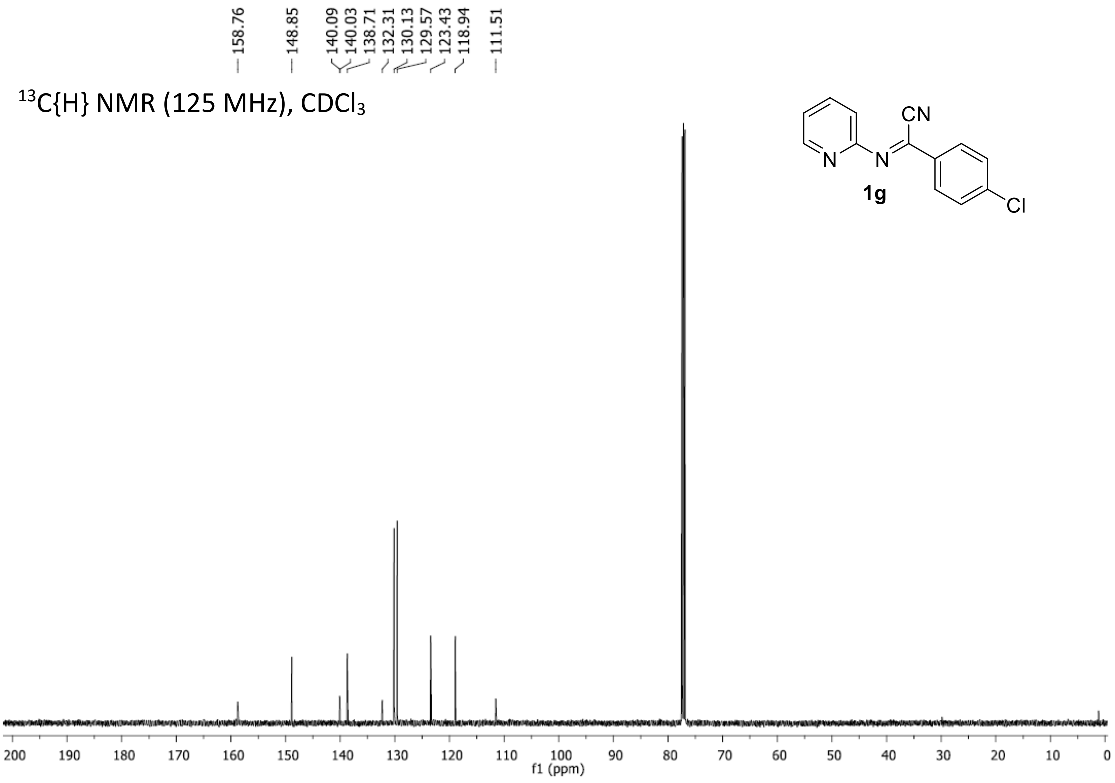
158.76
148.85
140.09
140.03
138.71
132.31
130.13
129.57
123.43
118.94
111.51
¹³C{H} NMR (125 MHz), CDCl₃
CN
N
N
1g
Cl
f1 (ppm)

^{1}H NMR (500 MHz), $CDCl_3$

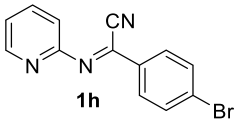

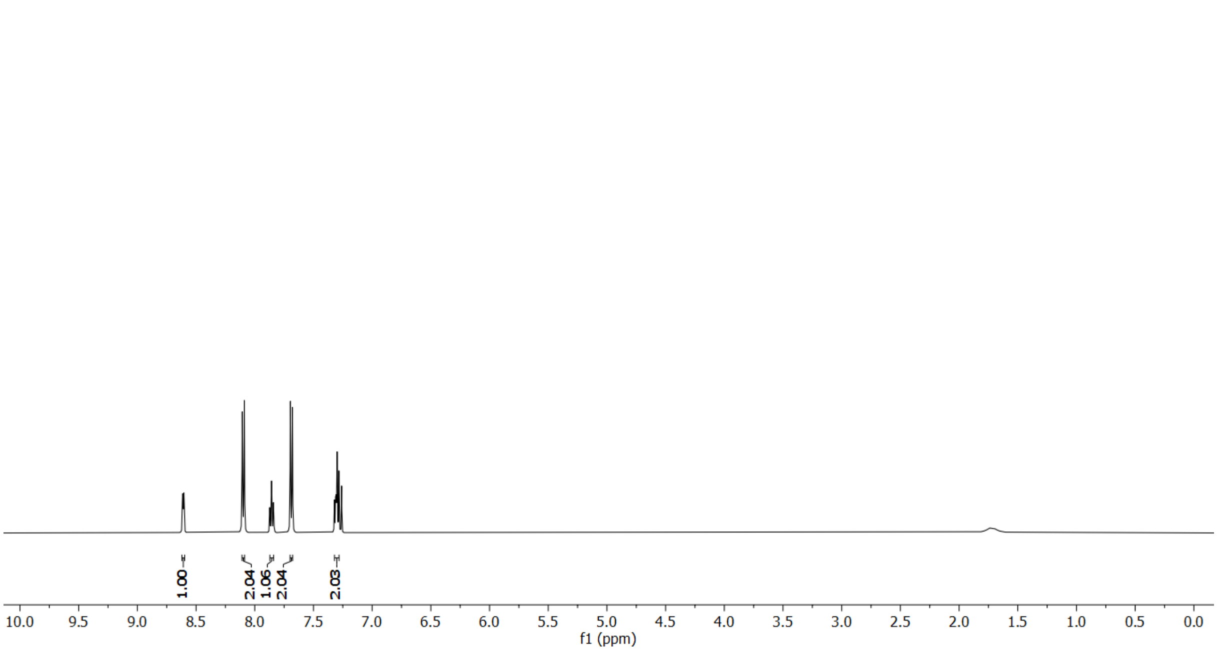

$^{13}C\{H\}$ NMR (125 MHz), $CDCl_3$

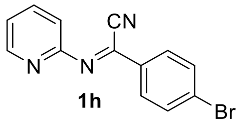

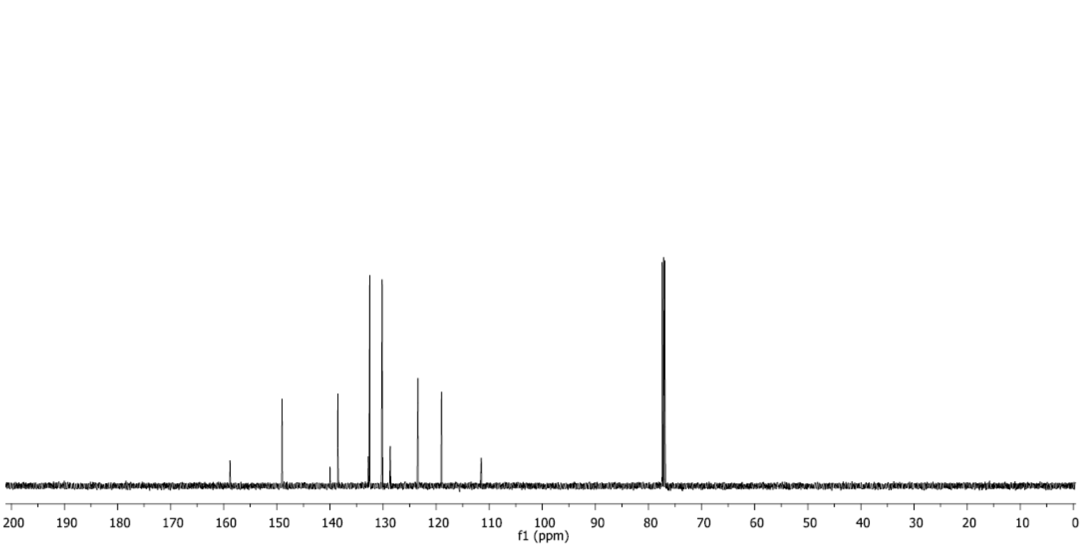

^{1}H NMR (500 MHz), $CDCl_3$

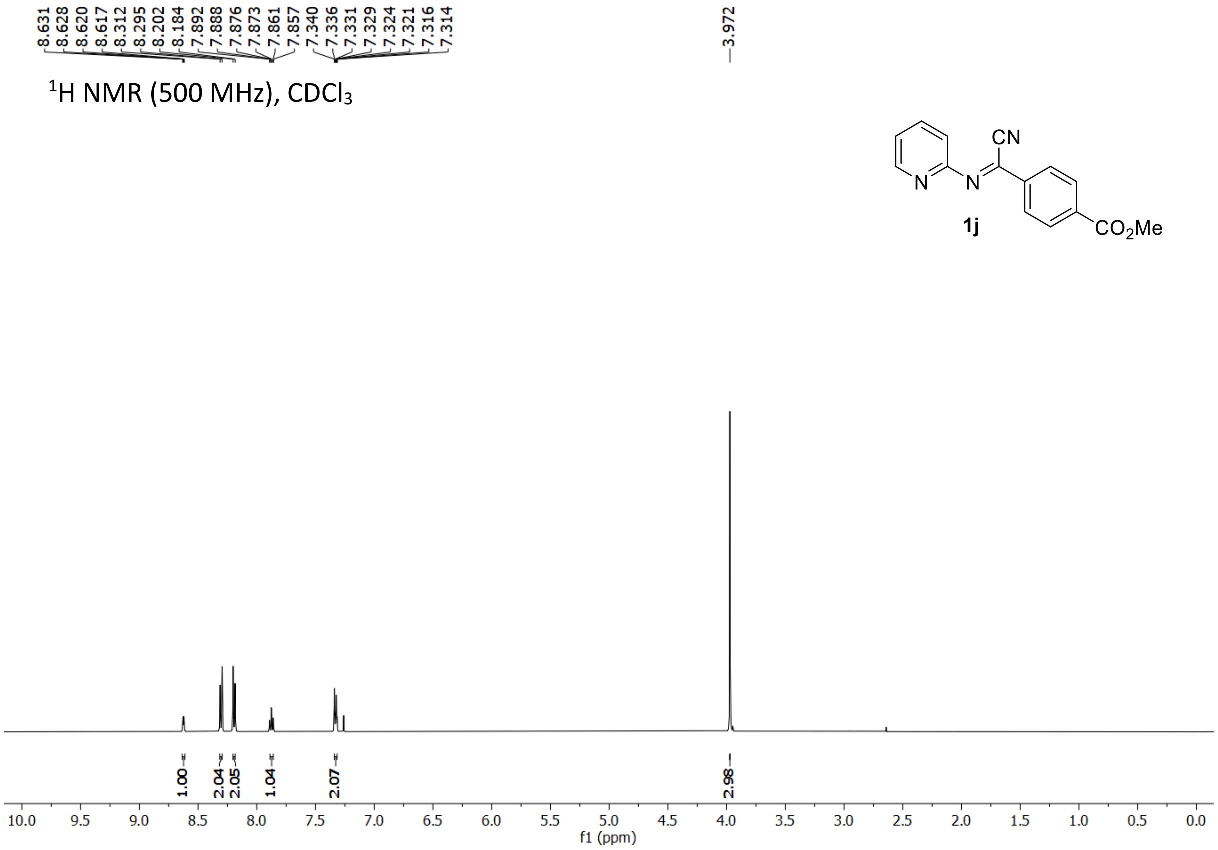

1j

^{13}C{H} NMR (125 MHz), $CDCl_3$

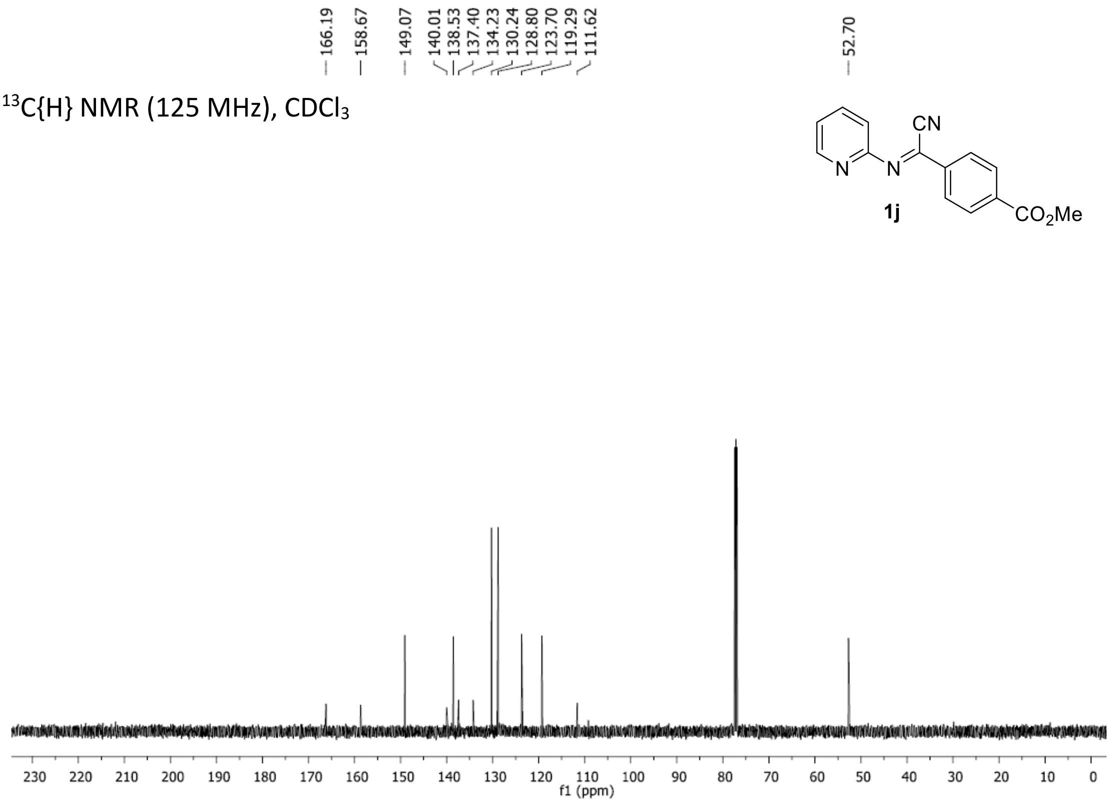

1j

^{1}H NMR (500 MHz), $CDCl_3$

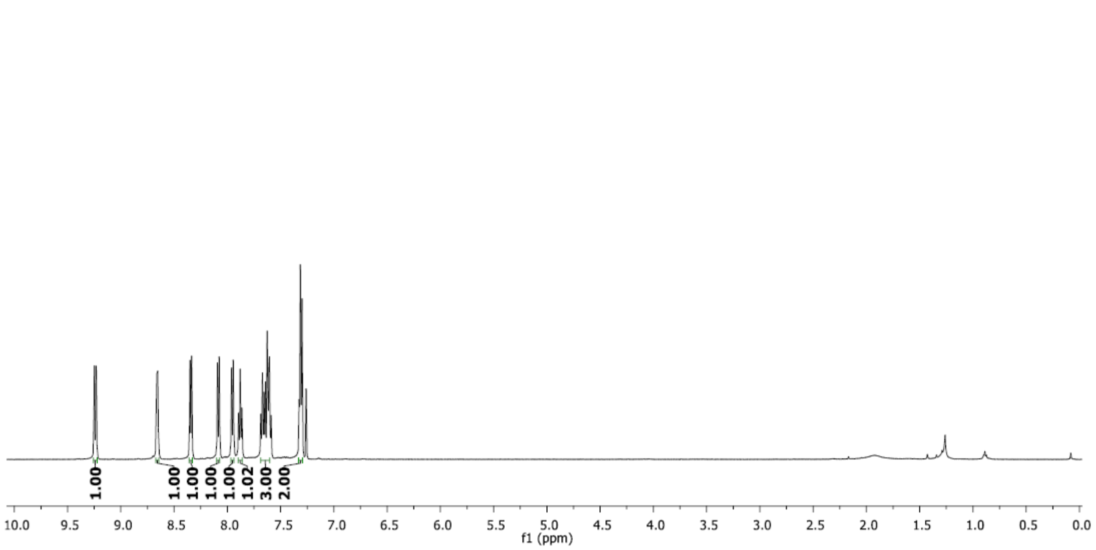

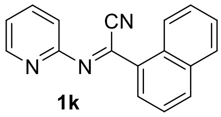

1k

^{13}C{H} NMR (125 MHz), $CDCl_3$

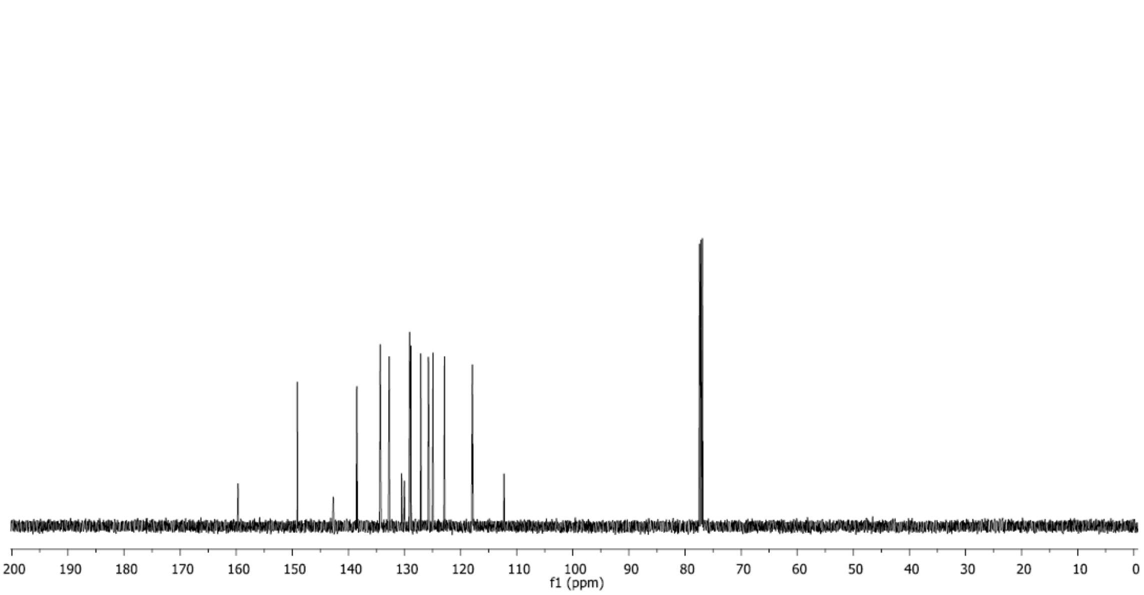

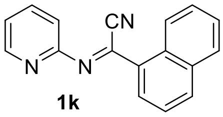

1k

^{1}H NMR (500 MHz), $CDCl_3$

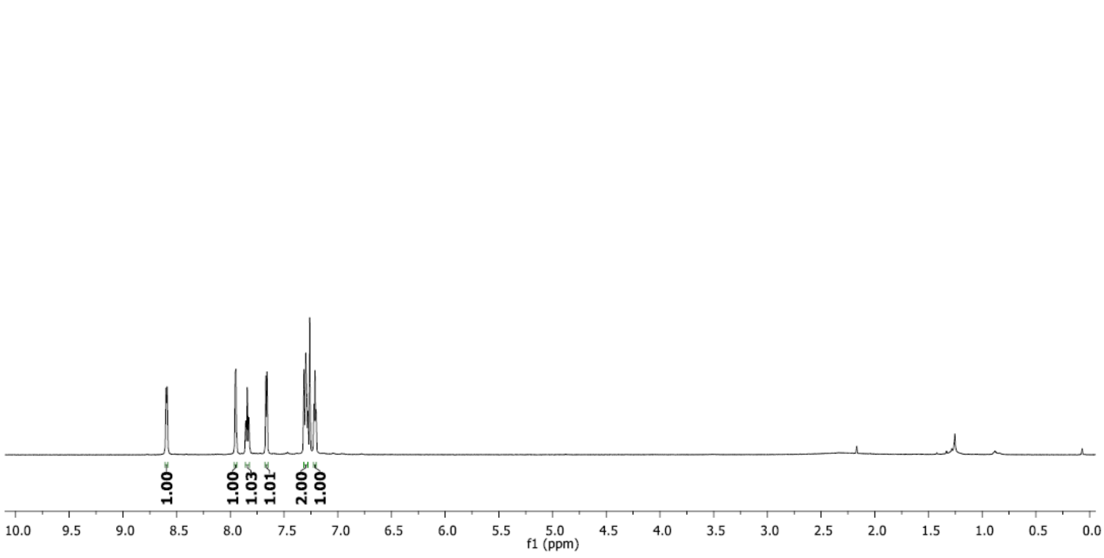

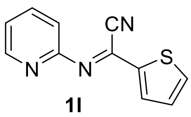

1l

$^{13}C\{H\}$ NMR (125 MHz), $CDCl_3$

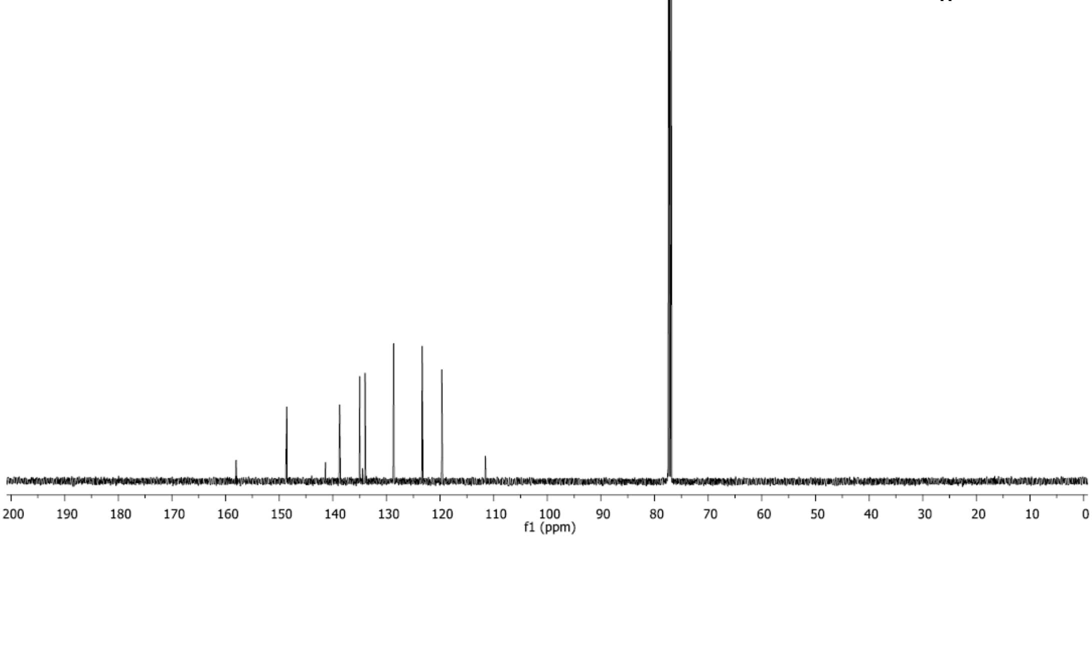

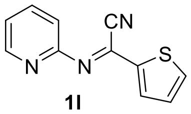

1l

^{1}H NMR (500 MHz), $CDCl_3$

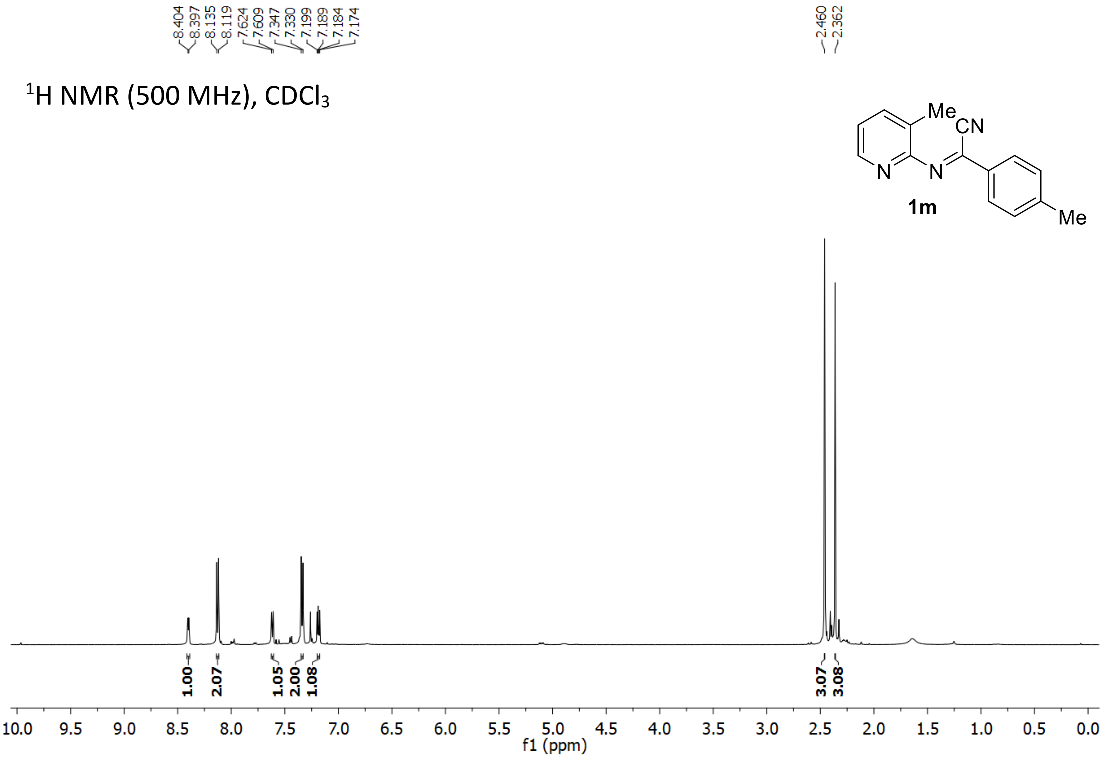

$^{13}C\{H\}$ NMR (125 MHz), $CDCl_3$

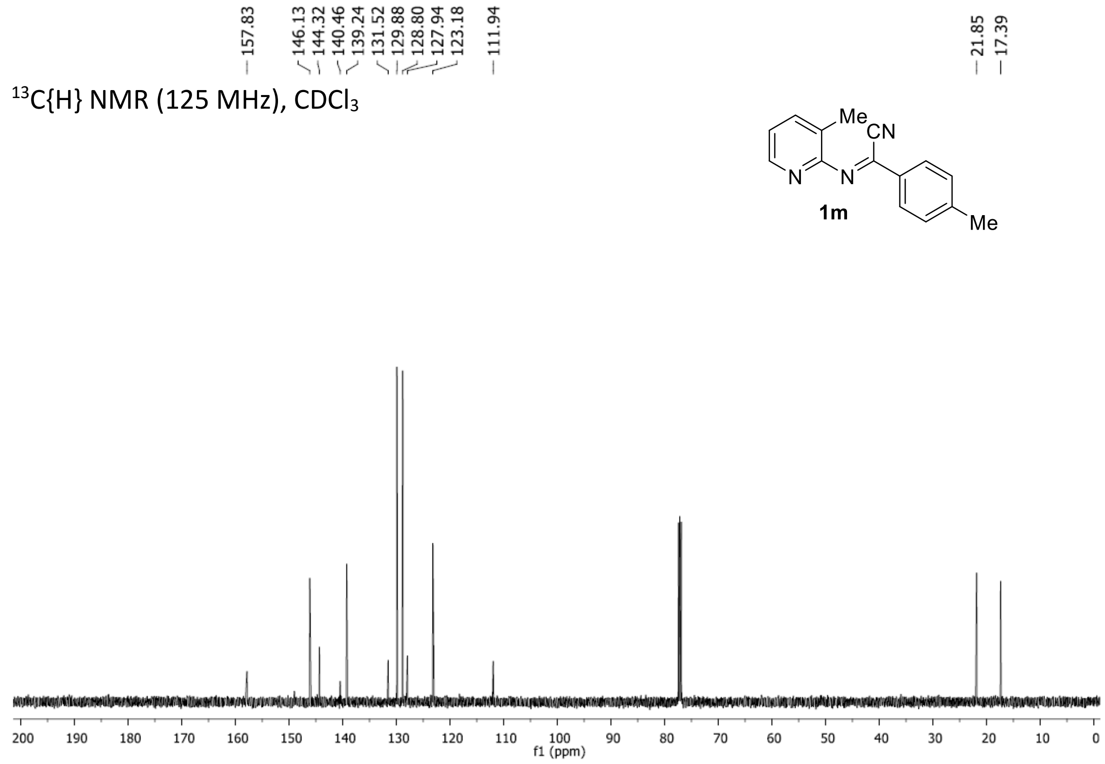

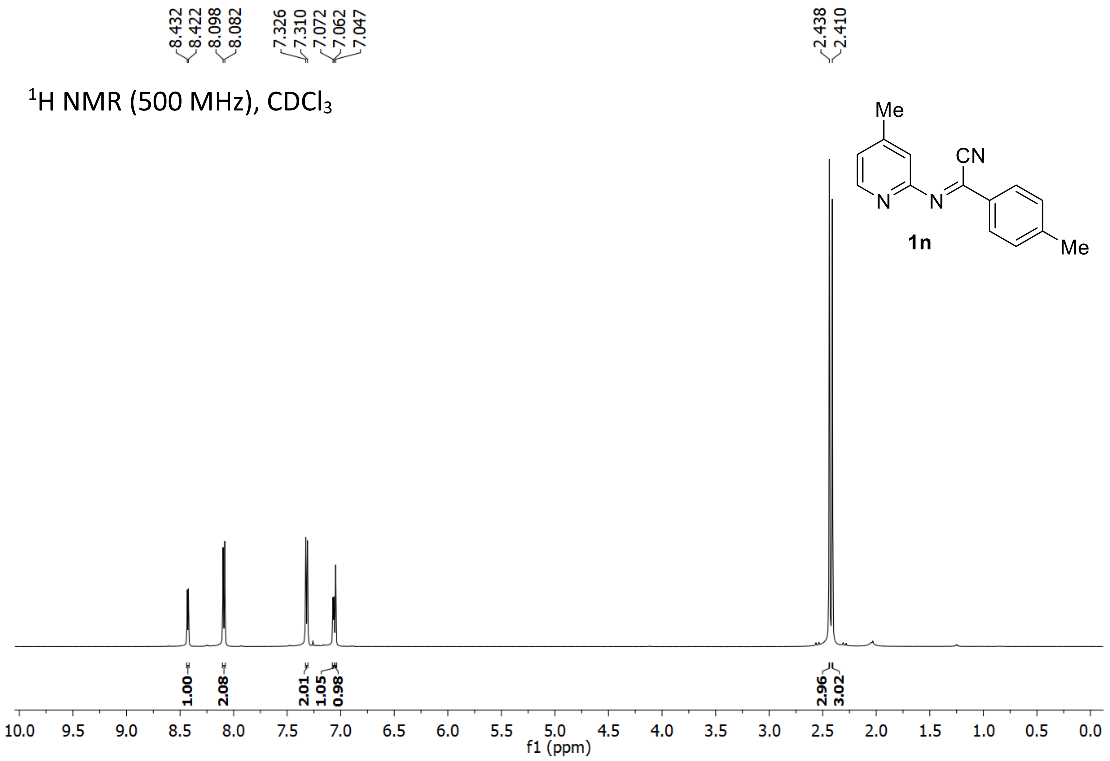
8.432
8.422
8.098
8.082
7.326
7.310
7.072
7.062
7.047
2.438
2.410
1H NMR (500 MHz), CDCl3
Me
CN
N
N
1n
Me
1.00
2.08
2.01
1.05
0.98
2.96
3.02
10.0 9.5 9.0 8.5 8.0 7.5 7.0 6.5 6.0 5.5 5.0 4.5 4.0 3.5 3.0 2.5 2.0 1.5 1.0 0.5 0.0
f1 (ppm)

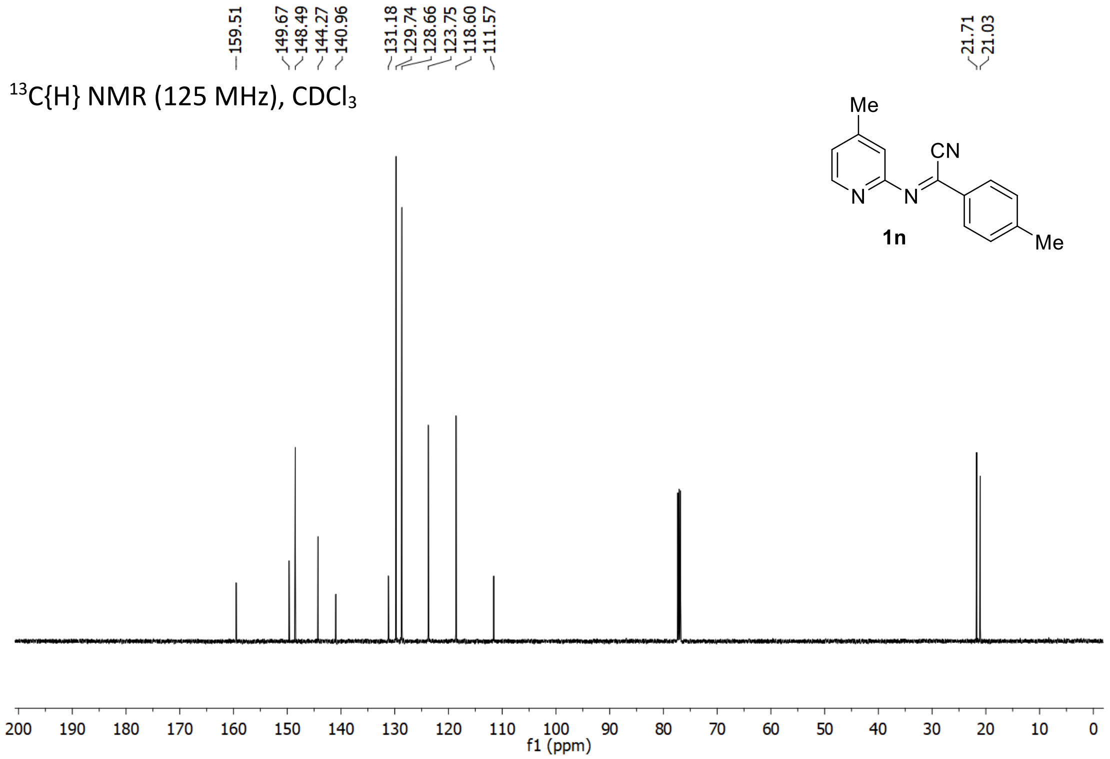
159.51
149.67
148.49
144.27
140.96
131.18
129.74
128.66
123.75
118.60
111.57
21.71
21.03
13C{H} NMR (125 MHz), CDCl3
Me
CN
N
N
1n
Me
200 190 180 170 160 150 140 130 120 110 100 90 80 70 60 50 40 30 20 10 0
f1 (ppm)

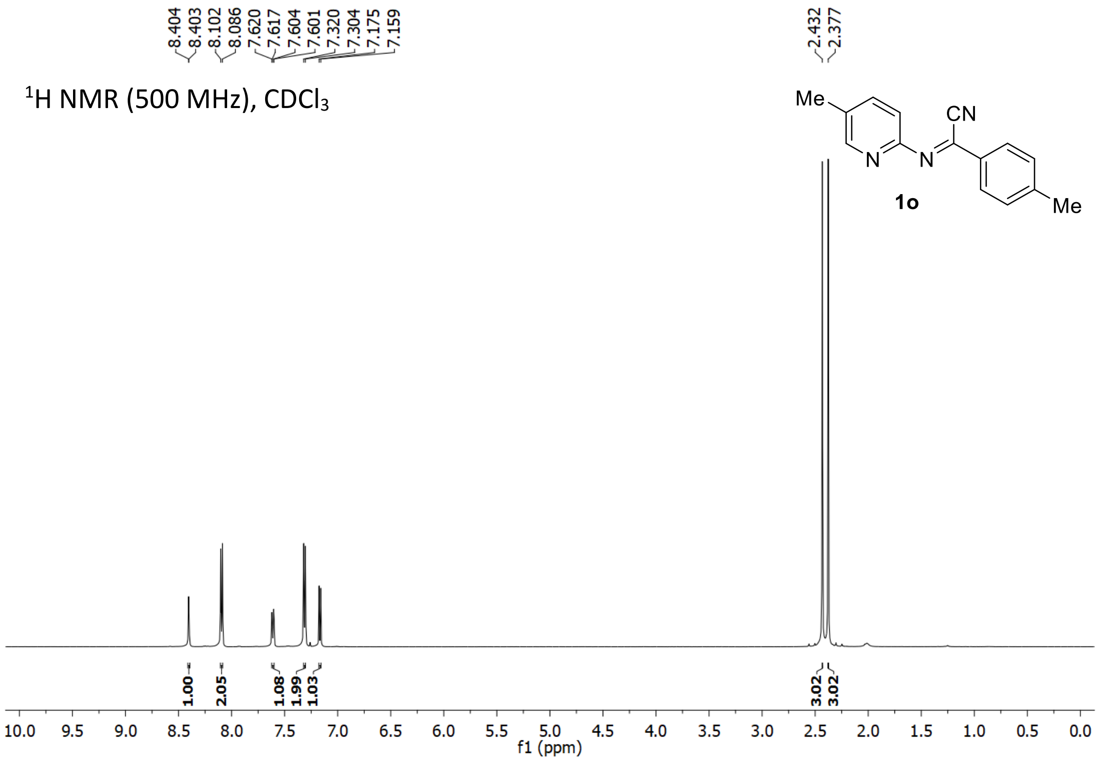

8.404
8.403
8.102
8.086
7.620
7.617
7.604
7.601
7.320
7.304
7.175
7.159
2.432
2.377
¹H NMR (500 MHz), CDCl₃
Me
CN
N
N
1o
Me
1.00
2.05
1.08
1.99
1.03
3.02
3.02
f1 (ppm)

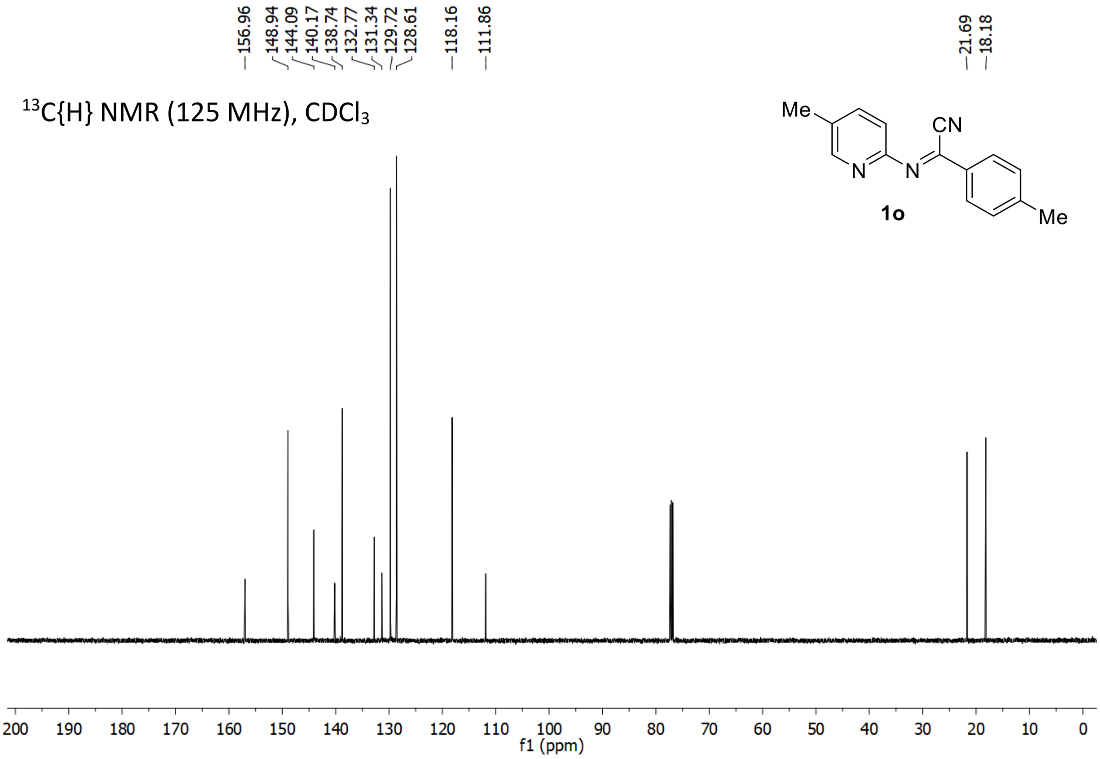

156.96
148.94
144.09
140.17
138.74
132.77
131.34
129.72
128.61
118.16
111.86
21.69
18.18
¹³C{H} NMR (125 MHz), CDCl₃
Me
CN
N
N
1o
Me
f1 (ppm)

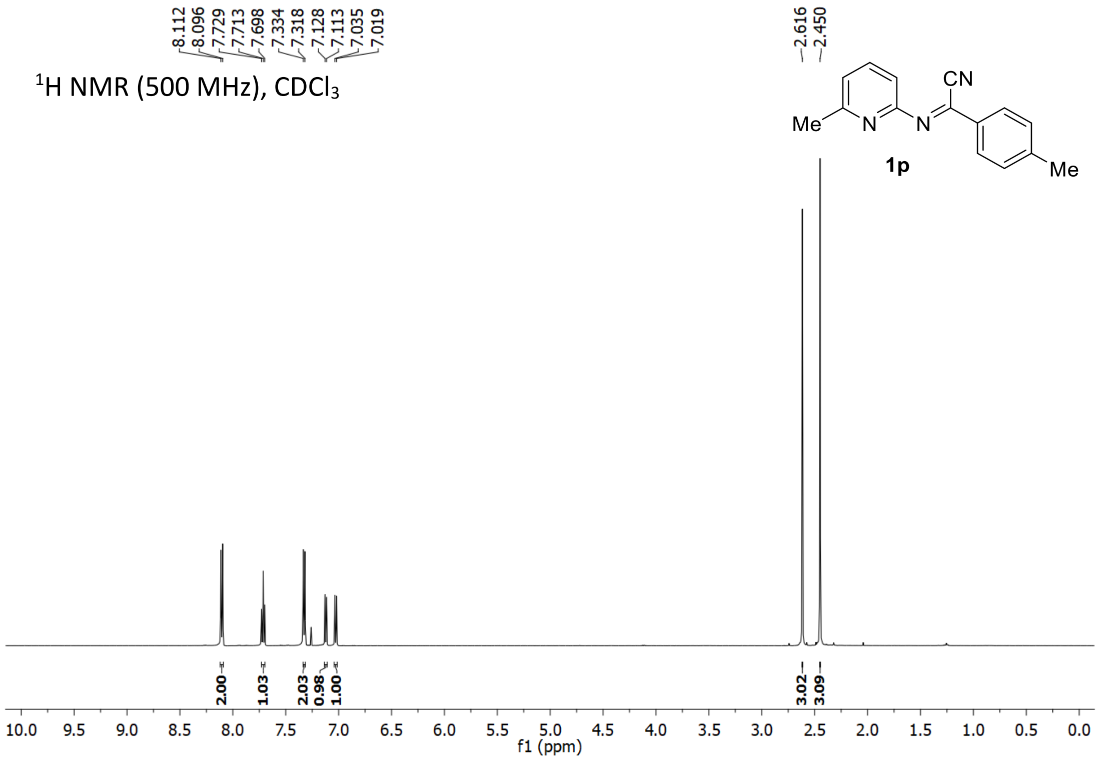

8.112
8.096
7.729
7.713
7.698
7.334
7.318
7.128
7.113
7.035
7.019
2.616
2.450
1H NMR (500 MHz), CDCl3
CN
Me
N
N
1p
Me
2.00
1.03
2.03
0.98
1.00
3.02
3.09
10.0
9.5
9.0
8.5
8.0
7.5
7.0
6.5
6.0
5.5
5.0
4.5
4.0
3.5
3.0
2.5
2.0
1.5
1.0
0.5
0.0
f1 (ppm)

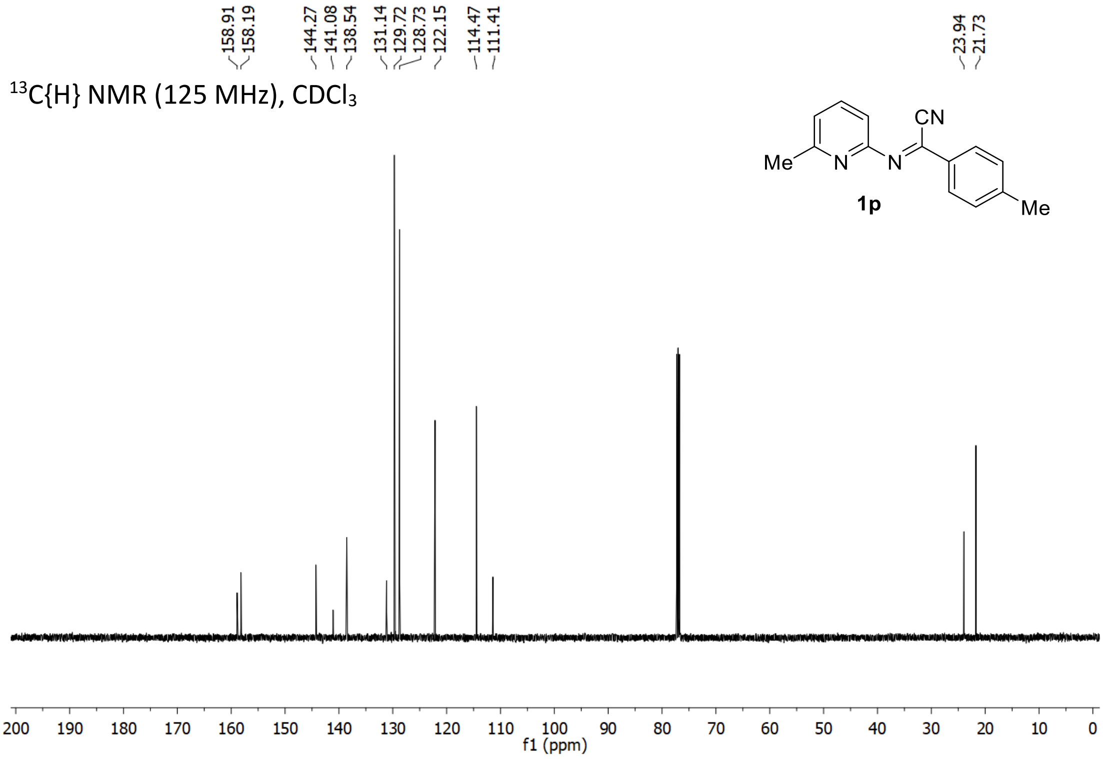

158.91
158.19
144.27
141.08
138.54
131.14
129.72
128.73
122.15
114.47
111.41
23.94
21.73
13C{H} NMR (125 MHz), CDCl3
CN
Me
N
N
1p
Me
200
190
180
170
160
150
140
130
120
110
100
90
80
70
60
50
40
30
20
10
0
f1 (ppm)

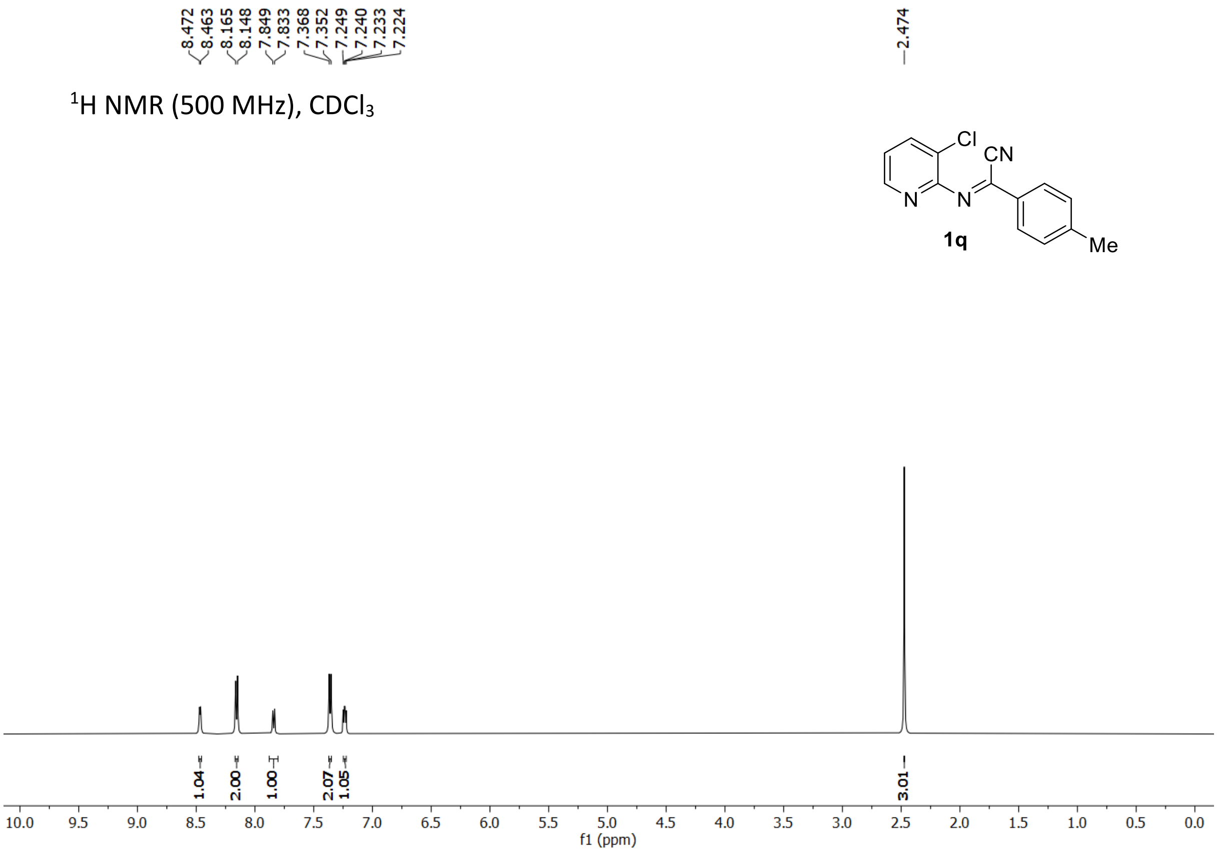
8.472
8.463
8.165
8.148
7.849
7.833
7.368
7.352
7.249
7.240
7.233
7.224
2.474
1H NMR (500 MHz), CDCl3
Cl
CN
N
N
1q
Me
1.04
2.00
1.00
2.07
1.05
3.01
10.0 9.5 9.0 8.5 8.0 7.5 7.0 6.5 6.0 5.5 5.0 4.5 4.0 3.5 3.0 2.5 2.0 1.5 1.0 0.5 0.0
f1 (ppm)

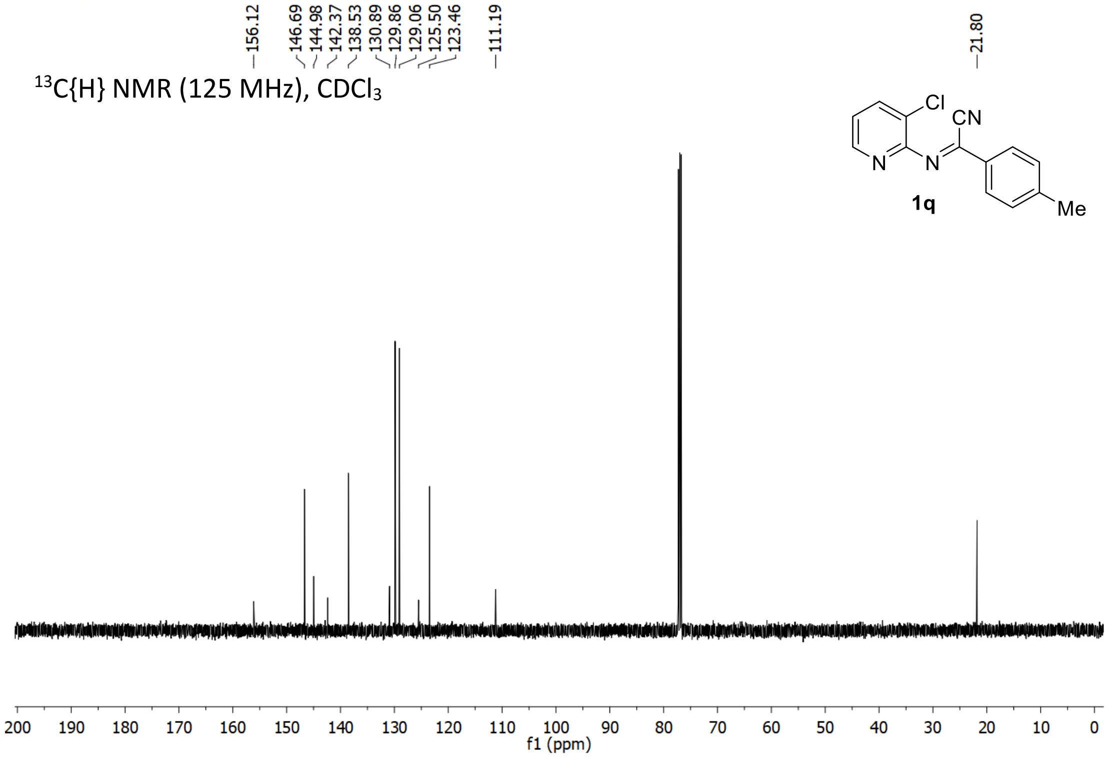
156.12
146.69
144.98
142.37
138.53
130.89
129.86
129.06
125.50
123.46
111.19
21.80
13C{H} NMR (125 MHz), CDCl3
Cl
CN
N
N
1q
Me
200 190 180 170 160 150 140 130 120 110 100 90 80 70 60 50 40 30 20 10 0
f1 (ppm)

^{1}H NMR (500 MHz), $CDCl_3$

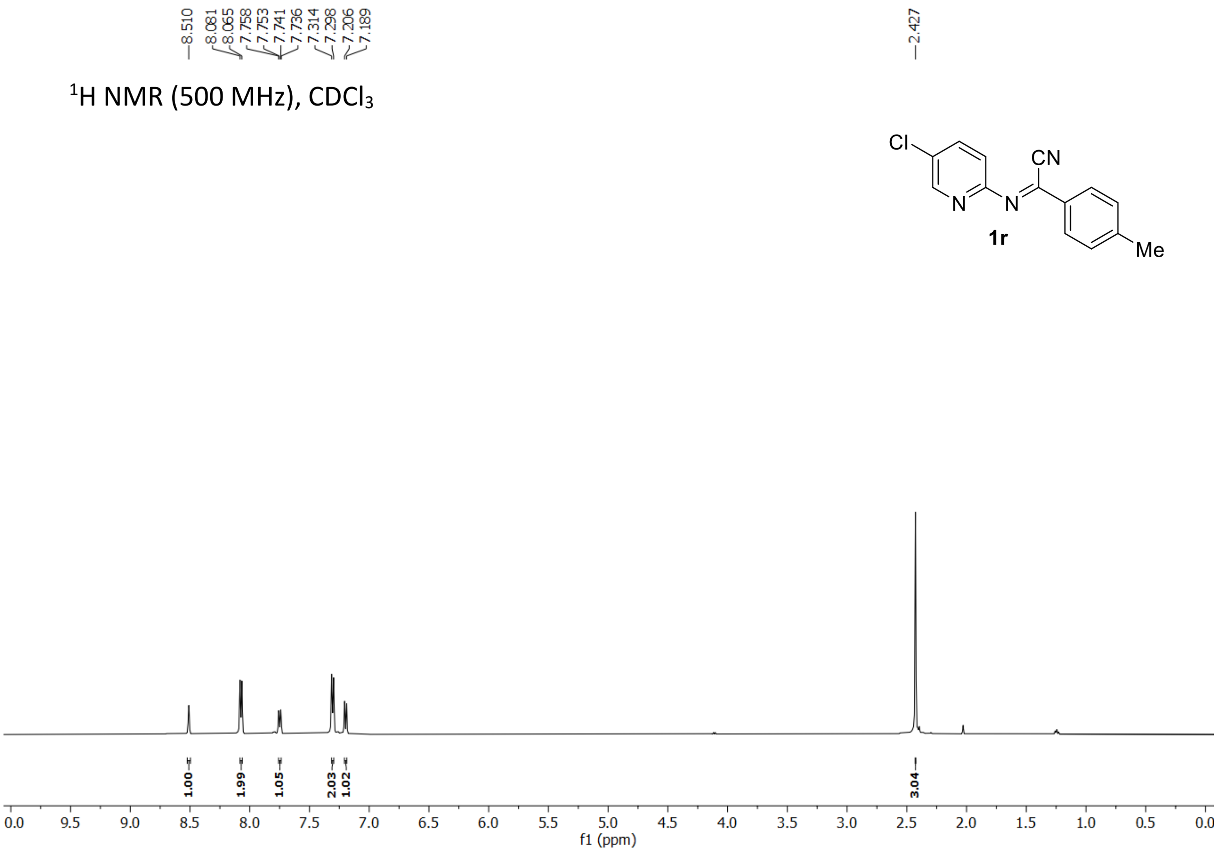

$^{13}C\{H\}$ NMR (125 MHz), $CDCl_3$

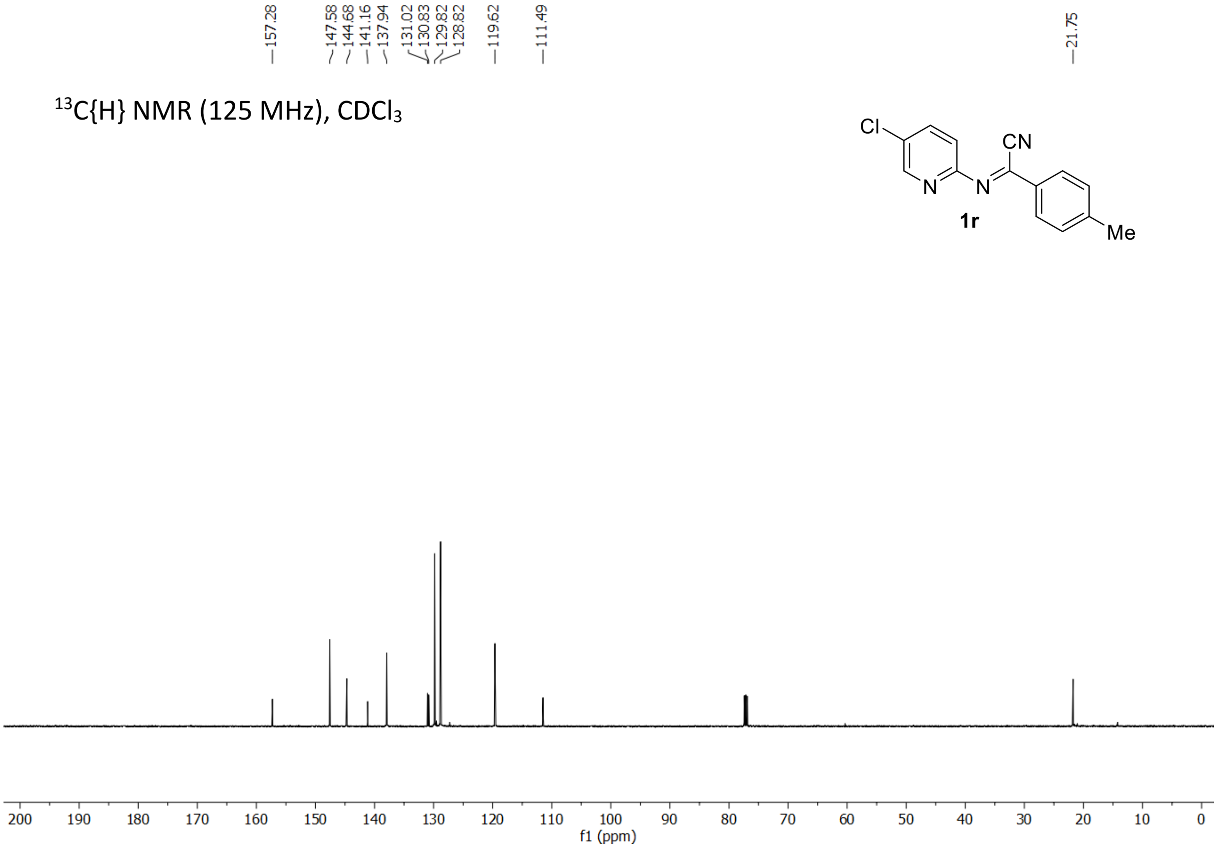

^{1}H NMR (500 MHz), $CDCl_3$

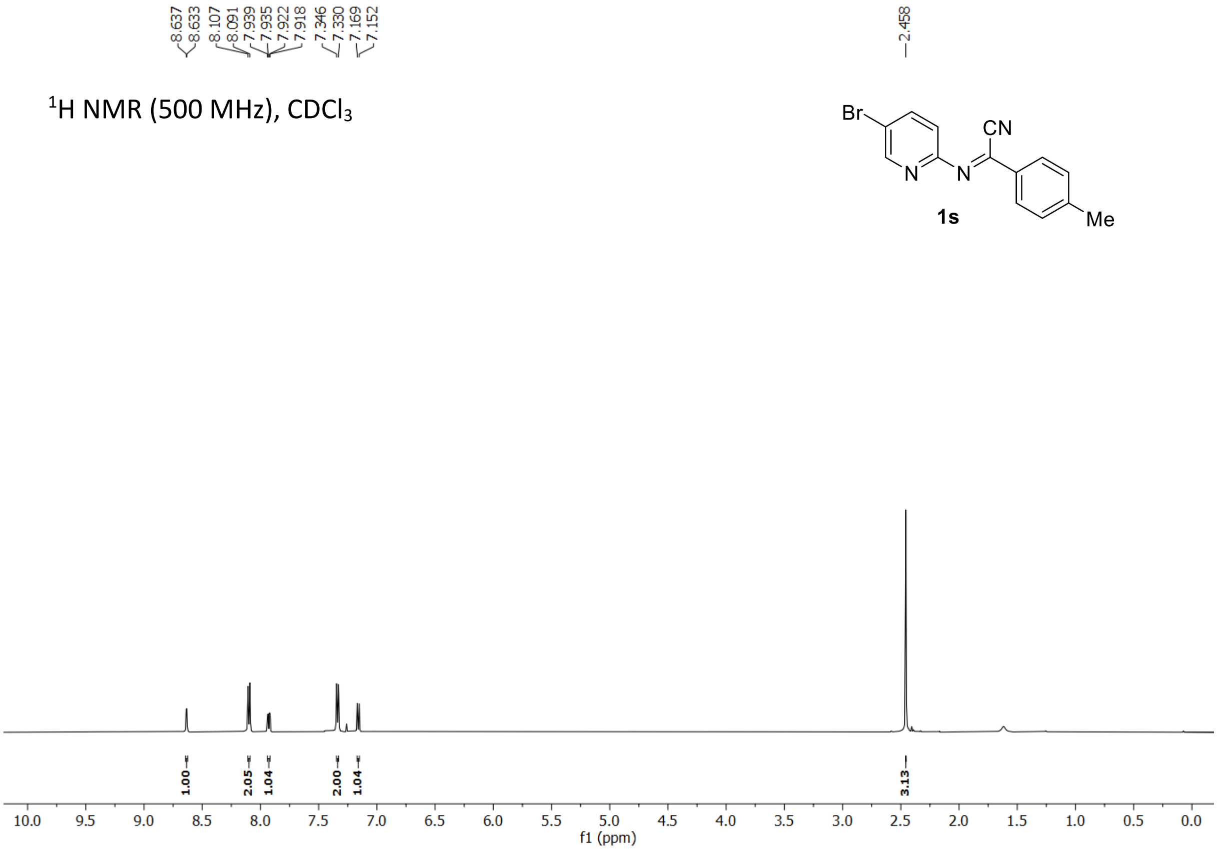

$^{13}C\{H\}$ NMR (125 MHz), $CDCl_3$

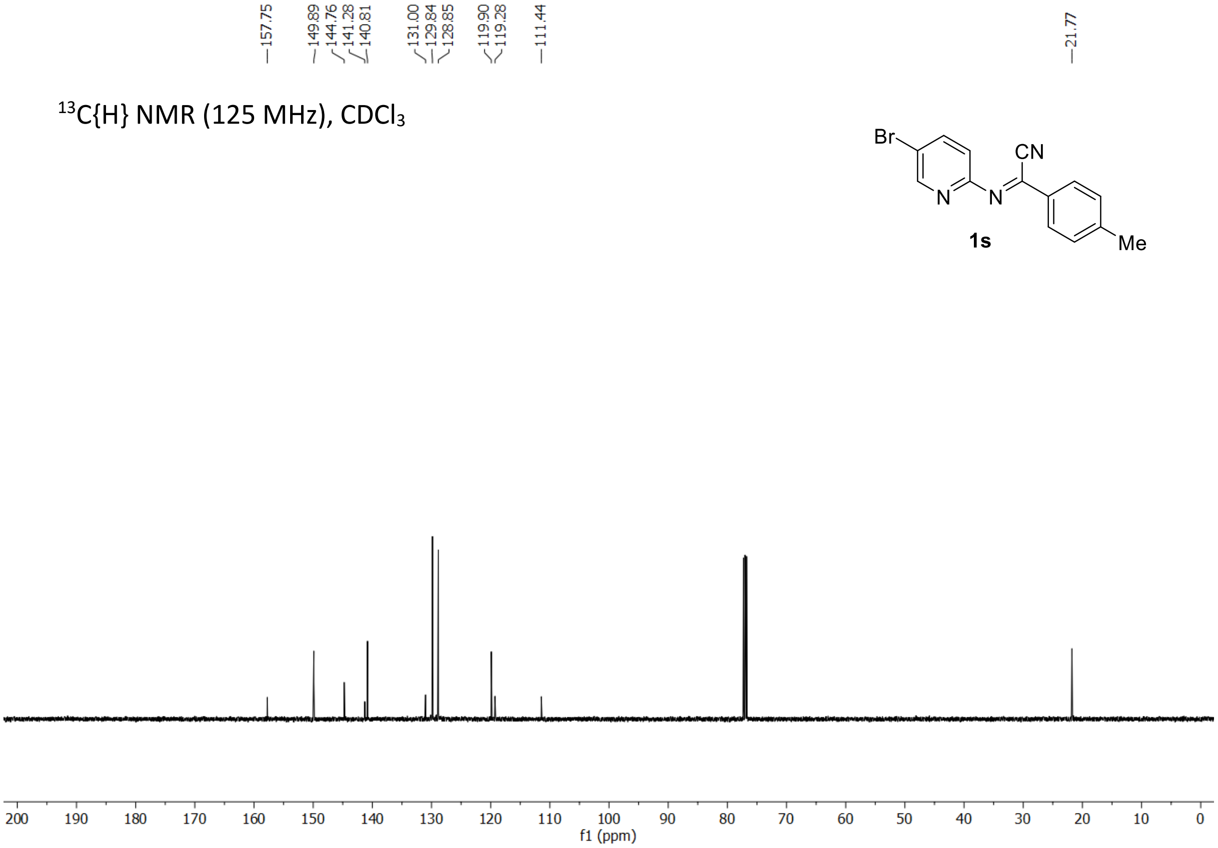

Copies of ¹H and ¹³C{H} NMR spectra of imidates 2aa-2ad, 2aj, 2ba-2hc, 2ja-2sa

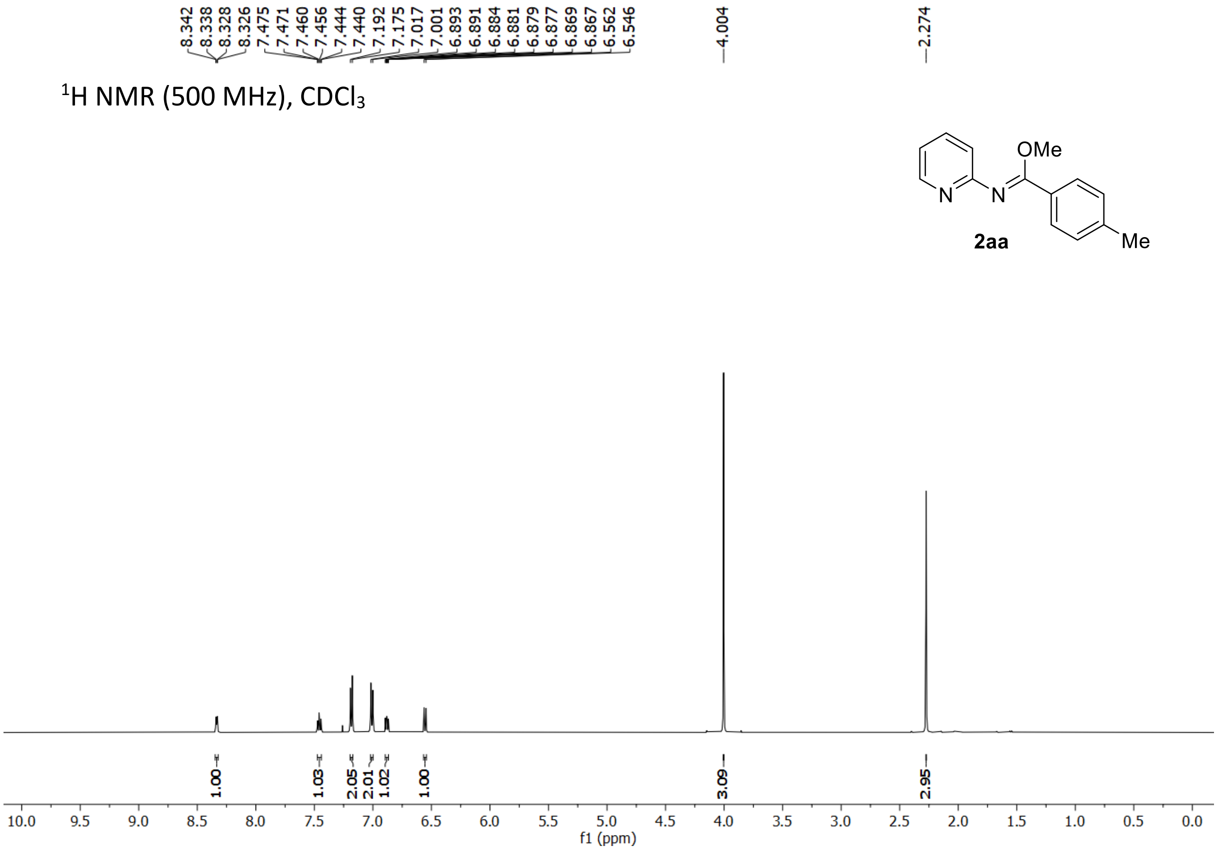

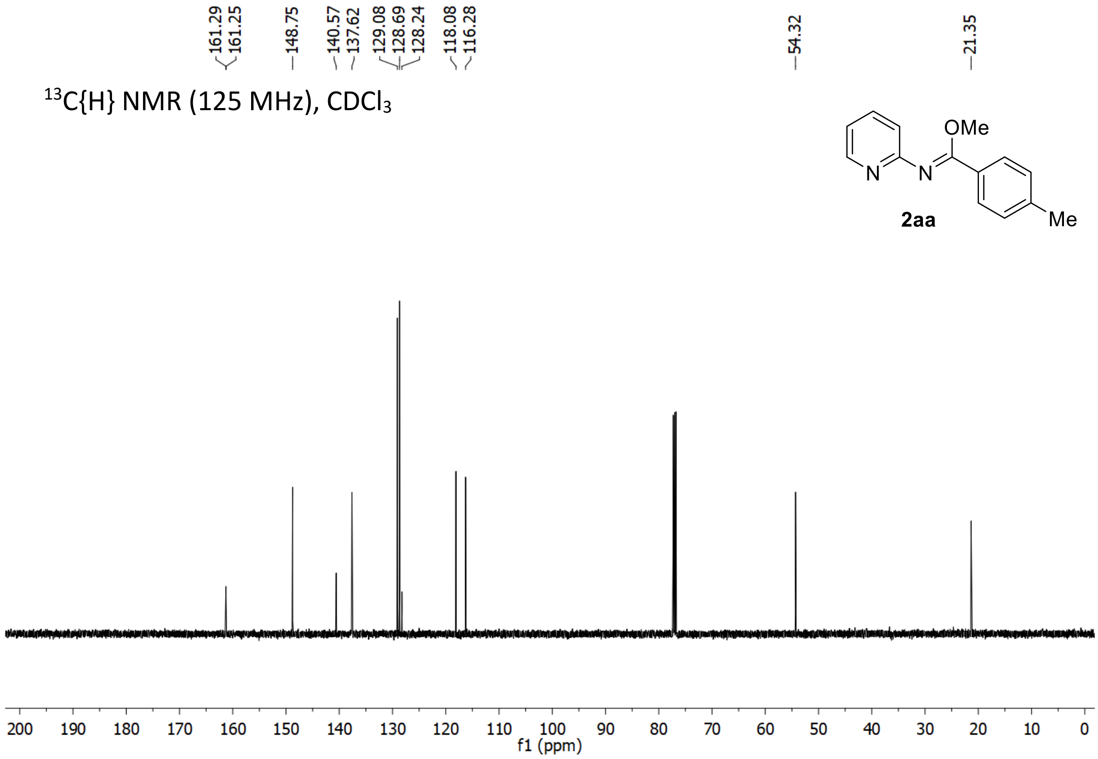

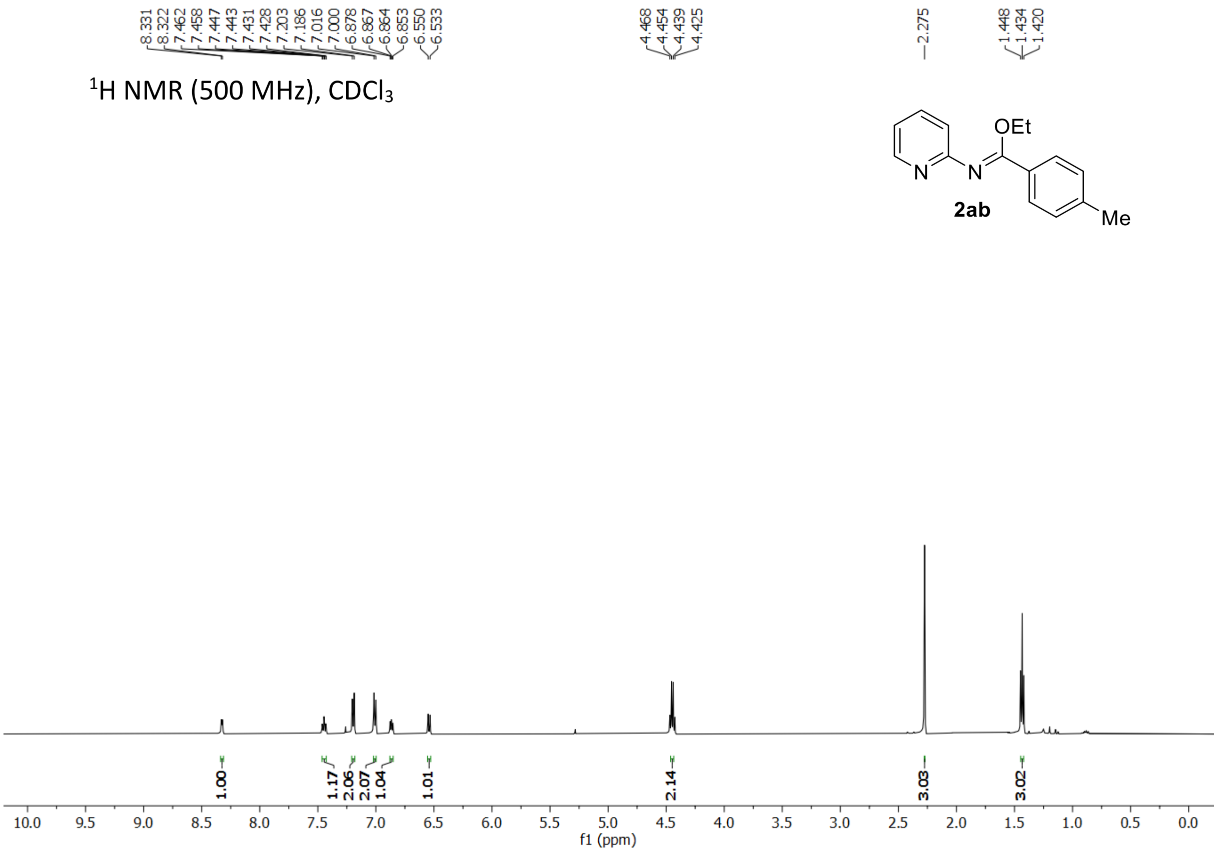
8.331
8.322
7.462
7.458
7.447
7.443
7.431
7.428
7.203
7.186
7.016
7.000
6.878
6.867
6.864
6.853
6.550
6.533
4.468
4.454
4.439
4.425
2.275
1.448
1.434
1.420
¹H NMR (500 MHz), CDCl₃
OEt
N
N
2ab
Me
1.00
1.17
2.06
2.07
1.04
1.01
2.14
3.03
3.02
f1 (ppm)

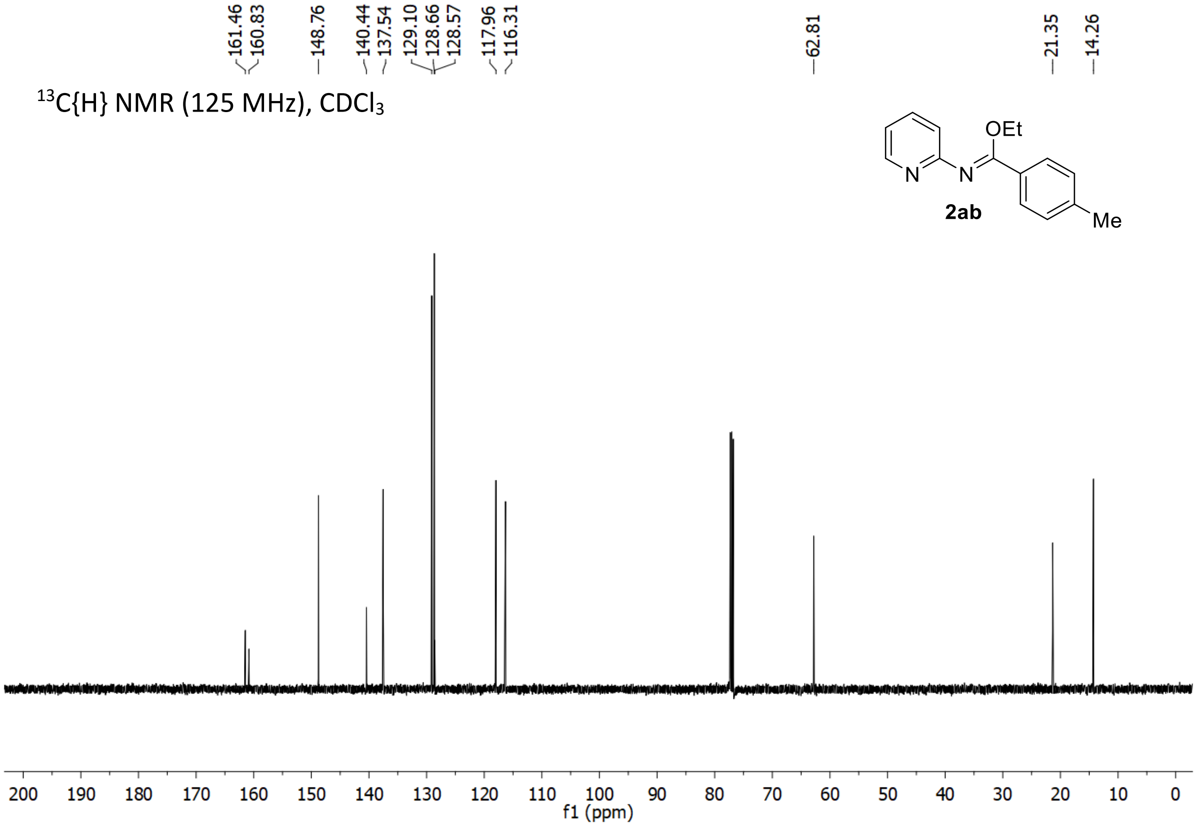
161.46
160.83
148.76
140.44
137.54
129.10
128.66
128.57
117.96
116.31
62.81
21.35
14.26
¹³C{H} NMR (125 MHz), CDCl₃
OEt
N
N
2ab
Me
f1 (ppm)

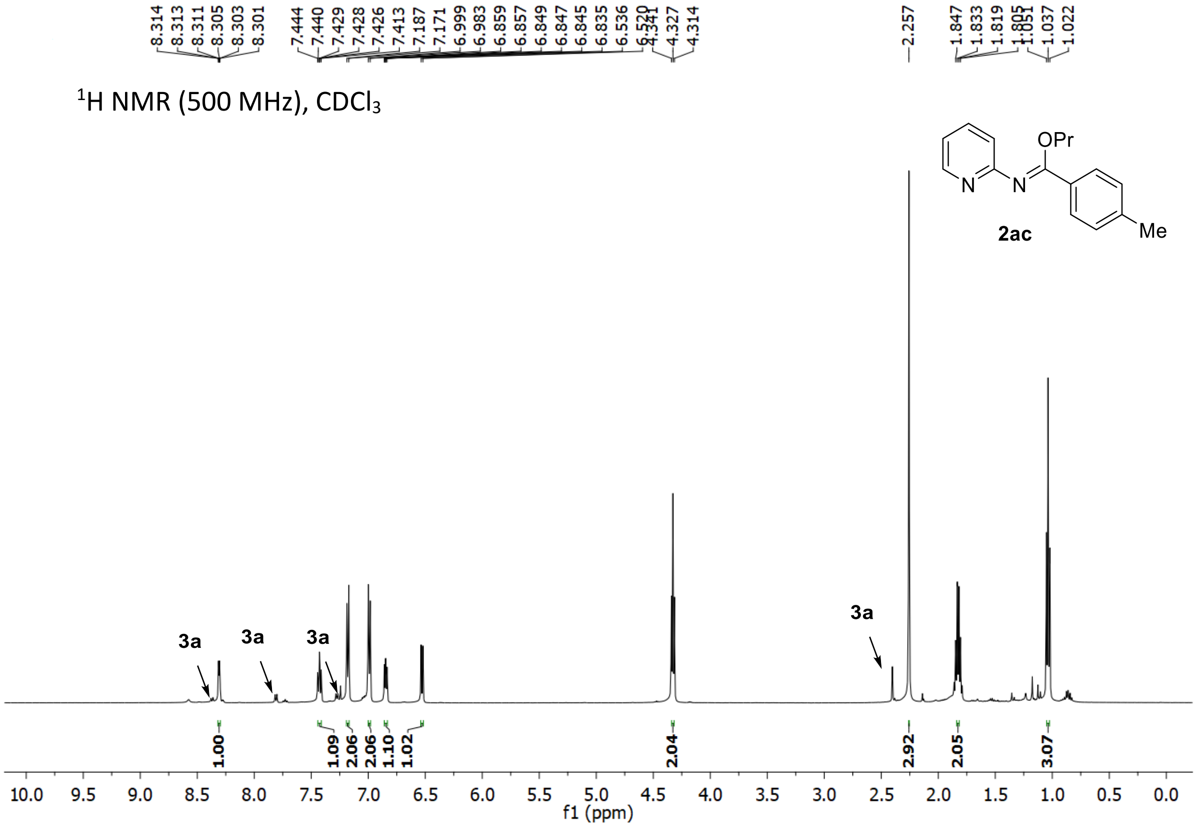
8.314
8.313
8.311
8.305
8.303
8.301
7.444
7.440
7.429
7.428
7.426
7.413
7.187
7.171
6.999
6.983
6.859
6.857
6.849
6.847
6.845
6.835
6.536
6.520
4.341
4.327
4.314
2.257
1.847
1.833
1.819
1.805
1.051
1.037
1.022
1H NMR (500 MHz), CDCl3
OPr
N
N
Me
2ac
3a
3a
3a
3a
1.00
1.09
2.06
2.06
1.10
1.02
2.04
2.92
2.05
3.07
f1 (ppm)

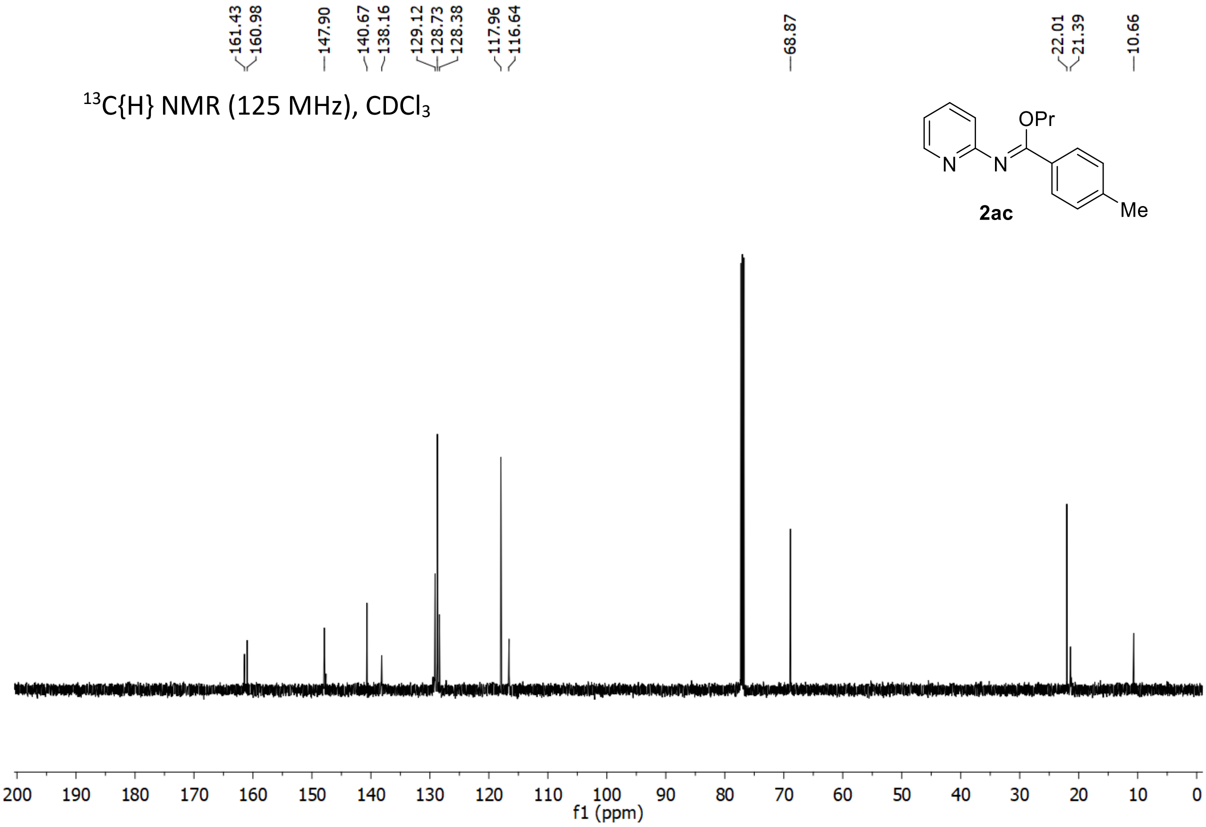
161.43
160.98
147.90
140.67
138.16
129.12
128.73
128.38
117.96
116.64
68.87
22.01
21.39
10.66
13C{H} NMR (125 MHz), CDCl3
OPr
N
N
Me
2ac
f1 (ppm)

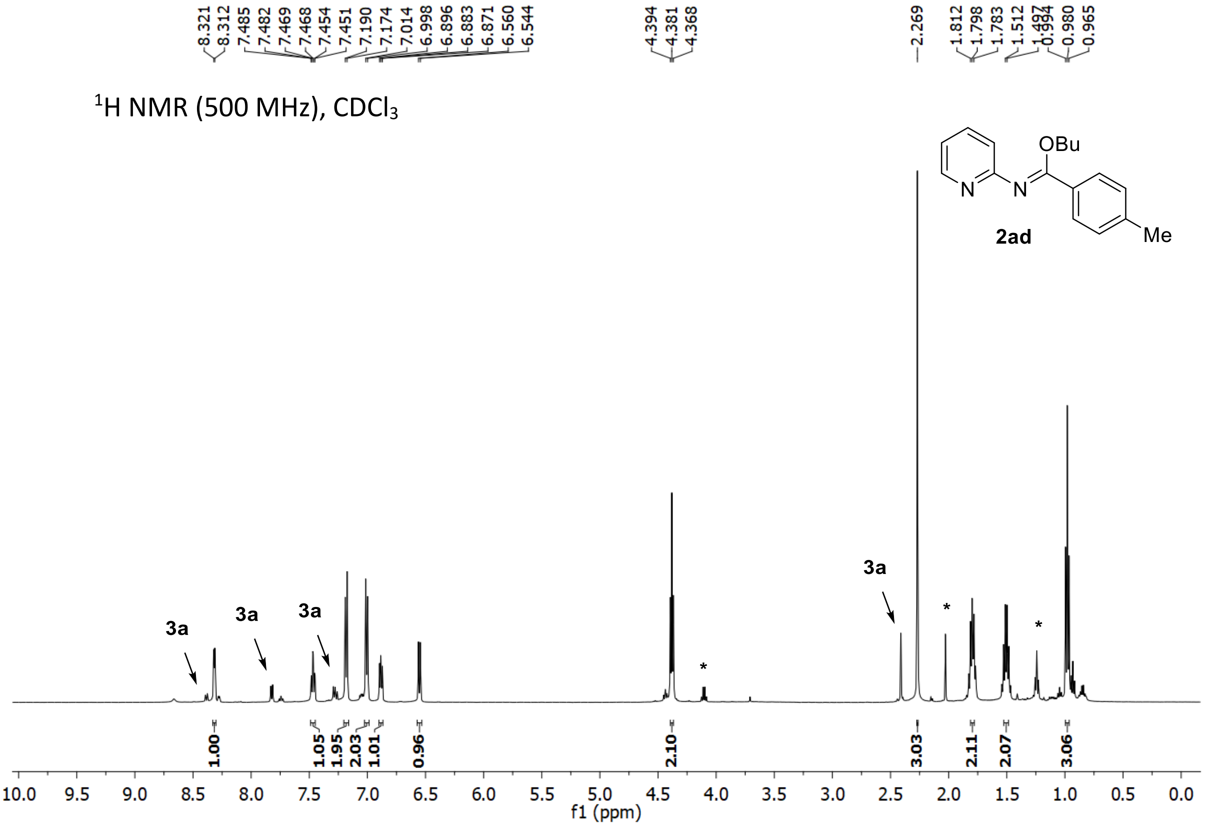
8.321
8.312
7.485
7.482
7.469
7.468
7.454
7.451
7.190
7.174
7.014
6.998
6.896
6.883
6.871
6.560
6.544
4.394
4.381
4.368
2.269
1.812
1.798
1.783
1.512
1.497
0.994
0.980
0.965
1H NMR (500 MHz), CDCl3
OBu
N
N
2ad
Me
3a
3a
3a
3a
*
*
*
1.00
1.05
1.95
2.03
1.01
0.96
2.10
3.03
2.11
2.07
3.06
f1 (ppm)

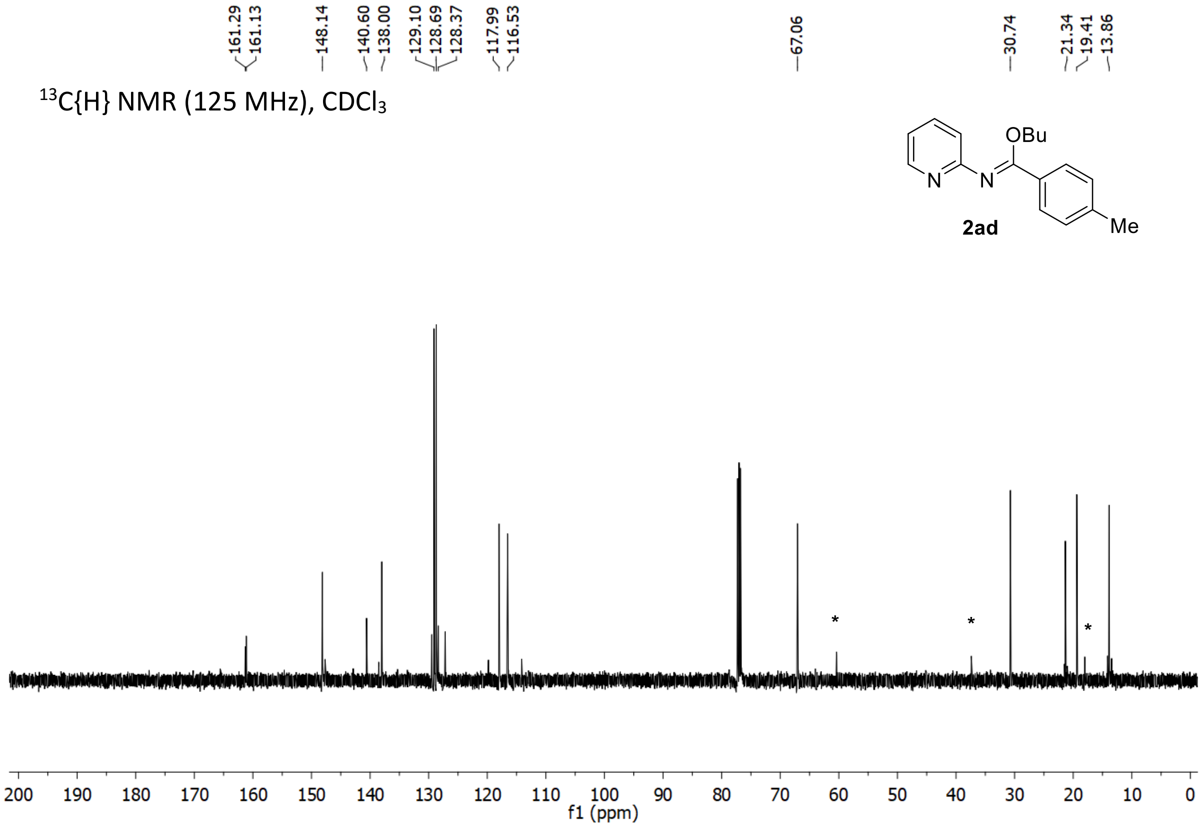
161.29
161.13
148.14
140.60
138.00
129.10
128.69
128.37
117.99
116.53
67.06
30.74
21.34
19.41
13.86
13C{H} NMR (125 MHz), CDCl3
OBu
N
N
2ad
Me
*
*
*
f1 (ppm)

* EtOAc

^{1}H NMR (500 MHz), $CDCl_3$

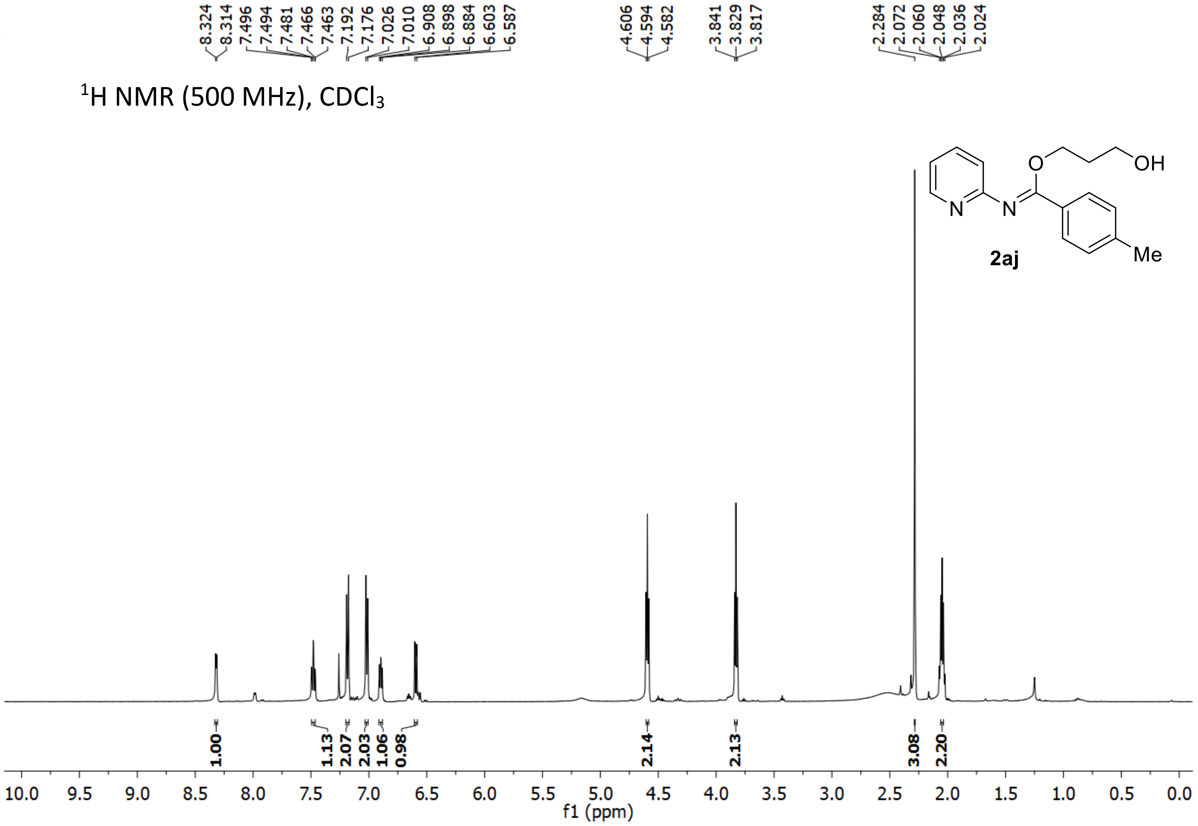

$^{13}C\{H\}$ NMR (125 MHz), $CDCl_3$

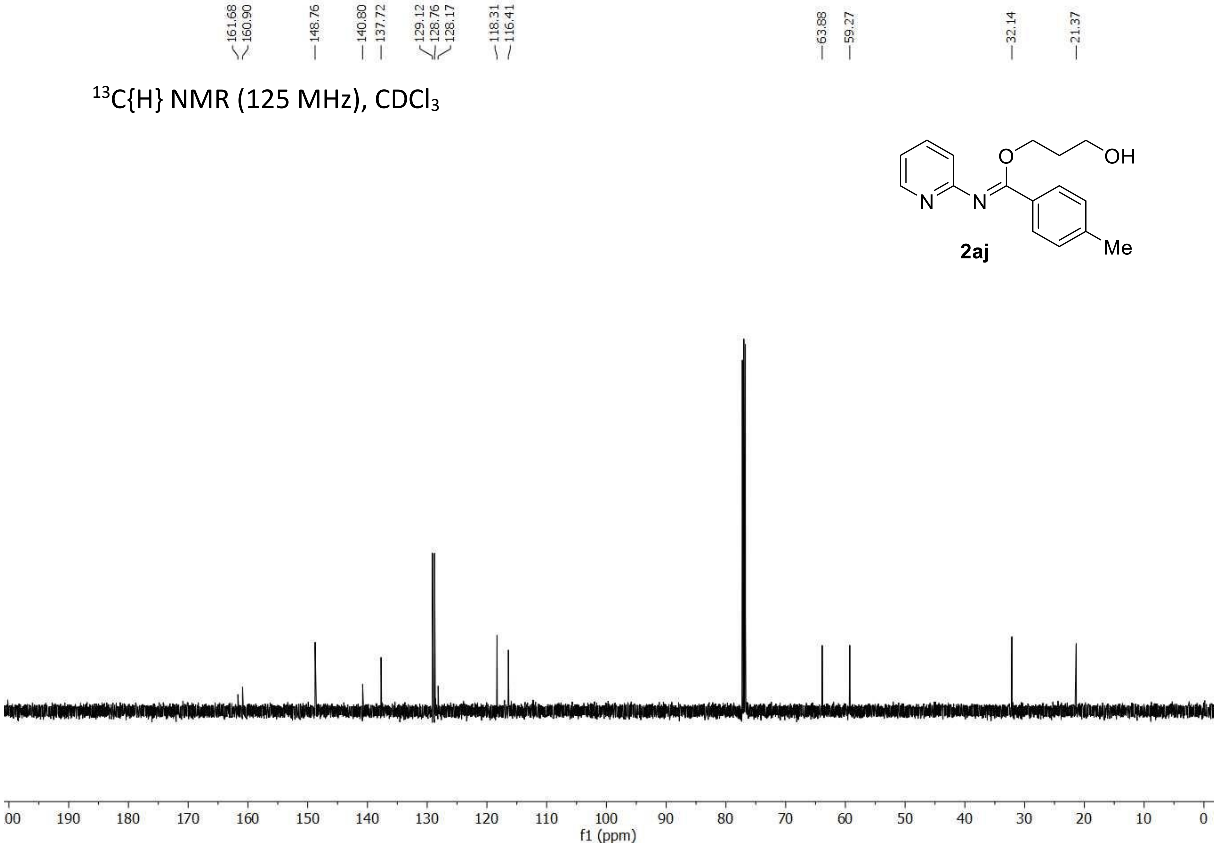

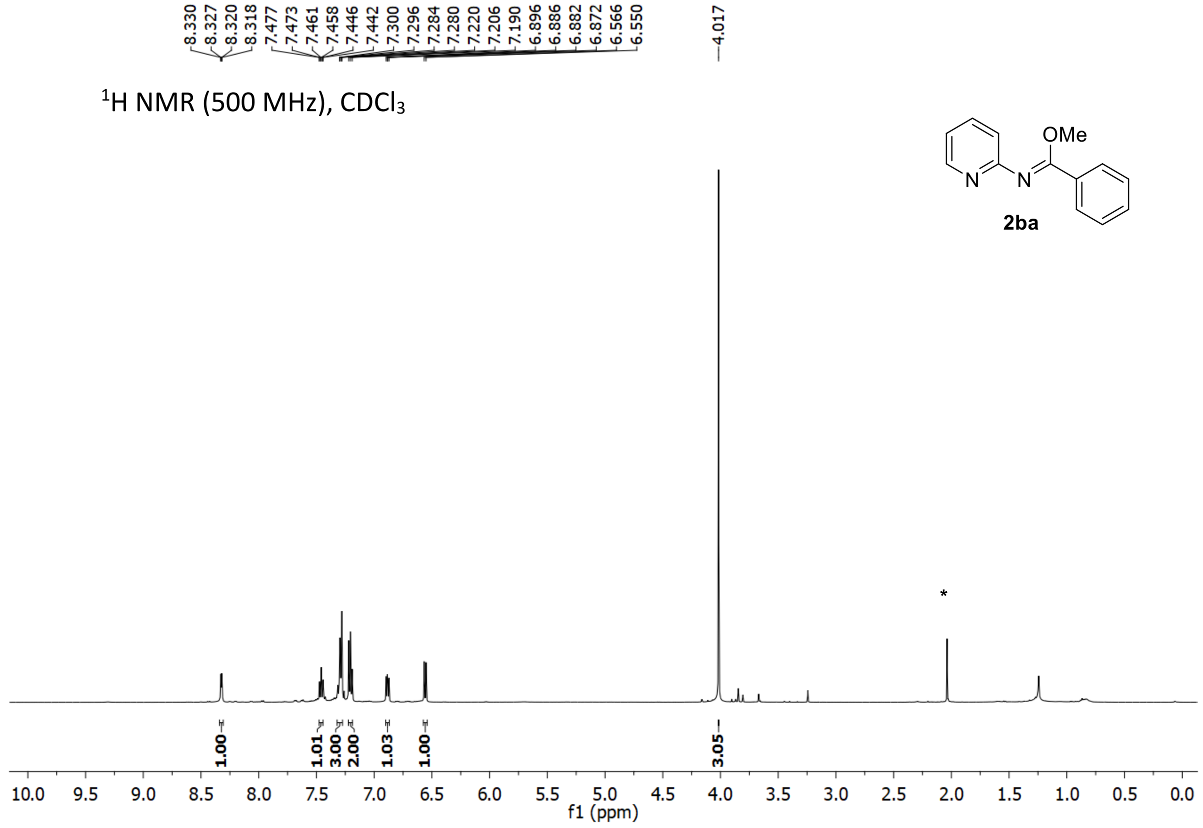
8.330
8.327
8.320
8.318
7.477
7.473
7.461
7.458
7.446
7.442
7.300
7.296
7.284
7.280
7.220
7.206
7.190
6.896
6.886
6.882
6.872
6.566
6.550
4.017
^{1}H NMR (500 MHz), $CDCl_3$
OMe
N
N
2ba
*
1.00
1.01
3.00
2.00
1.03
1.00
3.05
f1 (ppm)

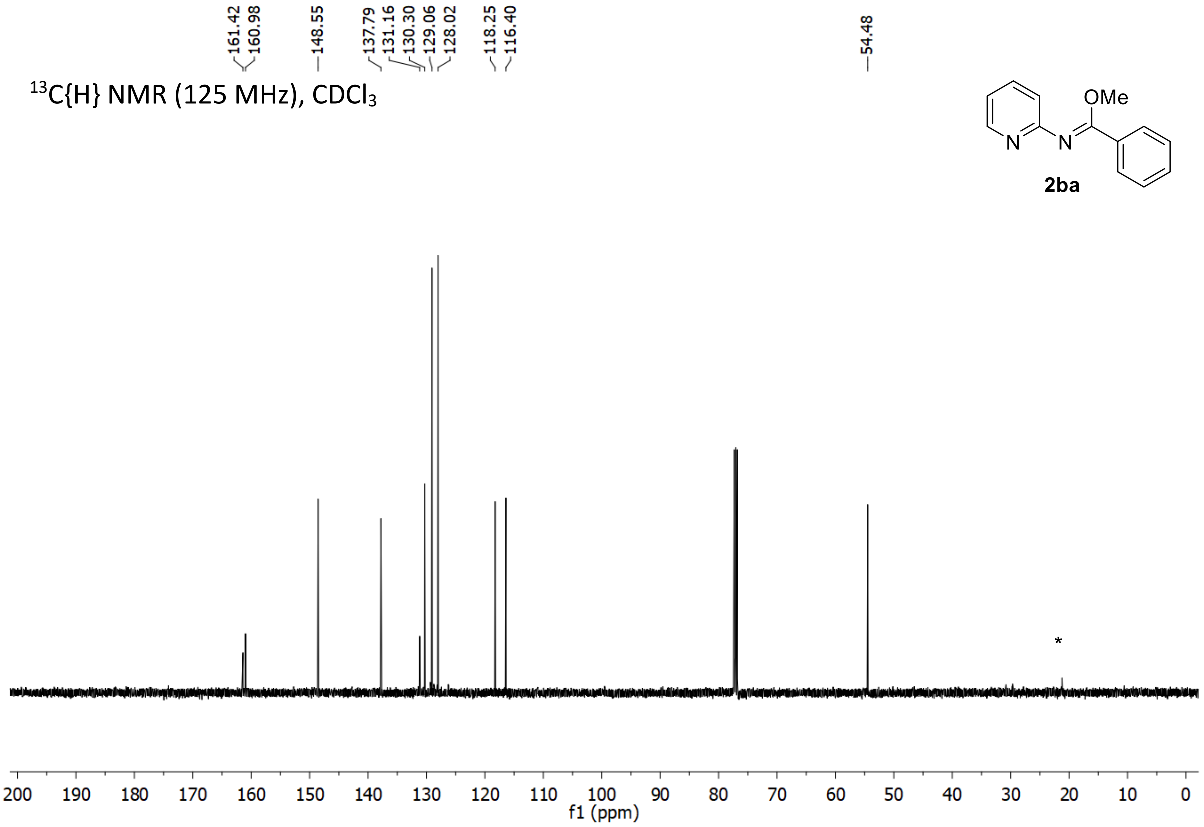
161.42
160.98
148.55
137.79
131.16
130.30
129.06
128.02
118.25
116.40
54.48
^{13}C{H} NMR (125 MHz), $CDCl_3$
OMe
N
N
2ba
*
f1 (ppm)

*acetone

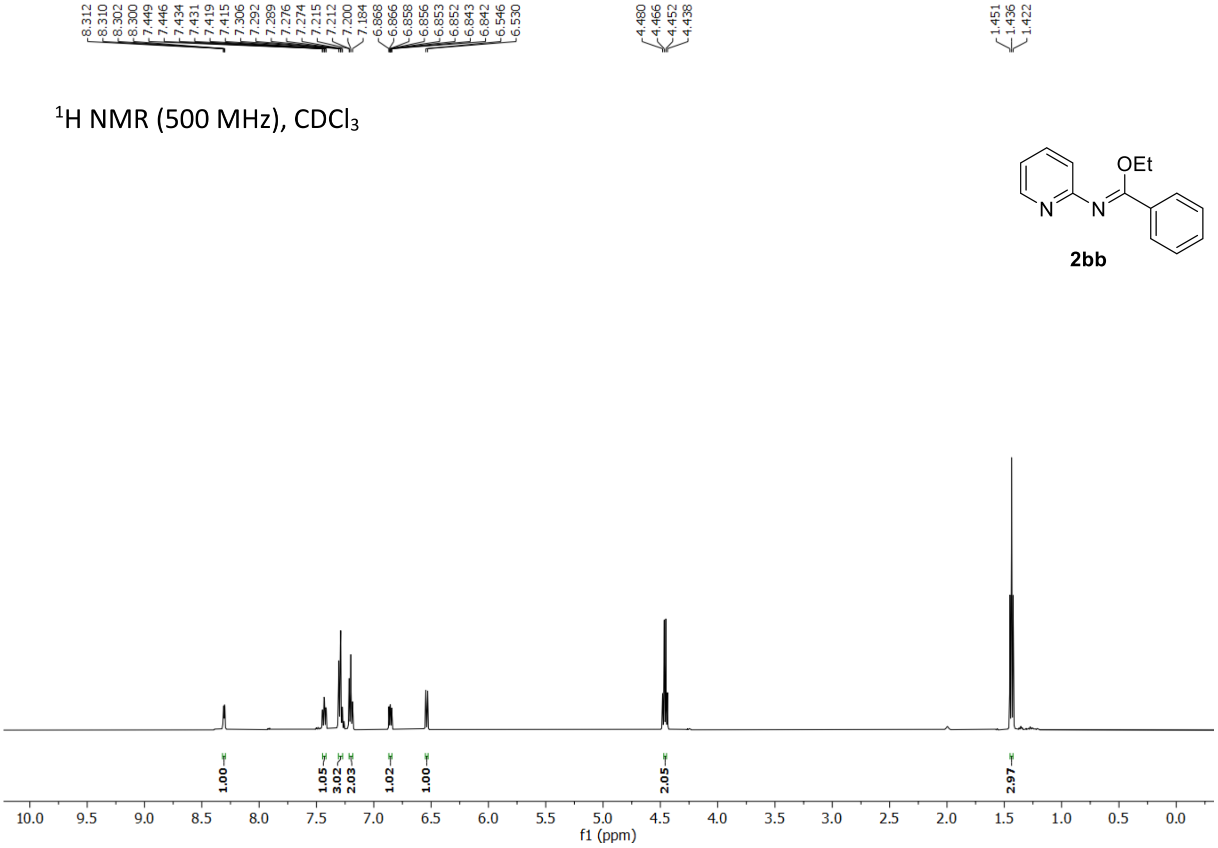
8.312 8.310 8.302 8.300 7.449 7.446 7.434 7.431 7.419 7.415 7.306 7.292 7.289 7.276 7.274 7.215 7.212 7.200 7.184 6.868 6.866 6.858 6.856 6.853 6.852 6.843 6.842 6.546 6.530
4.480 4.466 4.452 4.438
1.451 1.436 1.422
^{1}H NMR (500 MHz), CDCl$_3$
OEt
N
N
2bb
1.00 1.05 3.02 2.03 1.02 1.00 2.05 2.97
10.0 9.5 9.0 8.5 8.0 7.5 7.0 6.5 6.0 5.5 5.0 4.5 4.0 3.5 3.0 2.5 2.0 1.5 1.0 0.5 0.0
f1 (ppm)

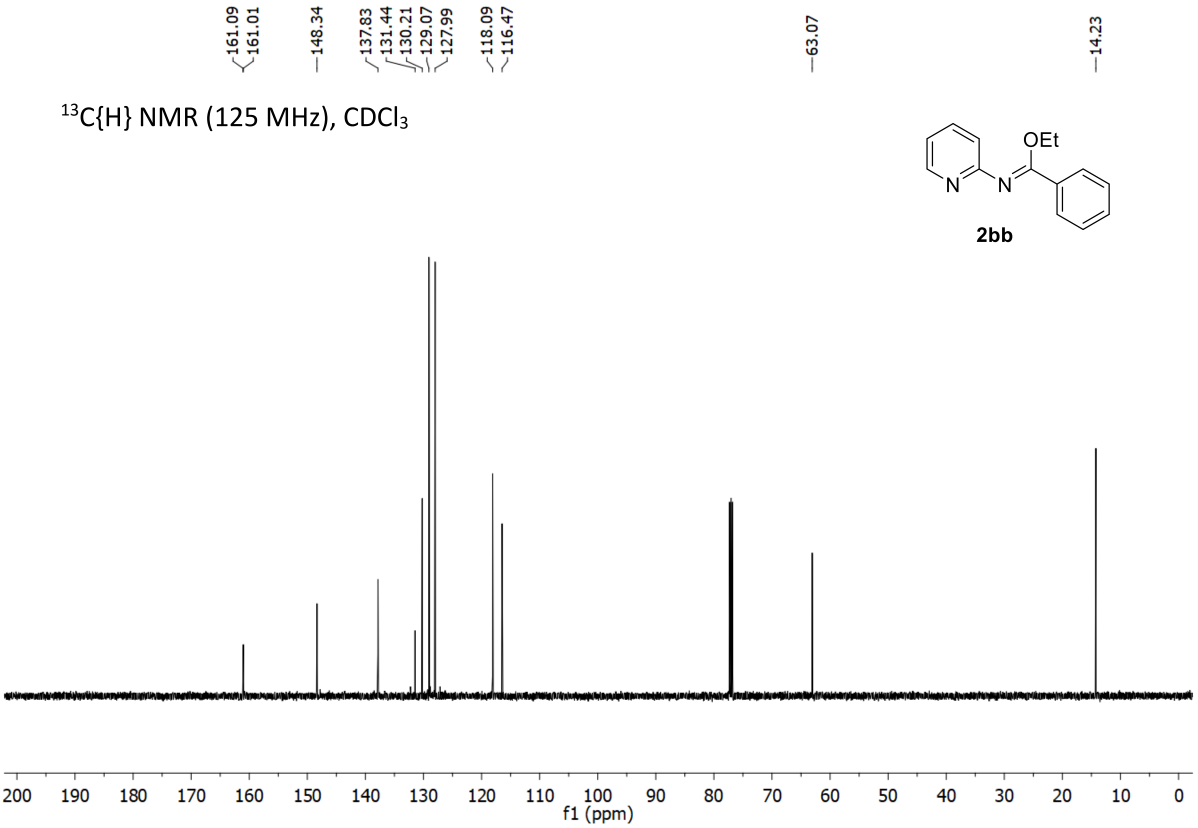
161.09 161.01 148.34 137.83 131.44 130.21 129.07 127.99 118.09 116.47
63.07
14.23
^{13}C{H} NMR (125 MHz), CDCl$_3$
OEt
N
N
2bb
200 190 180 170 160 150 140 130 120 110 100 90 80 70 60 50 40 30 20 10 0
f1 (ppm)

^{1}H NMR (500 MHz), $CDCl_3$

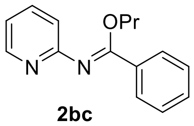

2bc

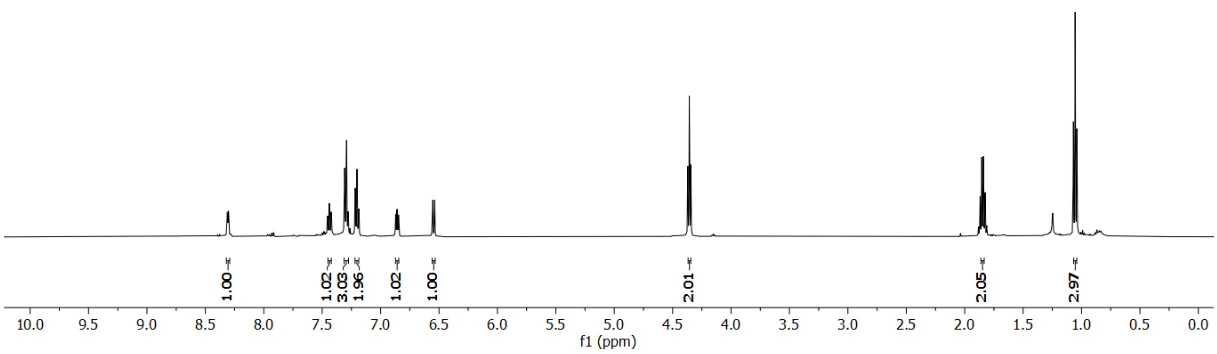

$^{13}C\{H\}$ NMR (125 MHz), $CDCl_3$

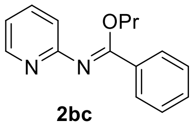

2bc

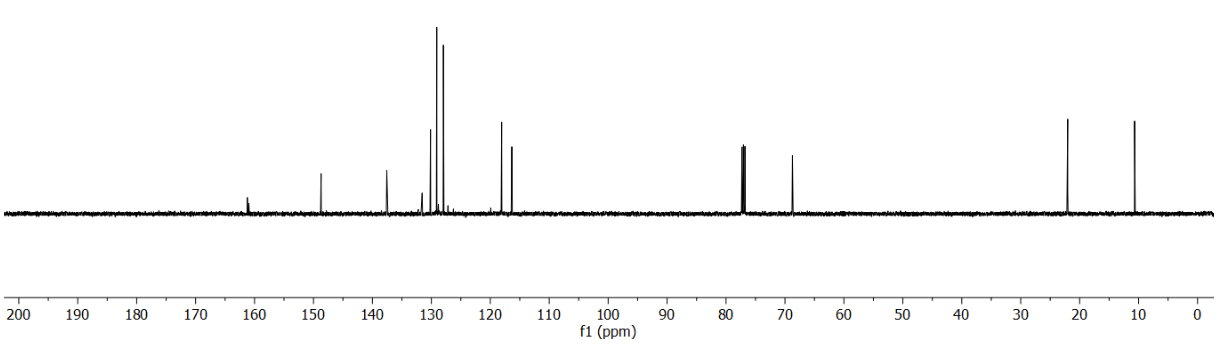

^{1}H NMR (500 MHz), $CDCl_3$

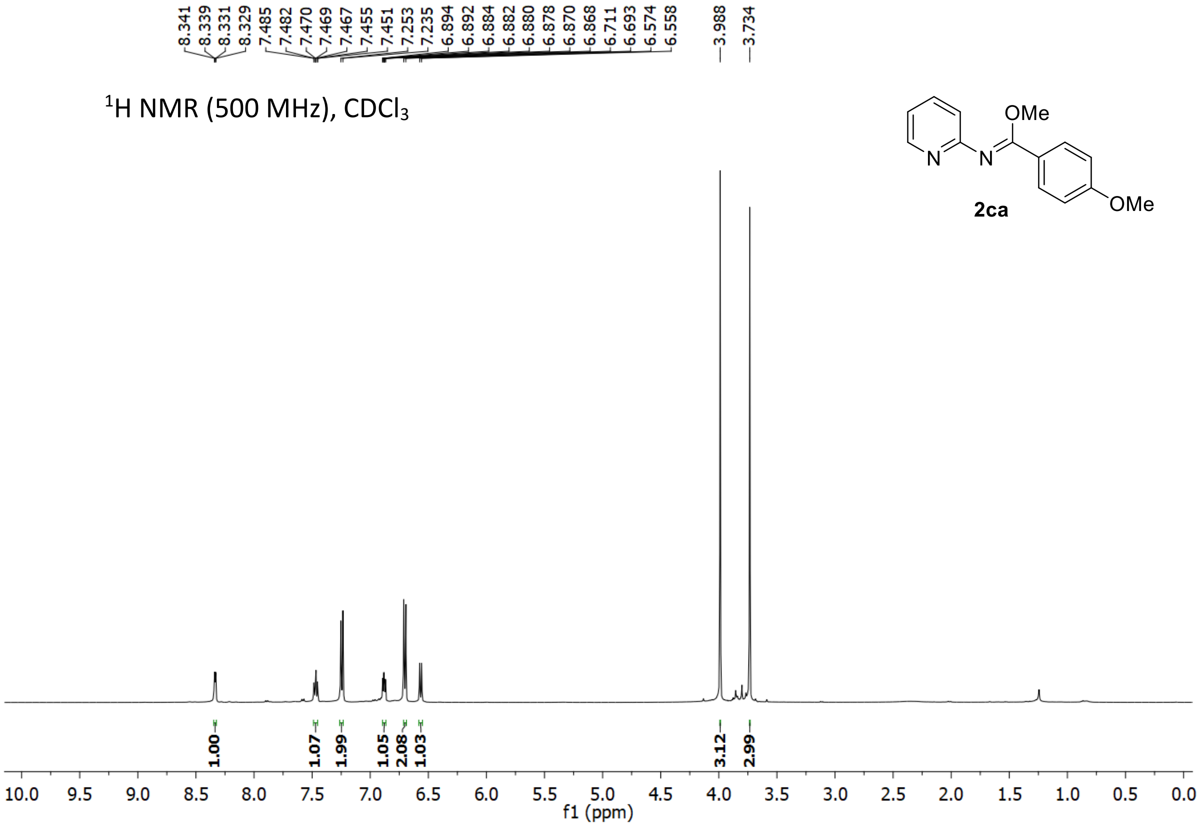

$^{13}C\{H\}$ NMR (125 MHz), $CDCl_3$

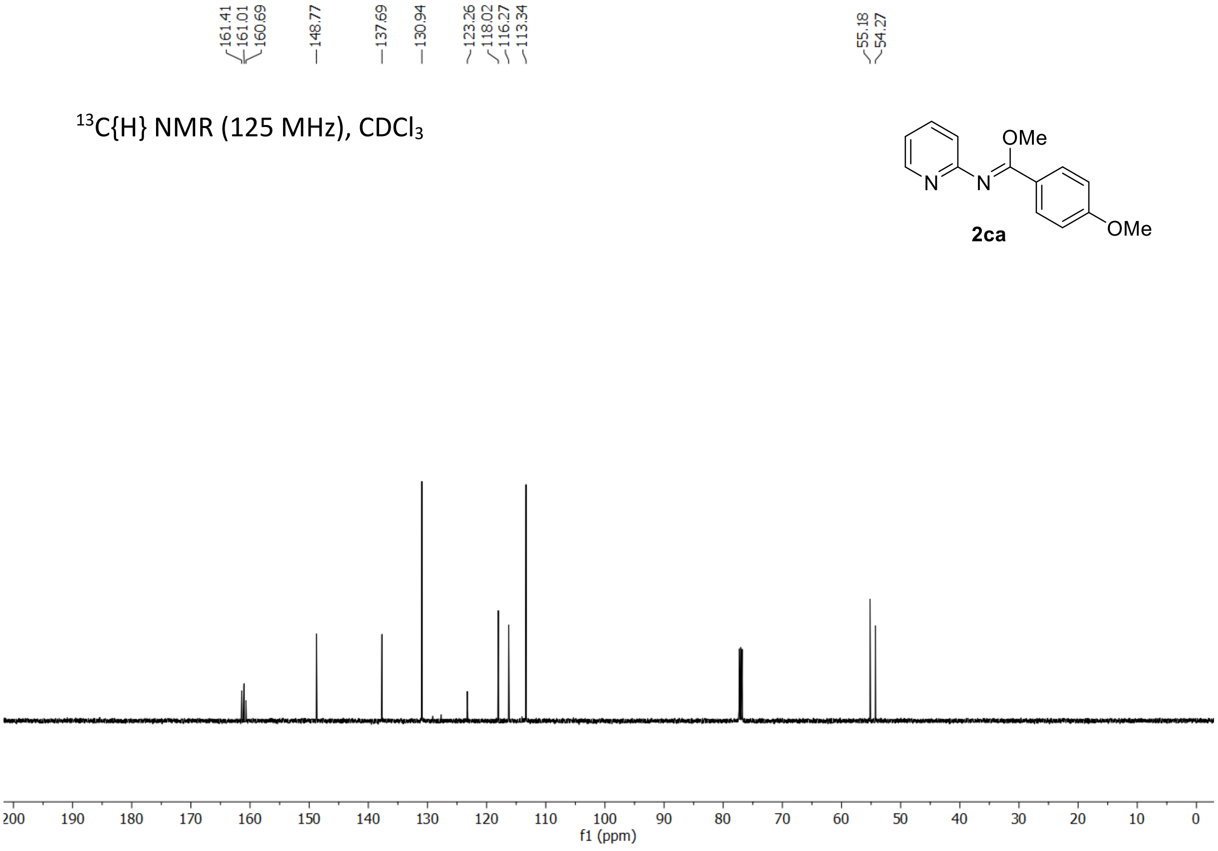

^{1}H NMR (500 MHz), $CDCl_3$

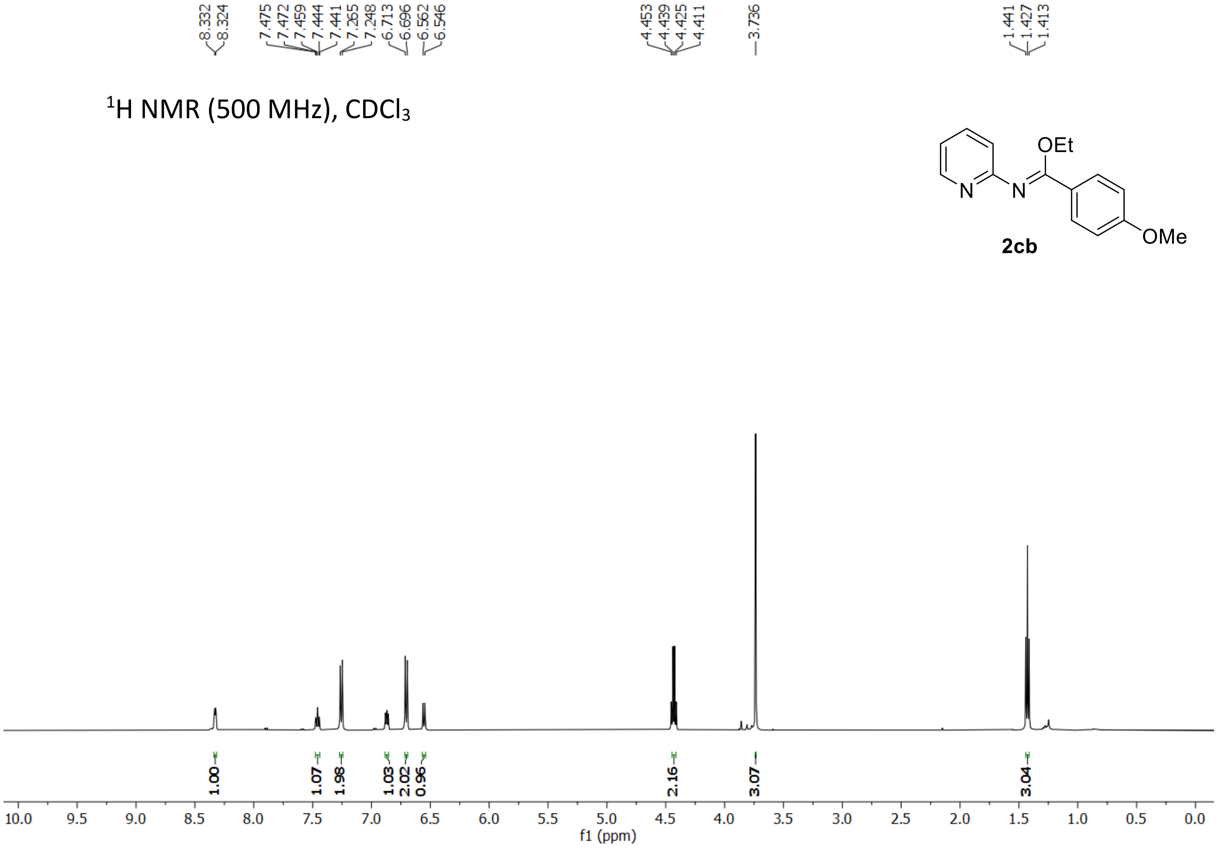

2cb

$^{13}C\{H\}$ NMR (125 MHz), $CDCl_3$

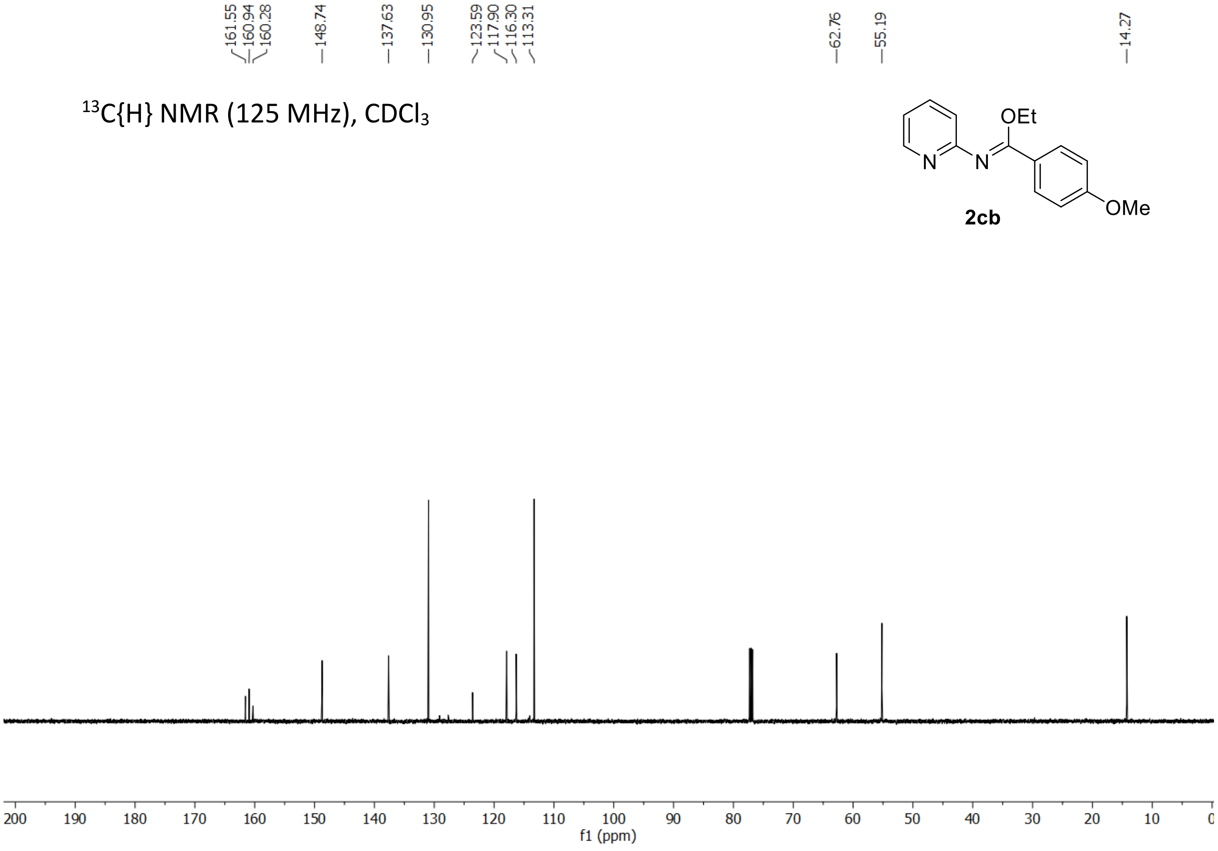

2cb

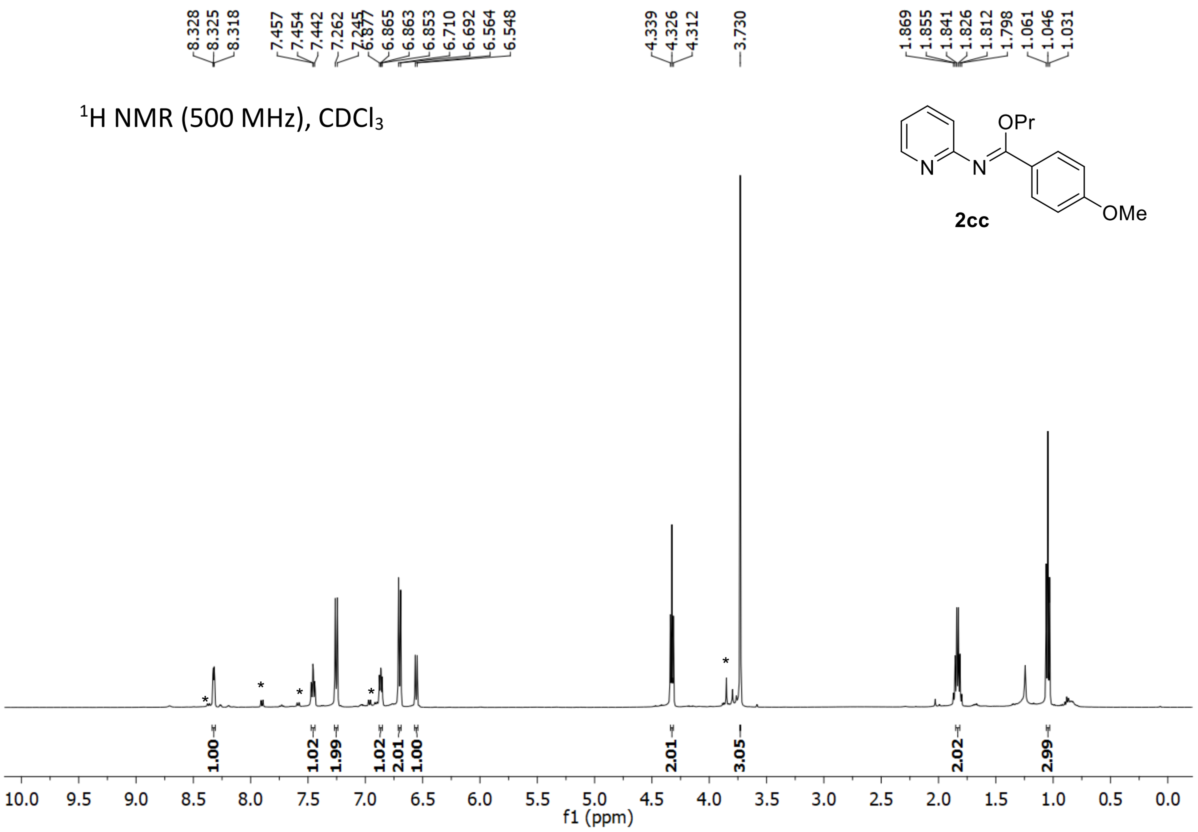

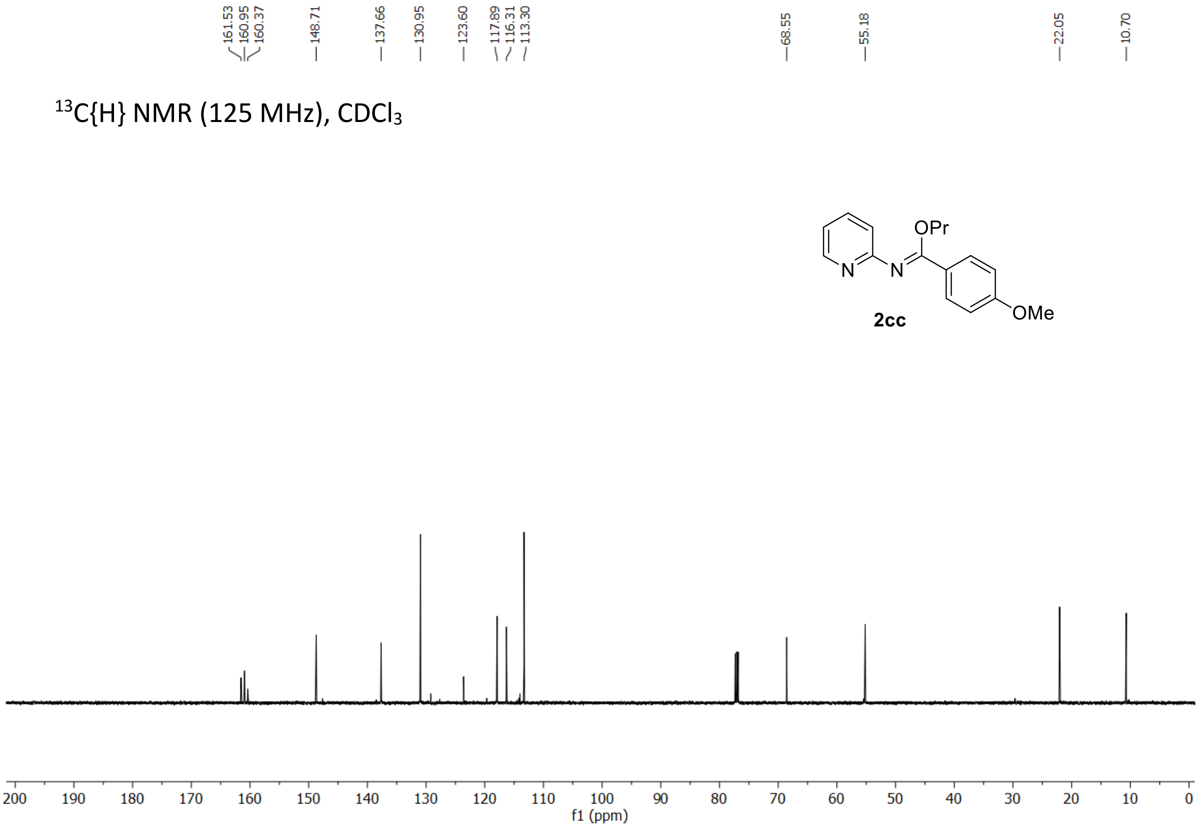

*corresponding amide

^{1}H NMR (500 MHz), $CDCl_3$

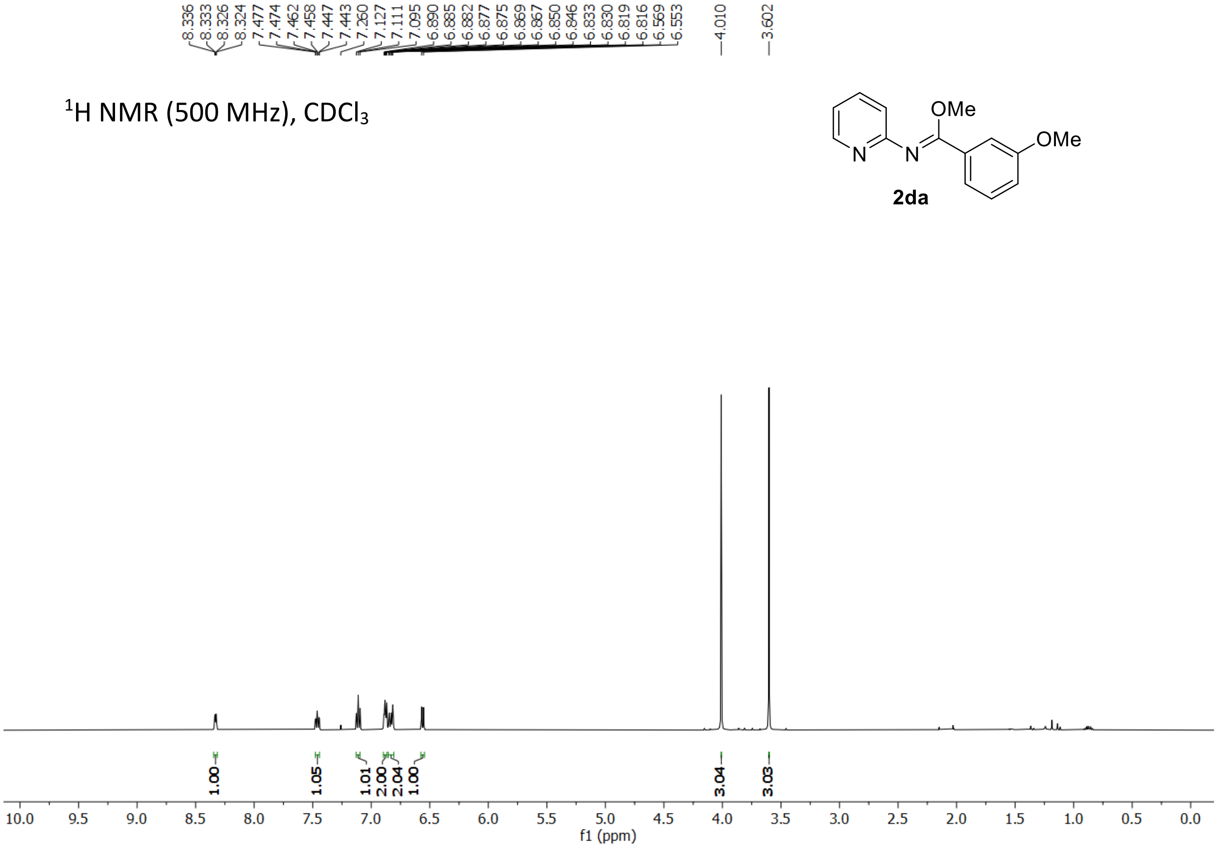

^{13}C{H} NMR (125 MHz), $CDCl_3$

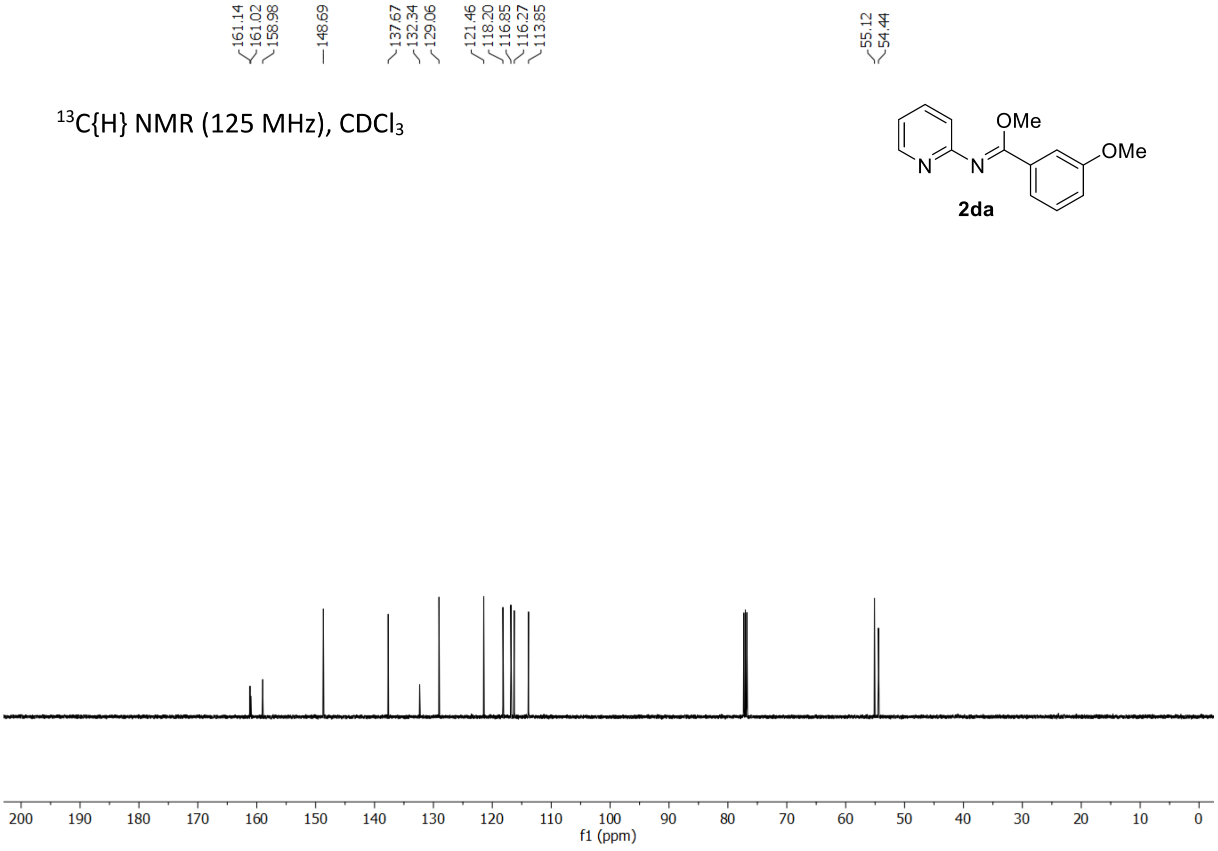

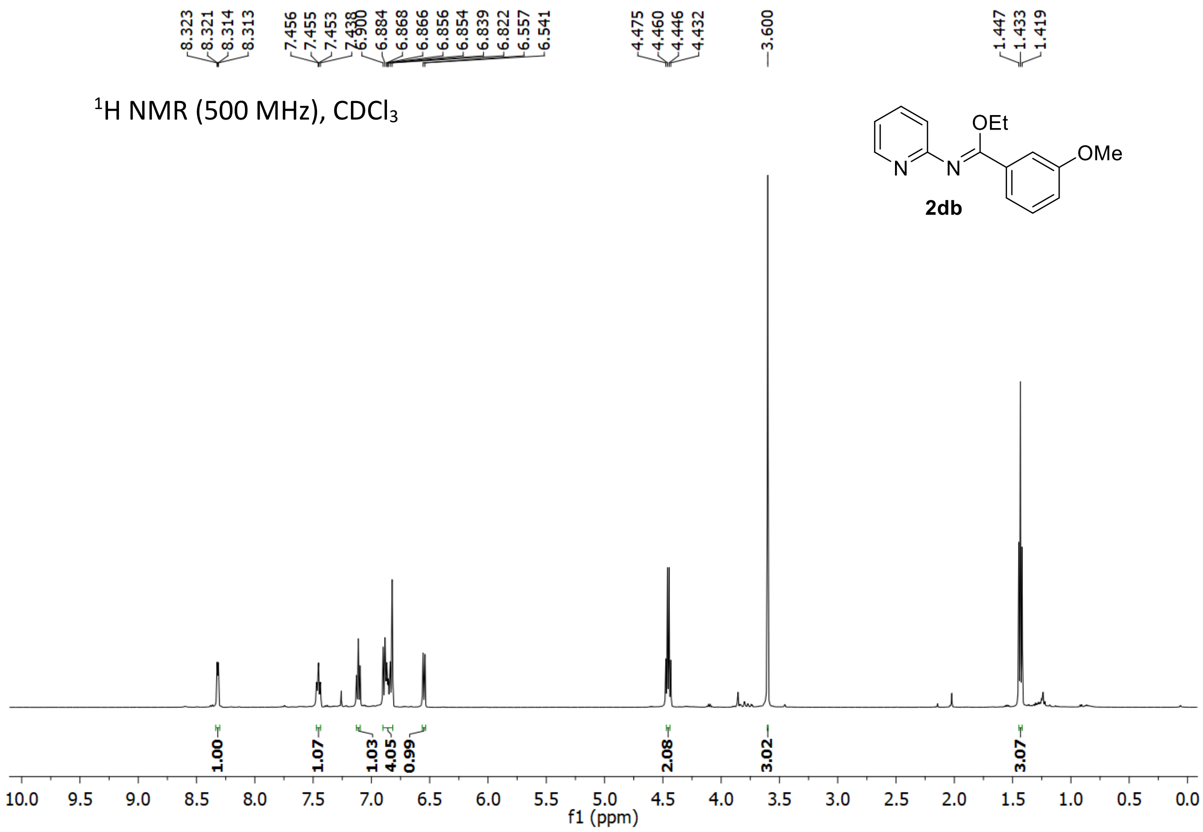

8.323
8.321
8.314
8.313
7.456
7.455
7.453
7.438
6.900
6.884
6.868
6.866
6.856
6.854
6.839
6.822
6.557
6.541
4.475
4.460
4.446
4.432
3.600
1.447
1.433
1.419
¹H NMR (500 MHz), CDCl₃
OEt
OMe
N
N
2db
1.00
1.07
1.03
4.05
0.99
2.08
3.02
3.07
f1 (ppm)

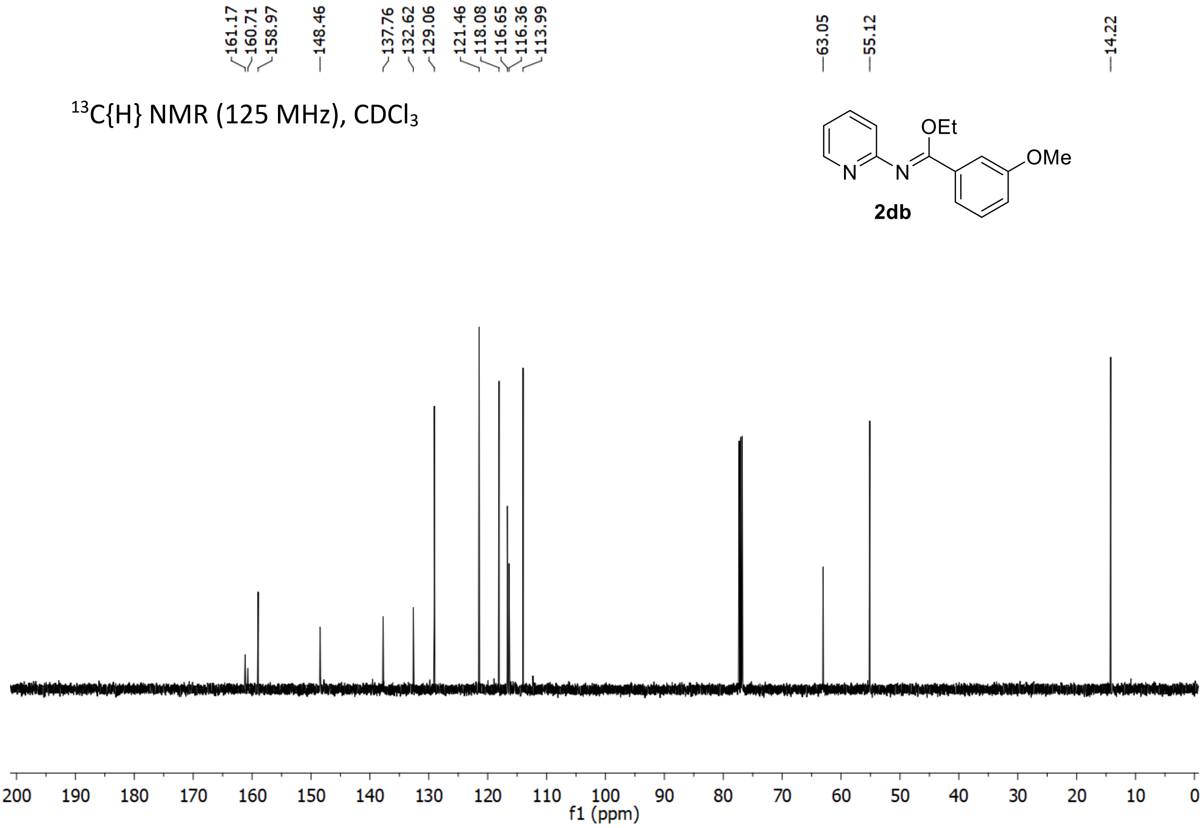

161.17
160.71
158.97
148.46
137.76
132.62
129.06
121.46
118.08
116.65
116.36
113.99
63.05
55.12
14.22
¹³C{H} NMR (125 MHz), CDCl₃
OEt
OMe
N
N
2db
f1 (ppm)

^{1}H NMR (500 MHz), $CDCl_3$

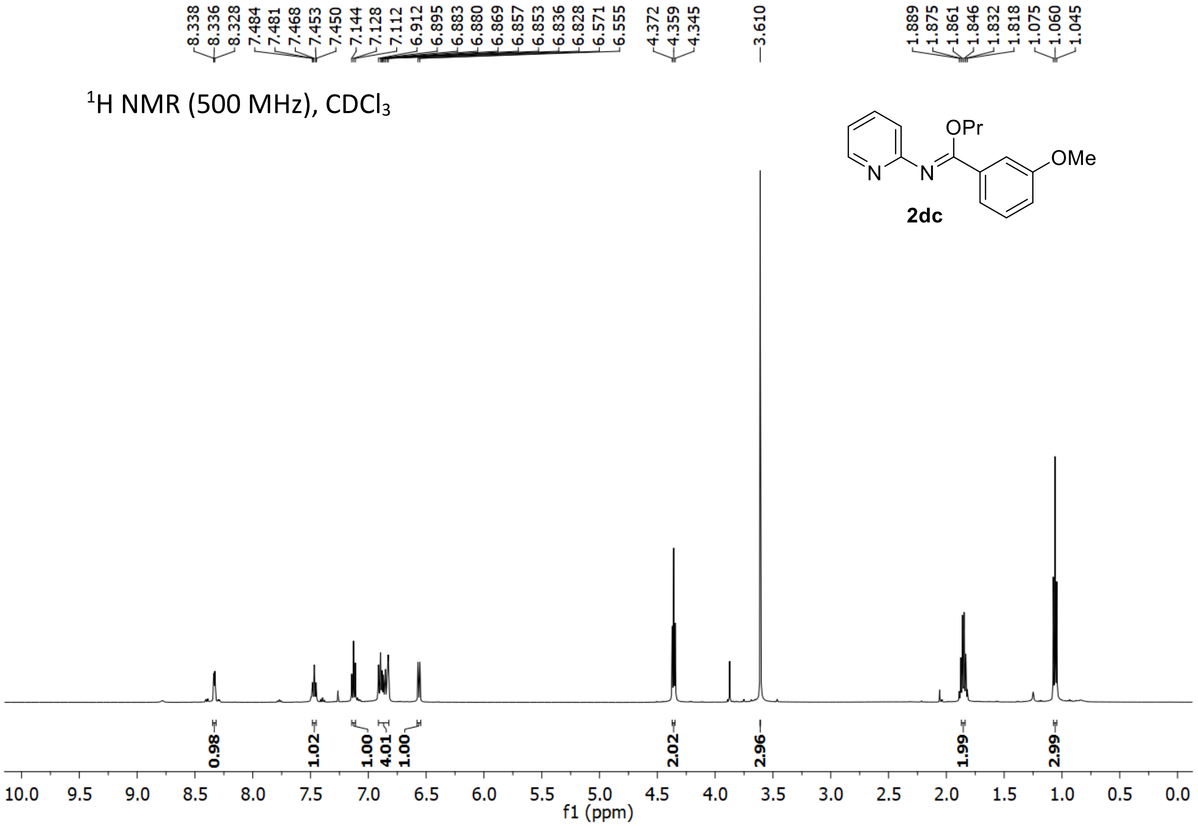

$^{13}C\{H\}$ NMR (125 MHz), $CDCl_3$

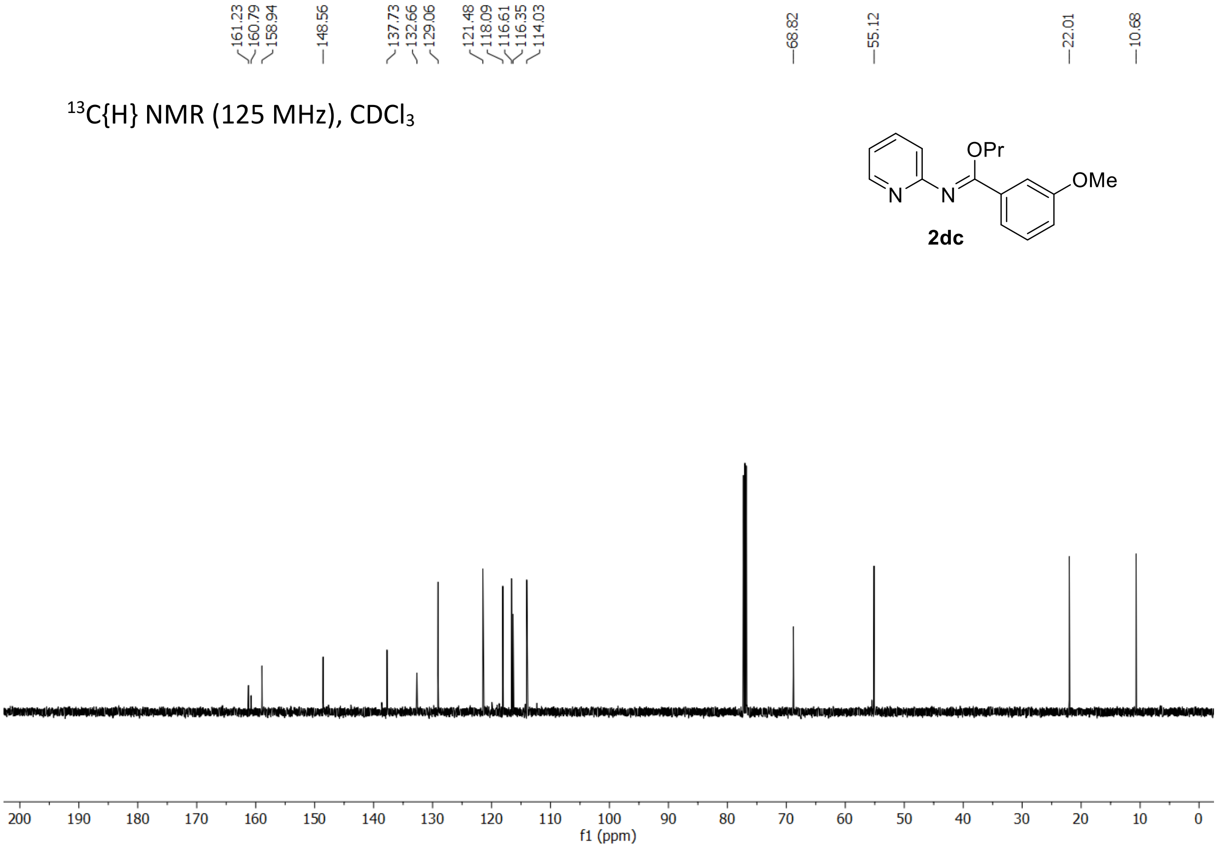

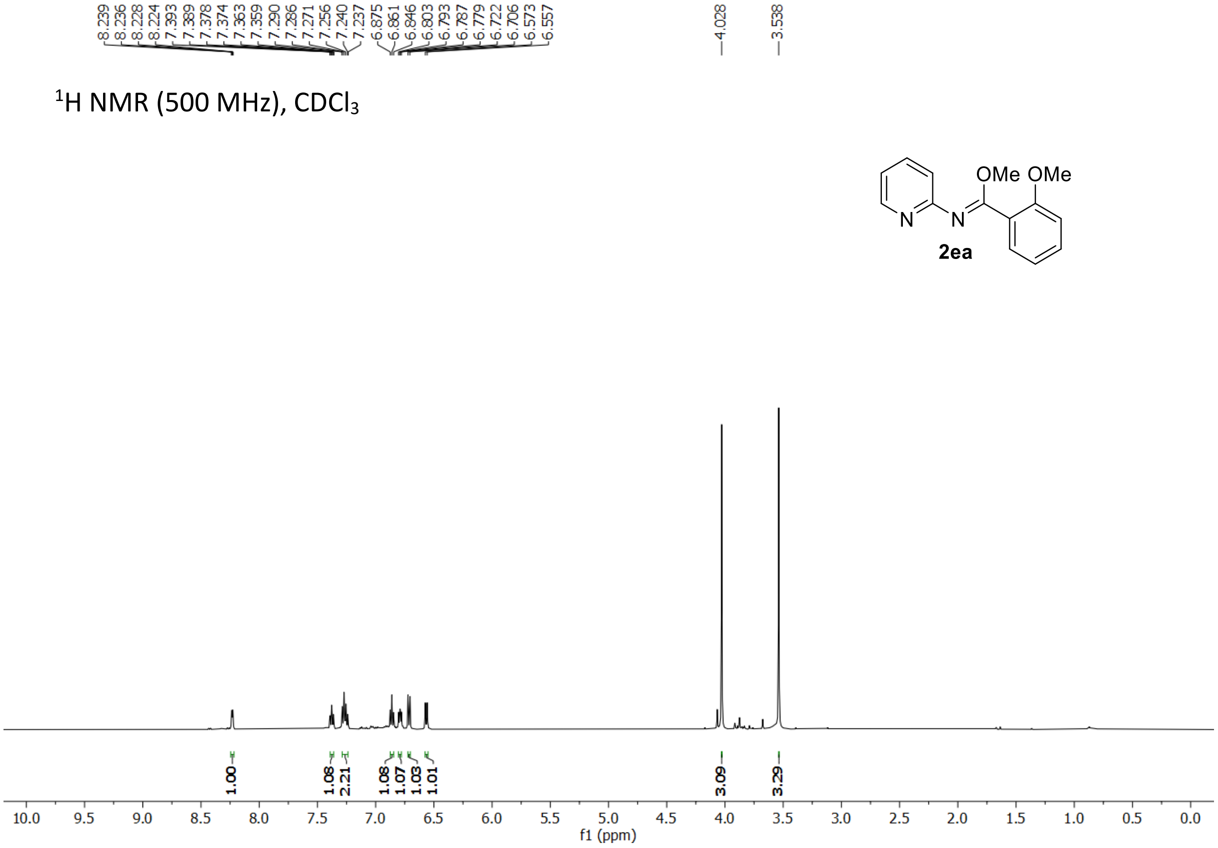
8.239 8.236 8.228 8.224 7.393 7.389 7.378 7.374 7.363 7.359 7.290 7.286 7.271 7.256 7.240 7.237 6.875 6.861 6.846 6.803 6.793 6.787 6.779 6.722 6.706 6.573 6.557
4.028
3.538
^{1}H NMR (500 MHz), $CDCl_3$
OMe OMe
N
N
2ea
1.00
1.08
2.21
1.08
1.07
1.03
1.01
3.09
3.29
f1 (ppm)

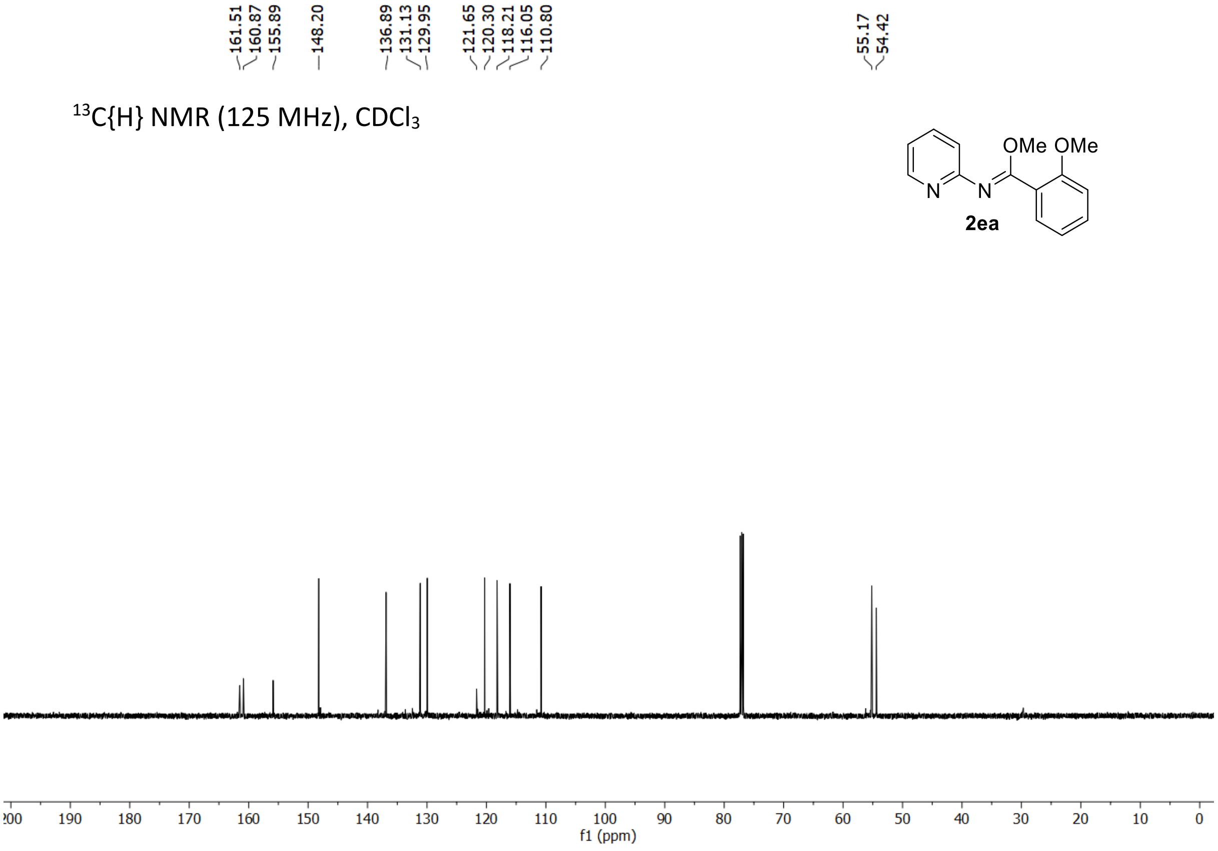
161.51 160.87 155.89
148.20
136.89 131.13 129.95
121.65 120.30 118.21 116.05 110.80
55.17 54.42
^{13}C{H} NMR (125 MHz), $CDCl_3$
OMe OMe
N
N
2ea
f1 (ppm)

^{1}H NMR (500 MHz), $CDCl_3$

2fa

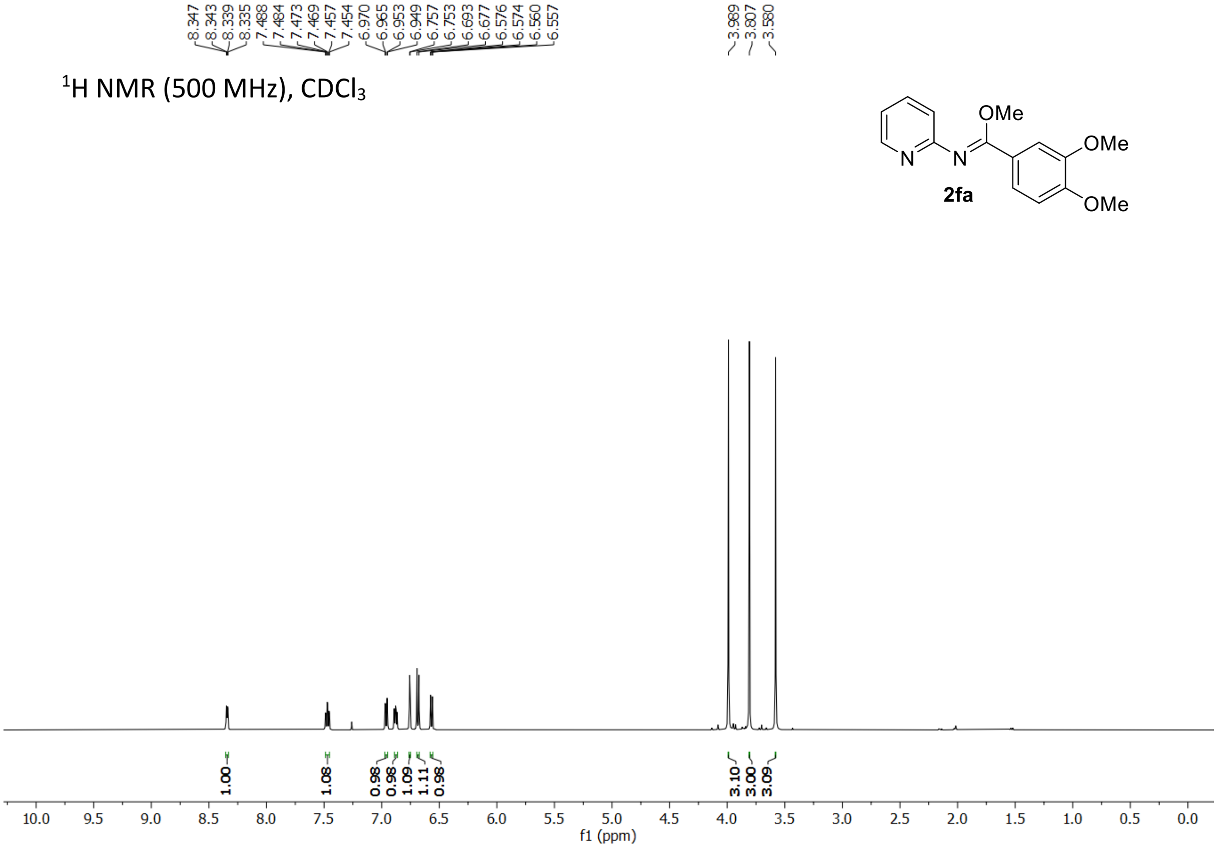

^{13}C{H} NMR (125 MHz), $CDCl_3$

2fa

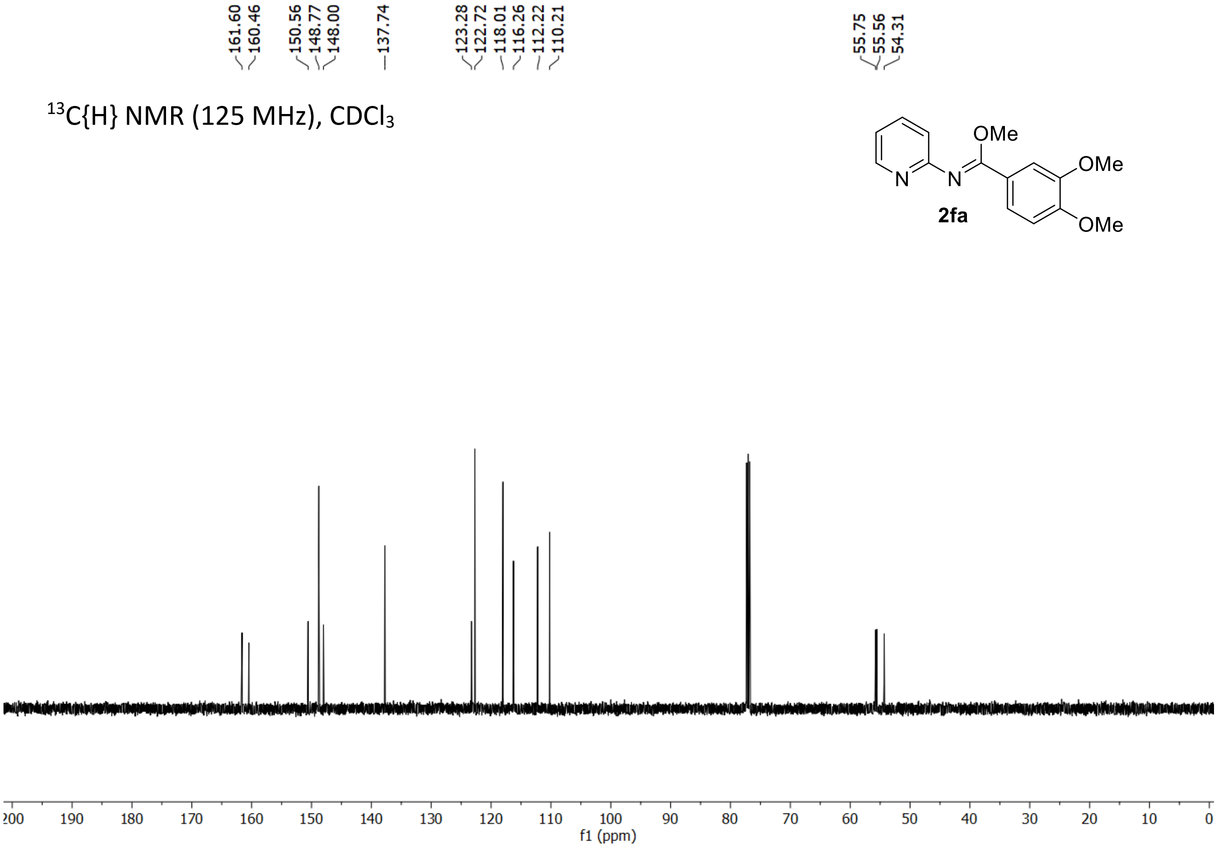

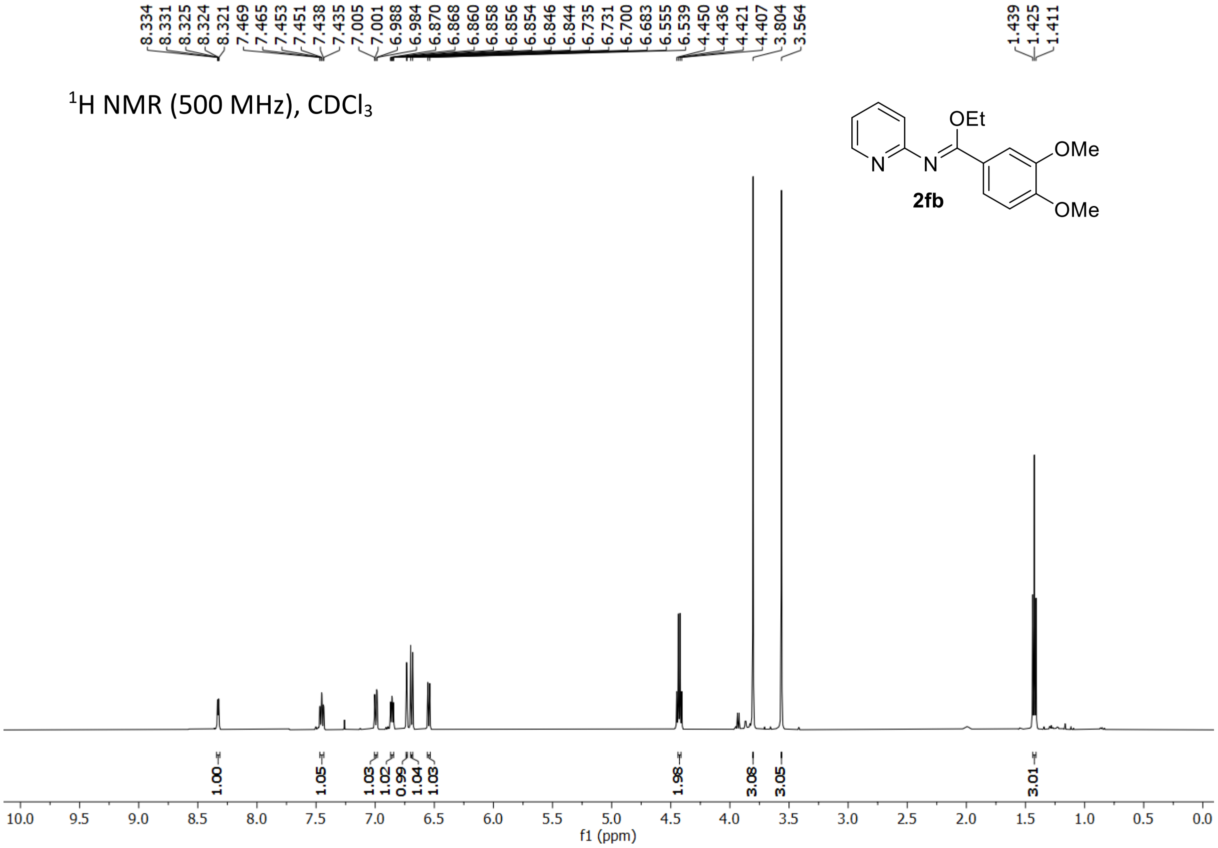
8.334 8.331 8.325 8.324 8.321 7.469 7.465 7.453 7.451 7.438 7.435 7.005 7.001 6.988 6.984 6.870 6.868 6.860 6.858 6.856 6.854 6.846 6.844 6.735 6.731 6.700 6.683 6.555 6.539 4.450 4.436 4.421 4.407 3.804 3.564
1.439 1.425 1.411
^{1}H NMR (500 MHz), $CDCl_3$
OEt
OMe
OMe
N
N
2fb
1.00 1.05 1.03 1.02 0.99 1.04 1.03 1.98 3.08 3.05 3.01
f1 (ppm)

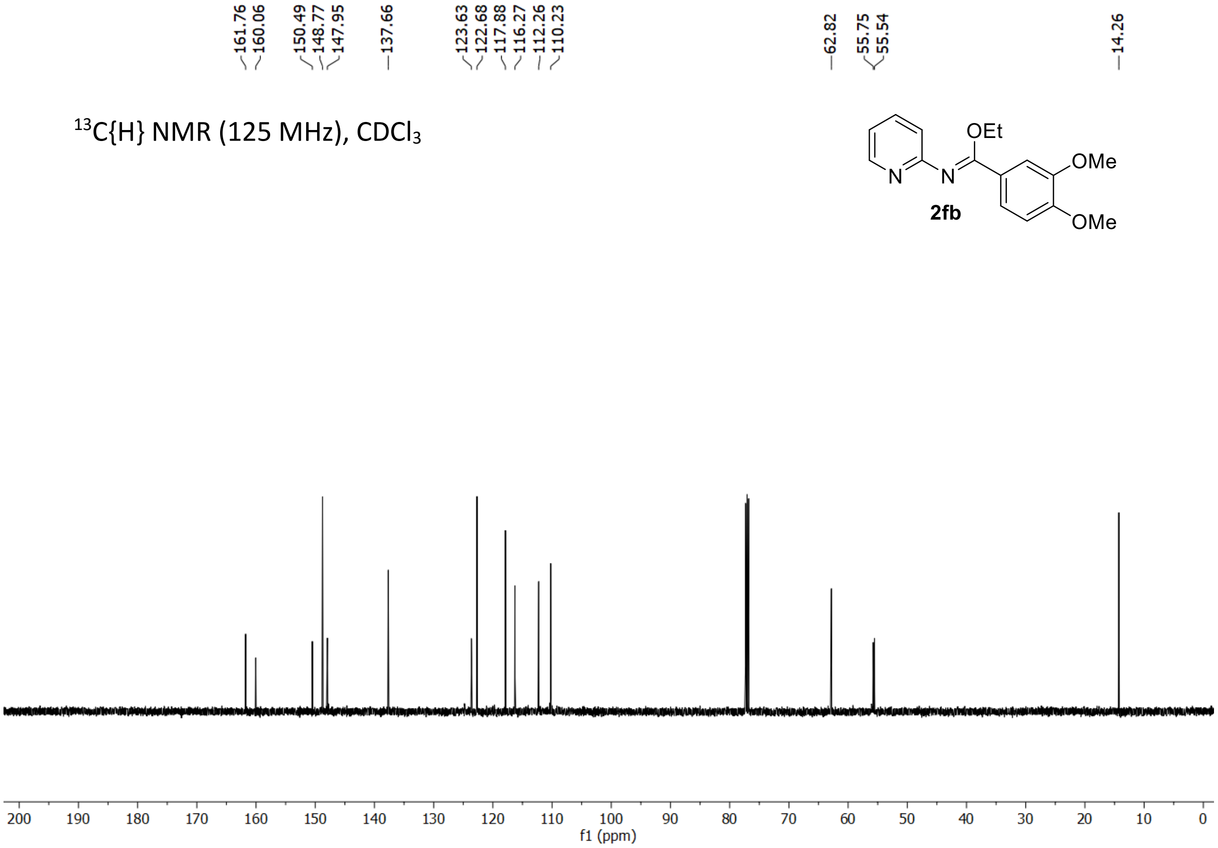
161.76 160.06 150.49 148.77 147.95 137.66 123.63 122.68 117.88 116.27 112.26 110.23 62.82 55.75 55.54 14.26
^{13}C{H} NMR (125 MHz), $CDCl_3$
OEt
OMe
OMe
N
N
2fb
f1 (ppm)

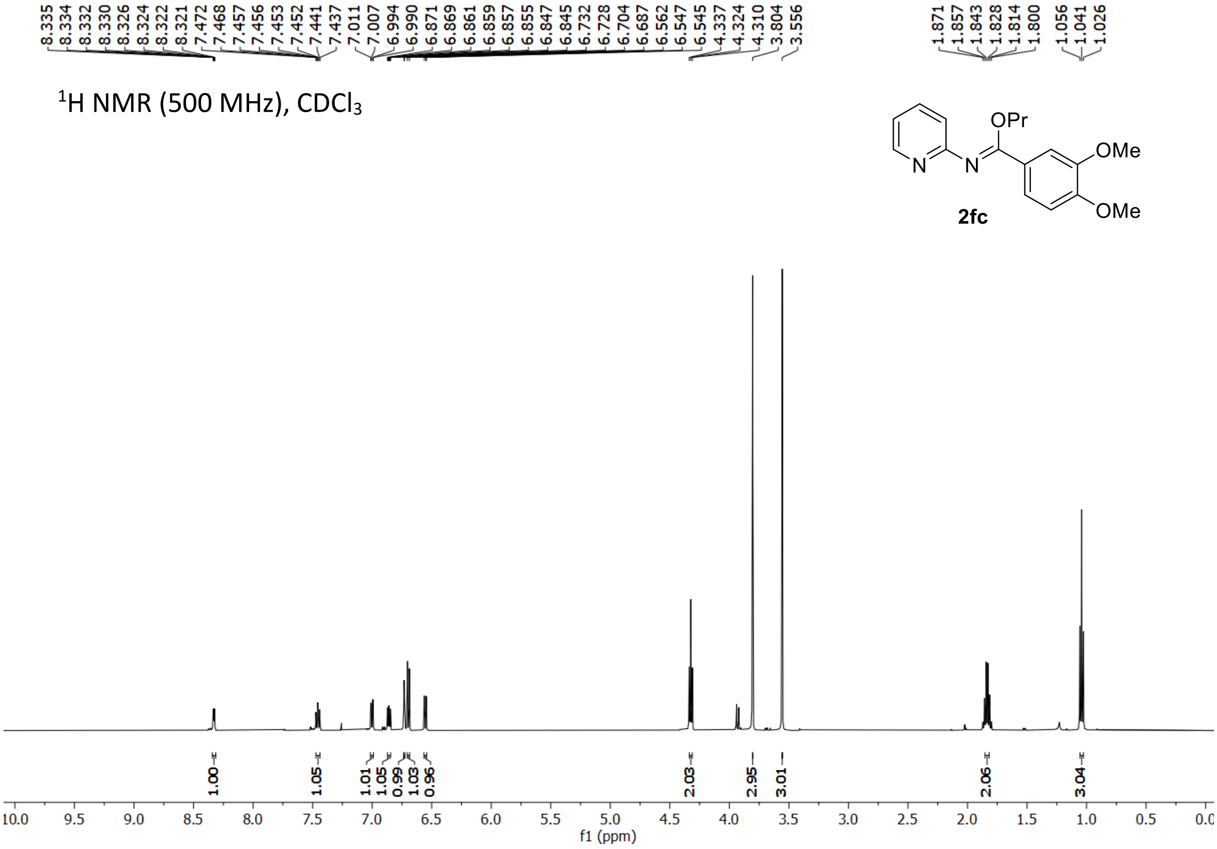

8.335 8.334 8.332 8.330 8.326 8.324 8.322 8.321 7.472 7.468 7.457 7.456 7.453 7.452 7.441 7.437 7.011 7.007 6.994 6.990 6.871 6.869 6.861 6.859 6.857 6.855 6.847 6.845 6.732 6.728 6.704 6.687 6.562 6.547 6.545 4.337 4.324 4.310 3.804 3.556
1.871 1.857 1.843 1.828 1.814 1.800 1.056 1.041 1.026
^{1}H NMR (500 MHz), CDCl$_3$
OPr
OMe
OMe
N
N
2fc
1.00 1.05 1.01 1.05 0.99 1.03 0.96 2.03 2.95 3.01 2.06 3.04
10.0 9.5 9.0 8.5 8.0 7.5 7.0 6.5 6.0 5.5 5.0 4.5 4.0 3.5 3.0 2.5 2.0 1.5 1.0 0.5 0.0
f1 (ppm)

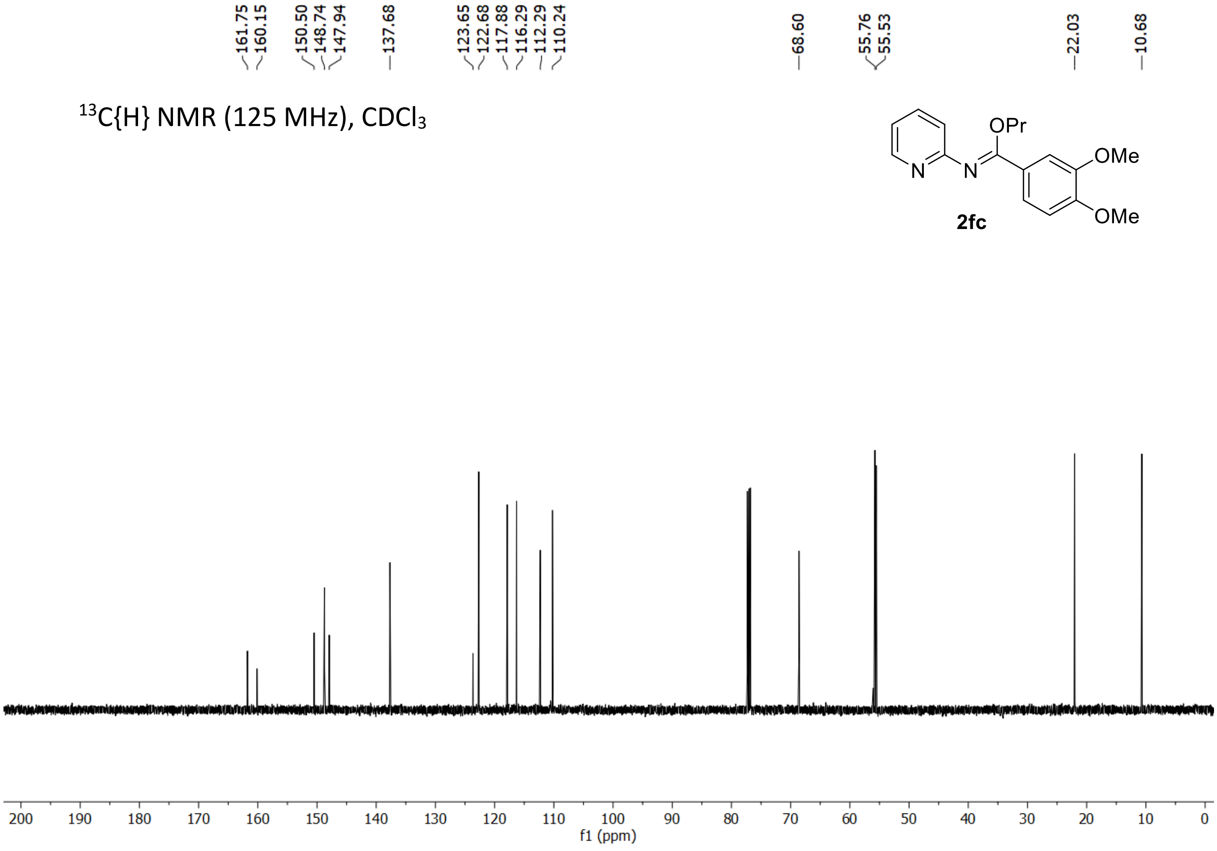

161.75 160.15 150.50 148.74 147.94 137.68 123.65 122.68 117.88 116.29 112.29 110.24 68.60 55.76 55.53 22.03 10.68
^{13}C{H} NMR (125 MHz), CDCl$_3$
OPr
OMe
OMe
N
N
2fc
200 190 180 170 160 150 140 130 120 110 100 90 80 70 60 50 40 30 20 10 0
f1 (ppm)

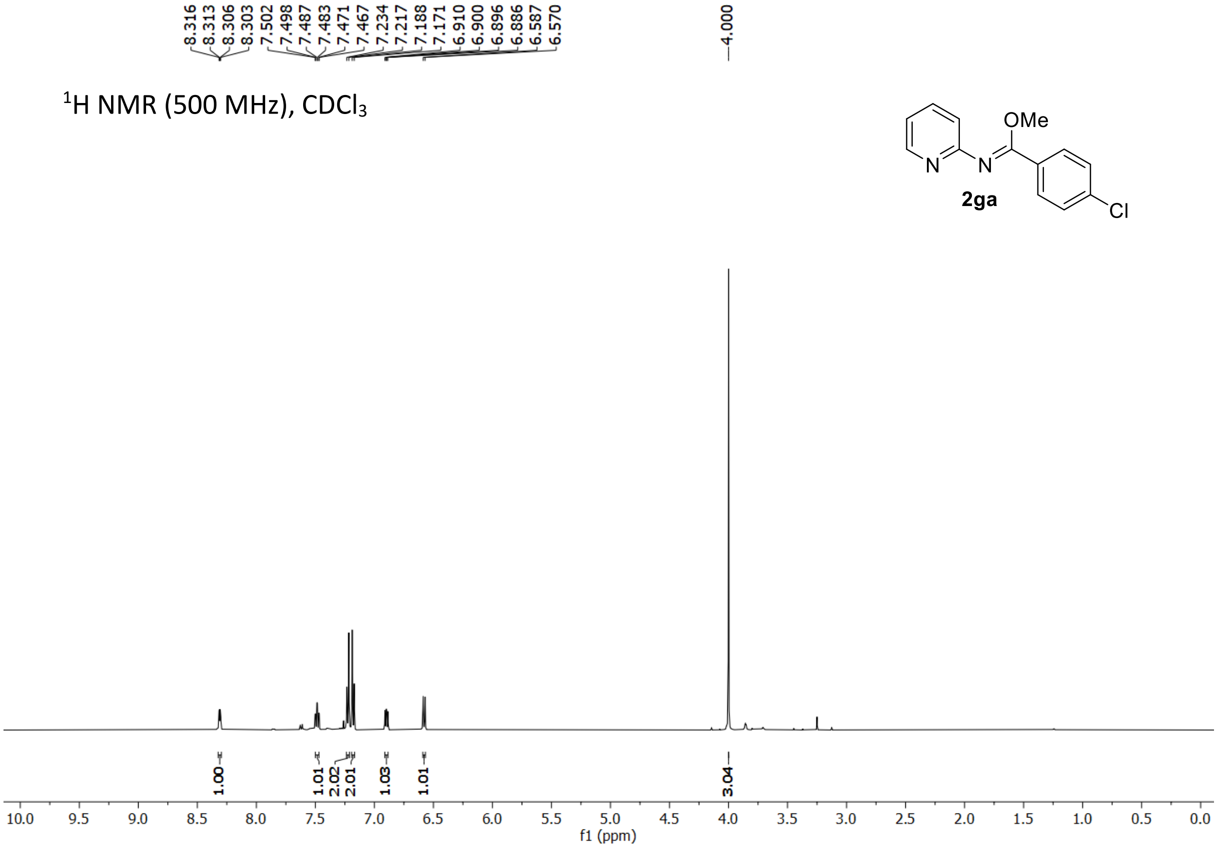
8.316
8.313
8.306
8.303
7.502
7.498
7.487
7.483
7.471
7.467
7.234
7.217
7.188
7.171
6.910
6.900
6.896
6.886
6.587
6.570
4.000
¹H NMR (500 MHz), CDCl₃
OMe
N
N
2ga
Cl
1.00
1.01
2.02
2.01
1.03
1.01
3.04
f1 (ppm)

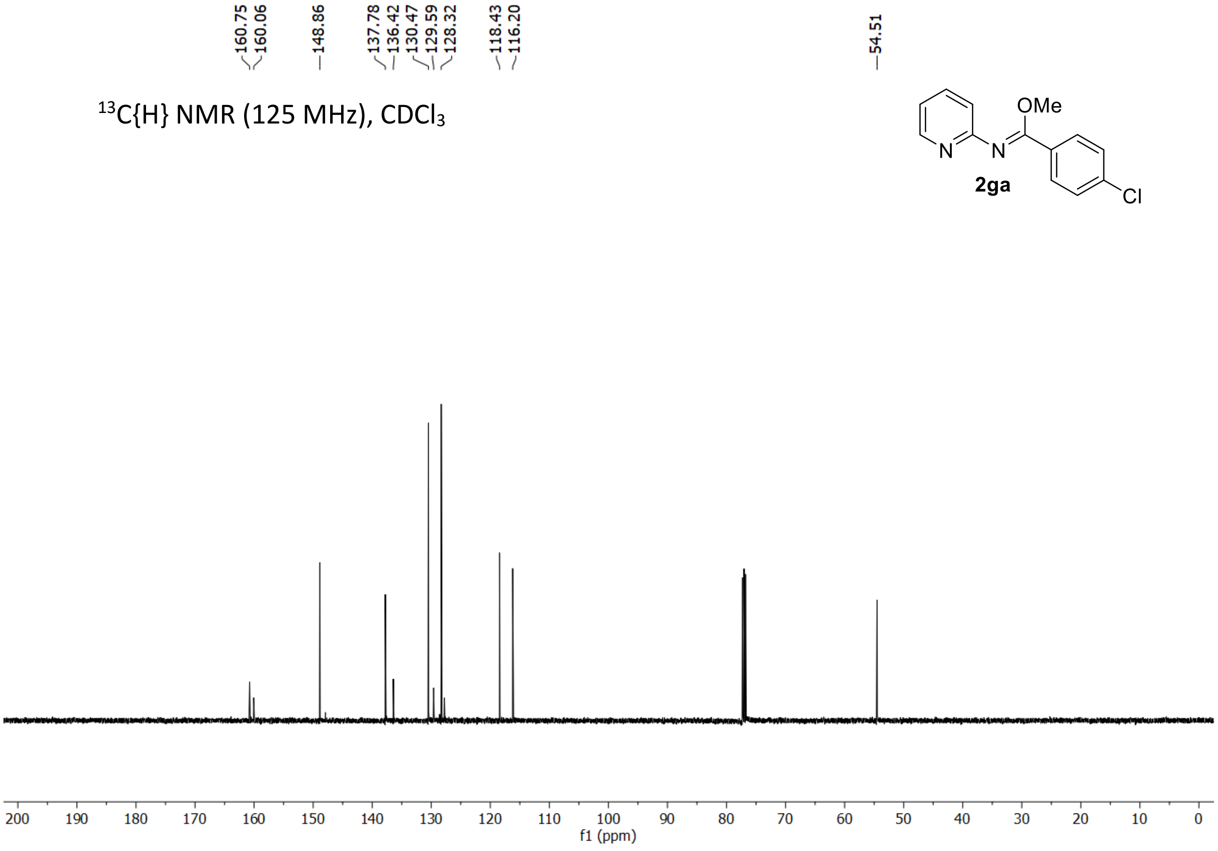
160.75
160.06
148.86
137.78
136.42
130.47
129.59
128.32
118.43
116.20
54.51
¹³C{H} NMR (125 MHz), CDCl₃
OMe
N
N
2ga
Cl
f1 (ppm)

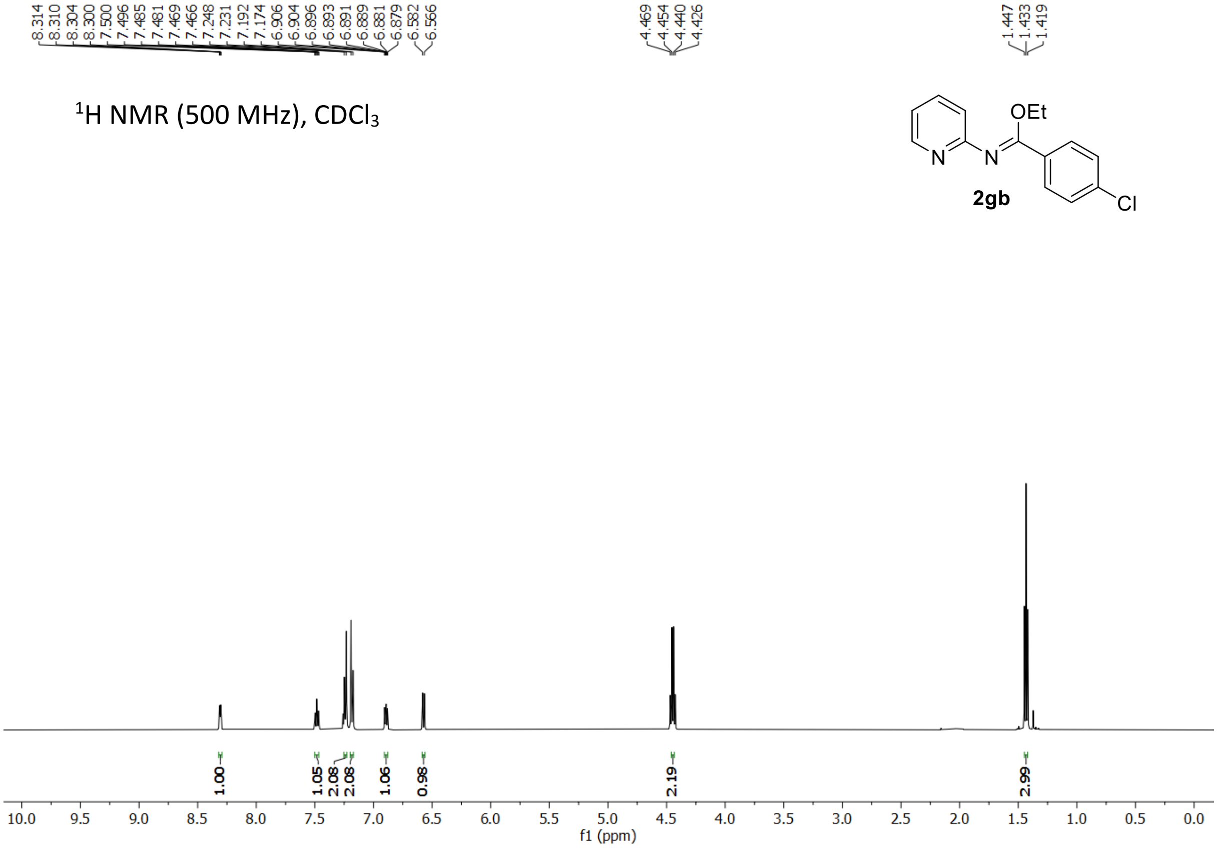
8.314
8.310
8.304
8.300
7.500
7.496
7.485
7.481
7.469
7.466
7.248
7.231
7.192
7.174
6.906
6.904
6.896
6.893
6.891
6.889
6.881
6.879
6.582
6.566
4.469
4.454
4.440
4.426
1.447
1.433
1.419
^{1}H NMR (500 MHz), $CDCl_3$
OEt
N
N
2gb
Cl
1.00
1.05
2.08
2.08
1.06
0.98
2.19
2.99
10.0 9.5 9.0 8.5 8.0 7.5 7.0 6.5 6.0 5.5 5.0 4.5 4.0 3.5 3.0 2.5 2.0 1.5 1.0 0.5 0.0
f1 (ppm)

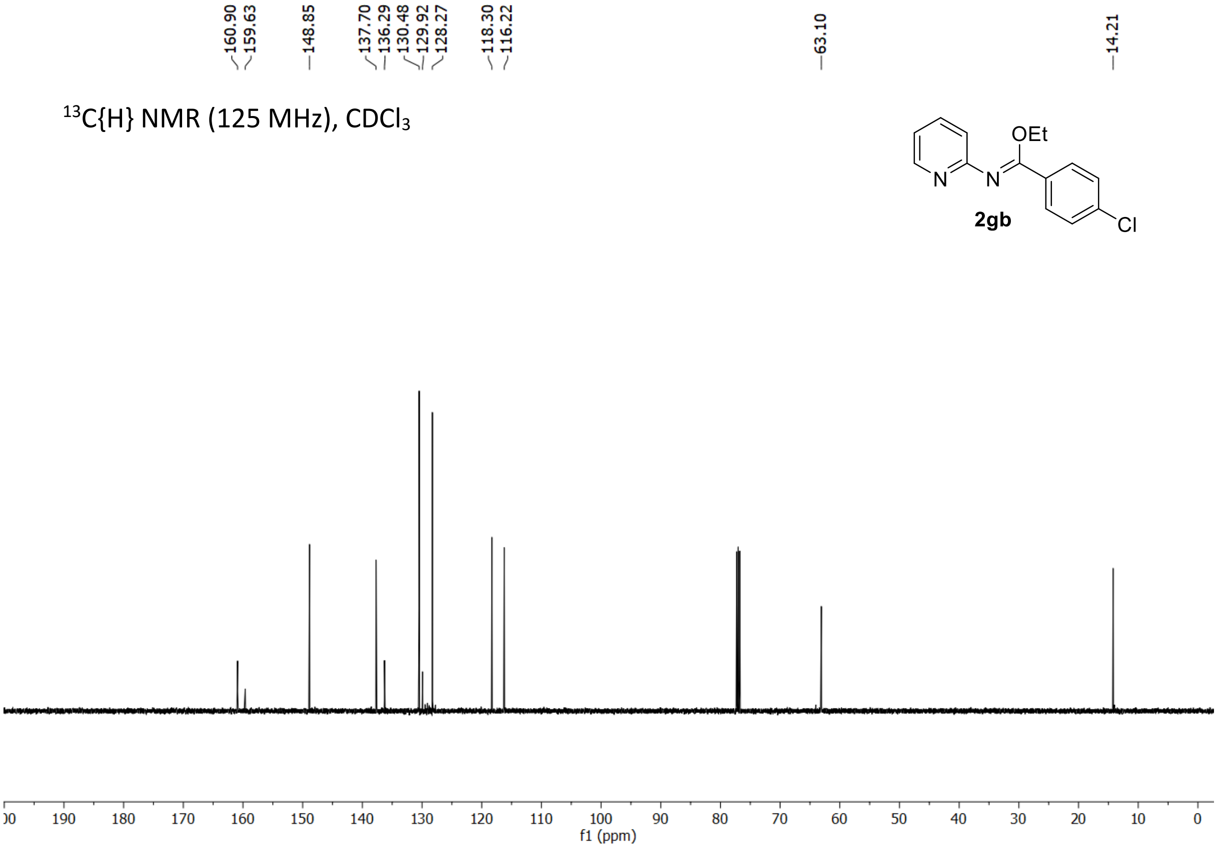
160.90
159.63
148.85
137.70
136.29
130.48
129.92
128.27
118.30
116.22
63.10
14.21
^{13}C{H} NMR (125 MHz), $CDCl_3$
OEt
N
N
2gb
Cl
190 180 170 160 150 140 130 120 110 100 90 80 70 60 50 40 30 20 10 0
f1 (ppm)

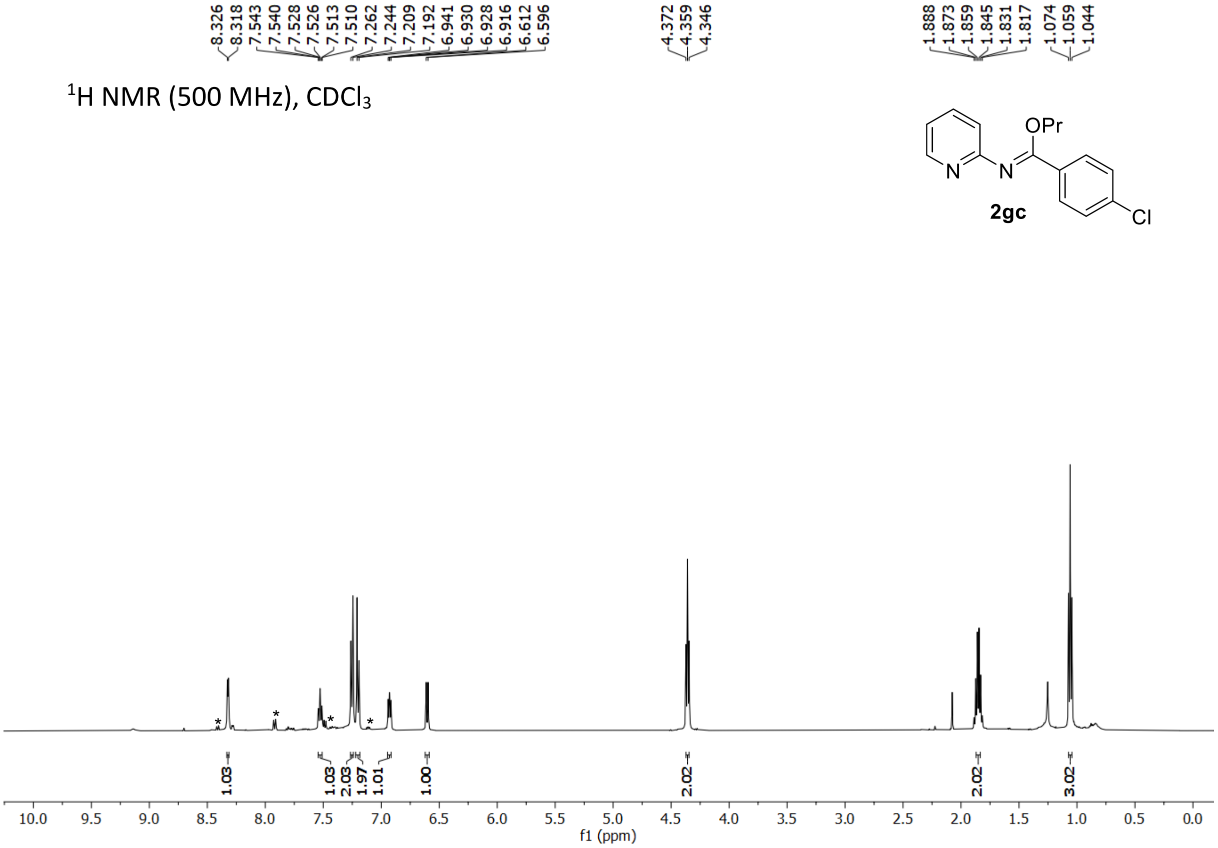

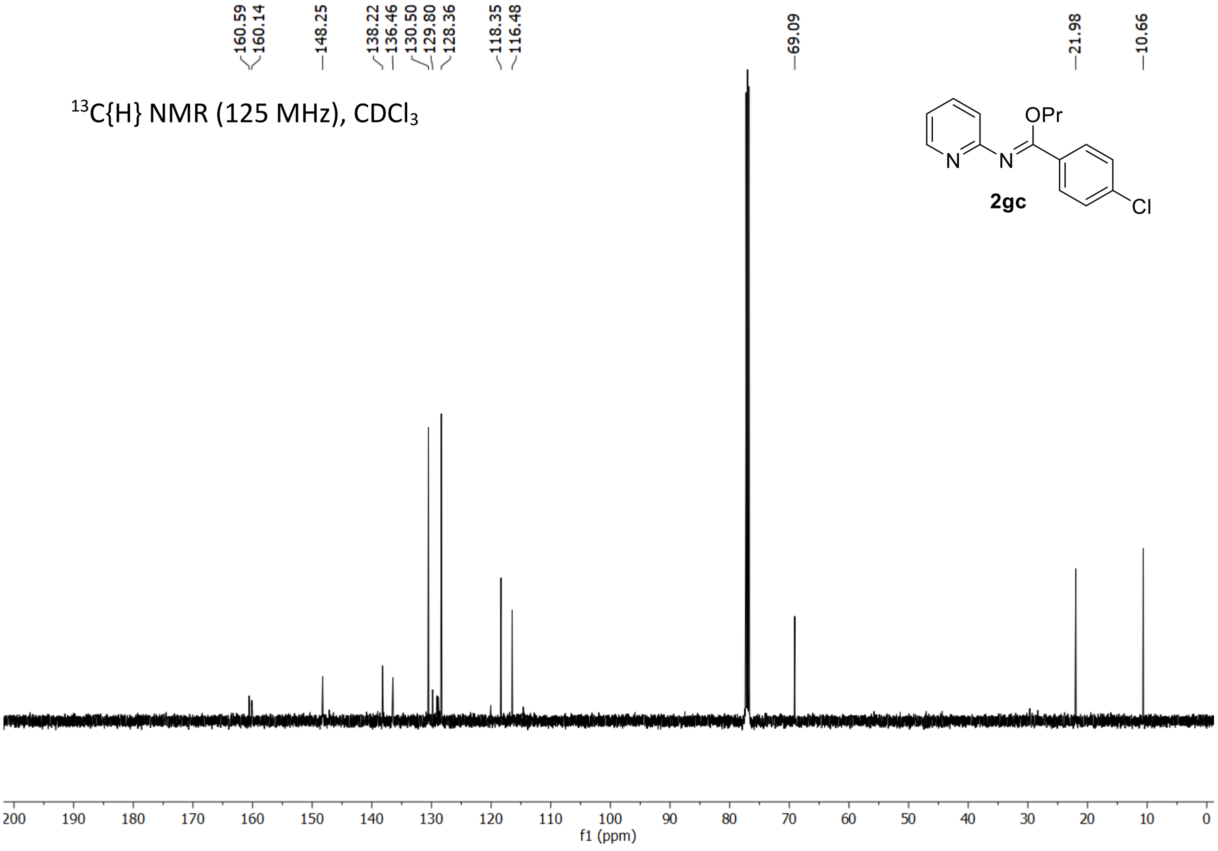

*corresponding amide

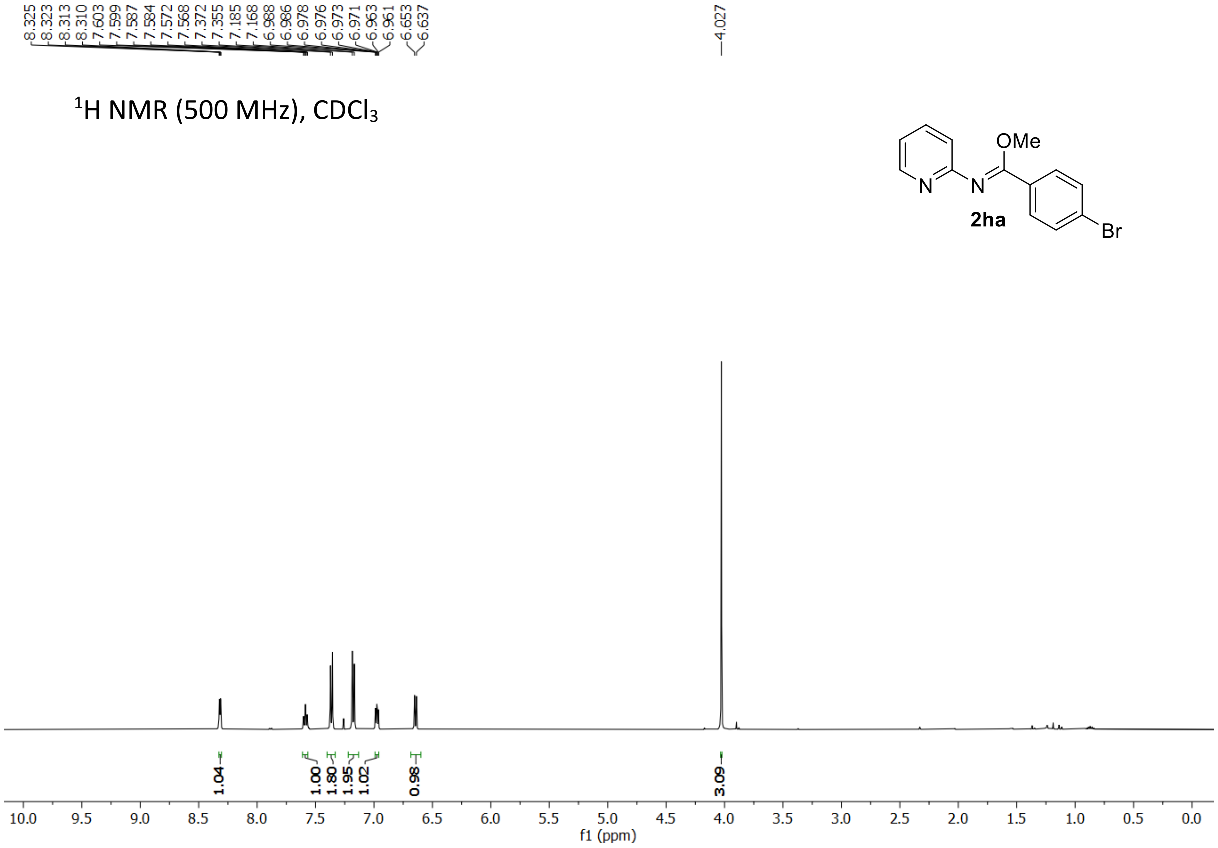

8.325
8.323
8.313
8.310
7.603
7.599
7.587
7.584
7.572
7.568
7.372
7.355
7.185
7.168
6.988
6.986
6.978
6.976
6.973
6.971
6.963
6.961
6.653
6.637
4.027
^{1}H NMR (500 MHz), CDCl$_3$
OMe
N
N
2ha
Br
1.04
1.00
1.80
1.95
1.02
0.98
3.09
f1 (ppm)

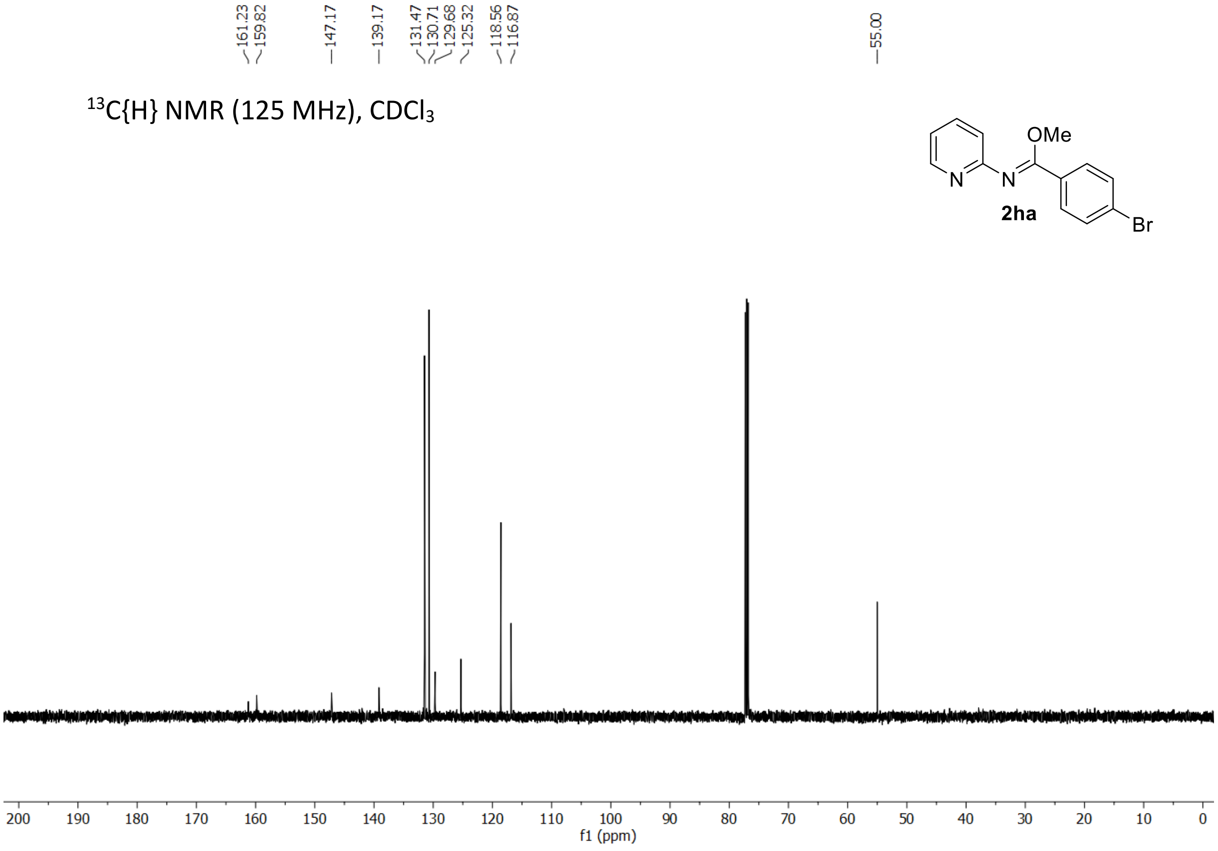

161.23
159.82
147.17
139.17
131.47
130.71
129.68
125.32
118.56
116.87
55.00
^{13}C{H} NMR (125 MHz), CDCl$_3$
OMe
N
N
2ha
Br
f1 (ppm)

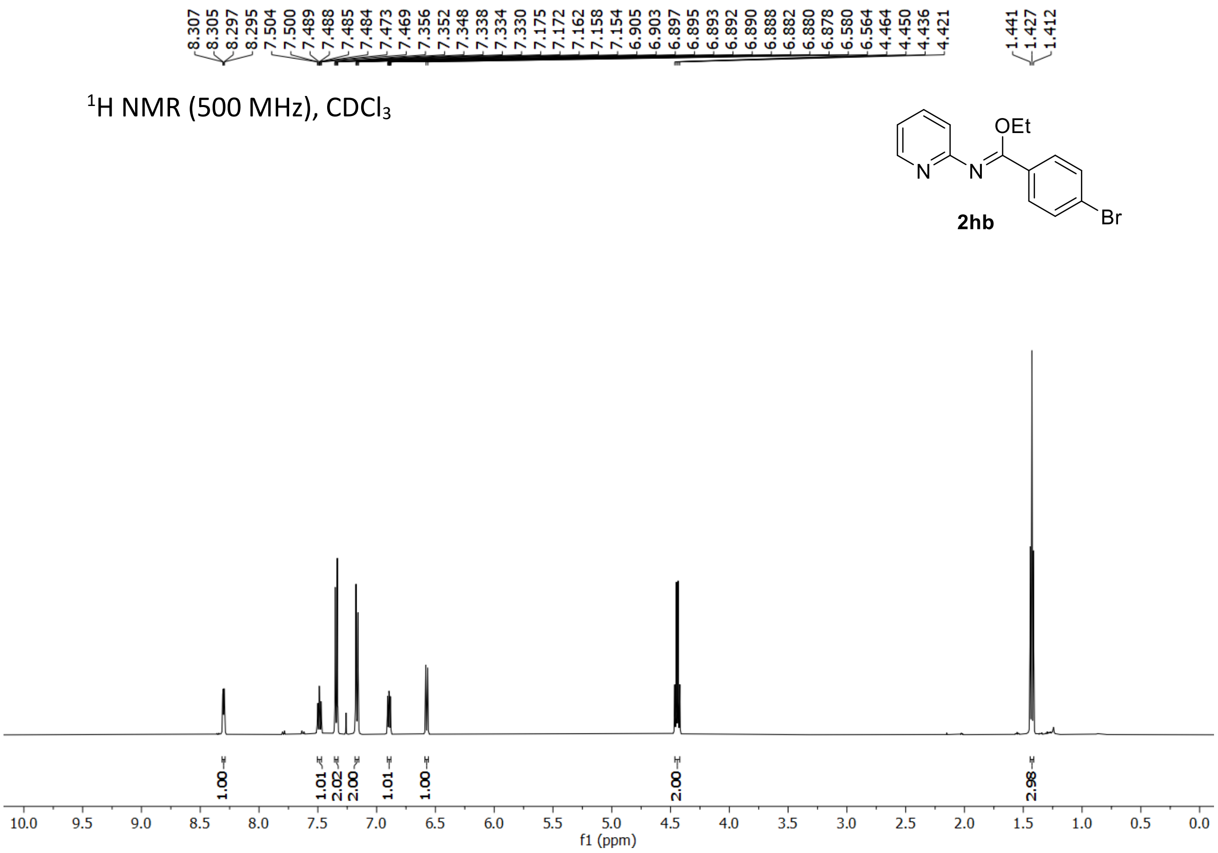
8.307
8.305
8.297
8.295
7.504
7.500
7.489
7.488
7.485
7.484
7.473
7.469
7.356
7.352
7.348
7.338
7.334
7.330
7.175
7.172
7.162
7.158
7.154
6.905
6.903
6.897
6.895
6.893
6.892
6.890
6.888
6.882
6.880
6.878
6.580
6.564
4.464
4.450
4.436
4.421
1.441
1.427
1.412
^{1}H NMR (500 MHz), CDCl$_3$
OEt
N
N
Br
2hb
1.00
1.01
2.02
2.00
1.01
1.00
2.00
2.98
f1 (ppm)

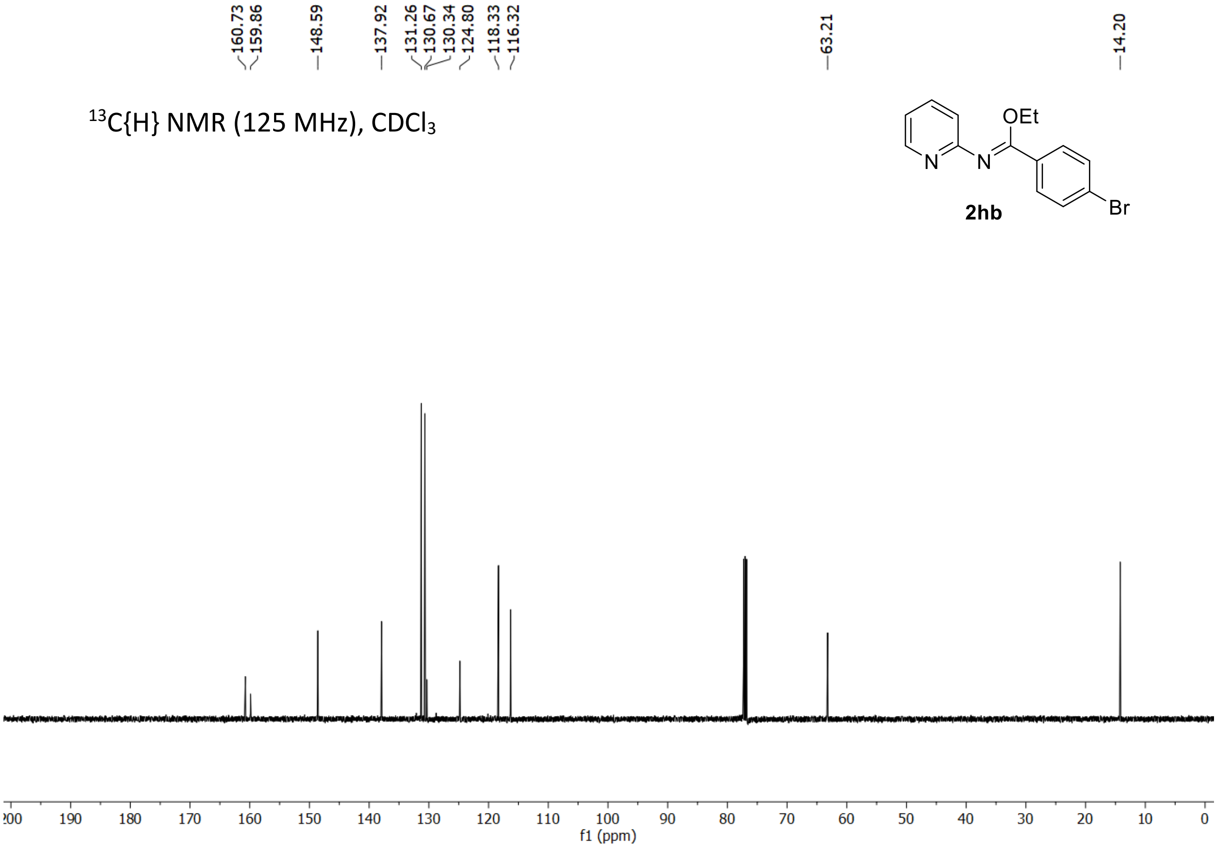
160.73
159.86
148.59
137.92
131.26
130.67
130.34
124.80
118.33
116.32
63.21
14.20
^{13}C{H} NMR (125 MHz), CDCl$_3$
OEt
N
N
Br
2hb
f1 (ppm)

^{1}H NMR (500 MHz), $CDCl_3$

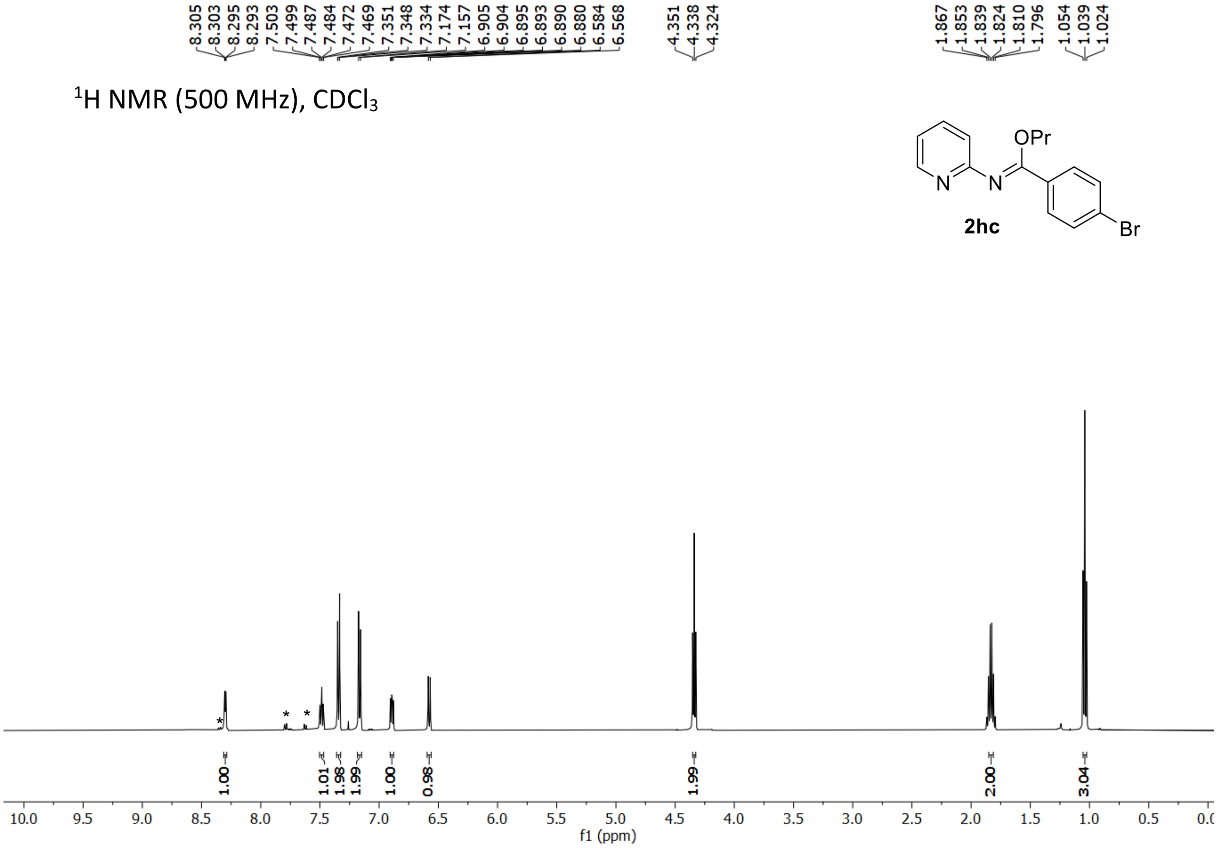

$^{13}C\{H\}$ NMR (125 MHz), $CDCl_3$

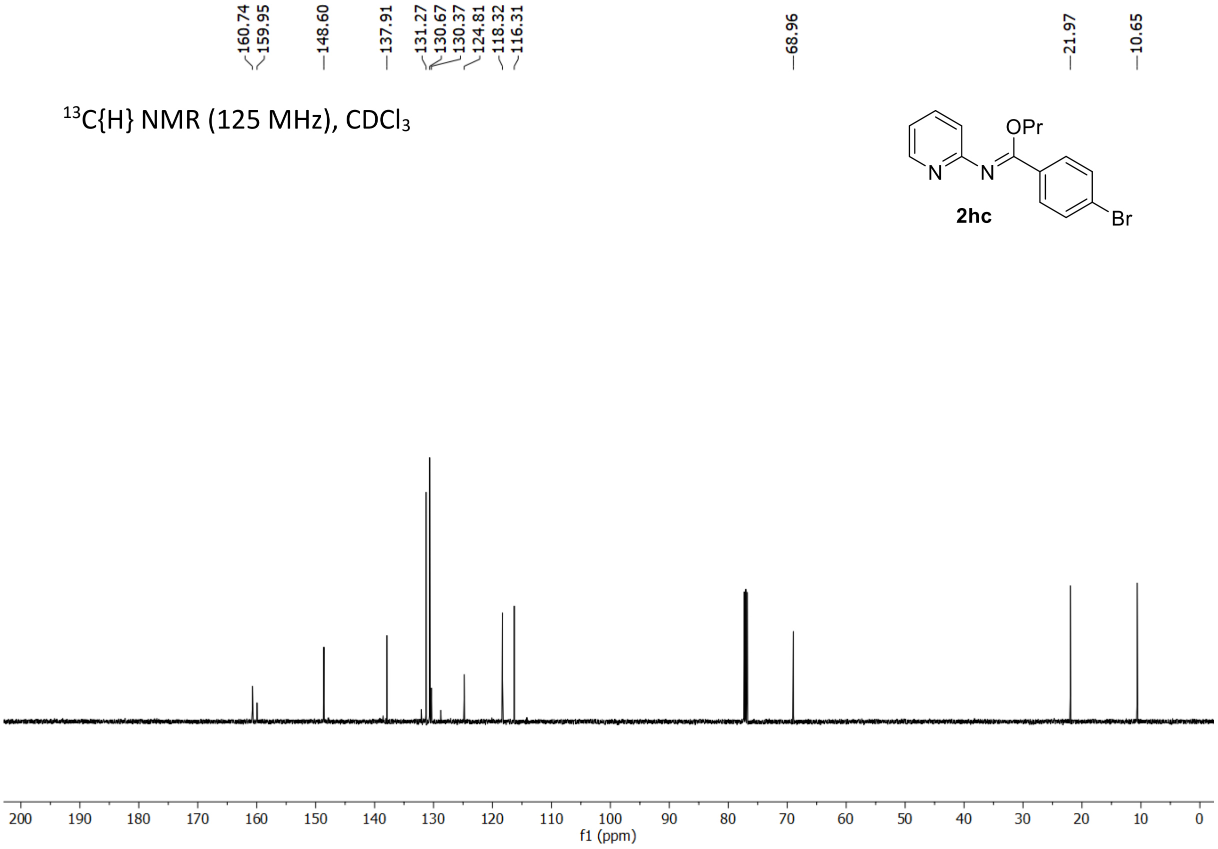

*corresponding amide

^{1}H NMR (500 MHz), $CDCl_3$

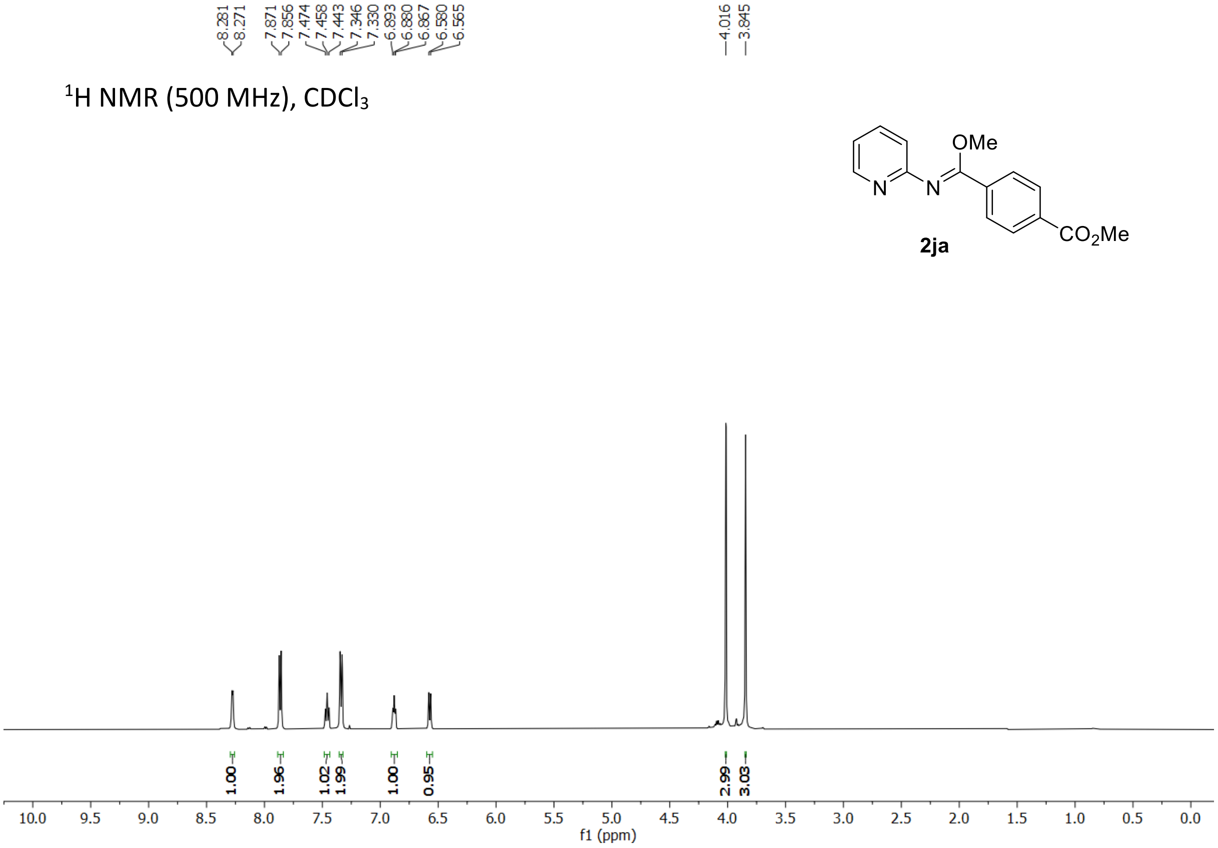

$^{13}C\{H\}$ NMR (125 MHz), $CDCl_3$

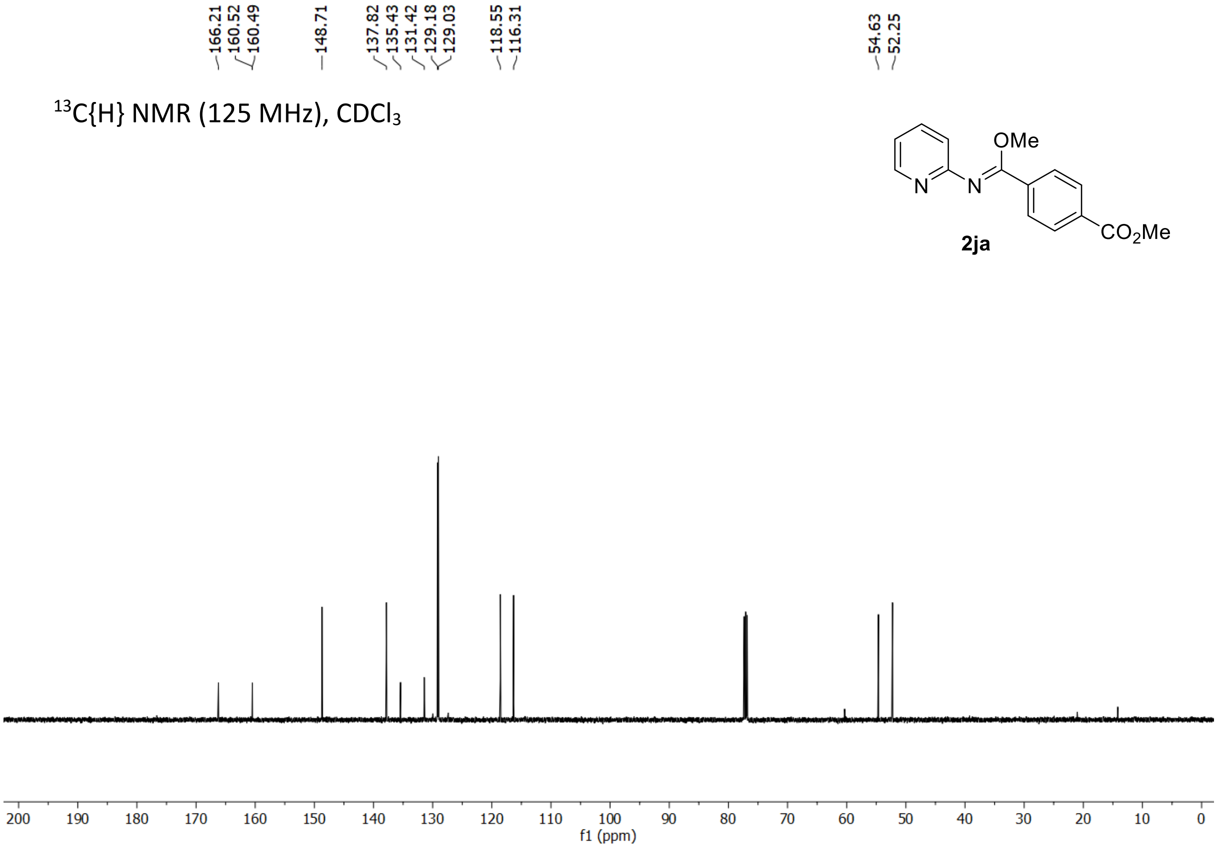

^{1}H NMR (500 MHz), $CDCl_3$

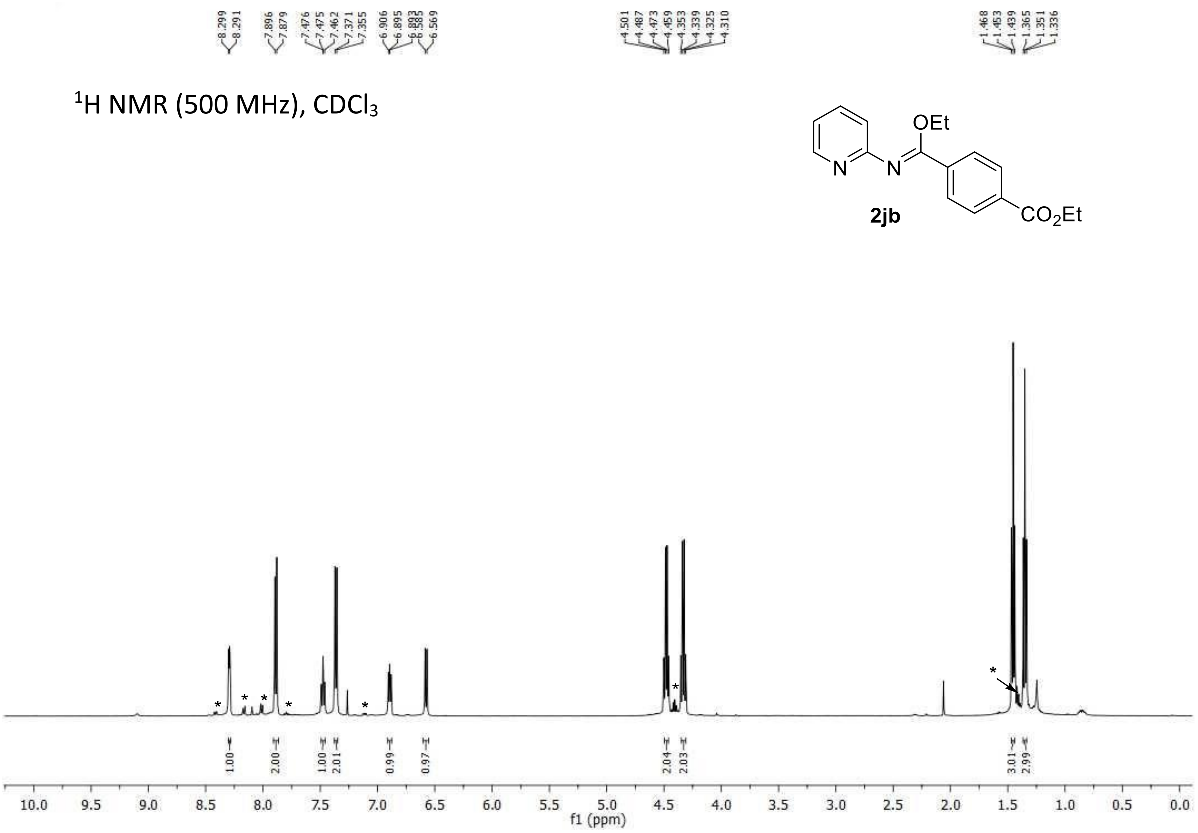

$^{13}C\{H\}$ NMR (125 MHz), $CDCl_3$

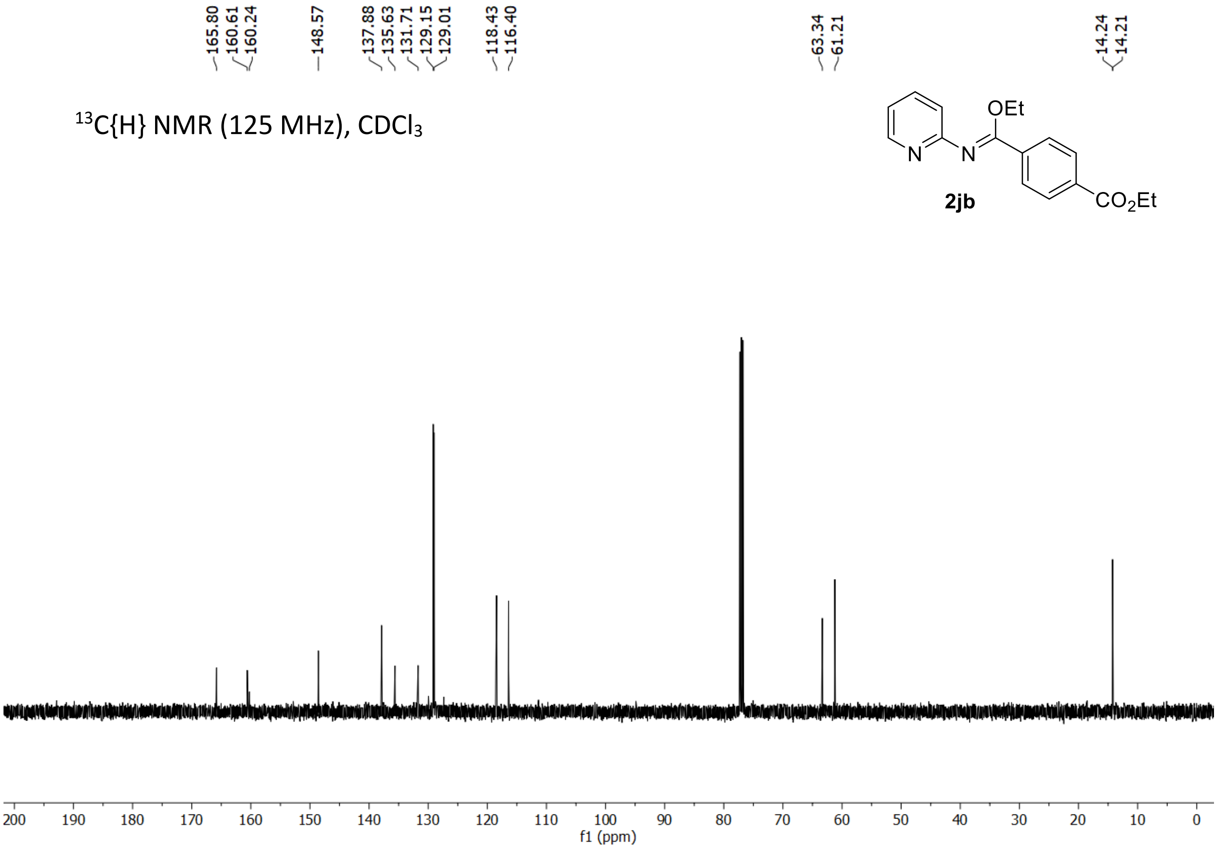

*corresponding amide

^{1}H NMR (500 MHz), $CDCl_3$

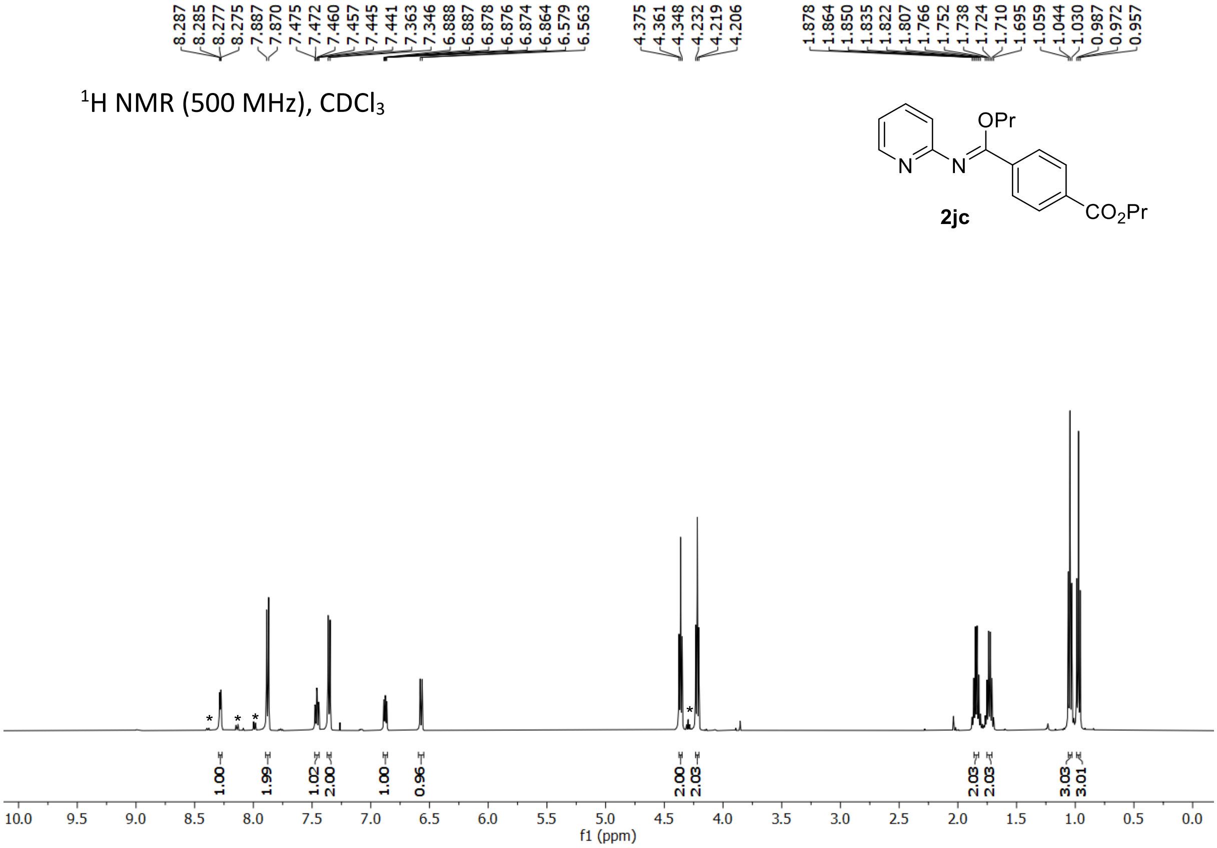

^{13}C{H} NMR (125 MHz), $CDCl_3$

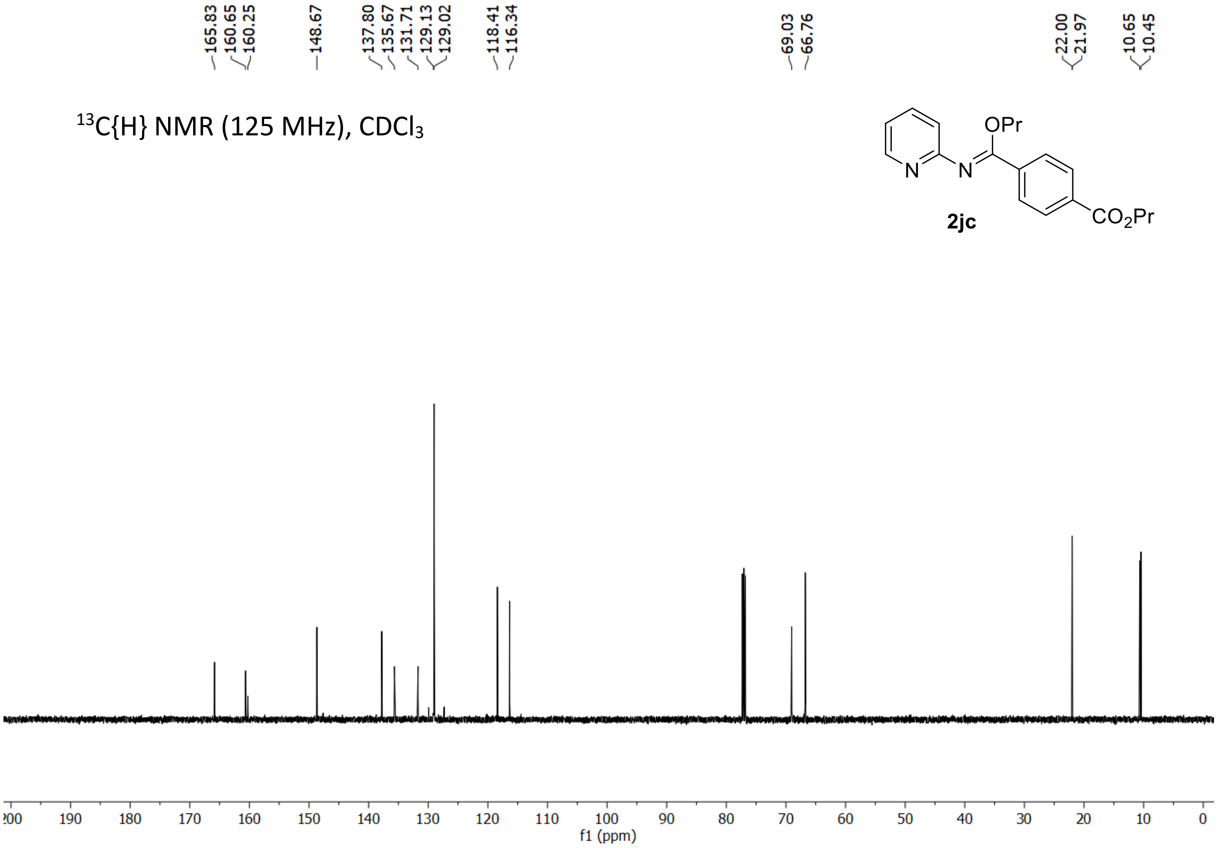

*corresponding amide

^{1}H NMR (500 MHz), $CDCl_3$

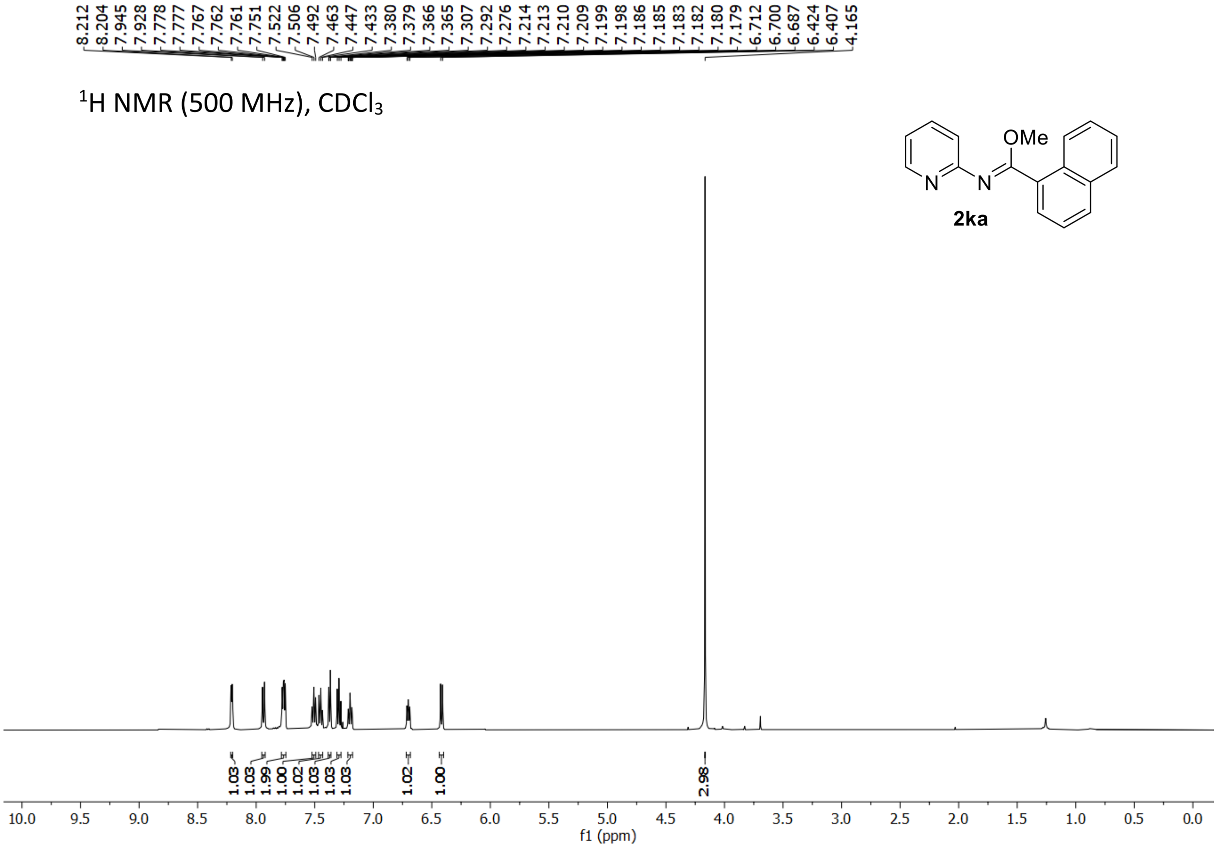

$^{13}C\{H\}$ NMR (125 MHz), $CDCl_3$

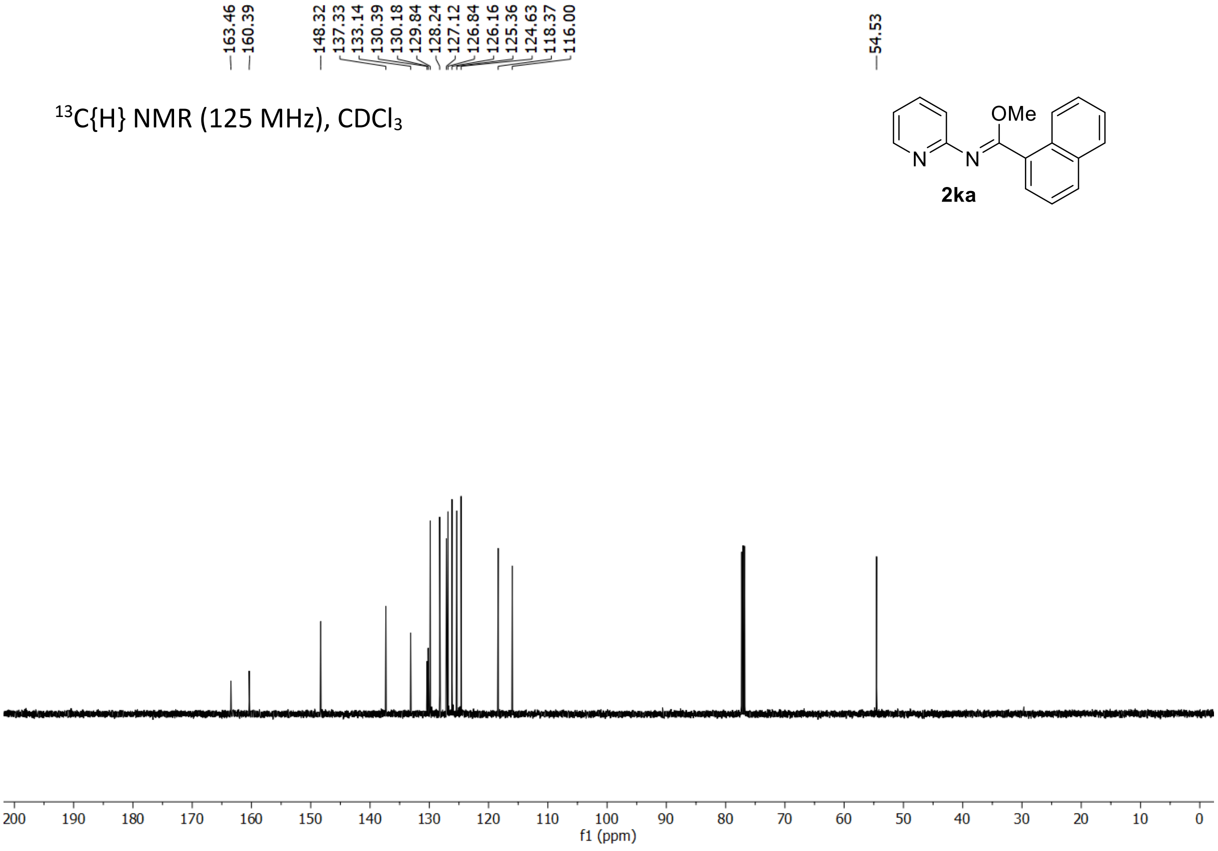

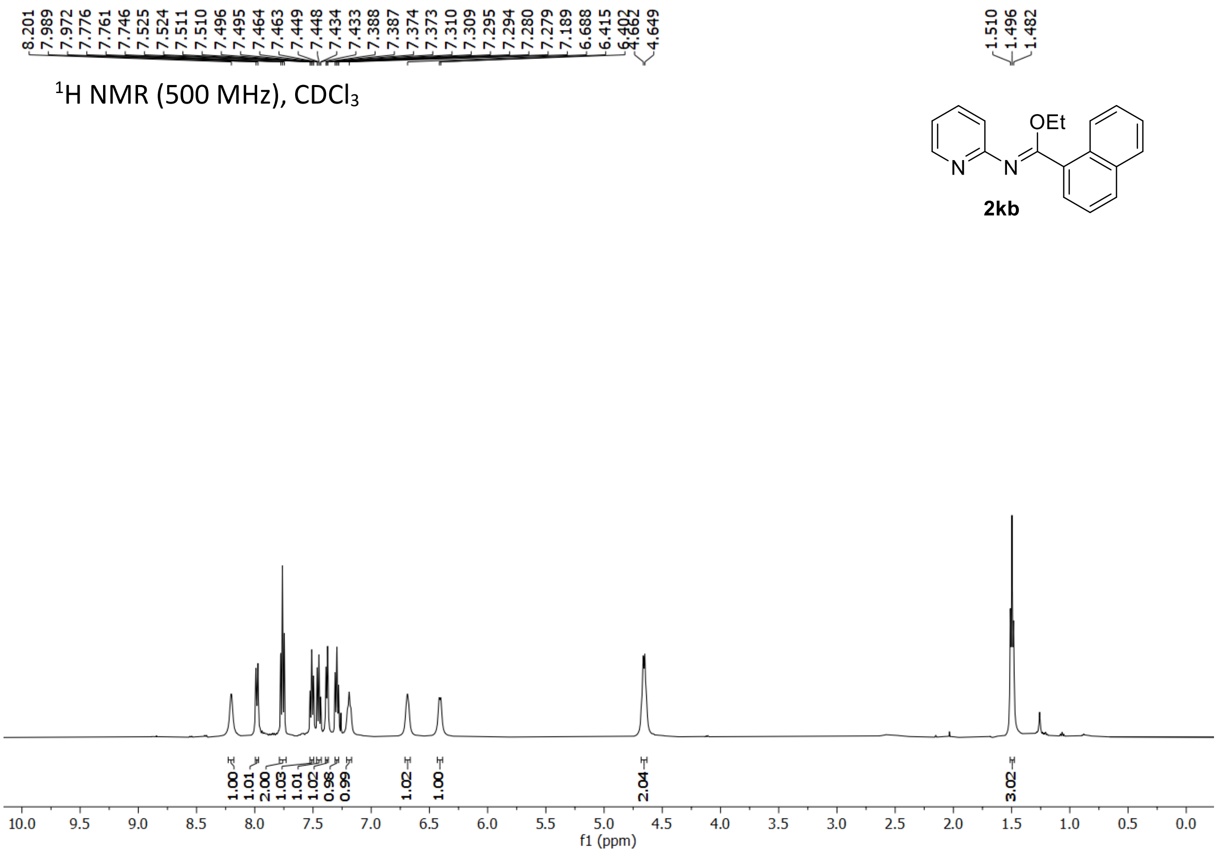
8.201
7.989
7.972
7.776
7.761
7.746
7.525
7.524
7.511
7.510
7.496
7.495
7.464
7.463
7.449
7.448
7.434
7.433
7.388
7.387
7.374
7.373
7.310
7.309
7.295
7.294
7.280
7.279
7.189
6.688
6.415
6.402
4.662
4.649
1.510
1.496
1.482
^{1}H NMR (500 MHz), CDCl$_3$
OEt
N
N
2kb
1.00
1.01
2.00
1.03
1.01
1.02
0.98
0.99
1.02
1.00
2.04
3.02
f1 (ppm)

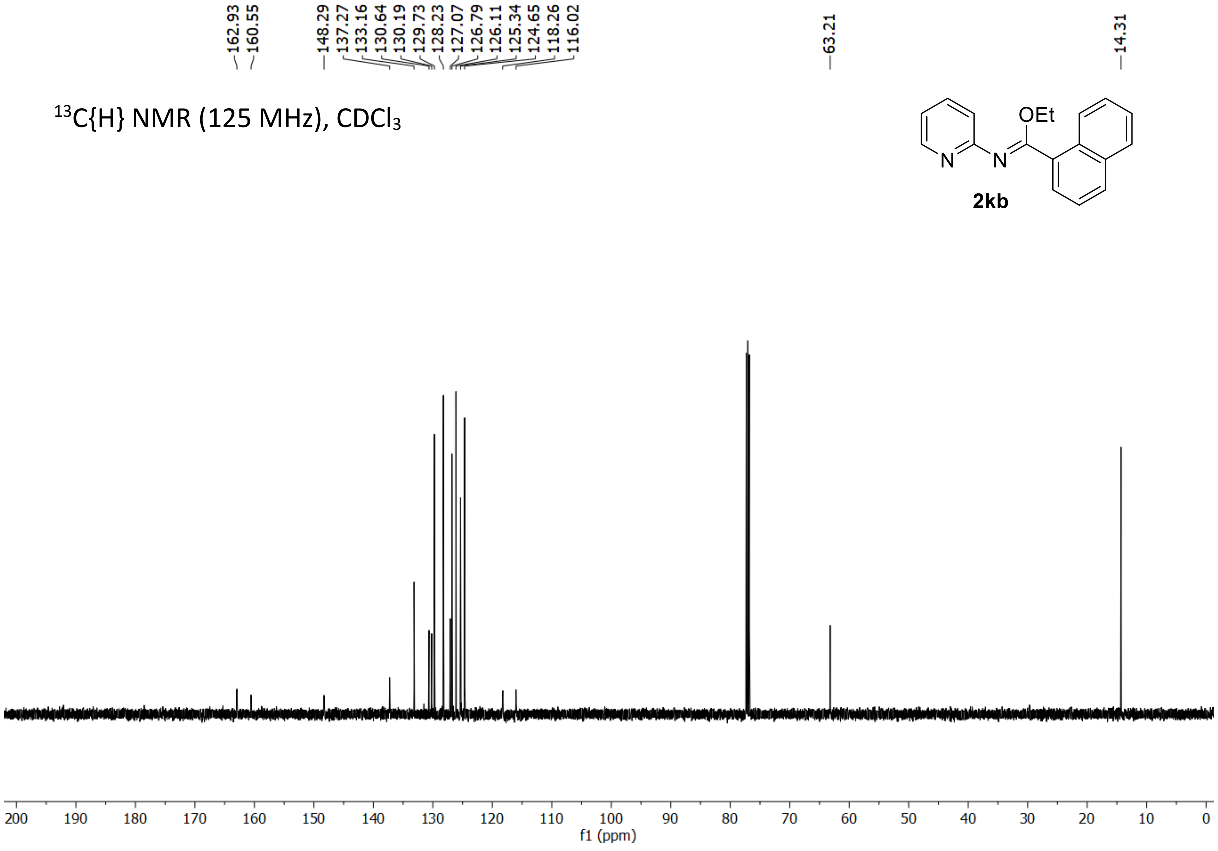
162.93
160.55
148.29
137.27
133.16
130.64
130.19
129.73
128.23
127.07
126.79
126.11
125.34
124.65
118.26
116.02
63.21
14.31
^{13}C{H} NMR (125 MHz), CDCl$_3$
OEt
N
N
2kb
f1 (ppm)

^{1}H NMR (500 MHz), $CDCl_3$

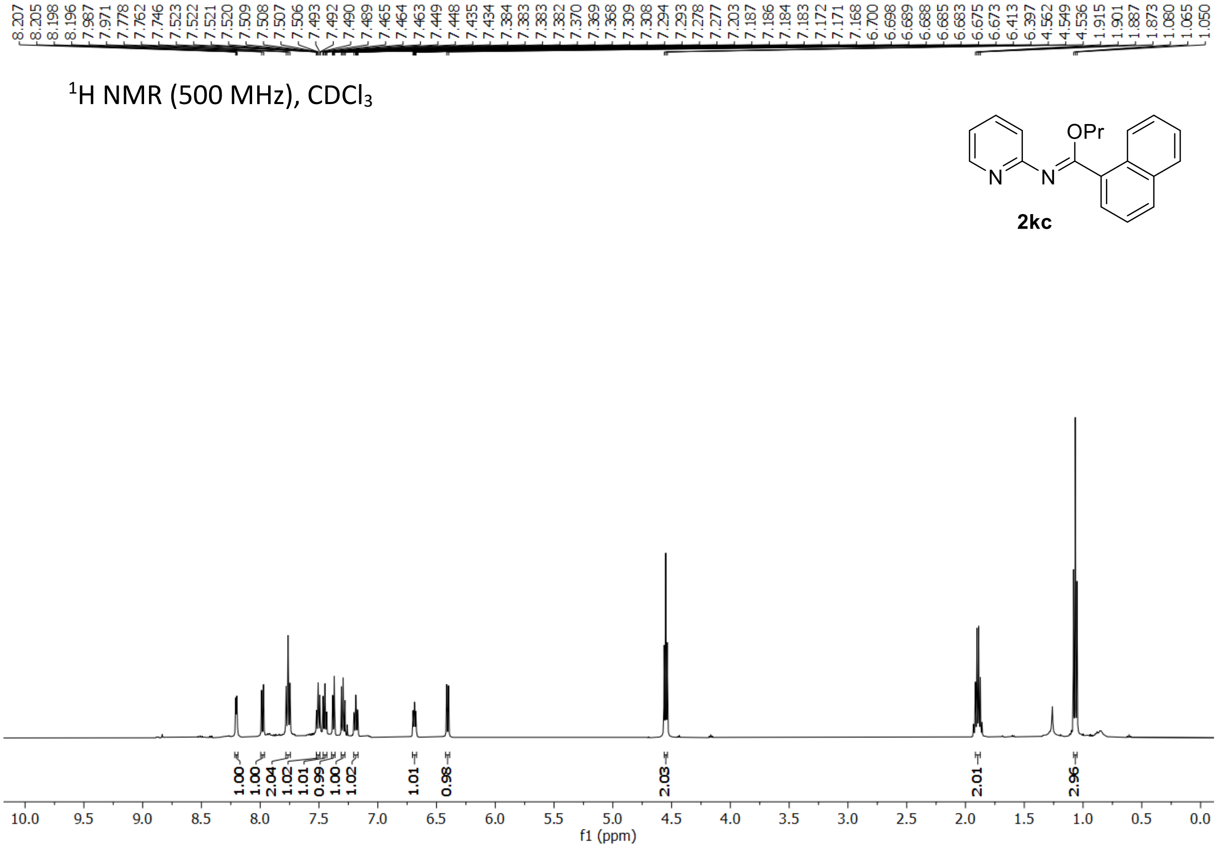

$^{13}C\{H\}$ NMR (125 MHz), $CDCl_3$

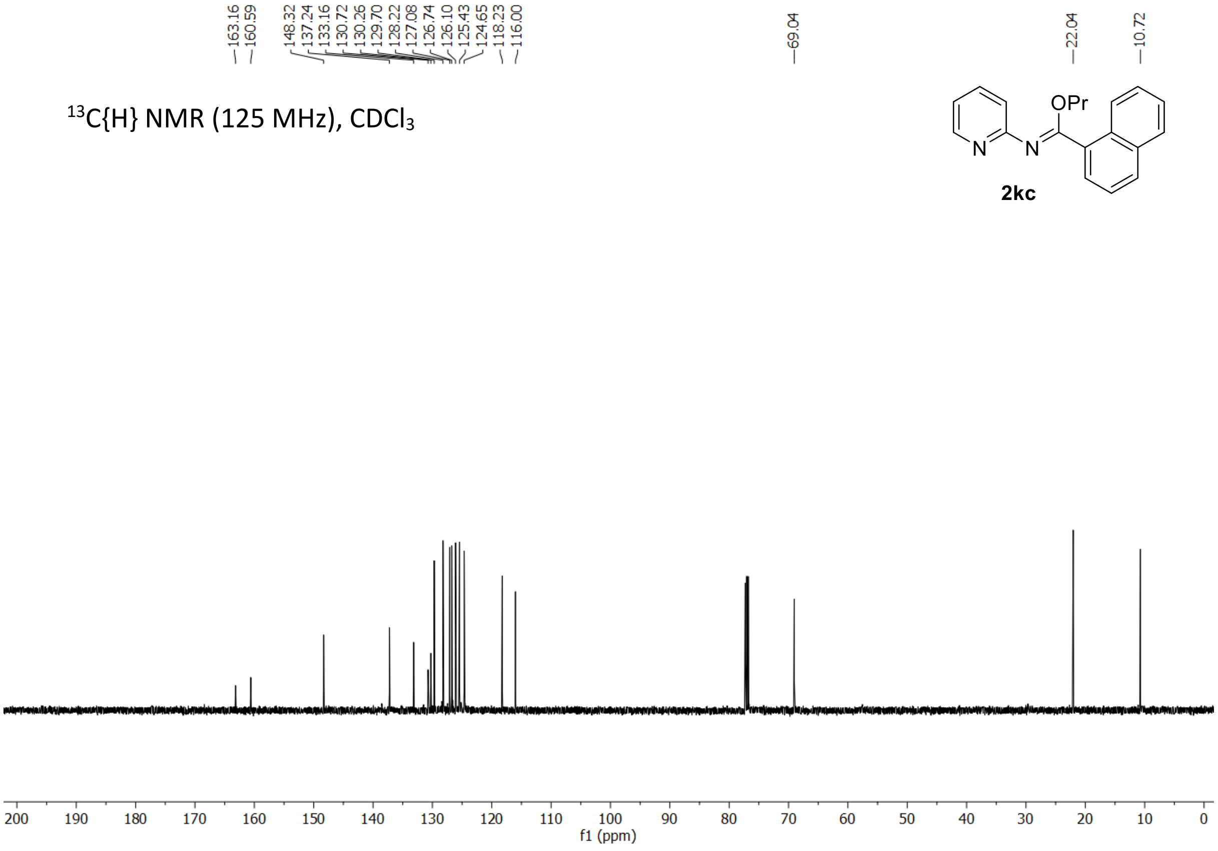

^{1}H NMR (500 MHz), $CDCl_3$

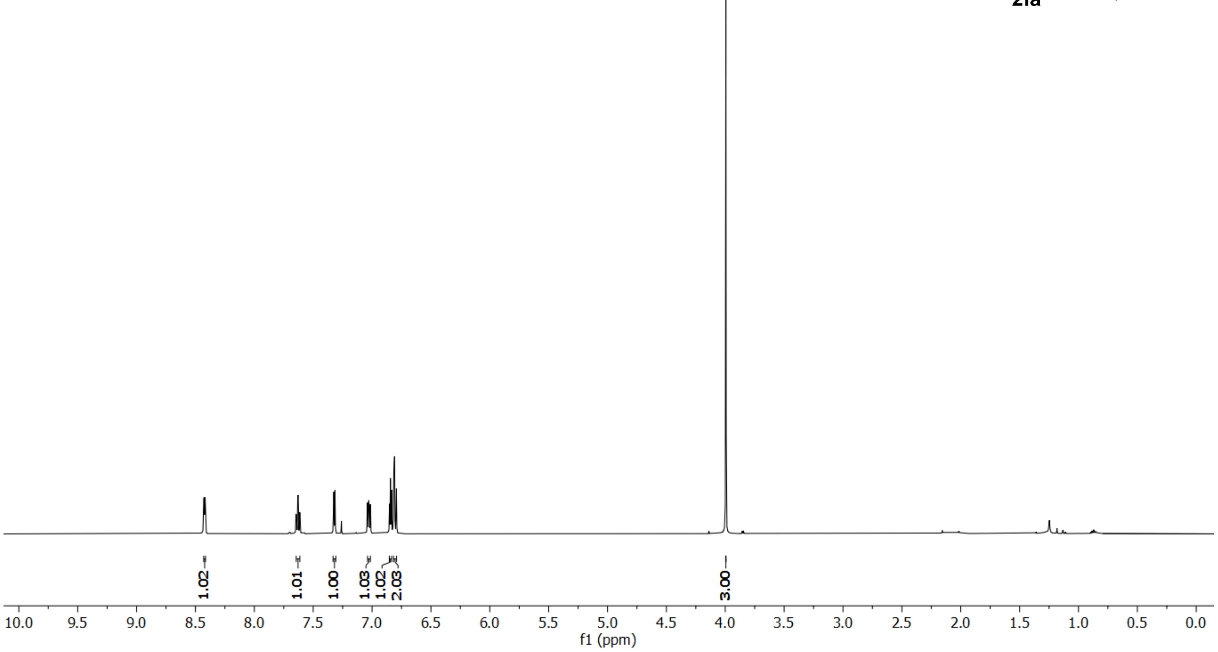

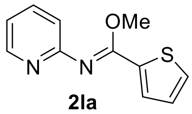

2la

$^{13}C\{H\}$ NMR (125 MHz), $CDCl_3$

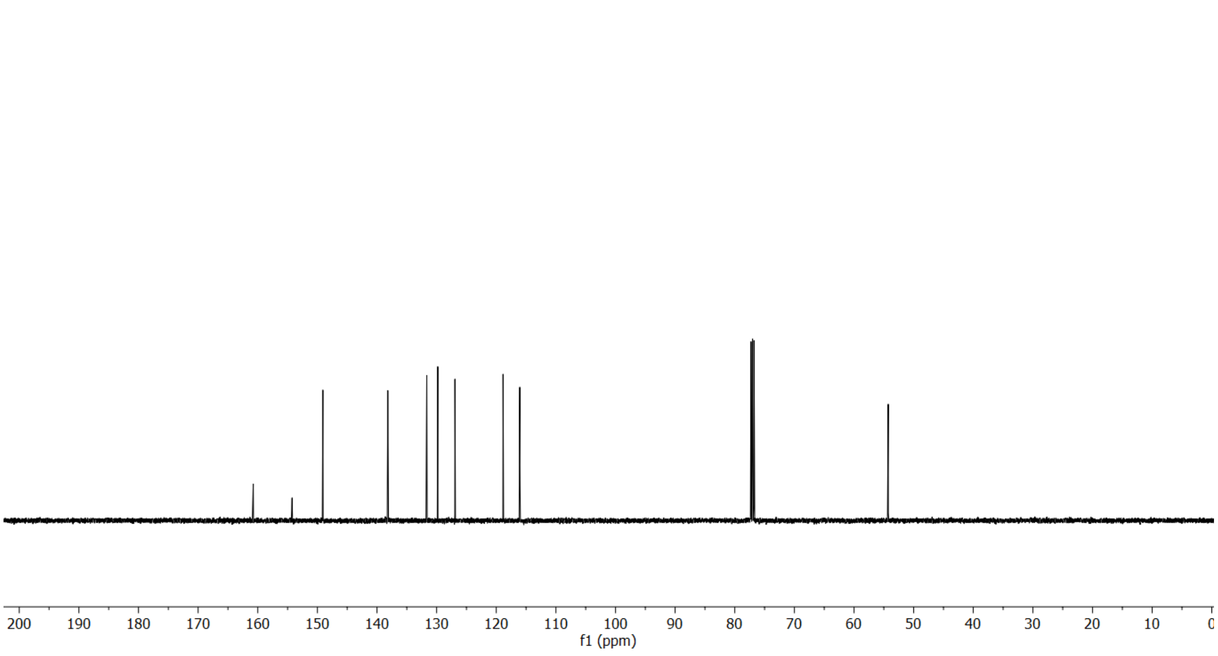

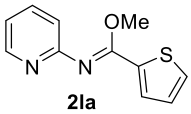

2la

^{1}H NMR (500 MHz), $CDCl_3$

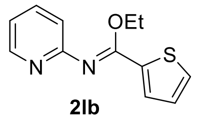

2lb

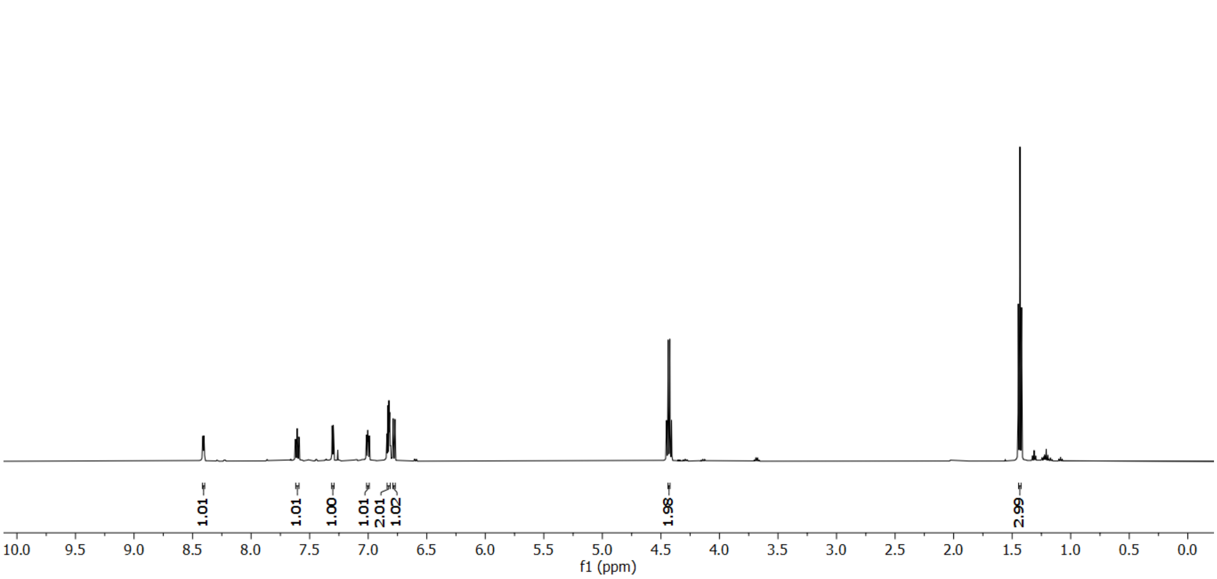

$^{13}C\{H\}$ NMR (125 MHz), $CDCl_3$

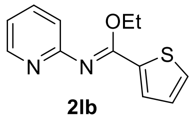

2lb

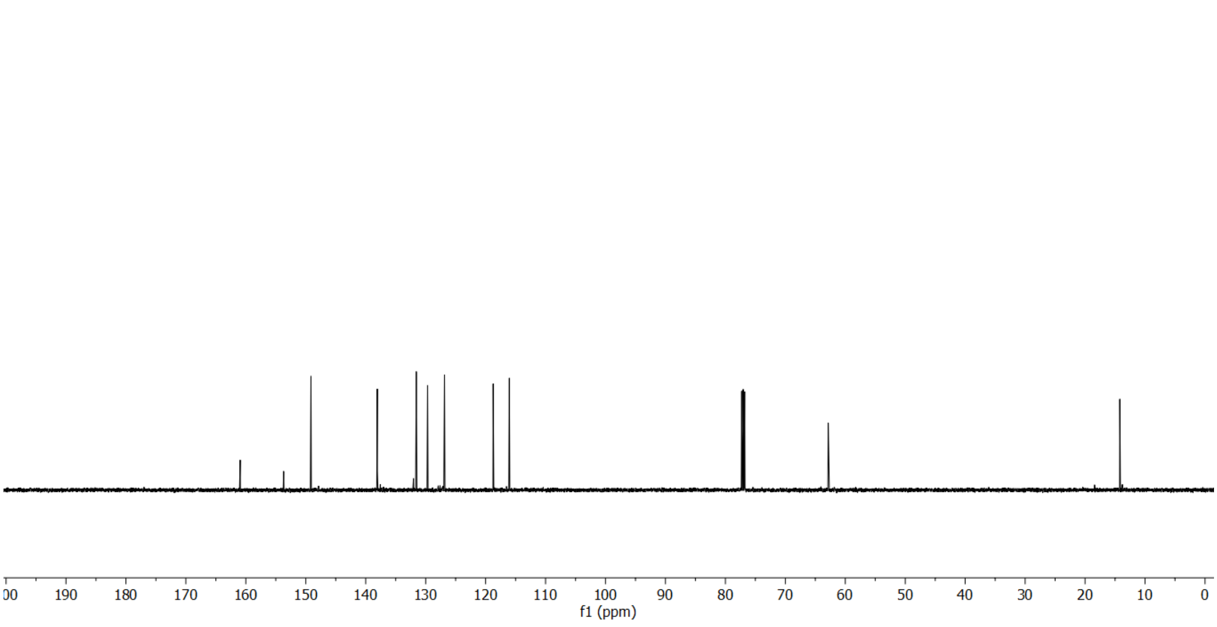

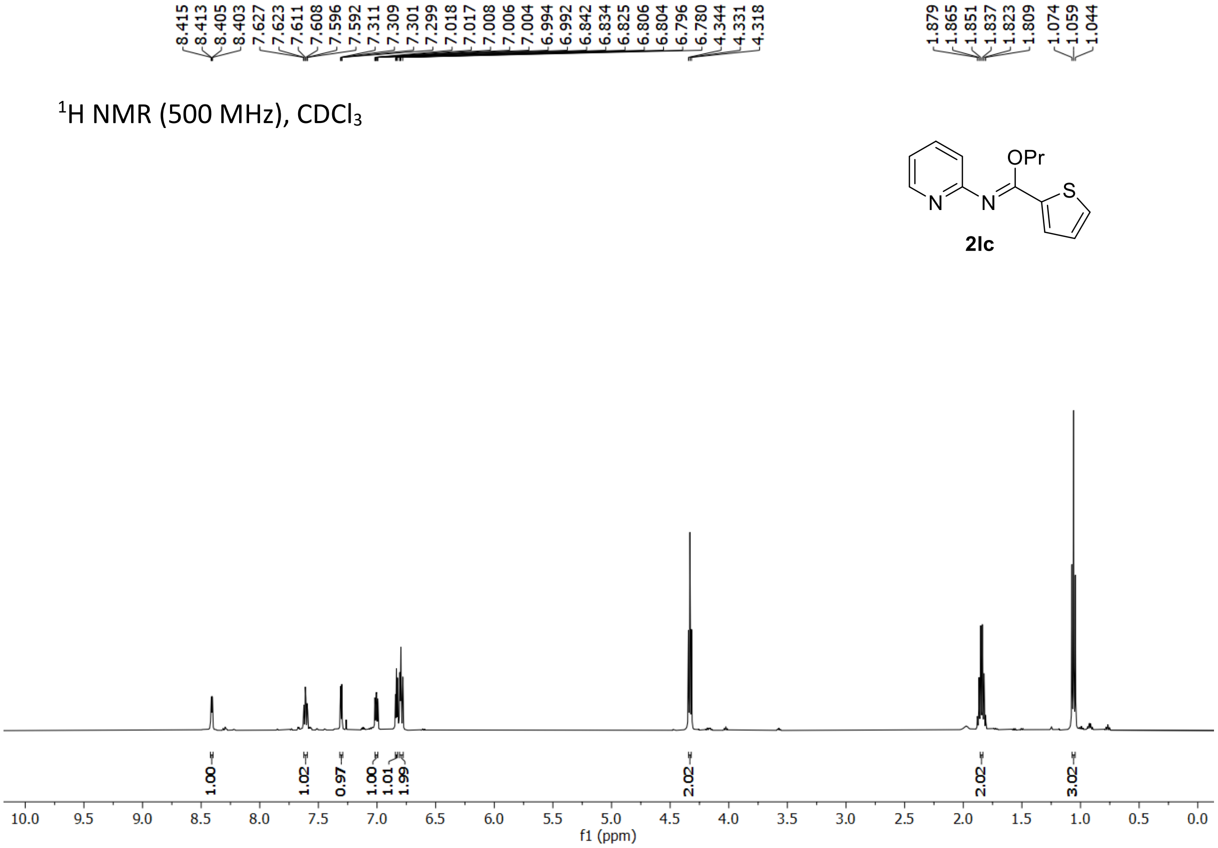
8.415
8.413
8.405
8.403
7.627
7.623
7.611
7.608
7.596
7.592
7.311
7.309
7.301
7.299
7.018
7.017
7.008
7.006
7.004
6.994
6.992
6.842
6.834
6.825
6.806
6.804
6.796
6.780
4.344
4.331
4.318
1.879
1.865
1.851
1.837
1.823
1.809
1.074
1.059
1.044
^{1}H NMR (500 MHz), $CDCl_3$
OPr
N
N
S
2lc
1.00
1.02
0.97
1.00
1.01
1.99
2.02
2.02
3.02
10.0 9.5 9.0 8.5 8.0 7.5 7.0 6.5 6.0 5.5 5.0 4.5 4.0 3.5 3.0 2.5 2.0 1.5 1.0 0.5 0.0
f1 (ppm)

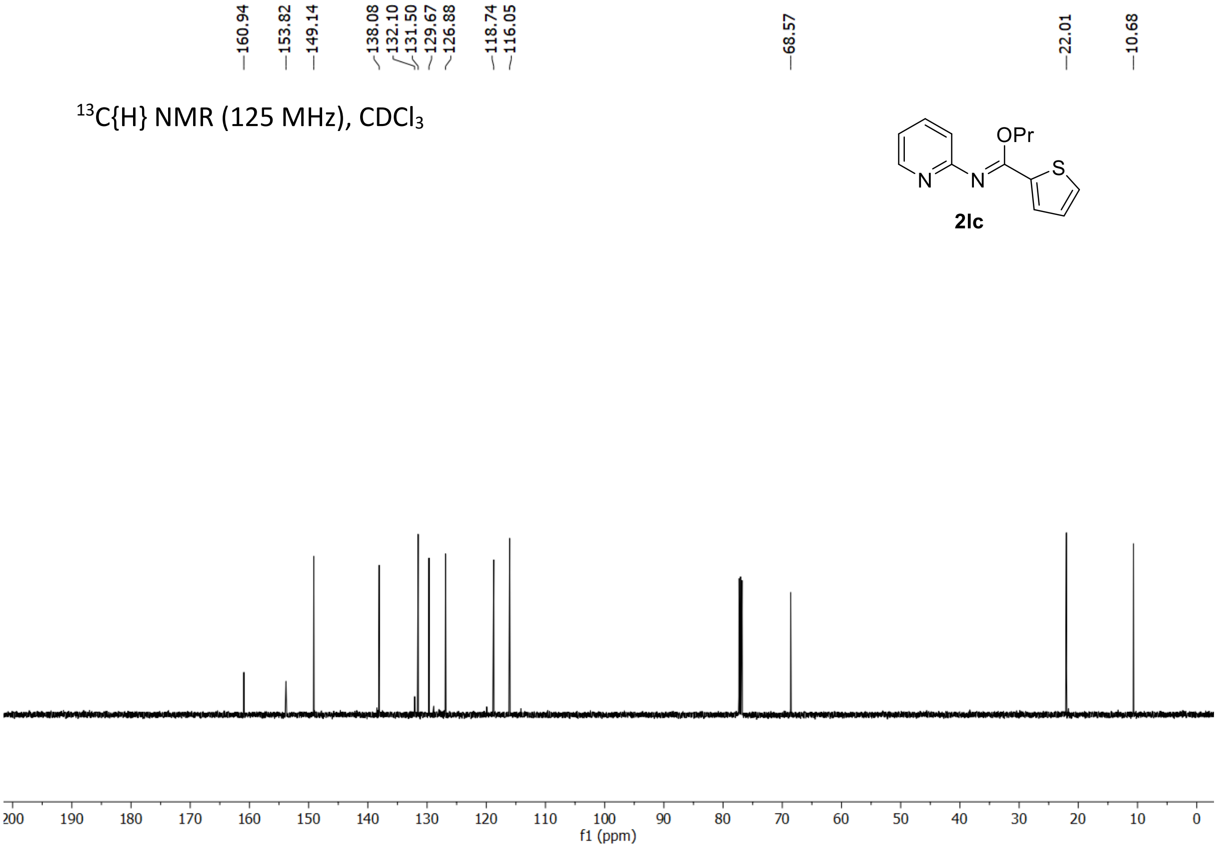
160.94
153.82
149.14
138.08
132.10
131.50
129.67
126.88
118.74
116.05
68.57
22.01
10.68
^{13}C{H} NMR (125 MHz), $CDCl_3$
OPr
N
N
S
2lc
200 190 180 170 160 150 140 130 120 110 100 90 80 70 60 50 40 30 20 10 0
f1 (ppm)

^{1}H NMR (500 MHz), $CDCl_3$

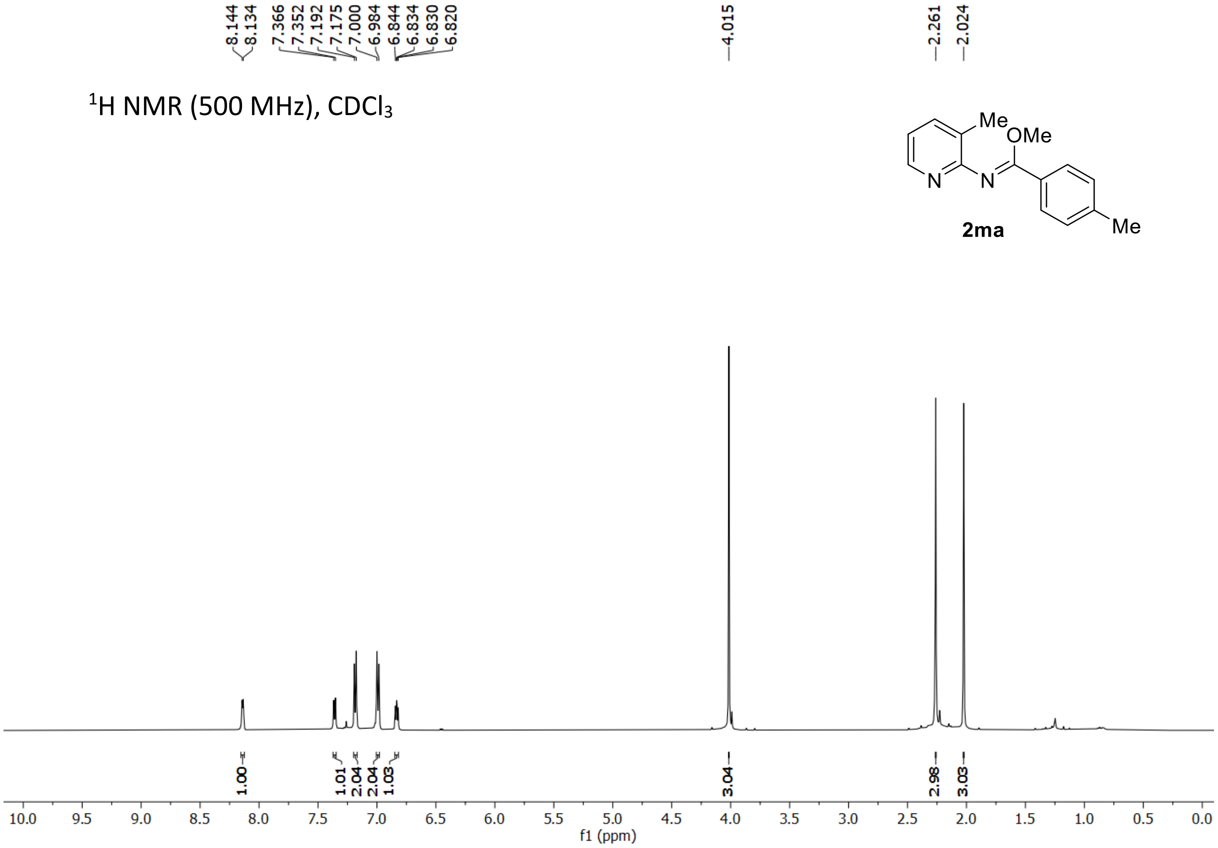

$^{13}C\{H\}$ NMR (125 MHz), $CDCl_3$

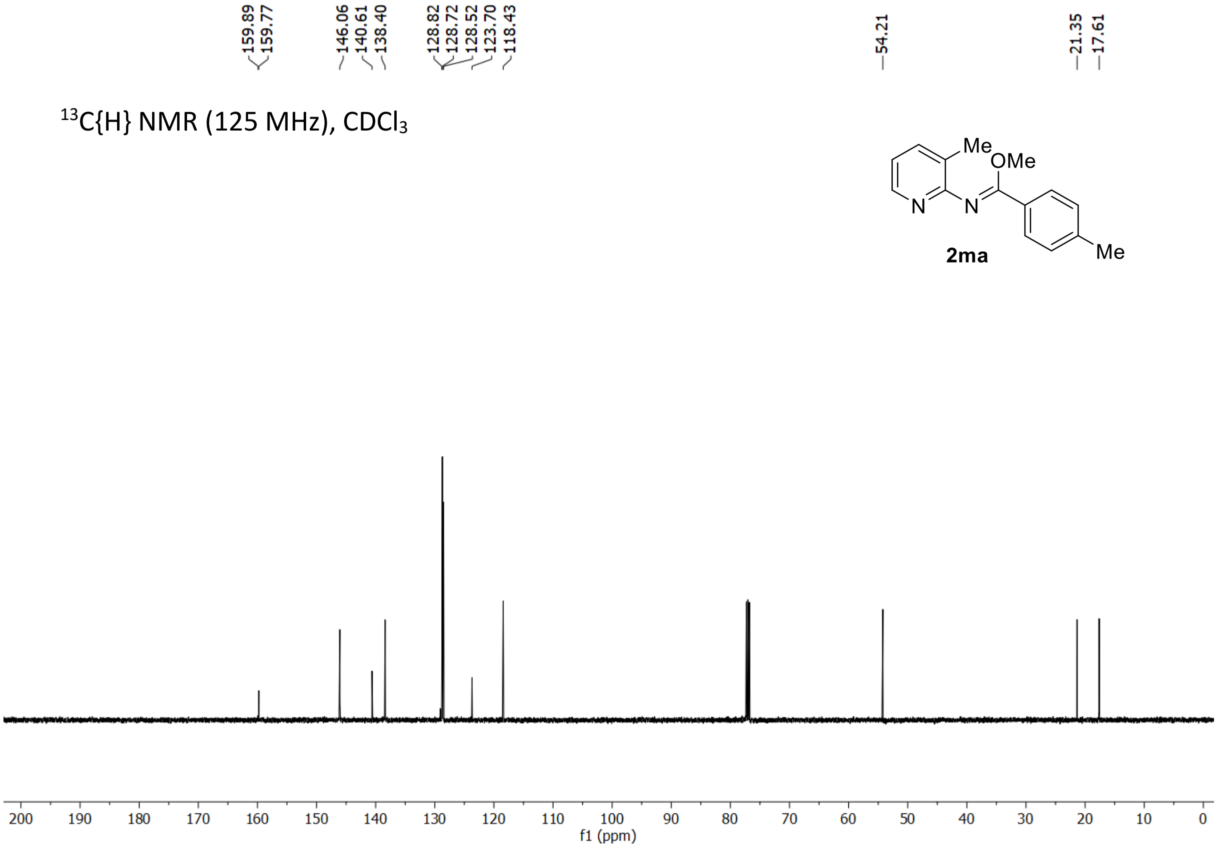

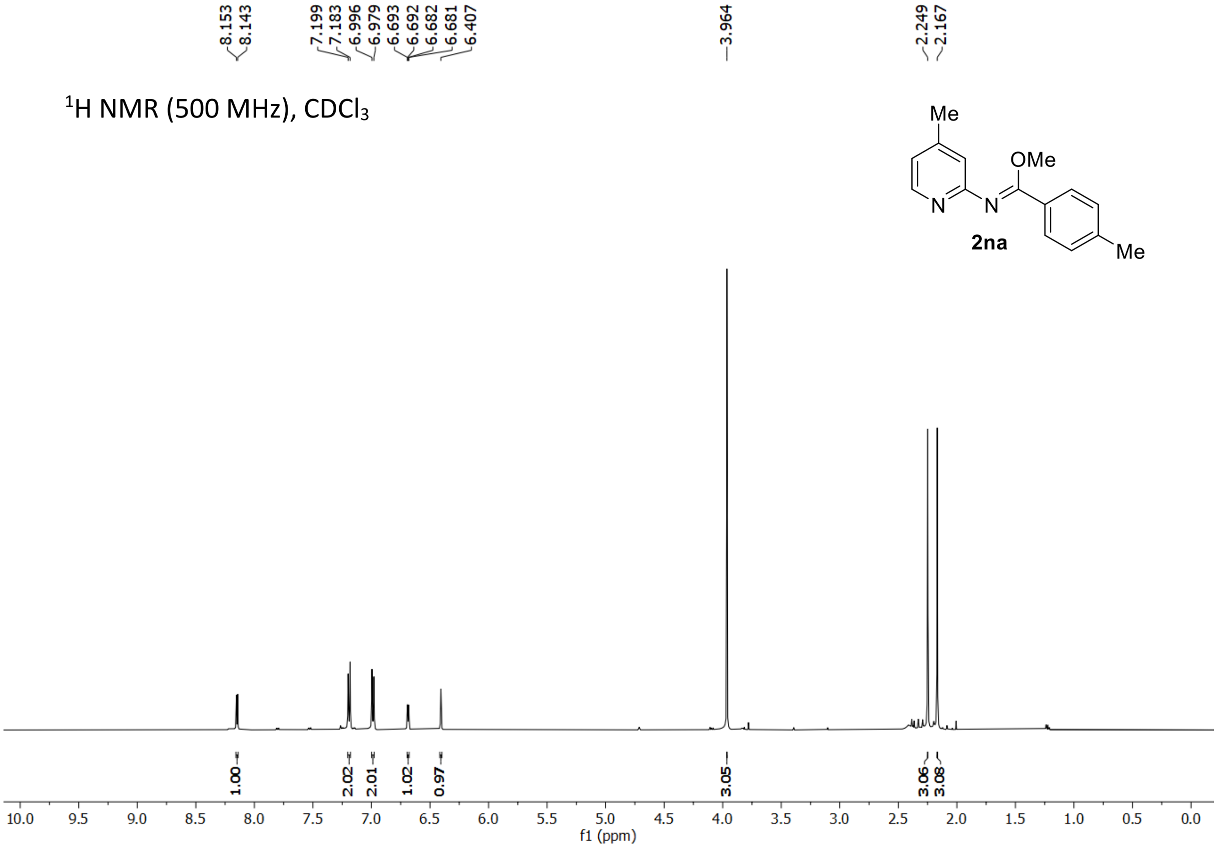
8.153
8.143
7.199
7.183
6.996
6.979
6.693
6.692
6.682
6.681
6.407
3.964
2.249
2.167
^1H NMR (500 MHz), $CDCl_3$
Me
OMe
N
N
2na
Me
1.00
2.02
2.01
1.02
0.97
3.05
3.06
3.08
f1 (ppm)

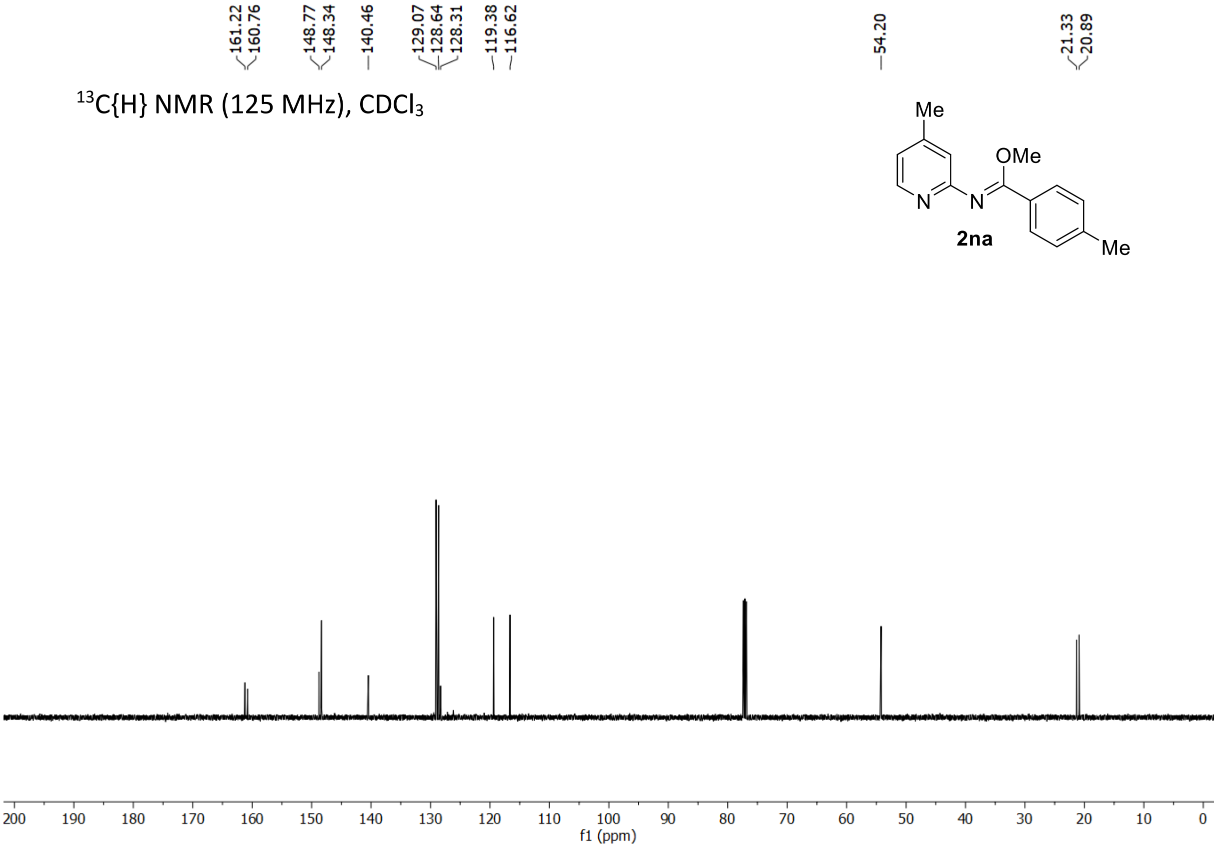
161.22
160.76
148.77
148.34
140.46
129.07
128.64
128.31
119.38
116.62
54.20
21.33
20.89
$^{13}C\{H\}$ NMR (125 MHz), $CDCl_3$
Me
OMe
N
N
2na
Me
f1 (ppm)

^{1}H NMR (500 MHz), $CDCl_3$

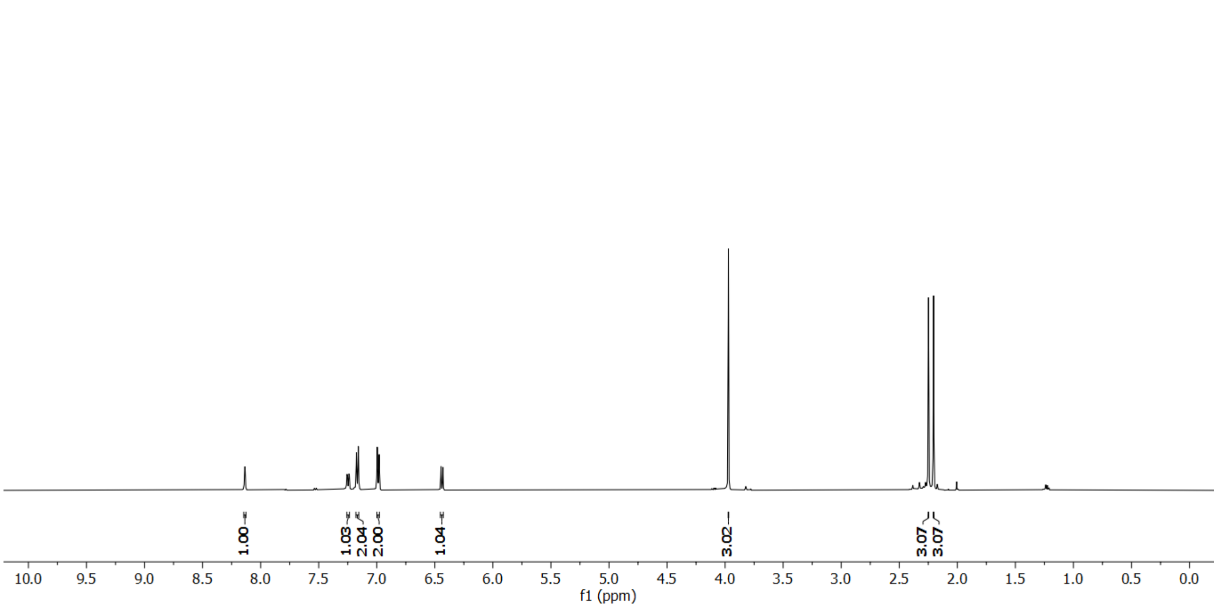

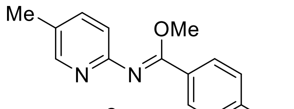

2oa

$^{13}C\{H\}$ NMR (125 MHz), $CDCl_3$

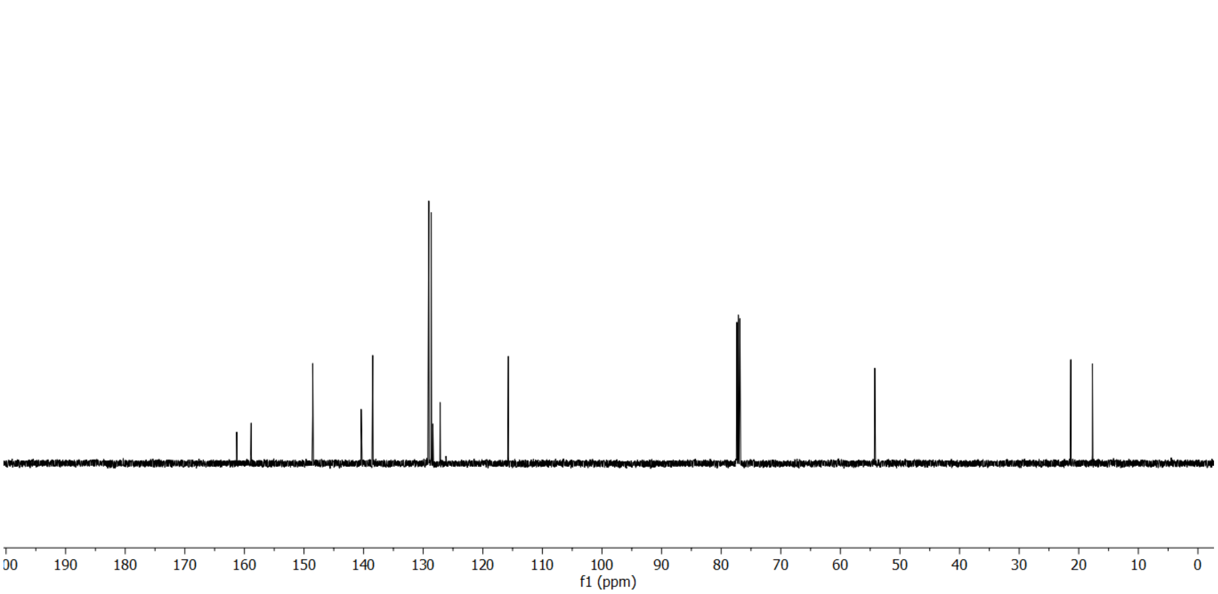

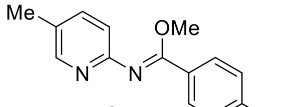

2oa

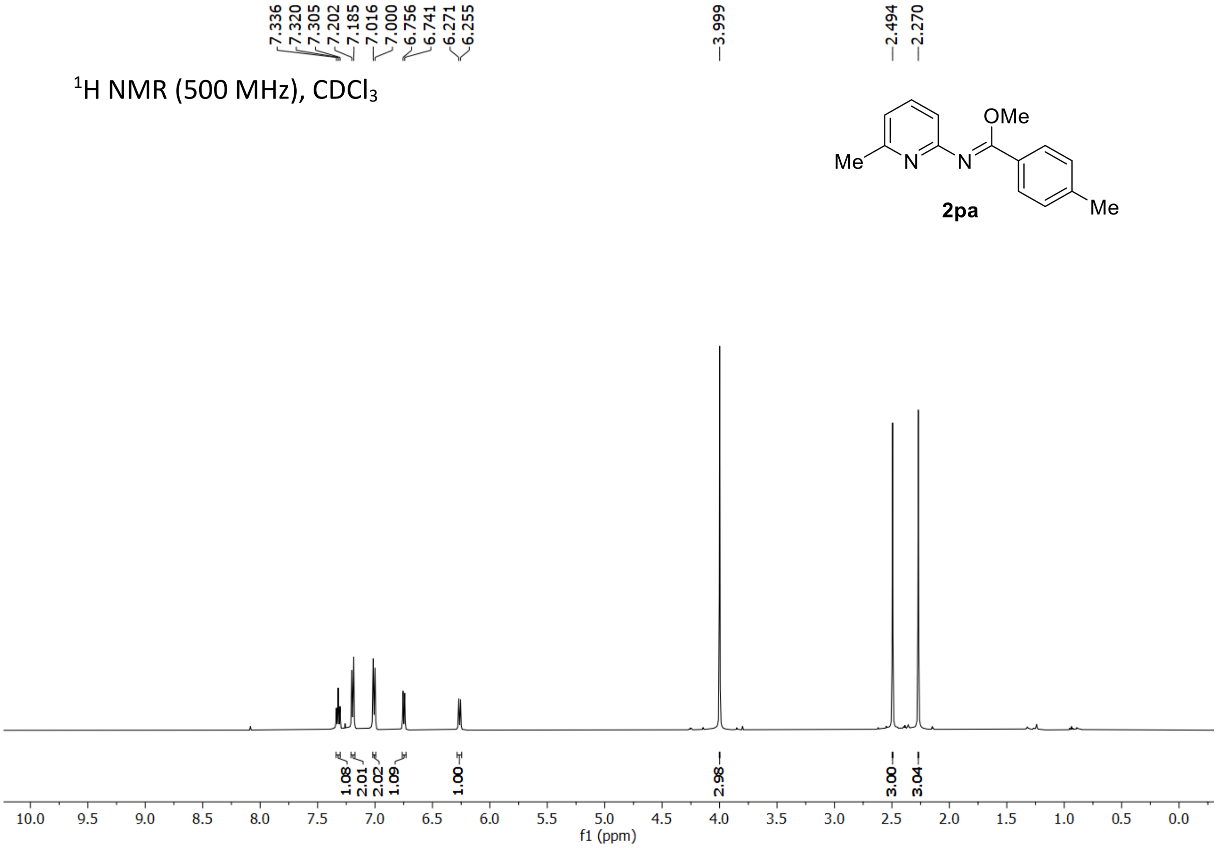

7.336
7.320
7.305
7.202
7.185
7.016
7.000
6.756
6.741
6.271
6.255
3.999
2.494
2.270
1H NMR (500 MHz), CDCl3
OMe
Me
N
N
Me
2pa
1.08
2.01
2.02
1.09
1.00
2.98
3.00
3.04
10.0 9.5 9.0 8.5 8.0 7.5 7.0 6.5 6.0 5.5 5.0 4.5 4.0 3.5 3.0 2.5 2.0 1.5 1.0 0.5 0.0
f1 (ppm)

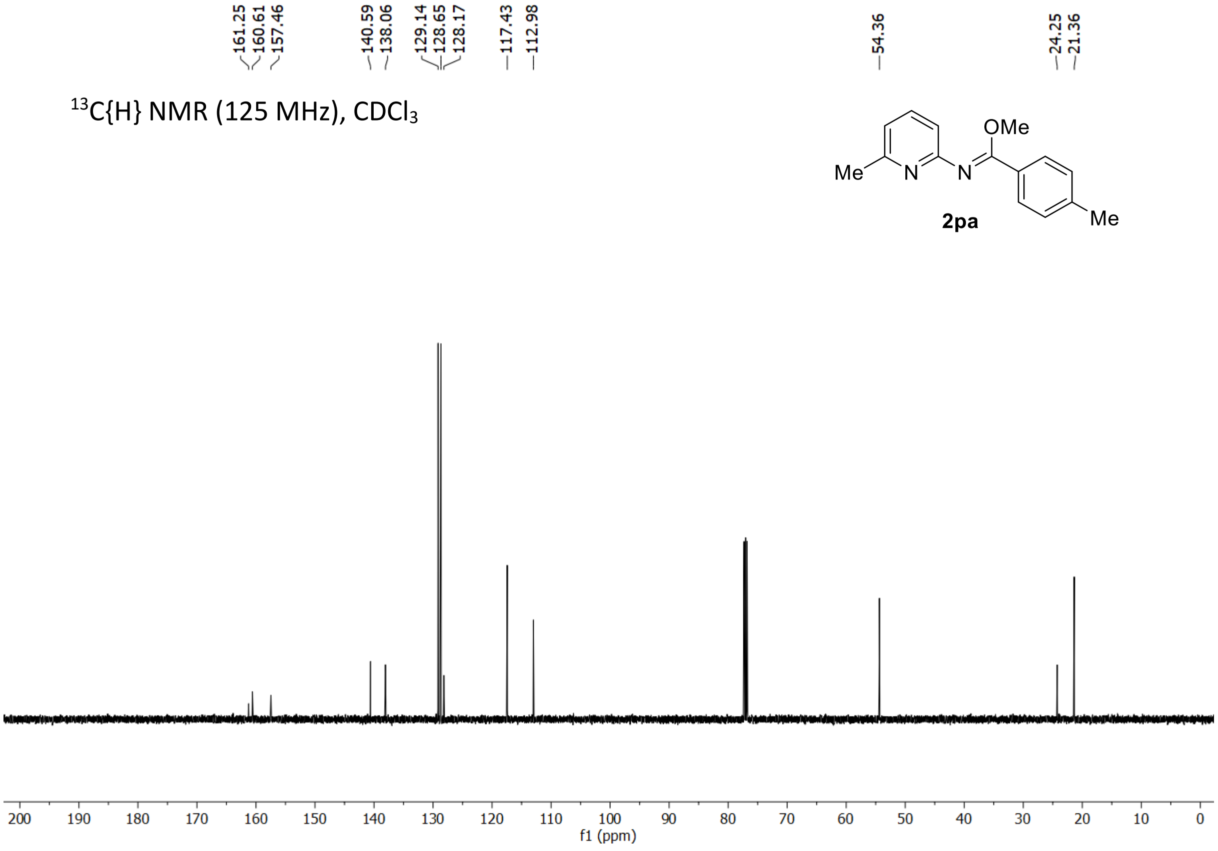

161.25
160.61
157.46
140.59
138.06
129.14
128.65
128.17
117.43
112.98
54.36
24.25
21.36
13C{H} NMR (125 MHz), CDCl3
OMe
Me
N
N
Me
2pa
200 190 180 170 160 150 140 130 120 110 100 90 80 70 60 50 40 30 20 10 0
f1 (ppm)

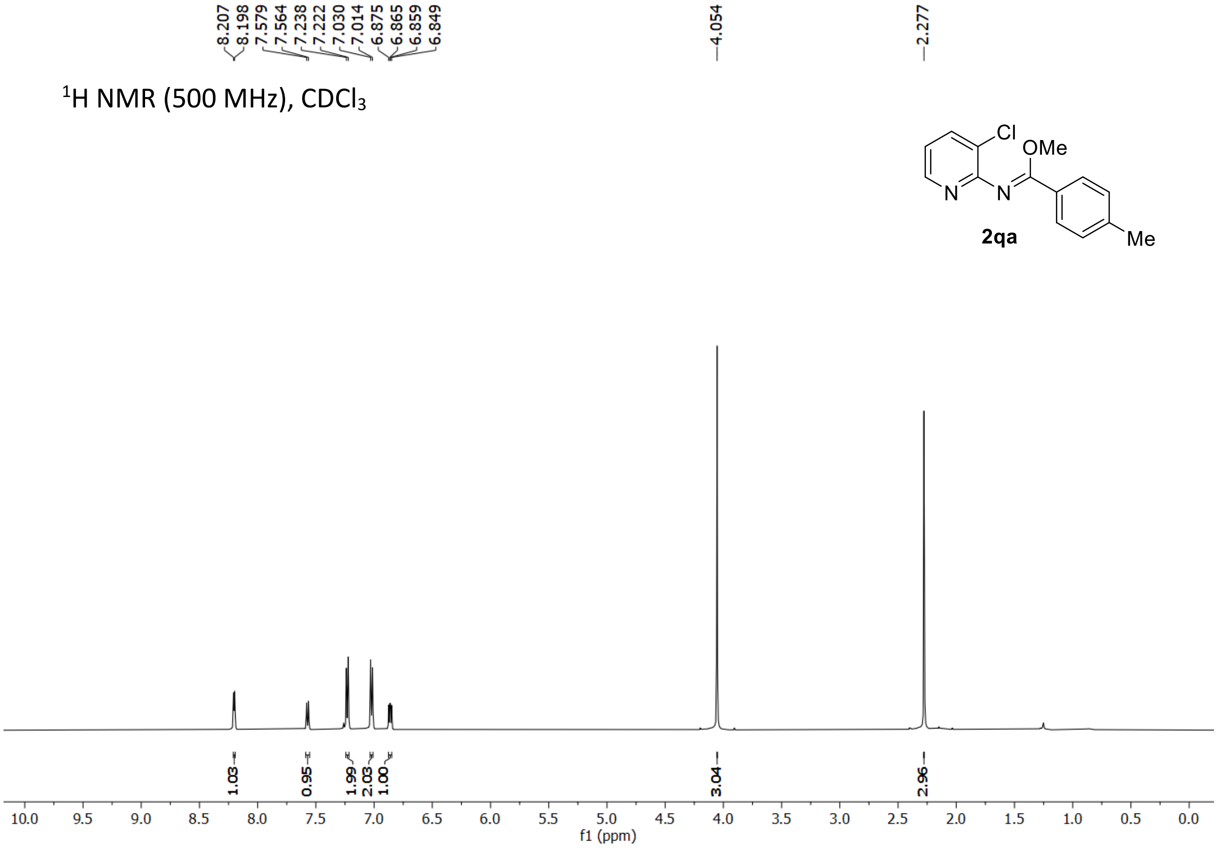
8.207
8.198
7.579
7.564
7.238
7.222
7.030
7.014
6.875
6.865
6.859
6.849
4.054
2.277
^{1}H NMR (500 MHz), $CDCl_3$
Cl
OMe
N
N
Me
2qa
1.03
0.95
1.99
2.03
1.00
3.04
2.96
f1 (ppm)

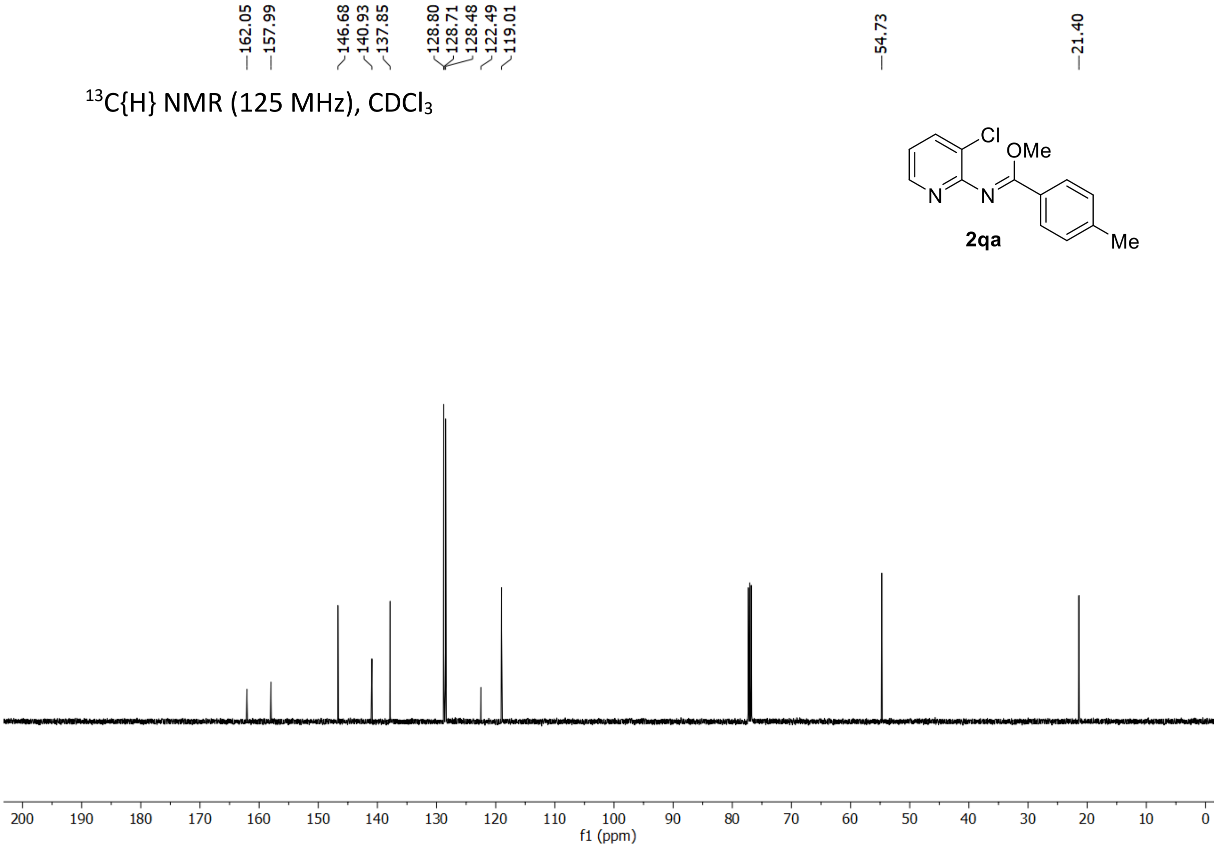
162.05
157.99
146.68
140.93
137.85
128.80
128.71
128.48
122.49
119.01
54.73
21.40
$^{13}C\{H\}$ NMR (125 MHz), $CDCl_3$
Cl
OMe
N
N
Me
2qa
f1 (ppm)

^{1}H NMR (500 MHz), $CDCl_3$

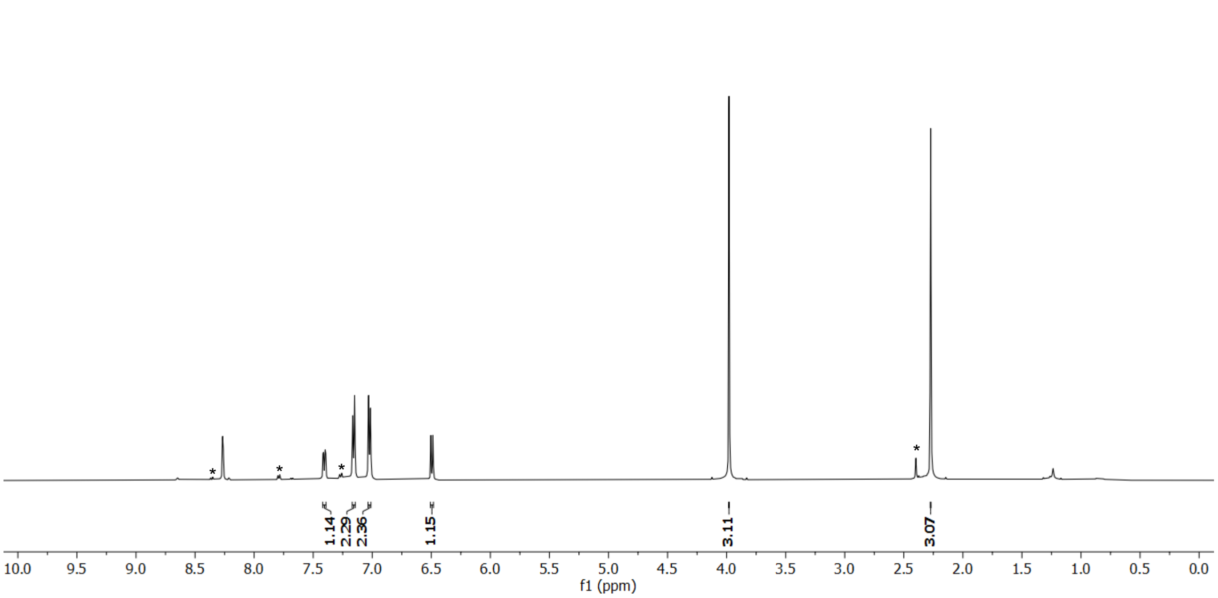

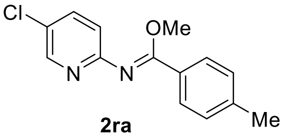

2ra

$^{13}C\{H\}$ NMR (125 MHz), $CDCl_3$

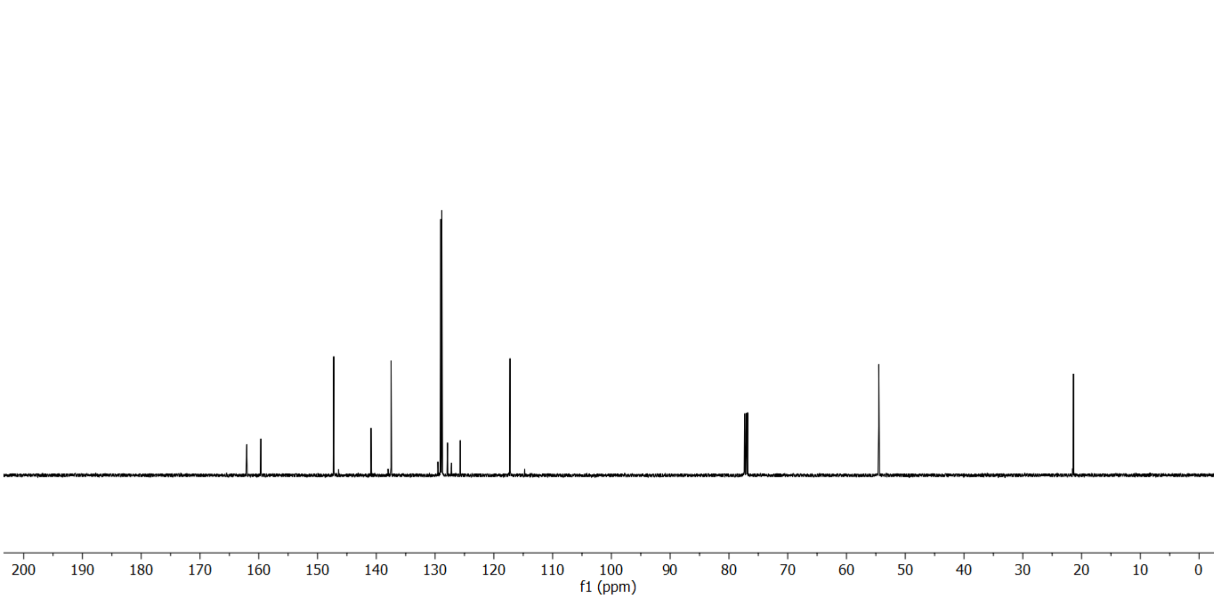

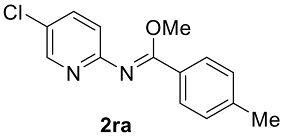

2ra

*corresponding amide

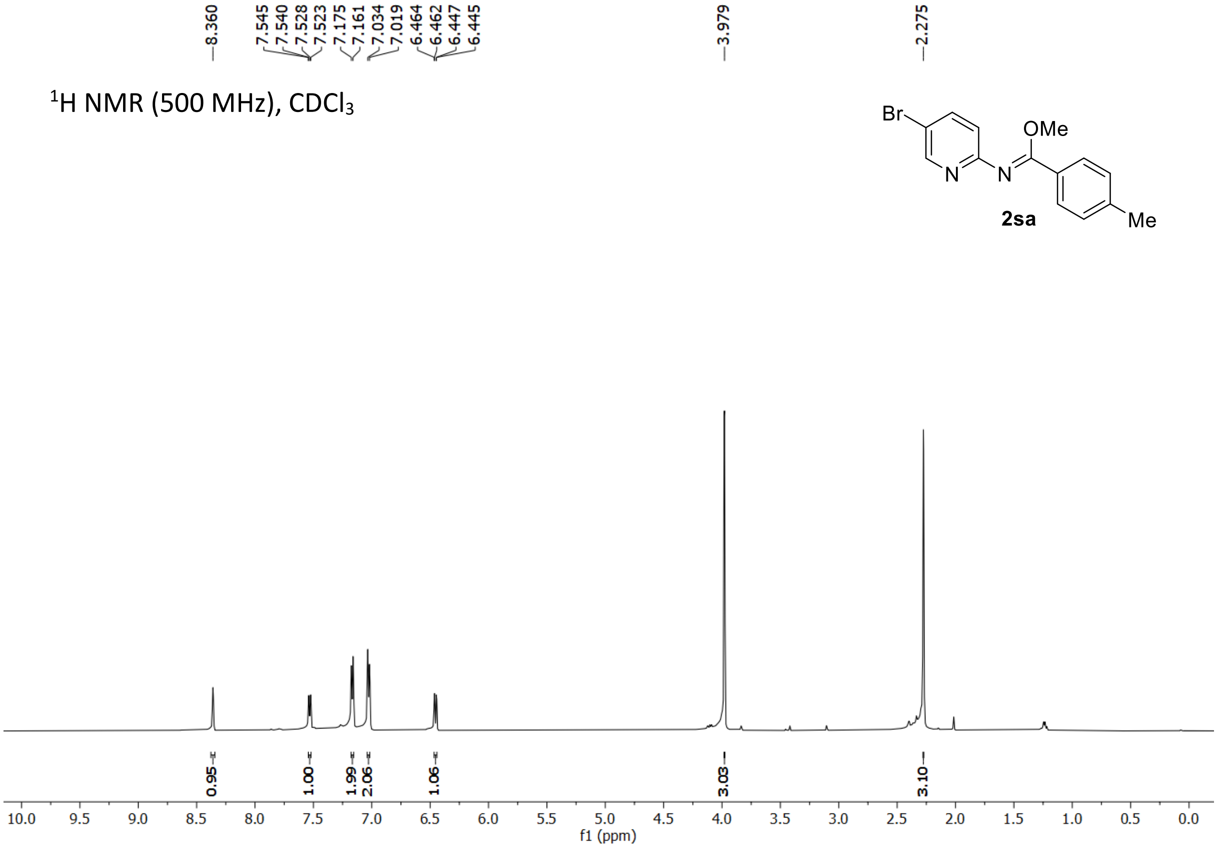
8.360
7.545
7.540
7.528
7.523
7.175
7.161
7.034
7.019
6.464
6.462
6.447
6.445
3.979
2.275
^{1}H NMR (500 MHz), CDCl$_3$
Br
OMe
N
N
2sa
Me
0.95
1.00
1.99
2.06
1.06
3.03
3.10
10.0 9.5 9.0 8.5 8.0 7.5 7.0 6.5 6.0 5.5 5.0 4.5 4.0 3.5 3.0 2.5 2.0 1.5 1.0 0.5 0.0
f1 (ppm)

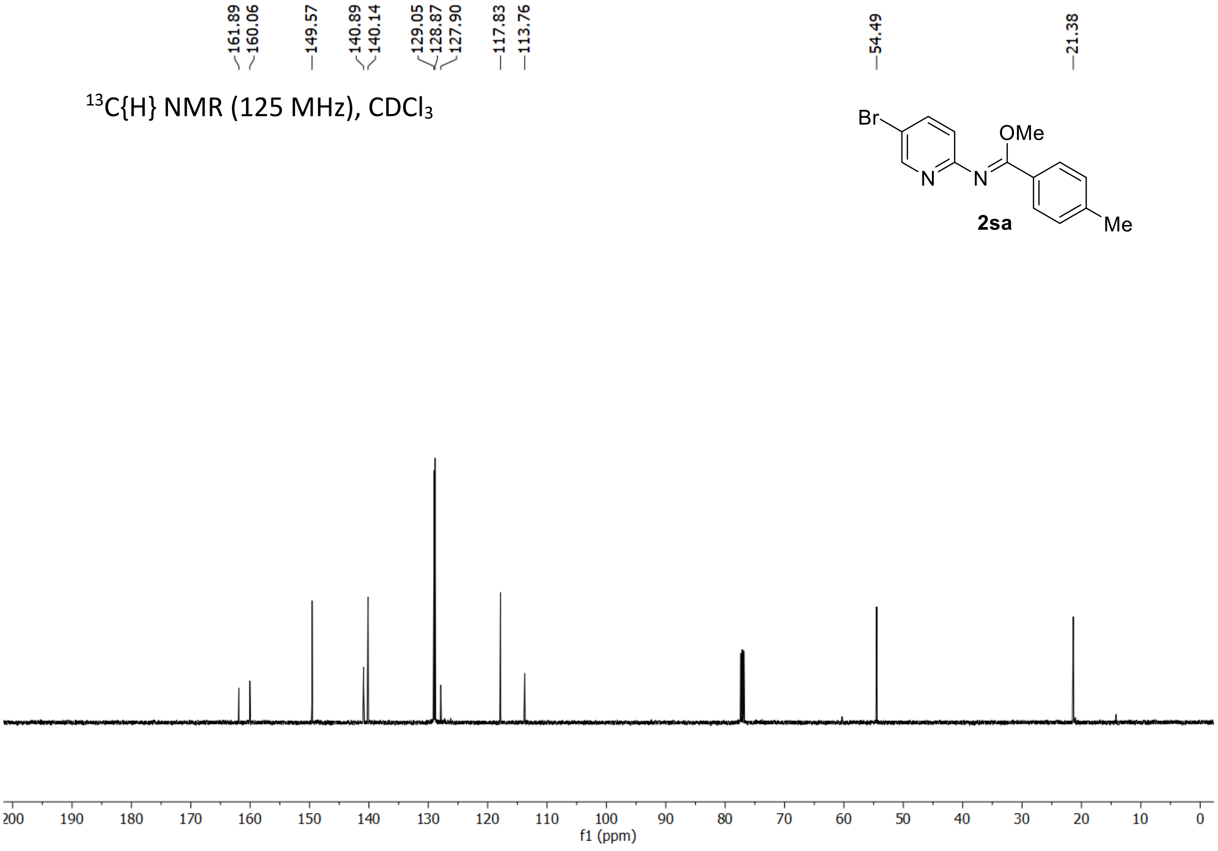
161.89
160.06
149.57
140.89
140.14
129.05
128.87
127.90
117.83
113.76
54.49
21.38
^{13}C{H} NMR (125 MHz), CDCl$_3$
Br
OMe
N
N
2sa
Me
200 190 180 170 160 150 140 130 120 110 100 90 80 70 60 50 40 30 20 10 0
f1 (ppm)
